# Individual and structural barriers to Latin American refugees and asylum seekers' access to primary and mental healthcare in Chile: A qualitative study

**DOI:** 10.1371/journal.pone.0241153

**Published:** 2020-11-06

**Authors:** Alejandra Carreño-Calderón, Baltica Cabieses, M. Eliana Correa-Matus

**Affiliations:** Programa de Estudios Sociales en Salud, Instituto de Ciencias e Innovación en Medicina (ICIM), Facultad de Medicina Clínica Alemana, Universidad del Desarrollo (UDD), Santiago, Chile; Centre for Sexual Health & HIV/AIDS Research, ZIMBABWE

## Abstract

**Background:**

Since 2010 there has been a growing population of refugees and asylum seekers in Latin America. This study sought to investigate the perceived experiences and healthcare needs of refugees and asylum seekers of Latin American origin in Chile in order to identify main barriers to healthcare and provide guidance on allied challenges for the public healthcare system.

**Methods:**

Descriptive qualitative case study with semi-structured interviews applied to refugees and asylum seekers (n = 8), healthcare workers (n = 4), and members of Non-Governmental Organizations and religious foundations focused on working with refugees and asylum seekers in Chile (n = 2).

**Results:**

Although Chilean law guarantees access to all levels of healthcare for the international migrant population, the specific healthcare needs of refugees and asylum seekers were not adequately covered. Primary care and mental healthcare were the most required types of service for participants, yet they appeared to be the most difficult to access. Difficulties in social integration -including access to healthcare, housing, and education- upon arrival and lengthy waiting times for legal status of refugees also presented great barriers to effective healthcare provision and wellbeing. Healthcare workers and members of organizations indicated the need for more information about refugee and asylum-seeking populations, their rights and conditions, as well as more effective and tailored healthcare interventions for them, especially for emergency mental healthcare situations.

**Conclusions:**

All participants perceived that there was disinformation among institutional actors regarding the healthcare needs of refugees and asylum seekers in Chile. They also perceived that there were barriers to access to primary care and mental healthcare, which might lead to overuse of emergency services. This study highlights a sense of urgency to protect the social and healthcare needs of refugees and asylum seekers in Latin America.

## Introduction

Protection of the rights of refugees and asylum seekers is a global task with urgent pending challenges [1]. Nowadays, the Latin American region is facing massive movements of people from Venezuela, as well as from other countries like Colombia, Haiti and Dominican Republic, who often claim refugee or asylum seeker status [2,3]. However, little is known about who those refugee and asylum seekers in Latin America really are, and their particular social and healthcare needs.

In Chile, migration has increased in recent decades, along with growth of refugee and asylum applications and the increase in waiting times for visa regulation [4,5]. In terms of asylum applications, according to data provided by the Ministry of Interior and Public Security, a total of 3,850 asylum applications were made between 2000 and 2010, while between 2010 and 2018 the number increased to 15,664 requests, which implies a growth of more than 400% for the entire period, equivalent to an average annual increase of 45%. Regarding applicant nationality, the Colombian community represents 45% of all applications in the last ten years, followed by 28% of Cuban nationals and 21% Venezuelans [6]. A Chilean refugee law [7] was passed in 2010 and established a new broad framework of protection for asylum seekers. It set out to adapt the existing regulations to facilitate implementation of the Geneva Convention. However, a decade after its promulgation, several problems have been reported in the application of this law, resulting in faults in the application process and in the implementation of its core principles [8]. Little research has been conducted in relation to the living conditions and healthcare needs of asylum seekers in the South American region [9,10].

One of the lesser known aspects of the refugee and asylum seeker application process has to do with their healthcare needs and existing access to healthcare services in the country of destination. In Chile, the public healthcare system is organized in primary healthcare networks nationwide, which are closely connected to secondary healthcare (specialist care including mental care) and tertiary healthcare systems (hospital care). Hence, primary care is the first contact between patients and the healthcare system, as it looks after health promotion, health prevention and lower complexity cases.

The right to public healthcare cover through FONASA (Chilean National Health Service) has been guaranteed to all regular migrants, refugees and asylum seekers [11]. However, several studies [12–14] have revealed that the effective use of healthcare services by international migrants in Chile is highly determined by the ability to overcome a number of technical, administrative and cultural barriers [14,15].

Considering that several studies carried out in Chile have revealed several violations of the humanitarian protection system available for migrants, only a few of them have focused especially on refugees and asylum seekers [16]. This study sought to investigate the perceived experience and healthcare needs of refugees and asylum seekers of Latin American origin in Chile. The focus of the study is particularly related to individual and structural barriers to Latin American refugees and asylum seekers' access to primary and mental healthcare in Chile.

## Methods

### Design and setting

We had the collaboration of several organizations to perform this study: A religious Non-Governmental Organization (NGO) Fundación Ayuda Social de las Iglesias Cristianas (FASIC), the academic organization Universidad Diego Portales (UDP), the international agency The United Nations High Commissioner for Refugees (UNHCR) and a number of municipalities in the Metropolitan Region of Santiago, Chile. Municipalities located in the Metropolitan Region were chosen because this region concentrates 63% of international immigrants in Chile, including refugees [17]. The study was conducted over six months, from August 2018 to January 2019.

### Sampling and recruitment procedures

The sampling strategy was intentional and reasoned, looking for criteria of representativeness of discourse, experience and meaning of the case study [18,19]. Recruitment criteria were established based on the characteristics of the qualitative paradigm [20], which aimed to cover the heterogeneity of the asylum experience and its relationship with healthcare scenarios, based on differentiated criteria for the three populations or perspectives of interest: A) population of asylum seekers, B) Public healthcare teams with experience in caring for this population, C) Staff of institutions responsible for recognition of international protection (Table 1).

**Table 1:** General socio-demographic characteristics of participants.

Universe	Sample	Country of origin	Sex
Refugees and asylum seekers	2 in the process of asylum seeker’s status assessment	Venezuela	F
		Venezuela	M
	2 with recent refugee status approved	Colombia	F
		Venezuela	F
	4 with long-term status of refugee	Colombia	M
		Colombia	F
		Peru	F
		Peru	F
Healthcare workers treating refugees and asylum seekers	4 primary care workers and healthcare authorities	Chile	M
		Peru	F
		Chile	F
		Chile	M
Chilean organizations which provide assistance to refugees and asylum seekers	1 NGO (FASIC)	Chile	F
	1 Faculty of Law (UDP)	Chile	F

For asylum seekers, the sample was selected based on theoretical and practical criteria. The theoretical criterion was the time spent as a refugee or asylum seeker in Chile, considering two persons in the process of asylum seeker´s status assessment, 2 with recent refugee status approved and 4 with long-term (more than 1 year) status of refugee.

It was not possible to access cases in which the refugee or asylum seeker request had been rejected. On the other hand, the practical criterion considered the feasibility of contacts and mediated recruitment from contact with NGO FASIC. This non-probabilistic sampling was selected given the unique study group and potential sensible topics to be discussed with them during data collection [21]. All participants could refuse to be involved or quit during the research process. The general criteria for inclusion in the study were: i) being a refugee or asylum seeker immigrant; ii) being at least 18 years old; iii) being of Latin American nationality; iv) having had a previous experience with the Chilean healthcare system.

For healthcare teams, a selective sampling strategy was also used, through a theoretical criterion that they provided healthcare treatment for asylum seekers. The teams were contacted directly by the researchers. The criteria for inclusion in the study were: i) belonging to a public healthcare team and ii) having experience working with refugee or asylum populations. Participants in this group regularly attended immigrant population, including refugees and asylum seekers. It must be noted that there is no mechanism available in the country for healthcare providers to distinguish between general migrants and refugees or asylum seekers, except for self-report. Hence, based on their testimonies, all healthcare workers selected in this study declared having experience attending migrants who defined themselves as refugees and asylum seekers. All participants included in this sub-group belonged to the primary healthcare system and had worked there for at least two years at the time of the study.

The members of organizations in charge of protecting this type of population were also selected based on a selective sample criterion and the theoretical criteria that were part of any of the institutions dedicated to the advice and recognition of the condition of asylum. Recruitment was made based on contacts established with the academic, social and healthcare institutions involved in the study. The two organizations that contributed to the recruitment of participants (FASIC and UDP) are the most experienced ones in supporting and accompanying refugees in Chile. The criteria for inclusion in the study were: i) belonging to a team of organizations linked to the international protection process ii) having experience working with asylum populations. Respondents included in this sub-group had been working for more than a year in social and legal orientation of people in a situation of refuge or asylum seeking in Chile. The study finally included a total of 14 participants, all adults from the Santiago Metropolitan region.

### Data collection, processing, and analysis

All semi-structured interviews were conducted by the principal investigator (AC) and a research assistant in Spanish. The interviews were audio-recorded, transcribed into Spanish and a thematic analysis of the information was carried out. All quotes were translated from Spanish into English by the authors (BC) and translated back into Spanish in order to check language and cultural consistency.

### Ethical considerations

The study was approved by the Ethics Committee at Universidad del Desarrollo on 23 July 2018 (#23/10-2018). Written informed consent, including for audio recording, was obtained from all participants. Confidentiality was safeguarded by the first author. Names and other personal details were removed from transcripts before analysis. Participants who were refugees or asylum seekers were reimbursed a US$5 bus fare.

## Results

A total of 14 participants were interviewed. Table 1 displays their general socio-demographic characteristics. We also present the main issues that emerged from interviews.

### Refugees’ poor knowledge of healthcare system and rights

Overall, refugees and asylum seekers were completely unaware of how the healthcare system in Chile works and did not have access to it until such time as a specific need arose, such as a health condition or a health emergency. *“I used to feel very worried about the idea that the girls could get sick (…)*. *However*, *we came with pending orthodontic treatments (…) and a lady offered us a plan of attention to all and we were happy*. *But of course*, *then we realized that it was very expensive*. *(…)*. *When I started working*, *I found out that as refugees we had the right to FONASA [public system] and that they [private system] had practically robbed us*.*” (W1*, *Refugee*, *woman)*.

In terms of healthcare, information on migrants' access to health had partially reached the healthcare teams, and although many of them had been trained, they still faced obstacles to providing care to these migrants. *“We do a lot of training with the [healthcare] teams but they always tell you that there was a case that they could not enter into the formal system*, *because they did not have Chilean ID card (…)” (W3*, *Nurse*, *woman)*.

Nearly all refugees or asylum seekers reported having sought healthcare in primary care structures and having been denied their right to healthcare due to submitting expired identity documents or documents related to the request for refuge. Although refugee applicants have the right to access health, many of them report not knowing how to become formal beneficiaries of the healthcare system in Chile. *“At the beginning*, *because of our health issue*, *we did it through FONASA (…)*. *But about two months ago*, *my daughter had a backache*, *went to the healthcare center but was not treated*. *They said she was un-registered or that the document was expired*. *I did not understand well*, *but they did not attend her*, *(…) then we paid*, *in pharmacy we asked them to give us something and that was what we did until the pain disappeared*.*” (M1*, *refugee*, *man)*.

Most respondents were unaware that access to healthcare is guaranteed as a human right in Chile, regardless of the situation in which their asylum application is found. For this reason, they generally did not complain when they were rejected from the public healthcare system and often went to private centers with direct out-of-pocket payments for health attention.

### Disregard of the Refugee Law by Chilean stakeholders

On the other hand, the two NGO employees identified difficulties in providing information both regarding the refugee and asylum application process, and regarding healthcare access. Although the Refugee Law was approved in 2010, the most critical issue that interviewees seemed to agree on was that there had not been a systematic implementation process for this law, and highlighted some barriers. These barriers included but were not limited to meeting specific conditions to apply or giving the wrong information. *“I believe that the situation today is very complex (*…*) at least from our perspective*, *which is the legal perspective*, *there are a number of obstacles in the asylum procedure that occur from the beginning*, *right*? *(…) and that has repercussions on everything*, *on the ability of the person to find work*, *to access health*, *(…) the basic rights*.*” (W2*, *Lawyer*, *woman)*.

### Lack of appropriate mental health services

Although in Chile the mental healthcare system has been integrated into primary care since 1993, mental healthcare needs of this specific population were reportedly hardly covered by public services, registering a loss of referrals in the case where the problems had been investigated in primary care, or simply lacking care due to the lack of Information and trust. In contrast to what happens with respect to healthcare in the period preceding forced migration, the majority of respondents reported having felt needs not covered under mental healthcare in Chile, which were related to episodes of distress, sleep disorders, emotional crises, despair, loneliness and even suicidal ideation.

*“That is all for us who are here*, *at first one is afraid*, *you will know that a person is Colombian because they are always afraid*. *Then the fear passes and the longing remains*, *not being able to return*… *and there we must know how to endure no more*, *pray*, *don't let crying beat you*.*” (W1*, *refugee*, *woman)*.

The lack of mental healthcare, or access to specialists through the referral system gave rise to a search for alternatives that could not been maintained over time, such as university-based psychology clinics, or sporadic care in the private system. These types of interventions rather point at the treatment of symptoms or controlling the crisis, but they have little chance of becoming a long-term therapeutic process. This was explained by the testimonies of respondents who were in psychiatric treatment in either a university clinic that closed its care facilities or one foundation's psychologist. *“I treated myself with a psychiatrist*, *because I was in treatment for depression*, *for the problem of post-traumatic stress*. *There were some psychiatrists from the University (…)*. *They treated me super well*, *excellent*, *but then they closed and I was left with nothing”*. *(W1*, *refugee*, *woman)*.

Another respondent stated: *“I’ve never looked for a psychologist or any other kind of support*. *Just FASIC provides us with yoga and the opportunity to talk to other people like us”(W5 applicant*, *woman)*. Yet another respondent maintained: *We are depressed*, *we are longing*. *The longing I think has more to do with the stability one had before*. *I have here a partner and colleague*, *a friend and colleague*, *who told me*: *I am eating badly*, *I did not eat badly*. *Of course*, *she is longing for the stability she had*… *but it is the longing*, *it is longing to be calm*. *That is*, *I have had*, *say an emotional carousel because*, *precisely*, *one is not there [in his country]*. *(W6 applicant*, *woman)*.

## Discussion

This study explored individual and structural barriers to Latin American refugees and asylum seekers' access to primary and mental healthcare in Chile. Participants’ experiences and opinions demonstrated the importance of mental health among refugees and asylum seekers, and the lack of instruments available to teams to support those in need of services. It is recognized that referral systems for mental health are not always effective, so that people "get lost" or "drift" and the difficulties that teams have to attend to a population whose experiences of violence, fear, remoteness with emotional ties and abrupt loss of security for political reasons, put them in a particular condition against which the usual psychotherapeutic instruments do not seem to be effective enough [22].

The identification of barriers to access to primary care and mental healthcare are important results of our research. Key issues identified were firstly the important barriers to access to primary healthcare, most of them connected with the lack of information on the international protection system to which Chile adheres. Second, mental healthcare needs of this population are hardly covered by public services, so when in need of emergency treatment, they tend to look for temporary care within the private healthcare system.

Barriers reported by applicants and institutional agents have been corroborated by the Ministry of Domestic Affairs, reporting that of the total number of requests made between the years 2010 to 2018, 74% were granted a temporary visa in an average waiting time of eight months [6]. The temporary visa application process in Chile can be repeated indefinitely. This causes uncertainty during the application waiting times for a new visa, which incidentally also limits applicant access to healthcare.

Economic problems, lack of job opportunities, as well as other barriers to heath care access already mentioned, have become social determinants that place refugees in a vulnerable position [23]. Hence, it should be noted that current regulations have created exceptional conditions for these people to be waiting endlessly [24,25]. This predicament affects their civil rights and transfers the responsibilities of the State to civil society, such as NGOs and religious foundations. These organizations in Chile, as in other migratory contexts, [26–28] are replacing some of the State's social and healthcare responsibilities. Ironically, it is organizations in civil society who are implementing the Chilean Refugee Law, but not the State. This situation is also worrying regarding the way the public healthcare system works. Neglecting the specific needs and rights of refugees and generating barriers to their healthcare access results in hospital and emergency services being overburdened. This hinders the proper functioning of the national health care system and has been reported in other countries as well [29].

Finally, from our results there is a need to discuss what we have called the *specificity of the needs of the refugee population and its consequences in healthcare*. Although the boundaries between the needs of migrants and refugees are permeable, the experiences of asylum seekers and refugees documented here have an important vulnerability component in areas that directly impact health conditions in general, such as exposure to violence physical and psychological, torture or imprisonment, and mental health in particular [30,31]. That is why a relevant recommendation that emerges from this study is the organization of a multi-sectoral system capable of making visible and prioritizing the specific needs of this population stands out, as well as avoiding situations of re-victimization that are taking place whenever the conditions of access to social rights of these people are not guaranteed. Similarly, in relation to effective access to the healthcare system, it is necessary for the sector to assume the challenges of including this segment of the population, especially in the field of primary care and mental healthcare. To achieve this, it is required not only to reduce the levels of misinformation regarding their right of access to health, but also to install in the existing healthcare model a focus on intercultural health, capable of welcoming the characteristics of this population and needs that are created from a flight marked by violence, so as to be able to instill resilience for better healthcare outcomes.

As this study was exploratory in nature, it was possible to reach saturation just for some dimensions of interest: 1) barriers to healthcare access 2) necessities in mental healthcare and 3) barriers related to the application process. For the other dimensions, further data collection is necessary [32]. The number of participants recruited corresponded to a theoretical sampling of an exploratory study that aimed at approaching a phenomenon understudied in Chile, preparing the ground for future studies that might establish a structured sense of relationships between variables.

## Conclusion

This study should be considered as a first approach to the healthcare situation of refugees and asylum seekers in Chile. It highlights the main individual and structural barriers these people currently encounter to access the healthcare and international protection system in Chile. As far as we know, this is the first study of its kind to describe testimonies of refugees and asylum seekers in relation to their healthcare needs in Chile. It also reports on experiences and concerns from the point of view of healthcare workers and members of NGOs who work to uphold the social, cultural and legal integration of refugees and asylum seekers in Chile. Our study considers a rather small sample of the populations identified in it, which may be regarded as a weak point, together with some degree of bias due to the recruitment process. Considering the migration flow in Chile occurs through its northern border, other regional studies are needed. Likewise, the specific mental healthcare needs of refugees and asylum seekers, and resources available by the public mental healthcare system need further research.

## Supporting information

S1 File Interview guides. (PDF)

S2 File Transcripciones completas. (PDF)
