# Supplementary material for: Individual and structural barriers to Latin American refugees and asylum seekers' access to primary and mental healthcare in Chile: A qualitative study
Source: PLoS One. 2020 Nov 6;15(11):e0241153. doi: 10.1371/journal.pone.0241153 (PMC7647080; doi:10.1371/journal.pone.0241153)
Supplement: S1 File — (PDF) [file pone.0241153.s001.pdf]

nexo Nº5: **Instrumentos de recolección de datos**

Tabla 1. ***Distribución de las técnicas por objetivo específico con preguntas directrices***

	TÉCNICA	FOCO DE OBSERVACIÓN / PREGUNTAS DIRECTRICES
**OE 1**<br>**OE 2**<br>**OE 3**	**ETNOGRAFÍA Y OBSERVACIÓN NO PARTICIPANTE**	– Cuáles son las dinámicas de interacción en los espacios de solicitud de asilo<br>– De dónde proviene la información que manejan respecto a la solicitud de asilo<br>– Cuáles son sus vínculos sociales en Chile (familiares, amigos, pareja, connacionales, chilenos)<br>– Percepciones de la relación con las instituciones de protección internacional<br>– Percepciones de violencia institucional<br>– Efectos de la burocracia sobre su acceso a derechos y cobertura de necesidades sociales
	**ENTREVISTAS DE ENFOQUE BIOGRÁFICO NARRATIVO**	– ¿Cómo era su vida en su lugar de origen? ¿Qué recuerdos guarda?<br>– ¿Cómo fue su historia migratoria? ¿Había pensado antes en cambiar de país de residencia, cuándo viajó, por qué y a través de qué medio?<br>– ¿Cómo fue su infancia y sus relaciones familiares?<br>– ¿Cómo ha sido su salud en su vida? ¿cómo y dónde se atendía en su país de origen? ¿se ha atendido en los servicios de salud chilenos?<br>– ¿Por qué se vino a Chile? ¿eligió voluntariamente este país? ¿Hay algo que la sorprendió positiva y/o negativamente de esta elección?<br>– ¿Cómo ha sido la experiencia vivir en Chile? ¿Ha tenido problemas? ¿Dónde ha buscado apoyo o soluciones?<br>– Cuáles son sus trayectorias territoriales y temporales cotidianas (trabajo/casa/ocio/religión)<br>– ¿Qué proyección encuentra en Chile?
**OE 4**	**ENTREVISTAS SEMI-ESTRUCTURADAS**	– ¿Cuál ha sido su experiencia con población refugiada?<br>– ¿Cuáles cree que son hoy las principales necesidades de los solicitantes de asilo y refugiados en Chile?<br>– ¿Qué dificultades enfrentan durante el proceso de solicitud, en el reconocimiento de la condición y la inserción en la sociedad chilena?<br>– ¿De qué manera las instituciones están favoreciendo y/o dificultando el proceso de asilo?<br>– ¿Qué herramientas ha utilizado su institución para atender a esta población?<br>– ¿Qué recursos cree que necesita su institución para mejorar la atención de este tipo de población?
**OE 5**	**ENTREVISTAS SEMI-ESTRUCTURADAS**	– ¿Cuáles son las necesidades de salud que presenta la población refugiada?<br>– ¿Cómo se trata la especificidad que les da o puede dar su condición de refugiados o solicitantes de asilo?<br>– ¿Qué barreras o facilitadores de acceso y de adherencia terapéutica tiene esta población?<br>– ¿Cómo se trabaja el acompañamiento y apoyo en casos de riesgo bio-psico-social?

Pautas de Instrumentos De Investigación

Pauta **de Observación no participante**

<table>
<tr><td>Hora de Inicio:</td><td></td><td>Lugar de Observación:</td><td>Fecha :</td></tr>
<tr><td colspan="4">Nombre de Investigador que Realiza la Observación:</td></tr>
<tr><td>Hora:</td><td>Hechos Objetivos, sucesos y diálogos</td><td colspan="2">Comentarios y Reflexiones</td></tr>
<tr><td></td><td></td><td colspan="2"></td></tr>
<tr><td></td><td></td><td colspan="2"></td></tr>
<tr><td></td><td></td><td colspan="2"></td></tr>
<tr><td></td><td>Practica y Dinámicas cotidianas de personas con antecedentes de refugiados en espacios cotidianos:</td><td colspan="2"></td></tr>
<tr><td></td><td>Vínculos sociales en Chile (familiares, amigos, pareja, connacionales, chilenos)</td><td colspan="2"></td></tr>
<tr><td></td><td>Relación con las instituciones de protección internacional</td><td colspan="2"></td></tr>
</table>

Preguntas e Interrogantes emergentes	Información nueva “posibles respuestas”

Pauta de **Entrevista Biográfica Narrativa** a. Refugiados y Solicitantes de refugio

- ¿Cómo era su vida en su lugar de origen? ¿Qué recuerdos guarda?
- ¿Cómo fue su historia migratoria? ¿Había pensado antes en cambiar de país de residencia, cuándo viajó, por qué y a través de qué medio?
- ¿Cómo fue su infancia y sus relaciones familiares?
- ¿Cómo ha sido su salud en su vida? ¿cómo y dónde se atendía en su país de origen? ¿se ha atendido en los servicios de salud chilenos?
- ¿Por qué se vino a Chile? ¿eligió voluntariamente este país? ¿Hay algo que la sorprendió positiva y/o negativamente de esta elección?
- ¿Cómo ha sido la experiencia vivir en Chile? ¿Ha tenido problemas? ¿Dónde ha buscado apoyo o soluciones?
- Cuáles son sus trayectorias territoriales y temporales cotidianas (trabajo/casa/ocio/religión)
- ¿Qué proyección encuentra en Chile?

Pauta de **Entrevista Semi-Estructurada** a Informantes Claves,

- ¿Cuál ha sido su experiencia con población refugiada?
- ¿Cuáles cree que son hoy las principales necesidades de los solicitantes de asilo y refugiados en Chile?
- ¿Qué dificultades enfrentan durante el proceso de solicitud, en el reconocimiento de la condición y la inserción en la sociedad chilena?
- ¿De qué manera las instituciones están favoreciendo y/o dificultando el proceso de asilo?
- ¿Qué herramientas ha utilizado su institución para atender a esta población?

-¿Qué recursos cree que necesita su institución para mejorar la atención de este tipo de población?

Anexo Nº6: **Hojas Demográficas de Participantes**

Hojas Demográficas.

INSTITUTO DE CIENCIAS E INNOVACIÓN EN MEDICINA
Facultad de Medicina
Clínica Alemana - Universidad del Desarrollo

Programa de Estudios Sociales en Salud

Ser refugiado en Chile:

Trayectorias institucionales y experiencias de salud de solicitantes latinoamericanos de protección internacional

Por favor recuerde que su información es confidencial. Usted puede dejar de responder preguntas en cualquier momento.

Nº	Preguntas	Respuestas
1	¿Cuál es su edad? (años cumplidos)	
2	¿Cuál es su sexo?	a. Femenino<br>b. Masculino<br>c. Otro
3	¿Cuál es su estado Civil?	a. Soltero<br>b. En una relación (no institucionalizada)<br>c. Casado<br>d. Separado<br>e. Divorciado<br>f. Viudo
4	¿Cuál es el nivel más alto de educación que usted ha completado?	a. Básico<br>b. Enseñanza Media<br>c. Técnico<br>d. Profesional Pregrado<br>e. Profesional Posgrado
5	¿Cuál es su ocupación?	
6	¿Se encuentra trabajando?<br>¿Dónde?	
8	¿Cuánto tiempo lleva trabajando en la temática de migración y refugio? (sólo si aplica)	
