# Supplementary material for: Individual and structural barriers to Latin American refugees and asylum seekers' access to primary and mental healthcare in Chile: A qualitative study
Source: PLoS One. 2020 Nov 6;15(11):e0241153. doi: 10.1371/journal.pone.0241153 (PMC7647080; doi:10.1371/journal.pone.0241153)
Supplement: S2 File — (PDF) [file pone.0241153.s002.pdf]

TRANSCRIPCIONES ACTORES INSTITUCIONALES

PAG 1-10
ASISTENTE SOCIAL - RECOLETA

I: Queríamos un poco conversar con ustedes sobre la experiencia de trabajo de la comuna de recoleta con la población refugiada, como históricamente, después como ver cómo ha ido funcionando los últimos años esta población. ¿Qué experiencia tiene usted de trabajo con ellos?

P: Claro es que mira: Cuando nosotros llegamos no había nada en cuanto a información registros sobre comunidad emigrante mucho menos sobre refugiados. O sea por experiencia propia yo venía de vínculos con fasic y con la vicaría viendo estos temas pero más a allá de esto no. yo sabía que vivían algunos chiquillo chiquillas refugiados con esa característica. Y tuve la posibilidad de sentarme con ellos de conversar de trabajar de darle un poco de confianza y Encontré dos tipos de refugiados: refugiados políticos que fueron Víctimas de represión pero con una posición definida podríamos decir cuya condición aquí eran poder insertarse a la vida civil, poner a ser parte de su vida lo cotidiano, seguir haciendo lo que ellos eran dirigentes sociales . Entonces es así cómo me vínculo con un grupo que siga siendo seguimos funcionando sobre todo tratando de poner en tema el tema de la condición de ellos y sus compatriotas a la luz pública. O sea lo están tratando de hacer no con tantos problemas y ahí nosotros estamos dándoles apoyo, pero no develando que ellos son refugiados, sino que son vecinos.

I: ¿porque se encontraron con una situación de personas ya organizadas en torno?

P: no no no no organizamos pero o sea muy bajo perfil, para ir colocando el tema porque pasa con los refugiados de América, pasa con los de Colombia principalmente, Perú Incluso un tiempo. Que la gente viene con mucho recelo mucho temor, que ambas partes las víctimas y los victimarios se encuentran en el país. Entonces resulta un poco complejo si es que no les dan garantías para que ellos puedan que se abran. Me paso harto con los colombianos, los colombianos son muy desconfiados unos de otros, hasta ahora no conozco organizaciones de refugiados colombianos. Vienen colombianos que son profesionales en situación migratoria regular... en el caso de los refugiados todo el tiempo lo es visto ahí nomás. Entonces qué me refiero en su condición es a nosotros, entonces en ese sentido vienen derivados de fasic vienen con una coordinación previa con ellos y ya sabemos. Pero es bien difícil en el caso de la comunidad colombiana te junten. En el caso de la comunidad Colón peruana no ha sido así estemos acá Todos abiertos como refugiados no, si no es que trabajan en una organización a través de una labor social porno con la comunidad y con todo eso. Entonces eso le favorece porque también están van trabajando su historia con la gente, Cómo rompiendo esos temores estos son tal y cual pero en el caso de los colombianos es más complicado. En el caso de los palestinos están tan lejos de su país y por otro lado ha sido otra historia no es la misma que la de los latinoamericanos eee... y palestinos refugiados aquí ya tienen larga Data entonces son poquísimos los que están en esta situación de Refugio ahora ellos son una comunidad fuerte, el país en el que se siente vulnerado, en este caso también las características son distintas. Después hay sólo un refugiado africano pero no lo ubicó en recoleta pero sé que vive. Así un poco es como la... de hecho la mayoría de los refugiados que están aquí están con un proceso con vista algunos están con definitiva, hay otros que no están todavía con la visa temporaria de refugio esperando que le dé la respuesta final e... solamente me tocó un caso de salud de un Dominicano que el corazón estuvo internado en el hospital muy mal y que estaba en esa situación de regresarte único campo de refugiados dominicano que me tocó. Después el resto Tú sabes que

acá el acceso a la salud es como mucho más viable ellos han tenido mucho más información que el migrante común y corriente. Porque de hecho siempre hay un sistema de protección que alerta de que ellos visitan el refugio. Tengo algunos refugiados o algunos que han estado tramitando su visa de Refugio que han pasado por los programas calle por ejemplo no tienen dónde acudir los presupuestos son tan bajos tan pobres, que hay que alojarlos en algún lugar. La vicaría, fasic el mismo departamento de refugiados no tienen los recursos como para todos y DAS los entregan como a mitad de año en junio Julio. Este año peor porque se han retrasado ¿Entonces qué hace la municipalidad? que también el área social ya pasó por una capacitación de refugio, la municipalidad por esta condición ellos no hacen ningún tipo de: no no no te vamos a ayudar. Por el contrario el total acceso para que una persona que está en situación de refugiados tenga prioridad en atención antes de un migrante común y corriente. Ahora tampoco son muchos los refugiados no es alta la demanda.

I: ¿el colectivo no por ejemplo que está pasando con ellos?

P: la mayoría no viene como condición de refugiado viene como migrante económico. Entonces yo creo que hay un grueso qué busca oportunidad nada más, no está ni aquí ni allá, no se define ideológica políticamente. Sabemos que hay un grupo no lo hemos visualizado. Pero la mayoría de los venezolanos son migrantes económicos y nosotros Incluso en la conversación siempre le preguntamos ¿y usted no va solicitar refugio? No .yo vengo y pienso que en algún momento el problema se va solo resolver el problema allá y me regreso.

I: no están usando en la vía del Refugio como...como forma

P: por lo menos lo que nosotros atendemos aquí no todos están solicitando visa. No están solicitando refugio.

I: ¿y con esta persona que ustedes han atendido refugiada Cuáles son las necesidades más urgentes? ¿Con las que llegan? ¿Cuáles son las principales demandas que hacen ellos a los municipios? ¿A las instituciones el general?

P: mira necesidades ellos yo creo que ellos como nunca se trazaron salir y hay una salida urgente rápida impensada. Yo creo que ellos… llegan ¿y qué hacemos no? O sea ni si quieras son demandantes ellos lo único que espero es protección. El municipio si bien es cierto para poder brindar este servicio hemos llegado a un acuerdo con refugio y con fasic.

I: ¿Claro porque ellos llegan derivados por fasic?

P: Claro.

I: ¿no llegan espontáneamente?

P: Muy pocos muy pocos los que conocen y saben del título de migrantes y refugiados.

I: Claro por eso

P: de repente a entonces a me pueden atender. Pero en el caso de la mayoría vienen derivados e...

I: Vienen derivados de Fasic

P: y Fasic los deriva cuando ya no puede atender una necesidad o cuando quiere como ya están ubicados en esta **zona** conozcan los servicios para que ellos puedan ser atendidos, entonces nosotros explicamos Como ya tiene la visa que está el registro social de hogares, están la becas para los niños no y tratamos que ellos se incorporen no como refugiados en una organización si no como en las organizaciones vecinales para que tengan más resguardo incluso, Entonces siempre nos orientamos para Cuanto más se abran mejor, O sea que no digan que son refugiados sino que Cuanto más se conozcan con personas mejor, que es una forma de resguardar que se protejan como persona también.

I: O sea evitar que se aíslen en la figura del refugiado

P: Si. No es que no queramos que se evidencian si no ya es decisión de cada persona, yo creo que cualquiera Qué es refugiado no y le cuesta un poco No tengo confianza con esta persona no sé si vendrá de parte de allá o de parte de acá. En el caso de los colombianos me han complicado porque ellos están mirando para atrás quién es. Tuvimos a una señora que ella decía que a ella la seguían. Entonces nosotros no pudimos tener Claridad de que si realmente la seguían O si ya estaba haciendo un cuadro psicótico. Pero Había que probarlo después No ya la perdimos. Entonces son como esos casos así e... Por nuestra falta también de equipo no, porque no Tenemos mucha gente. ¿A quién se la derivó? se la derivó a salud mental al cosam ¿la van a tomar por salud mental? o Vamos viendo otras cosas. Son casos aislados no es el común no son todos Yo creo que la sensación de cada refugiado cuando recién sale o cuando recién llega es Resguardarse sobre todo.

I: ¿Y en términos de necesidades como Prácticas de resolver: vivienda, educación, salud funcionan igual que un migrante económico?

P: Ellos de repente tiene por el sistema de protección la visa, es lo primero que tienen que eso, que les permite acceder a todos los servicios.

I: ¿eso se da una vez que se reconoce la condición o no?

P: Qué ellos cuando lo solicitan como solicitante le dan una visa.

I: Esa es la visa que más rápido obtienen a partir de la solicitud.

P: no se espera Por eso hay temporarias de 8 meses 4 veces o 5 veces hasta que el estado defina ya Tu estatus de refugiado te doy una visa definitiva.

I: Por eso **hay** visas temporarias de 8 meses que se van repitiendo y con esas son las que van..

P: con esas pueden acceder a la ficha A Servicios etcétera.

I: **Okey** ¿Acceden más o menos rápidos a ESOS servicios?

P: Claro una vez que ellos tienen la visa pueden acceder a la célula y pueden acceder a todo.

I: ¿Entonces ellos resuelven problemas de vivienda educación como inmigrante con visa?

P: Claro porque en ese caso Sí porque ellos no reciben algo más especial. Eventualmente cuando **fáci**l tiene das Y se requiere ayudar a una familia ya con un arriendo. Porque como vienen sin nada. Vienen vendiendo algo juntando el dinero ya saben a lo que vienen pero ellos no ¿A dónde los alojamos? Ahí está el conflicto porque a veces no existen los recursos, No hay una casa especial de acogida para los refugiados, No hay departamentos especiales asignados para los refugiados.

I: ¿Tampoco hay una red de derivación para los casos más específicos de ellos como el caso de esta colombiana? ¿Cómo se activa?

P: Claro nosotros de todas maneras no los dejamos, Él asunto es que nos comunicamos vamos viendo: en qué están los procesos cómo se están manejando. El tema como Te digo a veces no es tan fácil Y no corresponde a nosotros solamente resolverlos. Pero si nosotros tenemos que estar atentos a esos casos.

I: ¿y los van siguiendo más caso a caso?

P: Sí hasta que no se te pierda porque de repente no te dejan dirección no te deja nada Entonces ahí es cuando nosotros ya... Bueno yo lo dejo a consideración de **fasic** para que ellos puedan ayudar, que es una red más….

I: ¿Y eso pasa que se pierden como que son una población más difícil de retener?

P: O sea más difícil de ubicar sí, porque si ellos deciden no debelar su condición de refugiado no la debelan nomas. Lo bueno que pueden pasar por cualquier servicio y nosotros nos damos cuenta cuando de repente de sociales: ¿su visa tiene solo 8 meses? Entonces nosotros nos damos cuenta que si no lo tramitó por la vía regular está como refugiado. Pero no es algo que ellos de repente…

I: ¿La tendencias es esa que ellos no debelen en su condición? ¿O va cambiando?

P: es que como te digo yo sé que han aumentado los refugiados, yo sé cuántos son y son pocos los que tienen vínculos con la muni o yo sé que son refugiados pero hasta ahí nomás. Según el informe de fasic hay vecinos que son refugiados en recoleta pero yo creo que como te digo por esa posibilidad que ellos tienen de decidir si nos confían que son o no son refugiados. Porque de repente nos confían que son refugiados por una necesidad pero si no tienen la necesidad y todo les ayudó a resolver el estado entonces ellos continúan su vida por que interesa es seguir haciendo su vida, y creo que parte por ellos también y como te digo en la comuna no es como vaya primero por migrantes no. Acá la gente que ya tiene la cédula se va para cualquier servicio, salvo que tenga una duda ya puede venir acá tengo este problema o esta duda. Mientras que no se sienta rechazado por que también puede ser el efecto contrario, lo rechazaron en algún servicio ya no quiere acercarse a ninguno que eso de repente pasaba al principio con los refugiados, de repente tu ibas a un lugar y bueno por simplemente ser extranjero no le daban ningún tipo de atención entonces la gente ya se replegaban y prefería quedarse callado. Pero es más complicado ubicarlos porque ya es decisión de ellos

I: Entonces es más difícil pesquisar las necesidades especiales que puedan tener de salud o de salud mental.

P: Yo creo que sí. Parte de una experiencia cercana es que es distinta la atención de un profesional en salud mental que de uno que conoce el tema a cualquier profesional de salud mental. O sea el hecho que tú te sientas perseguido no es producto de su fantasía es producto de una realidad que sufre y que vivió y que si no está bien entendida. Un profesional común y corriente puede decir este está psicótico tiene delirio de persecución, pero sí formó parte de una realidad de tortura de persecución como e puedes decir que los ha inventado. Yo hay siento que debería haber una comprensión bien del tema y de la condición para saber cómo abordar estos cortes estas roturas que tiene un refugiado y que requiere de salud mental.

I: ¿y en estos momentos está siendo cubiertas por los servicios de salud?

P: Ahí si me agarras no sabría decirte si están cubiertas por lo menos yo estado haciéndole el seguimiento a fasic y yo sé que fasic ha tenido atenciones de salud concretas en el programa, no sé si todavía se mantienen y yo siempre sugerido que se mantengan ahí en FASIC. Yo sé que algunos lo han derivado a los cosam pero yo sé que a cosam te derivan por una parte más terapéutica de vínculo de esas cosas

I: ¿Y por ejemplo porque para ustedes es importante que se mantengan en FASIC?

P: No es que se mantengan sino que ojalá la propuesta de atención en salud mental Sea por un profesional que está capacitado en ese tema no, Por eso es que te decía que de repente preferible que mantenga la atención énfasis porque puede ser Que ellos tengan mucho más conocimiento sobre el tema que un psicólogo o un psiquiatra que esté en otra área.

I: claro ¿De repente dentro del sistema salud pública se puede diluir su condición o la lectura de sus síntomas dentro de un cuadro más específico?

P: Claro yo he visto y he conocido casos de que gente producto de las torturas o violaciones terminan en un cuadro de salud mental y cuando pasa psiquiatría son atendidos como cualquier caso de salud mental. entonces ahí me queda la duda si es que se profundiza en el caso se puede dar otro tipo de alternativa, porque yo no soy especialista en esto pero me imagino que de repente hay casos como que son más complejos y devienen en estos casos yo me he quedado pensando nada más.

I: ¿Y a nivel de Salud Municipal Cuánta preparación tienen Los funcionarios los equipos para poder Isaac invisibilidad esta particularidad en pacientes?

P: No Sabría darte una respuesta por que no estoy en este mundo y sería injusta si me pusiera a opinar. Yo creo que ahí el equipo de salud mental debería proponerse tocar el tema y ver si ellos mismos están en condiciones de asumir la atención de un refugiado o no. Si saben si están atendiendo a refugiado o no sería interesante saber.

I: Claro porque nosotros vemos en general que esa condición se diluye en la atención en salud.

P: Conocí un caso hace muchos años en atención central el joven ingresó para que el cosam lo atendiera y había situaciones de repente este delirio de persecución decidieron darlo de alta porque por esa situación había fallado dos veces. Entonces ahí mi intervención o sea mi alegato fue considerar que viene de una situación específica y en vez de alejarlo o dejarlo había más bien que mantener la continuidad y tratara un poco de general que la psicóloga que tenía cambio estuvo un

tiempo sin sicólogo había una situación en que perjudica el proceso de recuperación que se logró recuperar pero demoró... Demoro harto y eso sobre todo a los hijos de detenidos políticos o desaparecidos y esto es lo que a mí me sorprendió porque Chile con tanta experiencia en el tema y no ha logrado…

I: Porque es un sector pequeño de la sociedad que lo considera una experiencia que ha elaborado el tema pero claro a nivel de servicio no es algo que necesariamente esté instalado.

P: Claro y a mí me llamó harto la atención porque una fueron los padres y otra los hijos que originalmente no vivieron la situación de violación o tortura pero viven la situación de los padres, o vieron cosas que de repente uno no sabe que las vieron y no saben cómo expresarla quedaron con las imágenes ahí o con la negación que no pasó nada en absoluto.

I: ¿Esas experiencias en general ya que son poquitos los casos que entran a salud mental como las elaboran la población refugiada? ¿Es una experiencia personal?

P: Si lamentablemente no hay… en los pocas experiencias exitosas revertir estas condiciones es cuando se mantiene el grupo o sea cuando vienen de una experiencia que son como los rezagos de las organizaciones que han mantenido un vínculo se han apoyado entre sí pero los que han querido entre comillas romper por el temor y todo se han sentido más solos y más vulnerables a la situación. Entonces en esos casos yo he visto más vulnerabilidad y más riesgo que en los otros. Un colectivo que yo conozco que son como súper disciplinados y todo entre ellos se apoyan y yo veo que ellos van a hacer… con sus recursos están manteniendo mejor que otros que estén solos o que les cuesta volver a recuperarse y todo eso.

I: ¿Y hay un trabajo por estas colectividades por ejercer el refugio porque hoy en día se está viendo o sea usted misma dice se dice que están aumentando los casos pero no se está traduciendo en las necesidades que por ejemplo llegan a la municipalidad?

P: El derecho a refugio es un derecho que nosotros planteamos porque es un derecho humano pero yo creo que nadie quiere llegar a la condición de refugiado. Ahí esta la diferencia es un sistema de protección sí. Pero es distinto al derecho a migrar yo decido cuando me voy no otros van a decidir cuando yo me tengo que ir no. Yo creo que ahí está la diferencia entonces la diferencia en un sistema de protección que es legítimo y que hay que preservar frente a una condición que también es legítima y que uno lo puede determinar.

I: ¿Porque estábamos viendo también los distintos cambios de políticas que están viniendo ahora se está de alguna manera desestimulando el tema del refugio? ¿O sea la solicitud el ejercicio del refugio porque de alguna manera se están haciendo entrevistas de **admisibilidad** la gente en frontera está diciendo no puede pedir refugio porque su caso no corresponde no habilita? ¿La misma regulación migratoria también?

P: Es que es distinto la condición de refugiado de la que alguien decide irse del país porque acá no encuentra posibilidades. Yo conozco refugiados gente que ha sido perseguida por el estado o para militares que es distinta que a alguien que como acá se está cayendo la moneda yo me voy no me puedo considerar un refugiado ahí. Porque eso generalmente es una opción. Ahora lo que sí sé es que aquí en esta última etapa por gente de Colombia que viene que dijeron me negaron el refugio

pese a ser que tiene el documento de ser desplazado el tema está que es parece es política de este gobierno no aceptar las solicitudes de refugio salvo que haya que firme condición no las encuentran.

I: ¿entonces si ha visto un aumento de las negaciones por ejemplo?

P: Por lo menos si me da la impresión que si por que ya han venido varios y yo les he orientado que hagan otro tipo de visa porque no hay las condiciones pero como te digo también la percepción de la que voy a buscar refugio me estoy muriendo porque en mi país no hay condiciones para poder trabajar no nada. O sea esa no es una condición para solicitar refugio y eso se está tergiversando.

I: ¿pero llego a eso que se estaba tergiversando que la gente estaba utilizando el refugio aun sin una persecución?

P: Parece que sí, no puedo evidenciar por qué en algunos casos venía la gente vengo a solicitar refugio pero me lo han negado entonces, ¿pero usted la están persiguiendo? No no me están persiguiendo. Pero se puede dar el caso salvo que sea un líder político reconocido entonces ahí se va cayendo a idea que solicitar refugio es más fácil que solicitar la visa.

I: ¿Esa idea estuvo en algún momento?

P: Puede ser en caso de Venezuela en algún momento.

I: ¿Y ahí se orienta a?

P: La regularización, bueno ahora los venezolanos perdieron la oportunidad los que ingresaron después del 8 pero o sea, de todas maneras van a ver gente irregular los próximos años también porque no se puede poner parámetros a la migración de la noche a la mañana así hasta ca tu ingresas y después para tu casa, difícil

I: Y la gente sigue ingresando si tiene necesidad

P: Con o sin información y acá mas bien lo que proliferan son traficantes de gente de personas. Por qué te dicen: no yo te voy a cobrar tanto y tú tienes una visa, no te preocupes. Después los dejan tirados en frontera los dejan ahí y la gente dice ¿bueno que paso con mi visa? A quien le va a reclamar.

I: ¿Esa es una población que está apareciendo?

P: Lo que pasa que eso empezó con los dominicanos hace unos años y los dominicanos no dejaron de ingresar hasta hoy siguen ingresando.

I: ¿Cuándo le pusieron esta visa especial? ¿Consular?

P: La visa de turista que tenían que sacar desde el país de origen y no todos califican para turista. Pero sin embargo pagaban 1.000 2.000 dólares podían haberse venido con esa bolsa de viaje haca no sabían, pero pagaban 1.000 o 2.000 dólares para cruzar acá supuestamente les iban a dar la visa pero los tiraban ahí en paso no autorizado o sea yo creo que las políticas represivas restrictivas que se han dado no solamente en chile la experiencia de otros países no funcionan.

I: ¿están poniendo más vulnerables a cierta población?

P: Si es que los miramos desde los derechos humanos se está fomentando la vulneración de derechos, eso

I: ¿y qué recursos usted cree que necesitan los municipios que tiene población refugiada viviendo en su comuna para poder atender a esta población?

P: Yo creo que necesitamos economía, presupuestó para poder atender, no tanto pero para poder lograr darle alguna ayuda porque como te digo si viene alguien que arrancó de la noche a la mañana no le voy a decir vaya búsqueme una vivienda y nosotros se la pagamos como lo hacemos con los migrantes comunes, está en **shock** no sabe qué hacer, por lo que a él tenemos que acoger y llevarlo a un lugar y si es posible no dejarlo solo. Pero no hay esas condiciones en Chile es terrible.

I: ¿y usted ha atendido principalmente a familias o personas solas?

P: De todo, de ambos caso de familia y personas solas

I: ¿Y qué diferencias se producen entre familias y personas solas?

P: Yo creo que hay menos ansiedad, o sea no es que no haya ansiedad, hay menos ansiedad cuando está la familia obviamente las necesidades son mayores pero están más tranquilas porque están juntos. Pero si hay uno acá y los otros se quedaron allá, el que está acá no sabe cómo lo están pasando allá más que por la cuestión económica, ¿o sea estas bien?

I: Por la seguridad

P: Nosotros desde ahí es bien difícil porque la gente está como ida muchas veces, y los que nos queda es mandarlo a un refugio no ¿cómo se llama? Un albergue pero tampoco están las condiciones.

I: ¿Un albergue como del comercio?
P: Común una casa del estado o de acogida pero no lo es lo mismo la gente está en otra en una casa de acogida un refugiado no está perdido. Mira es un problema que tiene que verlo el **acnu**r con el estado y dar los recursos suficientes para que estos e pueda dar si es que se le asigna a una municipalidad o una ONG eso es los de menos la cosa es que resuelva un tema que puede tener sus implicancias para la salud mental en mediano o corto plazo.

I: ¿Y los tiempos de espera se han ido alargando? ¿También ha visto eso?

P: Si en el caso de los refugiados si las visas no están entregando de inmediato están dos tres cuatro años están con la visa temporaria.

I: ¿O sea hay una tendencia a alargar el proceso y a negar?

P: O sea yo creo que gente que tiene preparación organizativa de repente comunicacional que puede ir superando la condición de peligro y se puede asentar sobre todo cuando tiene comunicación con la familia.

I: ¿y quienes no tienen esas condiciones?

P: No asegura nada bueno, a mi particularmente es una situación que me afecta por lo que trato por favor **fasic** resuelve, lo bueno es que la municipalidad en cierta forma ha entendido y le da mayor prioridad que a un migrante económico.

I: ¿Ese ha sido el compromiso más tangible?

P: si ha habido dos casos, dos familias colombianos creen, que se les ayudó con el pago del arriendo pero así la encargada funciono súper bien. Pero como te digo en cuanto a salud cuando son familias tú los ves pero cuando están solos ahí tú los vez creo que todo el día están pensando y eso es malo.

I: Claro y como no tienen trabajo todo el círculo de una espera eterna de no poder

P: Y la idea es reencontrarse con la familia hay personas que no esperaban una situación de arrancar del lugar y venirse tan lejos una cosa es que arranques y te vayas de aquí al norte y otra cosa del norte al extremo sur.

I: Claro procesos más largos y de más incertidumbre.
I: ¿Si ha vivenciado u observado situaciones en que a los refugiados se **haya** sido más difícil formalizar su solicitud de refugio?

P: si hay varios casos. Las dos cosas

I: ¿que no llegan a la instancia?

P: las dos cosas, primero que a pesar que están con el documento de reasentamiento no les entregan el refugio acá.

I: ¿O sea ya fueron reconocidos como refugiados en otro país?

P: Claro en Colombia particularmente y otros que les han dado la primera visa pero no les están dado la condición el refugio. Por lo que han habido casos que yo les recomendado que postulen a la otra **visa** porque hasta ellos mismos saben que no les van a dar.

I: ¿y esto fue un cambio de este gobierno de marzo a la fecha o el año pasado se daba igual?

P: Hay casos de marzo a la fecha que la tendencia es más. Del gobierno pasado han estado entregando las temporarias pero no han estado definiendo rápido el refugio eso es lo que no he notado. Pero como te digo los que he conocido acá son pocos pero igual son significativos.

I: ¿Y lo otro muy técnico es lo que menciono del das que es este auspicio que recibe **fasic** que como que lo solicita pero no llega? ¿Cómo que lo firma y cuando llega eso?

P: No sé, nosotros tenemos un DAS para migrantes y das tiene uno para refugio, pero no ahora está demorando más está con un tema de procedimiento, cada vez son más, no sé cómo sea en refugio no me imagino que sean tan estricta porque ya el hecho de ser refugiado es una condición complicada.

I: ¿pero eso es un fondo como grande o por cada persona?

P: no es un fondo global y de acuerdo a lo que la identidad postula se proyecta más o menos a cuantas personas va a cubrir.

I: ¿Y quién da este fondo?

P: La subsecretaría del ministerio del interior, el departamento de acción social. Les decimos el das pero realmente ese fondo se llama programa de...apoyo para migrante en situación de vulnerabilidad. La de **fasic** es la de refugiados (ORASMI).

I: ¿Las municipalidades postulan al otro y f**asic a**l de refugiado?

P: Claro nosotros no hemos querido postular al de refugio porque consideramos que fasic está haciendo bien la pega y la siga haciendo con lo que pueda hacer. Pero cada vez se han ido bajando los fondos.

I: ¿Y eso a nivel metropolitano o nacional?

P: Nacional fasic ve todo el país

DATOS:

CLINICAS JURÍDICAS UDP
PP 11-37

Grabación 181002_1335

Transcriptora María Paz Errázuriz
I1: investigador 1
I2: investigador 2
I3: investigador 3
E: entrevistada
El uso de comillas se ocupará para cuando las personas presentes en la grabación hacen cita de otra persona hablando.
La “mm” se ocupará cuando alguien esta hablando y el que dice esto empatiza con o que esta diciendo
La “mhm” es un asentimiento de respuesta
Eeh: tratando de seguir la idea
Aah: tratando de seguir la idea
… se ocupará cuando la persona hace una pequeña pausa alargando la última silaba o letra de la palabra
Em para seguir una idea, como buscando con que seguir
Eem para seguir una idea pero es más pausado
Eemm para seguir una idea pero es más pausado y más alargado en cada letra

I1: Cómo están viendo la situación de refugio en Chile en este momento, eeh, en términos desde la mirada de las instituciones. Cómo ha sido co como la describirías la situación de lo de del tema refugio en Chile en este momento

E: mmh yo creo que hoy es compleja [I1: mm] si tuviera que ponerle ahí un calificativo [I1: mhm] es compleja al menos desde nuestra perspectiva que es la perspectiva legal [I1: mm] eeh vemos una serie de trabas en el procedimiento de asilo, eeh que se dan desde el inicio ¿no? [I1: mm] entonces tenemos hemos podido visualizar problemas de ingreso al país [I1: mm] de solicitante, después problemas de ingreso del procedimiento de solicitante, después problemas durante el procedimiento para, por ejemplo, renovar documentos, situaciones de archivo de caso y solicitudes de desarchivo sin repsuesta [I1: mm] eem el plazo que dura el procedimiento que son todo caso no es algo de este momento sino [I1: mm] sino que es algo que ha sido así desde ya hace un tiempo digamo [I1: mhm] no no tiene, la demora no tiene que ver con el gobierno de turno [I1: mm] como sí las otras cosas que mencioné [mm[que la relación ha sido distinta [I1: mm] . Tenemos un problema, siguiendo desde la perspectiva legal eeh los defensoras y defensoras hee de las personas refugiadas [I1: mhm] y solicitantes en nuestro actuar como abogado y abogada también tenemos traba [I1: mm] eeh y luego en la lo reconocimiento eeh aah bajo mi entendimiento hoy hay una

definición legal de refugiados que no se está aplicando [I1: mm] y si sigo te podría decir la fase recursiva [I1: mm] eeh presentar los recursos es complicado ya legalmente cómo por el tema de plazo [I2: mhm] , pero además, incluso si uno los presenta las posibilidades de éxito son muy bajas [I1: mm] [I2: mhm] se demora mucho tiempo en resolverse, entonces, tenemos un un sistema que la teoría es lindo funciona bien [I1: mm] , como si uno lee la ley salvo algunos matices más o menos estao bien [I1: claro porque la ley está como bien re] sí tienes ahí [I: reelaborada digamos] tiene ahí unos matices [I1: mhmh] que que yo creo que habría que revisar [mm[pero en principio no es una mala ley [I1: mm] es una buena ley [I1: mm] pero luego la práctica, por eso matices que te digo que hay que revisar [I1: mm] se dan unas situaciones así muy complejas y y nada (persona caminando 9 pasos) entonces espacialmente hoy es un momento complejo porque mucho de los factores que yo mencioné eeh tienen que ver con la situación, con la autoridad hoy [I1: mm] otros no, otros son generales [I1: mhm] : el plazo, cómo te decía, general [I1: mhm] , las posibilidades de éxito del recurso son cuestiones general [I1: mhm] no variado [I1: el estado siempre así] [I2: mhm] pero el ingreso al procedimiento, por ejemplo, es algo que hemos visto que ha cambiado negativamente [I1: mhm]
I 3: El la primera, digamos el primer contacto, la la la la posibilidad de solicitar ¿a eso te refieres? [I1: mhm]

E: sí

I 2: y qué, que ha pasado en esta formalización de asilo que que que…

E: las personas no son formalizadas ese es el problema, no existe un práctica, que es una práctica de admisibilidad proceso que la ley no existe en la admisibildiad [I1: mm], la ley, al menos como nosotros la entendemos, como la literabilidad de los artículos es que tu manifiestas tus necesidades de protección y de ingresar al procedimiento y en seguida a ti eeh deberían remitir tus antecedentes a la secretaria técnica. Esa remisión de antecedentes debería ser como es en la práctica: que a ti te entreguen el formulario, tu lo rellenas, adjuntas los documentos que tengai y lo mandan [I1: mm] , pero resulta que hay personas que les hacen una entrevista como esta [I1: mhm] la persona cuenta su historia y el funcionario obviamente por directrices de su jefatura [I1: mm] le dice: “no te voy a dar el formulario, no voy a recepcionar tus antecedentes [I1: mm[y remitirlo a la comi… a la secretaria técnica como dice la ley ¿porqué? Porque parece que tu caso no es de refugio [I1: mm] porque no entiendo porque no viniste antes, porque no entiendo porque viniste ahora, porque no entiendo porque te inscribiste en el proceso de regularización [I1: mm[[I2: mm[porque tienes una visa de turista vigente y esto no es para turistas” [I1: mm[porque, ósea cosas que incluso entre ellas se contradicen [I1: claro] y eso es eso es grave [I1: mhm] . Eeh con el gobierno anterior al menos acá (se deja algo encima de la mesa) en la universidad teníamos una relación que no estaba exenta de conflictos, osea, [I1: mm] pensábamos distinto, y pensamos [I1: mhm] hasta ahora distinto en muchas cosas, pero por lo menos teníamos una estrategia de dialogo para avanzar [I1: mm] , con este gobierno esa puerta se cerró [I1: mm] derechamente, eeh me atrevo a decir que no fue, digamos, ellos la cerraron [I1: mm] . Cerraron la puerta y entonces hoy cuando tenemos este tipo de casos, tenemos que pensar en otras vías jurídicas distintas al diálogo como para poder intentar que estas personas ingresen al proceso [I1: mm] . Y y y para MI, particularmente eso es grave porque una cosa son las personas que logran llegar a una asesoría jurídica [I1: mm] con otras

instituciones o con nosotros, da lo mismo [I1: claro], pero logran llegar, y ¿todo el resto de la gente? Que el funcionario le dice: no, mejor pide una visa. Osea hoy día atendí a un señor que le paso esto que les comenté [I1: mhm] y le dijeron toma pide una visa y le pasaron un documento con los requisitos de la visa sujeta a contrato y el señor me dice: "yo no sé leer, no sé leer, no entiendo, primero no entendí lo que me dijeron. Pero sí entendí que no iba a poder entrar al refugio, segundo me pasaron este papel (papel moviéndose) que no entiendo que es [I1: mhm] ¿me puede decir lo qué es?" Entonces eso es grave (papel moviéndose) si ese señor no hubiese llegado a la atención jurídica ¿qué?, ¿se hubiese quedado irregular? [I 1 o 3: claro] Entonces eso es complejo.

I3: y tú crees que eso se debió se debe a directrices que vienen como desde el alto que cambiar radicalmente desde un un gobierno a otro o también con un tema de desinformación con respecto a quién tiene derecho aaaa aaa refugio y quién no.

E: yo creo que son las dos cosas [I3: mm] osea si o si estoy yo creo que segura en un 99,9% de que estas son directrices que vienen de alto mando [I3: mhm], ahora no se si viene de la jefa de la sección de refugio, del jefe de del dem o del ministro [I3: mm] , o de Chadwick [I3: mm], nosé de quién viene, osea o del subsecretario, nosé de quién viene [I3: mm]. Pero de que es una jefatura es claro [I3: mhm] , no creo que los funcionarios, osea, hay funcionario que han actuado de una forma y que ahora están actuando de otra y es el mismo funcionarios [I3: es el mismo funcionario] [I2: mhm] eeh lo cual molesta pero es real [I3: m] entonces yo creo que viene de arriba, pero sí yo TENGO la sensación de que no haaay una entendimiento como a cabalidad de lo que implica ser refugiado [I3: mm[. Yo creo que conocen la ley, conocen la normativa pero no estoy tan segura que sean funcionarios que comprendan como como desde el derecho internacional de los derechos humanos [I3: mm] la protección [I3: mm]. Es exigible que un funcionario del estado vele más ¿por la persona que por el estado? No lo sé [I3: mm], no lo sé. Eeh yo tengo, esta es mi opinión personal [I3: mm] no institucional sino mi opinión personal es que lo funcionarios estatales tienden a defender mucho como a la institución administrativa [I3: mm] y lo entiendo, pero cuando estamos hablando de la protección de refugiados, para mi hay dos opciones: una, o ese funcionario, con directrices superiores obviamente, se saca un poco ese rol y entiende que esta en un punto intermedio entre [I3: mm[el estado y la persona ó creamos una institución distinta [I3: mm[como una defensoría penal pública [I3: mhm] como existe en Argentina [I3: claro[para refugiados [I3: mm[. Pero acá eh es difícil porque tú no te puedes enfrentar igual a un caso migratorio que un caso de asilo, osea acá hay gente [I3: mhm] que no ha venido a contar que le preguntan: "ya a ver, y usted por qué no vino antes" osea como acusandolos de un delito en el fondo [I3: mm[como: "ya y a ver y usted dice que lo persiguieron pero yo como que no la noto muy afectada, ¿por qué usted no está llorando?" y es como… [I3: mm] ¿te das cuenta? Entonces eso es como, son temas, está bien uno puede tener funcionarios con distintos caracteres, somos personas, yo entiendo eso, podemos tener malos días [I3: mm] pero ¿con los refugiados? [I3: mm] eso es un poco intolerable.

I3: eso nosotros hemos detectado, digamos, en esta pequeña como recorrido que hemos hecho (papel moviéndose) hablando con distintas personas y en distintas instituciones que existe como una confusión un poco al respecto a la diferencia que tiene un refugiado con un migrate "espontáneo", digamos. Y de hecho, incluso con con personal de salud existe este tema, de que bueno, en el fondo son todos víctimas de algo ¿no? De alguna manera los migrante económicos

también huyeron, también había violencia en el barrio, también había persu... todos los colombianos estaban en situaciones de eeh guerrilla por decirte o o tensión entre la guerrilla y el ejército, qué se yo, entonces porque a unos sí y otros no. Ese cuestionamiento existe de parte de los funcionarios de… de, bueno, nosotros lo que hemos visto en funcionarios de salud, pero también en el fondo, un poco también lo re lo lo encuentran en como los mismo refugiados han ido encontrando estas alas instituciones. Entonces ¿cómo se mmh visibiliza esa diferencia?

8´50´´

E: Sí, es complicado. Yo siento, que eem… yo misma [I3: mhm] me tengo que sentar y pe pensar [I3: mm] con profundidad en eso, porque desde la teoría como desde la teoría desde el derecho internacional de los refugiados, hay cierta doctrina, ciertos expertos y expertas que hablan de eeh… mirar la migración en general como una cuestión de derechos humanos y cuando tú haces eso te das cuenta que hay una distinción que está mal hecha [I1 o 3: mm]. Para lo los estados la distinción es: migración voluntaria o migración económica [I1: mm] versus migración forzada que es sinónimo, para ellos, de refugiados [I1: mm]. Entonces sitúan refugiados a la derecha, imagínate tú, eeh… todo lo voluntario a la izquierda y sólo los refugiados son forzados [I1: mm]. Resulta que esta esta teoría ¿no? Lo que plantea esta doctrina es decir: no, hay una división que está más allá de los refugiados. Entonces entre los refugiados y pasar a los voluntario hay una [I3: mm] gamma de personas, que incluso pueden haber venido por motivos que en principio entendemos cómo económico voluntario pero son económico forzado [I 3: mm]. Y para los migrantes forzados, que es cómo el nombre que se les dá [I3: mm], para los migrantes forzados eeh si bien no aplica el estatuto de refugiado aplica el derecho internacional de los derechos humanos dónde tenemos principios, como por ejemplo, la no discriminación, donde tenemos principios como la no devolución, ósea esas personas igual están en un riesgo de volver a sus países y hay que tener un grado de consideración con eso [I1: mm]. Eeh son estándares generales del derecho que, insisto, aplican más allá de los refugiados [I3: mm]. Yo creo que, entender eso, como para los estado o o en un momento en el momento que Chile está viviendo es pedir dar cinco pasos [I3: mm] cuando todavía estamos todavía muy atrás. Entonces les decía al principio que yo tengo que sentarme a pensar cómo civilizarlo [I3: mm] porque hasta ahora nunca lo he pensado [I3: mm]. Yo lo veo en clases con los alumnos y alumnas cuando estamos viendo la materia ¿no? [I1: mm], partimos en general los módulos hablando de migración voluntaria [I3: mm] y forzada y esta perspectiva que yo te estoy diciendo [I3: mm], donde ellos rompen como su esquema [I3: mm] y muchos de ellos no comprenden cómo esto que es tan evidente [I 1 o 3: mm] no se trata así legislativamente [I3: mm]. Entonces ahí tenemos un punto, em yo no estoy, claro, no creo que TODOS los colombianos eeh sean refugiados [I3: mhm] hay gente que efectivamente tenía un temor fundado a persecución o hay gente que en verdad venían escapando de la violencia [I 1 y 3: mhm] generalizado del conflicto interno y yo creo que hay zonas y zonas y no me atrevería a decir que es igual en todas partes [I1: mm] de Colombia, entonces quizá en la medida que uno pudiese dar información a las personas que trabajan en salud, información de país de origen cómo: mira existen en Colombia las zonas rojas [I3: mm] he es distinto Bogotá que Cali, es distinto Cartagena de India que, que se yo, Buenaventura [I1: mm] , y tú les vas explicando que no sólo están las farc ni las guerrillas [I1: mm] está el LN [I3: mm] , está labacrim… y como que vas armando, te empieza a hacer más sentido y te puede hacer más consciente para no tener ese prejuicio de decir como: son vienen todos a lo mismo, son narcos [I1: mm], todos mienten… ¿cachai?

[I1 y 3: mm] entonces yo creo que por ahí podría ir, en parte, y también entendiendo eeh la migración como una cuestión de derecho humanos en general, comprendiendo que eeh yo tengo un bcg, te voy a dar un ejemplo para que se entienda, [I3: mhm] yo hago un curso de formación general donde tengo cuatro estudiantes que son de derecho pero todos los demás, son treinta y nueve en total, son de otras carreras [I3: mm]. El semestre pasado tuve la mitad del curso eran de obstetricia, por ejemplo [I3: mhm] y eran chicas y chicos que tenían experiencia directa [I3: con intención claro[[I2: m] hacia esta práctica y claro para ellos yo les decía: "lo importante de comprender la distinción entre lo voluntario y forzado, es que, claro ustedes no son abogados, no van a ver cuál es el estatus jurídico aplicable [I3: mhm] pero van a poder aproximarse distinto [I3: mm[. Hee y este semestre tengo menos obstetras pero también tengo de todas las carreras casi [I3: m] [I2: m] que hay en la universidad eeh y es bien interesante ver cómo vamos derivando como los prejuicios [I3: mhm] , vamos entendiendo que, chuta, en la migración no todos están en la posición de decir: "hay voy a salir a hacer un [I1 o 3:mhm] post doctorado no sé qué ¡no!. Eeh… se trata de gente que huye [I1 o 3: mm], a lo mejor unos huyen de la violencia, de la persecución perfecto refugiado. Otros huyen del hambre [I3: mm] de que se yo conflictos medioambientales, eeh de la pobreza [I3: mhm] y después podemos seguir ¿no?, de la falta de trabajo nos vamos moviendo hacia lo voluntario pero seguimos aquí. Y de pronto, como, empezar a comprenderlo de esa forma en alguna… con alguna… con algún mecanismo sería algo que pudiese servir.

I3: porque otro de los temas que ha salido también es cuánto sea eeh… necesario visibilizar o cuánto también hay una demanda de invisibilizar la situación de refugio, porque por ejemplo hablando con una persona de eeh… salud en Recoleta aparecía el tema de que son las mismas personas refugiadas que no quieren visibilizar su estatu de refugiado, porque claro, esta todo el tema de estigmatización… en el fondo tratan de vivir como migrantes normales ¿no? Entonces por otra parte existe la necesidad de empezar a mirar que cuando una persona le dice: "yo estoy en situación de refugio" a un… a alguien que está atendiendo en algún consultorio, entienda esa especificidad que quisiéramos las instituciones más dedicadas a la investigación o a la acción en este ámbito que que que existiera ¿no?. Pero también ha aparecido en investigación el tema que se busca un poco la invisibilidad de la condición de refugio, claro porque hay temas asociados al estigma, estigmas asociados al riesgo no cierto… en el poder, en el reconocer este posicionamiento de las etiquetas de qué tipo de migrantes hay. Entonces no sé cómo tú ves este tema, en el fondo, la atención en visibilizar o invisibilidad el tema del refugio.

E: yo creo que de todas maneras hay que visibilizar [I3: mhm] (se deja algo encima de la mesa como sonido de vaso), y yo creo que la forma de hacerlo es no confundiendo como los distintas (I1, I2 ó I3 traga), las distintas formas de civilizar [I3: mm] yo creo, o no creo, estoy convencida digamos… legalmente que ninguna persona tiene la obligación de decir que es refugiada e o qué es solicitante de refugio. PERO, em, decirlo no implica que la persona que está al frente te tiene que preguntar cuál es tu historia [I1: mm[, entonces yo no veo, desde mi perspectiva, no veo cuál es el problema en que la matrona [I1: mhm] eeh le entre alguna duda de situación migratoria, como por ejemplo, no sabe si con la visa de ocho meses puede atenderte [I1: mm[o no, estoy inventando [I1: si, perfecto[cualquier cosa. Eeh… investigue y le diga: "¿usted porqué tiene una visa de ocho meses?" y la persona, si bien no está obligada, eeh ella sepa que ocho meses es muy probable que sea una visa de refugio [I3: claro]. Entonces diga: "aaah esta persona puede ser refugiada" y decida no

preguntarle y quedarse con esa información y averiguar por dentro [I3: mhm] o decida preguntar: "¿Y usted es refugiada?" "si" punto y no le tiene que preguntar ¿y qué le pasó? ¡Nada! [I3: mhm]. Eeh eso es como una arista, la otra arista yo siento que desde el estado existe esta idea de… estoy pensando en el ministerio del interior en verdad [I3: mm[, existe esta idea de invisibilizar porque temen que las personas empiecen como a pedir refugio en [I3: mm[masa y crean que refugio es sinónimo de eemm… de de querer co como tener una visa más rápido [I3: mm[porque claro la visa de refugio no se paga, la tramitación es un poquito más rápida si logras ingresar al procedimiento claro [I3: claro[etcétera. Pero yo creo que eeh la forma en que ellos lo están atacando, que es con la invilización eeh trae más problemas que beneficio [I1 o 3: mhm], es como el típico dicho o que se usa acá en derecho es: yo que prefiero ¿no? Eeh... tener un culpable suelto o un inocente en la cárcel, yo prefiero tener un culpable suelto punto. Entonces acá es lo mismo: yo prefiero que haya gente que se aproveche y que el estado, que no creo que pase [I 1y 3: mhm] pero si llegara a pasar, el estado tenga que adecuar sus procedimientos para buscar mecanismo legales apropiados y proteccionales, del resto de las personas que no se aprovechan, ¿para dejar estas personas de lado? A mí se me ocurren ideas [I3: mhm] después se las voy a decir, en vez de tener una cuestión oculta, oscurantista un poco [I3: mhm] eeh y que entonces dejemos fuera gente que si lo necesita [I1: mm]. Y en ese sentido yo siempre he pensado que debiese haber folletos informativos de qué es el refugio, cómo se hace, cómo se pide [I3: mm], debería… todas las personas que solicitan refugio deberían poder entrar al procedimiento [I3: mm] y luego dentro del proceso tu podrías distinguir entre los casos que tienen sentido y los que no. Supongamos que alguien te anota en el formulario (hoja moviéndose encima de la mesa) "vengo por refugio económico" [I1: mhm] tú dices okey voy a hacer un procedimiento acedelaro porque no existe el refugio económico (hoja de mueve encima de mesa). Entrevistas a la persona, alguien capacitado y lo económico puede ser refugio [I3: mhm] al menos desde las directrices que ha dado la cnur [I1: mhm] eeh… entonces esa persona capacitada puede hacer la entrevista y darse cuenta que sí, esta persona realmente no tenía idea [I 1 o 3: mhm] y se metió acá porque le dijeron [I1: mm] o que no po, o que su problema económico era que no accedía al trabajo porque era perseguido políticamente porque [I1 o 3: mm] era opositor al gobierno… y ahí la cuestión empieza a cambiar [I3: claro] eeh… y tá vas armando, yo creo que hay mecanismos, y esos mecanismos significarían más gasto para el estado contratar más gente ¡sí! Pero te permitirían tener adentro a los que si lo necesitan [I3: mm] con mecanismos pa filtrar en vez de otra cosa… entonces de nuevo como volviendo a tu pregunta, para mí hay que civilizar si o si y arreglarnolá para con eso no pasar a llevar a las personas [I1 o 3: mm] y ahí es primordial escuchar a las personas, porque yo también te hablo desde mi rol de abogada y [I 1 o 3: mm] de académica de la universidad, ósea, yo no soy refugiada gracias a Dios entonces no sé cómo lo ven ellos. [I 1o 3: mm] Cuáles serían sus temores… para poder hacer un protocolo por ejemplo [I1 o 3: mhm] en los hospitales o en los consultorios. Eeh tratando de abarcar esos temores pero visibilizando

I3: claro, es que quizá esos temores tienen que ver mucho con lo que tú dices respecto a cómo la gente ¿accede? A hacer la la solicitudes de refugio. Porque las entrevistas que hemos hecho la mayoría es como un… un rumor que les llega ¿no? "Alguien me contó que existía el refugio. Yo ni siquiera sabía que existía el refugio, me lo escuche en la iglesia. Una amiga que vive en Colombia también fue refugiada en Canadá… entonces ella me contó que existía esta cosa." Entonces todos llegan casi por mecanismos que son un poco oscuros, tú usas la palabra oscurantista ¿no?. Y también están estos tabúes respecto a estos privilegios que tendría el refugio, entonces también no quieren

hacerlo ver a los otros, porque es como un bien preciado eeh que podría producir discriminaciones dentro de la propia comunidad ¿no?. Que tú no te hiciste eem no pasaste por irregularidad, no tuviste que hacer contrato de trabajo… entonces todas estas ideas respecto a estos beneficios que tendría el refugio.

E: claro, pero por lo mismo, osea eeh, de nuevo no hay que confundir [I3: mhm]. Ósea una cosa es que instituciones del estado y los empleadores que yo también veo ahí otro foco, comprendan [I3: mm] qué es el refugio. Y cuando ven un aviso de ocho meses, cuando vean que la persona les dice "estoy en un procedimiento especial que me van a decir, después, si me dan la definitiva o no [I1: mhm], chuta el empleador le haga algo click y diga "aah refugio" y tenga una reunión y entienda el principio de confidencialidad. Ó ó que haya otros mecanismos [I1 o 3: mm], eso no significa que la persona va a tener que contarle a sus vecinos ¿cacha? [I 3: mm] esa es como la diferencia. Eso es por un lado. Y lo otro es que eeh la información. ósea nosotros en la red de fronteras que participamos con el facing [I3: mhm] y otras instituciones hemos planteado mucha veces eeh tener folletos en la frontera [I3: mm] dónde diga así como "usted haga, usted no haga, y estos son sus derechos, este… perdón, este es el refugio, esto es lo que tú puedes hacer" pero ahí siempre la gente, yo diría, como del gobierno eeh se cierra un poco a esta posibilidad por este mismo miedo [I3: mm]. Y al final, insisto, pagan justos por pecadores y eso no corresponde. En un estado de derecho [I3: mm] si Chile, además (se deja algo encima de la mesa) ratificó, a firmado la convección, [I3: mm] la convección sobre el estatuto de los refugiados, la convención americana. Eeh y si se jacta [I1: mhm] además hacia el exterior [I1: mhm] de ser un país que promueve el asilo (se deja algo sobre la mesa) estamos mal [I1: mm] ¿no?
Yo creo que hay que sacarse el estigma de que lo (la entrevistada carraspea 2 veces) los refugiados vienen a aprovecharse [I3: mhm]. Además con todo el respeto del mundo que merecen los fondos das es que aprovechamiento. [I1 o 3: mhm] los refugiado les dan nada (en tono sarcástico). En en Alemania los refugiados son pocos [I3: mhm] pero tienen acceso, osea, cursos de alemán, te lo digo así, [I3: mhm] por experiencia CERCANA. Cursos de alemán eeh apoyo para ir a la universidad [I3: mhm] ósea… acá los fondos son súper [I3: de hecho ha sido súper interesante] acotados…

I3: también, es ver que la gente en estas esperas de ocho meses tan largas, que además existe un rumor con respecto a cuánto va a durarse esta va a esperar esa visa definitiva. Empiezan a dudar de no renunciar [E: sipo] a la solicitudes.

E: ¡exacto! Esa es la gente que atendemos nosotros todo el día, todo el tiempo. yo atiendo los días martes o como hoy dia en la mañana en facic [I3: mhm] y resulta que la mayoría de los casos que me llegan para atender en el fácil son esos. Ósea no sé si la mayoría, pero un porcentaje súper considerables [I3: mhm] personas que me dicen "mira sabes que mi empleador me está pidiendo la definitiva" hoy día atendí DOS CASOS de personas que me decían "cómo llego a la definitiva para poder tener el subsidio de vivienda [I1: mm], y necesito pedir un prestamos porque quiero atender a mi hijo y el banco me dicen que todo bien, pero cuando tenga la definitiva." Entonces yo decía "ya ¿cuánto tiempo llevas en el proceso?" desde 2016 eran los casos de hoy. Yo dije "mira YO te recomiendo que te esperes" además escuche los casos y me parecía que eran casos como bastante claros de asilo [I3: mm], decía "yo te recomiendo que te esperes, ya llevas un montón de tiempo… [I1 o 3: mhm] para qué vas a renunciar ahora. Eeh tu hijo, por ejemplo, en todo caso es mayor de

edad… busca la forma en la qu ese venga solito y acá, claro, y ahí fuimos viendo otras dudas. Pero en el fondo eeh, hay mucho de eso. La falta de información, el miedo, el que me dijeron qué. Ósea hay muchas veces la conversación con los patrocinados parte: "mire, a mí me dijeron esto pero usted sabe que dicen muchas cosas. Entonces yo vengo acá para que usted me clarifique" chuta y uno es como [I2: mm] no tengo ese don… eeh [I3 y 2: mm] voy a hacer lo posible desde mi conocimiento. [i1: claro] Pero al final es el estado el que debería brindar la información. Y muchas veces pasa que uno le dice una cosa y los funcionarios del estado dicen unas cuestiones así… "Por qué no te vas, (se escucha ambulancia a lo lejos) lo que tú tienes que hacer es agarrar tus cosas y devolverte a Cuba" Así le dijeron a una patrocinada hace un mes.
Entonces uno es como, insisto [I1 o 3: mm], yo entiendo que el funcionario estatal no es abogado, ósea, de profesión puede serlo, pero me refiero no es abogado de la persona [I3: mm], no es de la parte [I3: claro] como nosotros. Entonces entiendo que no le vay a decir "mira tu tienes trecientas opciones, [I3: mhm] puedes presentar trecientos recursos en contra de nosotros [I1: te vamos a esperar claro], yo entiendo eso te juro que lo entiendo pero entonces el estado debería tener una oficina como anexa de atención a la persona. Eeh creo que Argentina es un súper buen modelo en eso [I1: mhm].

I3: claro porque todo esto se pierde en todo este caos, ya burocrático, enorme que hay entorno a la (una palabra que no se entiende que dice) migración en general ¿no?

E: y al final ellos mismos pierden.

I3: claro

E: ellos mismos pierden porque la sección de refugio, no sé si fueron…
I3 o 1: mhm... no yo…
I3 o 1: no…

E: al departamento de extranjería. Piso tres tú abres una puerta entras como a un pequeño holl digamos, una cosa como la está allá, no se [I1 mhm] si lo vieron [I1: sí] [I2: mhm], esa parte que está ahí y hay oficinitas como esta así más chiquititas. Y aquí se atiende a las personas y después hay una puerta que tú entras y están, ósea la gente común y corriente no entra porque es donde están los abogados la parte interna, pero esta es la parte que ve la gente un hoollcito con sillas y unas oficinitas. Y resulta que ellos hoy [I1: mm] si tú vas hoy te van a dar hora para enero. Osea es un montón de tiempo. Estamos pensando, ósea, personas necesitadas de protección. Últimamente nos están llegando casos de personas, que por ejemplo, se les vence el turismo… entraron hace un mes y medio se enteraron que existe este refugio [I 1 o 3: mm], llegaron acá a la clínica, les dieron hora para enero y su visa se vence ahora en noviembre, de turista. Entonces u dices tengo hora en enero [I3: mhm] en noviembre me quedo irregular ¿qué hago? Si… si… una opción es irse, que no es opción para ellos [I1 o 3: mhm]. Opción dos, eeh prorrogar el turismo, son cien dólares, no lo tienen muchos de ellos no lo tienen. Opción tres si pido una visa, claro, pero a la hora que va a pedir refugio con una visa presentada (no se entiende la palabra que dice el investigador 3) el refugio le va a decir "oye pero tu ya pediste una visa" [I3: mhm] credibilidad "no tu no tienes necesidades de protección" [I1: por supuesto] entonces ¿cuál es la solución que le dan a la gente? Nada po

I1: que se quede irregular
E: que se quede irregular. Entonces si tú me preguntai (I1 carraspea), esto suena súper feo, pero jurídicamente lo mejor para un solicitante que está convencido que es solicitante es no hacer nada, es quedarse irregular hasta la cita

I1: mm

E: y

I1: con todas las consecuencias que tiene a nivel de…

E: que si después le dicen que no va a tener que pagar una multa. Ósea hoy nosotros estamos presentando acciones judiciales para entrar al procedimiento. Acciones judiciales que se demoran dos meses en tramitación [I1: mm] en el poder judicial y es de las más rápidas que hay
I1: Claro [I2: mm]

E: entonces la gente dice "chuta y qué qué puedo hacer más rápido, yo no…" lamentablemente le dicen nada po. Estamos haciendo todo [I1: claro] yo ya presente las protecciones, estoy esperando el informe, ósea estoy esperando ¿cachai? [I1: mm] estamos esperando todas y todos. Entonces hay una burocracia general con las migraciones pero en refugio pierden ellos porque si ellos te dan hora para enero, en cambio si tuvieran un proceso donde todos entran [I1: mm] todos entran ya filo todos entran todos entran yo después voy a tener un proceso de determinación, quienes siguen y quienes no [I1: mhm]. Te ahorras la fila, te ahorras la hora pa enero, te ahorras la irregularidad migratoria, ósea se están comiendo su propia cola [I1: mm]. Y en eso la verdad yo soy súper crítica porque siento que ellos son súper poco autocríticos [I1 o 3: mm] en eso también. Entonces no tengo ningún temor [I 1 y 3: mm] en decirlo.

I1: claro y en el fondo co como funciona después, más allá del tema de la solicitud del asilo. Cómo funciona la vida de estas personas en el fondo, cómo son sus eeh se resuelven sus otras necesidades: vivienda, educación, salud eeh… qué hace el estado para atender eso y qué hacen ellos en el fondo [E: claro] un poco. (Se deja algo en la mesa)

E: eeh de nuevo ahí es complicado, porque mientras son solicitantes, ósea mientras dura el procedimiento eeh yo entiendo que existe este fondo das [I3: mhm], entiendo porque trabajo los martes en el fácil, entiendo que existe este fondo das. Entiendo que es una cuestión súper ultra mega hiper acotada [I 1 y 3: mhm], eeh entonces realmente cuando viene la gente a la atención jurídica y nos pregunta cómo "oye eeh cómo puedo acceder a apoyo" en mi mente ese apoyo realmente no existe. Yo les digo "hable con la trabajadora social del faci porque yo no sé darle una respuesta." Eeh pero en el fondo yo creo que de todas las personas que me hacen esas preguntas… un diez por ciento debe recibir el apoyo efectivo [I3: algo].
Hay muchos temas de unificación familiar, hay personas que necesitan apoyo para traer a sus familiares y eso no existe (no se entiende la palabra que dice I3) ni si quiera con el das [I1: mm]. Eeh entonces yo lo que veo, desde mi opinión personal y la atención, es que son personas que se las

juegan todo el tiempo buscando un trabajo aquí acá, algunos tienen dos tres trabajos, osea son personas sumamente esforzadas eeh que están en un proceso con mucha incertidumbre, no saben cuánto va a durar, cuando termine no saben cuál va a ser el resultado, no sabe cuándo va a poder traer a sus hijos. Algunos tienen problemas laborales por el tema de la la adoración de la visa. Eeh en acceso a la salud, en general no he visto mayores problemas [I1 y 3: mhm] porque existe al parecer un acceso más o menos expedito [I 1 y 3: mm] en Fonasa. Eem pero claro también algunos de ellos eeh que tienen enfermedades y que refieren un tratamiento más específico, una recuperación más específica Fonasa no satisface a sus necesidades [I1: claro], entonces muchos de ellos dicen "voy a ir a Colombia, voy a ir a mi país de origen a operarme y vuelvo" y ahí vienen las dudas de i puedo hacer, como puedo lo hago… eeh y van, por ejemplo, a una zona distinta de la cual escaparon [I1 y 3: mm], van sólo a operaciones y se devuelven [I1: mm]. Eem… es complicado, es complicado la yo siento que la incertidumbre al final les provoca como muchos problemas prácticas [I3: mm] y muchos problemas psicológicos también [I1 o 3: mm]. En fásica y en atención psicológica a nosotros hemos derivado, no te diría en comparación numérica digamos no son muuuchos casos [I3: mhm] pero igual es considerable al menos una vez cada dos meses derivamos un caso a [I1: mm] a atención psicológica porque vemos ahí ya un tema….

I1: y porque derivan, en el fondo, cuando ya ven algo ya más… [E: sí] porque en el fondo una consultoria jurídico, me imagino, que no hay como una… eem… ósea hay atención pero en el fondo lo que tu puedes ver es desde el sentido común [E: si] de cómo está la persona ¿no?

E: es desde el sentido común [I1: claro] eeh a mí me ha pasado un par de veces que noto que la persona tiene un grado de estrés post traumático [I1 y 3: mm], ósea lo noto sin ser NADA experta [I1: mhm] es como desde lo que cualquiera de nosotros (no se entienda la palabra que dice I1 asintiendo esto) podría saber. Y las personas lo manifiestas [I3: mm] sabe que estoy mal (se deja algo encima de la mesa) [I3 o 1: mm].
Hoy día mismo me pasó, por ejemplo, ¿no? [I1: mm] hoy una persona de la atención me dijo "estoy mal, estoy mal y necesito ayuda. [I1: mm] Eeh cómo puedo hacer para tener ayuda psicológica porque no puedo, no tengo mi familia, me dijo, no tengo mi mamá, noo tengo mis tías, tengo mi hija de un año, estoy embarazada con un embarazo de alto riesgo y no estoestoy pudiendo" yo le dije ya tranquila vamos a hacer [I1: mhm], hice la derivación al tiro a la ebelideface [I1: mhm] y todo hee para que lo derive a la psicóloga [I3: mhm].
Pero es complicado, yo creo que una vida una vida con tanta incertidumbre legal [I3: mhm] es algo que nosotros no podríamos ni si quiera imaginar [I1: mm]. Yo trato, de verdad, de ponerme en el lugar [I3: mm] pero siento que no soy humanamente capaz de entender en porfundidad [I2: mhm] cómo se siente tener tanta incertidumbre, porque pa nosotros hemos vivido toda nuestra vida ellendo con la cédula [I1: claro] a cualquier parte o cualquier cosa: oye aquí está mi cedula. Si a mi me toman detenida este es mi país yo ya se lo que tengo que hacer, no por ser abogada [I1: mhm], por ser ciudadana [I1: ciudadana]. Es complicado.

I3: si porque eso es lo que estamos viendo en el fondo que también en esta situación de sobrevivencia, en el fondo, el tema salud queda completamente relegado a una dimensión emergencial ¿no? [E: si] de decuando ya estamos al borde de… una descompensación importante, una crisis importante, [E:si] existe la búsqueda

E: si, yo creo que en todo caso eso en mi opinión muy, insisto, [I3: mhm] muy inexperta [I1: mhm] y muy desde afuera en eso eeh yo creo que es algo que afecta a los migrantes en general [I3: mm], las personas migrantes en general. Yo creo que con los refugiados pasa todavía (se escucha algo apoyándose sobre la mesa) en segundo plano porque hay tanta incertidumbre [I1: mm] legal de mi estatuto de quién soy yo aquí (se apoya algo sobre la mesa) [I1: mm] que efectivamente queda relegado. Insisto en todo caso que yo he visto que hay un acceso más o menos equitativo ÑI3: mhm] eeh al menos en acceso y y en temas del embarazo en general la mujere embarazadas refugiado solicitante no he visto [I1: mm] que tengan mayor problema. Pero claro ins…en este tema de las enfermedades operaciones [I3: mhm] ahí ya veo que es problemático y ahí también me declaro súper incompetente [I3: mm] en cuanto al conocimiento porque yo no sé cómo funciona Fonasa con el tema de las operaciones [I3: mm], yo entiendo que pa chilenos y extranjeros es lento.

I3: claro no y ya solo para las interconsultar empieza a ser complejo ósea si si e… cuando están en buenas condiciones de salud, ósea no necesitan ¿especialistas?

E: ya…

I3: eem quien lo hace es el consultorio y la atención básica primaria, la que tenemos todos digamos, en temas como Fonasa A. Apenas se requiere de algo un poquito más especializado una interconsulta a un ginecólogo empiezan los pro [E: los problemas] claro, porque ellos están en Fonasa A entonces no pagan y entonces tienen acceso a se disminuye notablemente la cantidad de personas que atiende pa disponibles pa ellos ¿no? Especialistas. Entonces por eso después los que son operaciones, todo lo que no este cubierto por ges, por auge… ahí empieza como ladevaria en el fondo [E: claro] la adupcion que tenemos nosotros, chilenos, de ir a buscar algo final de… pagado, ellos no entran en eso. Entonces ahí empiezan a aparecer estas otras que tu dices ¿no? De las posibilidades de buscar alternativas en, volviendo a otros país o….

E: que complicado te juro [I3: mhm]. Pa mi desde afuera yo tengo Fonasa [I3: mhm] pero pa mi desde afuera, por ejemplo, yo no estoy inscrita en un consultorio [I3: mhm] hay un consultorio a… diez cuadras de mi casa [I3: mhm] y no estoy inscrita porque no entiendo cómo hacerlo ¡te juro! Ósea pa mi es mucho más fácil y me me molesta esto [I1 o 3: mm] pa mi es mucho más fácil pedir una hora en Integramedica [I1: claro] ¿cachai? [I1: y pagar la diferencia] [I2: mhm] y pagar la diferencia que tampoco es tanto y uno dice como ya. Imagínate que pa estas personas que está la incertidumbre, la ansiedad, el estrés post traumático, lo que vivieron y más encima es complicado entrar. Y cuando logra entra vay con el médico general [I3: mm], con la matrona etcétera pero después [I1: bueno] es complicado.

I1: y porque, claro, en el fondo con todos estos encuentros en ningún momento tu situación de refugio esta eeh… nose si ni si quiera verbalizada, alguna me han dicho que, claro, lo dicen… pero eso no socita nada en el otro porque…

E: nopo

I1: porque no lo conocen

E: nopo

I1: ósea no entienden que es una persona embarazada, de alto riesgo, está en situación de refugio… estamos hablando de una persona extremamente vulnerable

E: exacto, no acá nada

I1: es un factor más me entendí?

E: es una extranjera más

I1: claro

E: y ese eso es problemático. Es súper problemático. Para mí hace mucha falta e de capacitación de [I1: mhm]. yo lo he pensado muchas veces, incluso [I1: mhm] lo he pensa… lo hablaba con una niña de una colega de la universidad de la Universidad Católica [I1: mhm] a raíz de un curso que ellos están haciendo de contratación de migrantes… hay una clase sobre migrantes y refugiados [I3: mhm] desde la perspectiva de los derechos humanos… y entonces yo hablaba con ella y le decía “mira esto sería importante como capacitar, por ejemplo, empleadores” [I1: mm]. Hablando con ustedes digo: claro sería importante capacitar a funcionarios públicos pero ¿cómo? [I1: mm] [I2: mm]. Porque nosotros desde las universidades, la universidad de ustedes, nuestra universidad, no podemos llegar y hablar con el ministerio de salud y decirle como “hola queremos capacitar a sus funcionarios “[I1: mhm]. Las respuesta “y bueno quienes son ustedes”…

I1: claro

E: podemos teer experiencia, investigación, años de atención pero…

I1: claro. [E: así nomás] No está esta como necesidad

E: nopo

I1: ósea esto que te decía yo de de identificar que atender a un refugiado es distinto que atender a un migrante cualquiera es son miradas que vienen desde incluso en este caso, por ejemplo, si estás haciendo algo alusión a funcionarios municipales, corporación municipal de salud… entonces estamos hablando de personas que podrían su ósea tienen que concursar sus capacitaciones por decirte ¿no? Pero claro se enfocan otras cosas que que visibilizan como necesidad, porque el tema refugio no está considerado una necesidad.

E: claro

E1: un poco también por lo numero porque existe un poco como un mito a cuanto el refugio sea una necesidad primordial hoy día también en chile. Eem por una parte este como alarmismo de que hayan crecido como los muchos y deje que la gente está usando pa tratar de evadir las otras formas que… se han vuelto más rígidas las otras formas de… de acceso a la visa. Y por otra parte, hay, tener conocimiento de que el refugio es una realidad temporánea y que sí, hay gente que está pidiendo refugio en chile. Ósea están esos dos extremos.

I3: eeh eso te iba a preguntar, eso. Eeh en terrenos de ustedes, como es una trayectoria de más años de trabajo ¿Qué han visto respecto al flujo? Ósea han visto aumento, han visto aumento co… de las esperas, ósea como han id observando digamos, como ha funcionado el flu… ósea el asilo en estos últimos años.

E: mira, desde que partimos la clínica que fue el año 2009… eeh las leyes del 2010 [I3: mm] ¿cierto? El reglamento del 2011 yo creo que… no, no yo creo, EL 2011 [I3: mm] nos pensamos meter más de lleno en temas de refugio eeh… pero si o si la cantidad ha ido aumentando [I3: mhm] ¿ya? Yo creo no no tengo las cifras [I1 o 3: mm] porque nuestra base de datos recién partió el 2017 [I3: ya] [I1: mm] ya tienes lo que sabíamos (se escucha ambulancia a lo lejos) [I1 o 3: mm] eeh… y y en eso yo creo que anualmente atendíamos 50 personas refugiadas entre fase y la clínica quizá 100 Ï1: mhm]. Nosotros al año, yo creo que hacemos, unas estoy pensando 50... 100… o 200… o 300… atenciones a personas que vienen por temas de asilo [I1 o 3: ya], que sean después solicitantes eso es otra cosa [I1: claro] pero por temas de asilo… yo creo que la menos son 300 personas al año [I 1 o 3: mhm]. Entonces ha ido aumentando un montón, han cambiado los flujos... Al principio la mayoría la… obviamente, la mayoría de lo solicitantes que atendíamos personas por asilo eran [I1 o 3: mhm] colombianos y colombianas, después muto mucho a persona dominicanas mucha… hubo un tiempo que teníamos muchos casos, 30 casos, de mujeres dominicanas víctimas de violencia [I1 y 3: mm] y eso son los casos que abrimos [I1: mm], porque habían muchos más que no abrimos [I1: mm] por distintos factores. Eeh y después tuvimos como un bun de personas cubanas que veían solicitando asilo, que para mí hasta el día de hoy esas personas son solicitantes, vienen escapando un régimen que no les permite tener un grado de libertad. Muchas de ellas, claro, te dicen yo no tenía problemas mi problema es que no ganaba lo que yo quería [I1: mm] ya esos no son solicitantes. Pero es gente que dice “yo no podía trabajar porque por mi opinión política contraía al gobierno a mí me pedían cartas de recomendación del CDR, que es una institución cubana [I1 o 3: mm] y el CDR no me la daba, porque yo soy contrario al gobierno: soy un gusano” así les llamas allá [I2: mm] entonces al final tú tienes a una persona que no puede desarrollarse, que no puede trabajar, que no puede comer [I1: mm] no porque no le guste cuánto gana ni porque se afloja… es porque no lo dejan trabajar por su opinión política. Entonces yo creo que eso nunca lo comprendieron en el estado [I1 o 3: mm], estoy hablando del gobierno anterior, [I1 y I3: mm] nunca lo comprendieron. Entonces bueno…aumento los cubanos y cubanas y hoy en día estamos con fuerte con Colombia y Venezuela de nuevo [I1: mm] eeh Colombia de nuevo y Venezuela fuerte [I1: mhm] sin prejuicio de que van apareciendo otras nacionalidades más lejanas ¿no? De Asia [I3: sí… (no se entiende lo que dice)], Afganistán. Eeh en ese sentido, digamos, no tanto, aumentando mutando y el cuanto como a los plazos… eeh… yo no estaba acá el 2011 entonces no te sé decir. Pero si tengo la sensación de que antes era más rápido porque eran muchos menos

I1: claro

E: entonces se atendían al año 200 casos de asilo, entonces son como cifran del dem [I1, 2 y 3: mm] y hoy se atienden al mes mucho más de 200 yo creo que on casi que, en todo chile al dia, deben ser 200 atenciones al dia. Entonces tenemos una cuestión gigantesca y claro lo que antes se demoraba ocho meses ahora se demora 2, 3, 4 años incluso, y después si te dicen que no y tu apelas… [I1: e l p r o c e s o...].
Tenemos un caso en la clínica que estaba desde el dos mil… te juro que ya ni sé. Sé que era el procedimiento nuevo [I1: mhm] significa que fue al menos el 2010 y que después el 2013 le dijeron que no y presentamos un recurso y lo fallaron hace dos mes y hace dos meses es.. yo creo que es uno de los pocos casos que han cambiado la decisión
I1: aaa lo aceptar…
E: lo aceptaron [I2: mm]
I3: pero me imagino que como de otro año
E: ósea tanto tiempo sipo. Su hermana reconocida como refugiada en parte por eso lo reconocieron después [I1 y2: mm]. Y porque está el gobierno anterior y la jefa, digamos, igual teníamos una relación de dialogo entonces le explicamos, le acompañamos documentos ta ta tá. Sirvió hasta ante pero después pedimos por acceso a la información [I1: mhm] cuantas… cuantos casos se han presentado recursos y cuantos han sido fallado a favor y decía el año 2013 se presentó 35 de curso y se ha fallado 1 ¿la nacionalidad? La nacionalidad era nuestro patrocinador [I3: aah] ese uno era el nuestro [i3: era (no se entiende)].
¿Te das cuenta? Que como de 35 de ese año que se presentaron.
I1: que más que todas estas posibilidades de presentar recursos tienen que ver con las posibilidades que tengan acceso a centros como ustedes, osea esto y pensado en casos en antofagasta…Iquique otros lugares que tengan menos, digamos que tengan menos posicionados [E: exacto] pa sus comunas [E: exacto]

E: hay. Hay en Iquique hay en arica esta fasicarica, fasiciquique, fasicantofagasta y… fasicantofagasta y fasicaiquique tienen una relación con universidades o con algún centro [I3: mm], entonces hay asistencia jurídica especializada [I3: hay] eeh… pero también súper acotada ósea [I1: claro] en Iquique, por ejemplo, son dos abogados no es una clínica. Nosotros en la clínica somos 4 personas en el equipo docente, 3 de nosotros son abogadas y abogados eeh… y somos y los estudiantes [I1: mm] que son ellos los que hacen trabajos [i1: claro] en este semestre son 9 si es que son 10, 12, 8 [I1: osea hay] son todo un grupo [I1: todo un equipo, claro de más de 10 eprsonas] acotaaado sí. No lo otros que están ene le quipo no están todo el día en la universidad pero somos un equipo y estamos ahí tenemos capacidad de llevar casos ÑI1: mm] alla son 2 abogados y eso es todo. Por ejemplo ellos por mandato no ven casos de per personas migrantes que no pudieron ingresar al proceso, nosotros sí [I3: claro] ellos no. Ellos toman solo, en principio, tomas solo personas eeh después de que formalizan entonces [I1 y 2: mm] ya te queda toda esa gente afuera. Igual desde la buena voluntad ellos hacen todo lo que pueden [I3: claro] ¿no? Pero por mandato de aquí para allá [I1: mm]. Y después cuanta es la capacidad de presentar recursos eeh hemos hecho nosotros con el acnur y con fasic temas de capacitación des… desde lo que nosotros podemos hablar, desde la experiencia [I1 o 3: mm] lo que el acnur técnicamente puede decir y facic. Ósea hemos intentado peor todavía es muy poco, de hecho una de mis alumnas de tesis acaba de terminar, ayer mando el

informe de tesis, le puse un 7 entonces está muy buena [I1: mhm] eeh sobre acceso a la justicia de personas migrantes en general afectadas por órdenes de explosión, no tiene que ver con esto [I3: mm], pero sí ella en una parte trata el tema de la asistencia jurídica especializada a[I1: mm] y… cifra, nosotros no somos especialista en técnica cuantitativa y cualitativa claramente, pero ya desde una cuestión general que una abogada puede ver se da cuenta que es un treinta por ciento la gente que puede acceder a la asistencia. Imagínate con los refugiados [I3: mhm] si estamos con el oscurantismo ¿no? [I1: claro] [I2: mm] es súper poco. Entonces es co complicado, yo igual siento que esto puede mejor, que puede cambiar eem hace que ahora hay que esperar. Pero de que tiene que haber mayor capacitación… todo parte por ahí también. Eeeh como profesora también siento que desde las universidades en distintas carreras y distintas universidades, así como acá hay un cefege un curso de formación general, y además tenemos un electivo en derecho [I3: claro], y además tenemos esta clínica… eeh a mí me encantaría que en otras universidades [I1: mm] se abriera el tema.
I3: acá se ha ido posicionando como un tema transversal a la formación de los estudiantes
E: si, [I3: mm] si partimos teniendo solo la clínica después tenemos de…. La que tuvimos también fue mi profesora de clínica hacía también un electivo que claro, tenía un poco… se llamaba me acuerdo eeh… porque yo lo tome… era algo de la población en siglo 21 entonces era… el nombre igual no te llamaba mucho [I3: te llamaba eso]. Después con la Delfina, cuando ella era la directora de la clínica, lo cambiamos ah… eh… derecho internacional de los refugiados y después ahora yo… lo hago con otro profesor que es relator de naciones unidad [I1 y 3: mhm] de derechos de los migrantes entonces se llama: migrante y refugiados. [I1: ya…] Entonces del tema migrante yo veo refugiados [I1: okey] entones ya vamos metiendo el tema con los alumnos. En el cfege partimos el semestre pasado, son un montón de alumnos es un sábado y van un montón. Entonces es súper rico ver que hay ingeniero en gestión de control que están ahí interesados en el tema… pero hace mucha falta [I1: mm]. Ósea uno podría pensar y yo no se qué pasa en la universidad de ustedes, nosé que pasa en la Finis Terra, no sé que pasa qué se yo… estoy pensando en universidades facultados de derecho [I1: mm] con otro foque [I3: claro] ni siquiera sé si lo toman. No sé si en la Chile hay algo así. Yo se que en la Alberto Hurtado están avanzado en esto [I1: si], en la Cato de a poco se van metiendo… pero en otras universidades… [i1: no… claro…]

I1: ósea que claro, depende del inte interés que tengan compromiso pa tener parte de la planta docente porque si no ese tema no existe [E: exacto].
Ósea en nuestra universidad, en la universidad del Desarrollo no existía hasta que eeh… este programa estudios sociales en salud empieza a mirar migración, y dentro de migraciones emergen el refugio como como un tema… pero es un ósea un micromolécula al lado de todos los otros intereses que están mucho más en la maquina: como la productividad, la innovación, la tecnología, todos los otros sectores en lo que, esto es, absolutamente mínimo digamos…

E: exacto

I2: en preguntas tengo algunas (risas) [I1: que…]. Sí insinuas varias cosas que quería profundizar un poquito, artiendo esta si hay algunas particularidades que hay observado en este flujo nuevo, relativamente nuevo, en población del sern… (no se entiende esa palabra 46´ 38´´)

E: si yo creo que hay varias cosas: Uno es que no todos ellos se visualizas, yo creo que de apoco están empezando a hacer, pero no todos ellos se visualizan como refugiados, como personas en necesidades de protección. Muchos de ellos y acá hay un colega venezolano acá en la universidad que está haciendo su doctorado y lo hemos habaldo ¿no? El venezolano es una persona que es luchadora entonces en su mente es como "yo vengo acá, yo vengo a trabajar, yo vengo aportar. Yo no soy una carga. Ósea no me digas eso" ósea el refugiado igual carga, entonces ahí como que se... Por eso muchos de ellos desconocen el tema del refugio. Ya en el último tiempo la cuestión explotaba a tal nivel que ya chao con… se sacan ese chaleco y como que ya, me da lo mismo necesito protección. Pero antes no. Eso es como una primera cuestión
Segundo hemos visualizado muchos problemas en términos de documentación. Estamos justamente ahora trabajando, nosotros tenemos un proyecto particular de personas veenozalanas a parte de la atención general [I1: mhm] que eeh… coordinamos y con la Fernanda que es la profesora asistente de la clínica [I2: mhm], ella ve esa parte, y…. hemos visto problemas con los niños y niñas [I2: mhm] que hasta los nueve años en Venezuela [i3: tunotiencarné…] tú no tienes el carné. Entonces vienen la frontera traen sólo su partía de nacimiento, entonces claro, el niño entra por refugio, el papa y la mama no se sienten en necesidad de protección aunque en mi juicio las tienen. Piden una visa pero el niño está en refugio ¿Cómo se trata eso?
Segundo problemas para entrar sin documentación adulto y niño hay adultos que tienen el pasaporte vencido y que no se los renovaron. A mí me han contado MUCHAS personas que allá hay que pagarle a tramitadores montón de dinero… que no tienen eeh… entonces al final tenemos al final gente acá sin pasaporte, sin documento, no sabemos cómo funciona el proceso de filiación… que es ese proceso el registro civil por el cual tu puedes adquirir como una identidad que el registro civil de alguna forma credita. Te digo así tal cual, no sé cómo lo acredita, no sé dónde se pide, no sé cómo se pide. En el gobierno anterior con la jefa de sección de refugio de anterior, se hacía a través de la sección, entonces la persona iba a la sección cuando no tenía pasaporte ni documento de identidad… le daban un documento lo rellenaba, lo mandaba al registro civil y ma o meno esa es la información que teníamos, tampoco sabíamos cuánto demora. Hoy no tenemos idea de nada, justamente al almuerzo estaba hablando con la Fernanda sobre este tema y le decía ya Fernanda que hacemos eeh… hazte un acceso a la información publica al registro civil preguntando y plántate un día en el registro civil con un alumno, le dije, te parece como y pide hablar con alguien que te explique. Porque necesitamos explicarle a la gente y si nosotros no lo entendemos ¿qué les vamos a explicar? Entonces hay muy poca información eeh… sobre… la documentación, que es un problema que afecta a MUCHAS personas, no solo a venezolanos, pero particularmente a venezolanos [I1: mm].
y… otra característica que te pudiese mencionar sobre esa nacionalidad eem… (Silencio 49´51´´ al 49´54´´)no yo creo que esas dos cuestiones: la documentación y el la… el no sentirse refugiado (no se entiende lo que dice en investigador 1 en el minuto 49´59´´) [I2: mm]

I2: interesante lo de la vergüenza a pedir ayuda de los venezolano, porque lo había escuchado una vez y dije como "''aah yia… una persona" como que no, no pueden sentirse como frágiles

E: sí

I2: y ustedes tiene como harto trabajo en red de frontera po [E: si] y… como has visto la capacidad ahí de los funcionar y también han pesquisado ustedes mismo desde este centro en que no están capacitados en otras áreas

E: si

I2: se mantienen, continúan, ¿Qué pasa en el interior?

E: mira… igual insisto en la información de fronteras que nosotros tenemos es desde lo que nos cuenta las personas [I1: mhm] y desde los que nos cuentas las personas de las redes… entonces no es una información se primera, ósea no es tan [I3: no es tan de primera fuete] de primera fuente y si es de primera fuente es desde el relato de una persona [I2: claro] le creemos y todo pero también la realidad de cada lugar…

I1: claro, es muy desde la percepción

E: claro. Eeh… yo he visto como… altos y bajos en el fondo. Siento que desde el 2013 a la fecha sí o si hemos avanzado positivamente. Ósea… el 2013 1014 teníamos relatos de que en la frontera había fidades de blancos y negros, eso yo nunca lo he vuelto a escuchar. Pero se mantiene la discrecionalidad de cuánto dinero te pido.. acá en la universidad tenemos un estudio sobre violencia sexual en contra a las mujeres migrantes en cuando a su trayecto migratorio a chile y ahí damos cuenta de… cómo… el hecho de que un día te piden quinientos dólares, y otro día te pidan mil, y otro dia te pidan cien… hacen que mujeres caigan ¿no? en manos de traficantes que a su vez las violentan sexualmente. Esta enfocado por ahí pero saca esto a la luz [I1: mm]. Eso es por una falta de regulación que existe y yo creo que eso se mantiene. En temas de refugio yo he visto que es menos el rechazo en fronteras. Yo creo que un pick como de bondas (leves risas) eeh… el año pasado 2017 dónde no había casi, entiendo yo, rechazos de solicitantes de asilo en frontera [I1 o 3: mhm] y ahora hemos ido como volviendo un poco a los rechazos pero siempre mejor que lo que pasba hasta 2014 ¿ya? En cifras, no me recuerso ahora. Yo hice un acceso a una información pidiendo los rechazos y los motivos del rechazo en fronteras, creo que la hice el año pasado, entonces ahí la tendría que mirar… no me acuerdo como ha sido la cifra. Pero las cifras tampoco te van a decir cuantas de esas personas manifestaron necesidad de protección [I1: mm].

I3: claro porque en rechazo en general

E: rechazo en general [I1:mhm]

I1: y estos montos que tu dices lo que se les pide como dinero… ¿bajo que, digamos, figura se pide ese dinero? ¿en forma de corrupción como de…?

E : no, en forma de acreditación de turismo

I1: aaah perfecto

E: si

I1: ya

E: pero lo….
I1: la famosa como maleta turística en el fondo, que tu tienes que demostrar que traes plata.

E: pero ahí está el punto. Porque legalmente tú no tienes que demostrar que traes plata [I1: mhm] (se deja algo sobre la mesa). Esa es la cuestión, la ley lo que dice es que el funcionario de frontera determinará cuándo solicitarla a la persona acreditar un sustento económico. Ósea ya te dice primero que el funcionario puede pedirlo o no pedirlo [I1: mhm], y cuando lo pide… no dice cuánto es ni cómo se calcula [I1: claro]. Yo entiendo que no puede decir el monto pero podría decir como calcularlo, o podría decir sobre la base del índice de… [I3: claro] otra cuestión variante. Nada. Entonces eso provoca que haya gente (I1 o 3 carraspea fuerte) que probablemente, gente europea o rubia que no les piden NADA [I1 y 3: nada] nada… ósea ni se les ocurre preguntarles cuánta plata trae y hay otra gente que le dice "cuánta plata tare… a no es suficiente" ósea he sabido, las hermanas de Piciga nos han contado muchas veces esta historia… personas que saben que el de barba pide cien y el pleao te pide deciento, y que las mujer es la más pesada de todas, pero la mujer que esta los martes es la que siempre mantiene un monto [I1: es la que mantiene un monto constante]. Ósea sé porque se sabe [I3: eso… eso…]

I3: eso existe como figura jurídica. Ósea yo he visto que… nosé cuando uno entra a España tu podí entrar… tu sabes que te pueden pedir pero existe un monto que te pueden pedir. Ósea te piden cien dólares por día que te vas a quedar. Entonces si tú tienes un pasaje de quince días podrías mostrarse un monto que quinientos dólares, ósea esta cómo CLARO qué es lo que te van a pedir

E: claro, y cómo [I3: mhm]. Porque, por ejemplo, acá yo no sé si unapersna puede mostarr eso con su tarjeta [I3: crédito]¿ de crédito? ¿tienen que traer la plata en efectivo? Ósea es tan grave que hay mucha gente que hay ahí mismo en la frontera, que se presta plata con traficantes de personas, y ahí al menos desde nuestra investigación, ahí es donde muchas acuden, recurren, o caen en manos de traficantes de personas, tanto consciente o coacionadamente [I1: mm],ósea… estoy pensando en mujeres que saben que lo que están haciendo es ilegal pero no pueden volver a dominicana porque el marido les va a sacar la cresta ó estoy pensando en persona que "no mira si el Alexi… el Alexi te pasa todo legal" y la mina vá con el Alexi y el Alexi la pasa po, pero resulta que después se dá cuenta cuando pasa y no vió niun control, que fue de noche, que habían cosas medias raras, que el tipo les pidió el pasaporte y no los devolvió… [I1 y 3: mm] entonces ahí empiezan a darse cuenta que son traficadas. Sobre todo en el paso de mujeres ¿no?

I1: tu decías hay un… hubo un momento de tráfico de mujeres dominicanas más pick, porque bueno esto te lo pregunto por otro lado porque yo viví en dominicana y allá hay en la organización internación de migraciones nos pidieron un estudio sobre tráfico de mujeres dominicanas en el mundo y miraba a chile como uno de los posibles destinos en ese momentos, estábamos en el 2016

E: claro (I1 carraspea fuerte). No sé si hubo un momento… no se te decir si hubo un aumento de trafico [I1: mhm] como delito [I1: mhm] pero sí durante 2017 nosotros recibimos en la clínica, la mayoría de las personas que vinieron a hacer consulta fueron dominicanas hombres y mujeres [I1: mm]. Eeh… el 2017 yo tengo la base de datos, me acuerdo, que fueron 584 atenciones en todo el año y en esas 584… 260 fueron de personas dominicanas [I3: dominicanas]. Eeh… eso también tiene que ver con que la gente se pasa se corre la voz, oye aquí tienen dominicanas y conocen el tema [I1: claro] [I2: mm]. Ahí muchas de estas personas manifestaban de que alguien lo pasó en las fronteras, ósea de que había trafico había, de que lo denunciaran no. Porque ellos lo que les interesaba era en verdad… eeh tener su documentación al día [I1: mm]. Porque chile pudieser un lugar por la exigencia de visa consular desde el 2012 [I1: si…] [I3: claro, si…].
Eso es directamente proporcional a la cantidad [I3: mhm] de personas que ingresan irregular y en ese sentido al trafico de personas.

I3: fue como el efecto [E: mhm] domino dede la re la impoción de visa consular [E: ¡sí!] que después se genera esto…

E: y esto puede seguir pasando ahora, de hecho yo no sé… hasta ahora no hemos visto como ha influido con las personas haitianas. Pero… lamentablemente podría ser algo que se repita o quizá el factor idiomático va a jugar a favor para que las personas haitianas no… sean traficadas [I1: mm]. Como no entender y después no no entiendo prefiero no ir. Y yo espero que sea ahí, ósea para por todos los riesgos [I2: mm] asociados sobre todo con las mujeres. [I1: mm]
(57´13´´ al 57´20´´ hay silencio y sonidos bucales poco modulados. No se entienden pero es para ver quien sigue con la siguiente pregunta)

I2: mencionaste que si bien desde el 2010 hubo una gran cambio. Siguen habiendo matices en la ley.

E: sí, osea el 2010 el gran cambio es que se hizo la ley [I1: mm] [I2: sí] [i3: claro] ¿cierto?. Eh... pero los matices que hay en la ley precisamente tiene que ver con… primero eeh… bueno esto es mi opinión personas igual, [I2: mhm] con el ingreso al procedimiento, osea la ley tiene un articulo 26 y siguiente el 27 que hablan esto de… eeh... la persona se presenta ante, dice, cualquier funcionario de la administración del estado puede ser la gobernación provincial o el departamento de extranjería y migración manifiesta su solicitud de asilo y luego viene el artículo 27… el funcionario que se entere eeh… de la solicitud de una persona remitirá los antecedentes en el mas breve plazo. Para mi ahí no hay ningún paso previo, pero legalmente claro no existe una admisibilidad, entonces no debería existir una entrevista donde yo te escucho donde yo diga mira si, mira no [I1: mhm] eso no debería pasar. Pero tampoco dice cómo tiene que ser po. Esta este la persona manifiesta y luego remite los antecedentes debería haber algo que te explicite: la persona entonces otorgará el formulario, o el formulario estará disponible en el sitio web… algo con el famoso formulario.
I1: claro el formulario no debería estar sujeto a que alguien te lo entregue
E: no debería estar sujeto a alguien que te lo entregue
I1: claro
E: NO debería estar sujeto a que alguien te lo entregue. De hecho la ley 9.880, la ley de base administrativo, en algún artículo que no me acuerdo ahora… habla de que para trámites que sean

generales la autoridad debe proveer formularios. Entonces yo siempre he pensado… no, no siempre esto es mentira (entre leve risa), hace un tiempo que me senté a pensar en esto que debería haber así como los formularios de visa… [I1: mm] tu deberías poder descargar el formulario así de mandarlo por correo [I1: mhm] y que el trabajo fuera de lo otro, fuera del tipo que se sienta a determinar y dice ya… hay países que existen procedimientos abreviados [I1: mm] no es lo mejor pero es MEJOR que dejar fuera a personas en necesidad [I1: claro]. Ese es un matiz el otro matiz tiene que ver con la autoridad que decide [I1: mm] eeh… la… una mí otra alumna de tesis que está haciendo sobre faltas de bio procesos en temas de refugio… hizo ahí entrevistas y varias personas le dijeron que piensan que no debería ser el ministro del interior ósea el subsecretario del iterior quien tome la decisión. Debería ser la comisión, porque resulta que la comisión discute el caso, se sienta a sesionar, piensa en el fondo se IMPREGNA del caso ¿y el que toma la decisión es otro? Es raro eso [I2: mm]. Sabemos que en la práctica o tenemos ma o meno conocimiento que si en la práctica la comisión dice A, el subsecretario va a decir A ¿pero si no?
¿Te das cuenta? Entonces esos son matices que yo… yo pienso que la ley 20.430 debería ser reformada.

I1: y en este momento la comisión está formada quienes y cuanta expertices tienen como para poder, realmente, fallar en torno refugio y no refugio.

E: ¿la ley? Establece que la conforman seis personas [I1: mhm] de las cuales sólo cinco votan [I1: mhm]. Ya seis son tres personas del ministerio del interior, dos de relaciones exteriores y una del acnur [I1: mhm] la del acnur es la que [I3: ve todo] opina pero no vota. Y de estas cinco, de las del ministerio del exterior uno si o sí es el jefe del departamento de extranjería que preside la comisión [I1 y 3: mhm] si o sí quién sea. Ahora Alvaro Belolio ¿no? [I3: mhm]. Después de estas dos personas, hasta donde yo entiendo, una es una persona es del gaviete del subsecretario y la otra es el oficial del legibilidad que hace el estudio en la secretaria técnica, en palabras sencillas de la sección de refugios el dem [I1: mhm] que analiza el caso, la que hace la entrevista, la que hace el informe, esa persona.

I3: osea es una persona clave digamos

E: ¡Es la persona clave! Digamos [I1: la que va a recopilar] y después [I1: los datos] yo que en algún momento iba también la jefa… Laurelia… en su tiempo. Entonces no sé si ahí va siempre la Aurelia y el oficial de legibilidad se estaba al lado a aír así sh sh sh y ella era la oficial… ahí no sé [I1: mhm]. Menos sé cómo funciona ahora. Y de las dos personas de relaciones exteriores entiendo que una eeh.. es de… del tema de derechos humanos, siempre ehe sabido que este señor… como se llama…. Hay un señor que siempre iba… [I1: mm] no me acuerdo… pero es un tipo que trabaja en el área de derecho humanos del ministerio de relaciones exteriores Pedro algo…. Y la otra persona de relaciones exteriores no sé quién es. Lo que si se es que para la última sesión que se hicieron por primera vez en la historia de la ley no sé si toda la comú… (no se entiende lo que dice 1 hrs 01´53´´) porque no es obligación llamarlo. Históricamente en todas la sesiones que se han hecho desde la sesión 1 a la fecha +ósea 2010 o 2011 hasta ahora siempre el acnur había ido… ahora no lo citaron en la última que se hizo. Asique no tengo idea

I1: ese es como un gesto político… como del…?

E: no… en mi opinión personal es un gesto político. Yo siempre que están súper fraccionadas, quebradas las relaciones entre el gobierno y la sociedad civil, no te sabría decir como es con el acnur [I1: mm] el acnur es una organización internacional… no debería haber un quiebre ahí [I1 y 2: mm] pero te podría habar desde nuestra experiencia con la sociedad civil, ósea yo he sabido oficial y no oficialmente que hay un quiebre. Oficialmente lo he leído en el diario de la columnas de Alvaro Belolio he visto y he sido objeto de sus faltas de respeto en el congreso nacional hacia las clínicas jurídicas en general eem… lo cual me parece bastante absurdo porque somos instituciones… no lo entiendo bien, en mi mente como que no me hace tanto sentido [I1: mm]. Eeh… siento que hay un fraccionamiento como desde lo político cómo que ellos creen que todos los que no estamos de acuerdo con su política migratoria es por que tenemos algún tema ideológico y, yo personalmente, no me lito en ningún partido político y me da lo mismo el partido político… me importan las personas. si viene… te voy a dar un ejemplo extremo he sabido que políticamente es un poco incorrecta las cosas que dice Jose Antonia Kast a mi me da lo mismo si es Jose Antonia Kast, Piñera o Florcita Mutua que propone un cambio en la ley 20.430 y yo lo voy a apoyar osea… [I1: mhm] no es político ¿no?. Eeh… y lu luego ¿no? Eso es como oficial y no oficialmente eeh hay personas que trabajaban en extranjería y que ya no trabajan ahí y que nos han dicho ósea [I1 y 2: mhm] hay directrices desde el mismo subsecretario que no se dialoga con las clínicas jurídicas, ni nosotros ni la Alberto Hurtado [I1 o 3: mhm] ni el servicio jesuita por ejemplo. [I2: mm]

I1: tendrían que haber abierto ahí.

E: Ósea mis alumnos los han amenazado con llamar a los guardias cuando han ido con directrices especificas mias [I1: mhm] y han ido acompañados por una ayudante [I1: mm] ósea no han ido solos, han ido con una ayudante que son mis ojos y mis oídos que me llaman por teléfono. Y en la fila como “usted quién es” “no vengo acompañando” “¿de dónde viene” “de la clínica jurídica” “váyase o llamo a los guardias” [I1: mm] váyase o… ósea… no han denegado… hemos ido a presentar documentos por oficina de parte y nos dicen “aaah…. De la Diego Portales” pam al suelo… ¡al suelo! Bueno yo les digo a mis alumnos, bueno deben tener una caja abajo y no te lo tomes personal, pero ellos dicen que la actitud les parece asi… y otras veces que no los han dejado… les han dicho “no, no recibimos las cartas” [I1: mm] Entonces ahí es un fraccionamiento complicado.

I1: interno el tema de la credibilidad, bueno yo trabaje en Europa con estos temas en Italia, y ahí había un tema con respecto cómo acceder a las pruebas de la persecución. [E: mm] entonces incluso en las comisiones se ponían médicos, psiquiatras, había todo un tema psicológico en torno a la búsqueda digamos de esta verdad respecto a esta persecución ¿eso aparece en la… en los casos que ustedes ven ó cómo se comprueba la veracidad de estos relatos?

E: La credibilidad siempre es un tema [I1: mm] eeh a mi juicio mal entendido por parte de la autoridad porque al final se provoca un cuestionamiento de. Eeh yo por temas de confidencialidad no te lo puedo mostrar… pero tengo una entrevista eeh… que yo pueda acompañar a la patrocinada, una entrevista de legibilidad. Las preguntas eran “¿Por qué usted mintió cuando dijo tal cosa? ¿Por qué usted oculto la infor…?” y resulta que nuestra respuesta la patrocinada no quiso hablar en ese

momento nos dijo “no voy a contestar” [I1: mhm] entonces después dimos las respuestas por escrito y nuestra primer cotación fue que no nos parecía correcto asumir que había mentido…. La patrocinada se había confundido por ejemplo con una fecha, dijo: yo tenía veintidós años y tenía veintiuno estamos hablando de una cuestión lo… y ahora tiene cuarenta, estamos hablando e una cuestión que a todos nos puede pasar. [I1: claro] Eeh… entonces la credibilidad siempre es un tema yo creo que acá está mal entendía se entiende como un migrante que viene aprovecharse y por eso le aaaah le busco la pillería [I1 y 3: mm] todo el tiempo sin entender que hay otros factores que influyen en la persona. Eeh en la legislación la presentación de documento, en la legislación chilena, la presentación de documento es una cuestión entre un derecho y una obligación. Ese es otro matiz que habría que revisar. Mi alumna de tesis sugiere que debería estar puesta como un derecho y no como una obligación [I3: mm] o al menos no como esa forma rara que está puesta [I3: mm] hoy.
Eem… pero dice que bueno en la medida que lo tenga. El estándar internacional es de probabilidad si yo le pongo un número es un estándar de cincuenta por ciento, ósea la autoridad tiene que conversarse que es probable que esa persona le podría pasar o le haya pasado lo que dice. Pero no tiene que ser certeza [I1: mm] ni… duda razonal… nada de eso ¿Por qué? Porque un refugiado eeh… (ríe) yo siempre le hago un ejercicio a los alumnos, le supongo un cronómetro de un minuto y les hago pensar en las tres cosas que se llevarían si es que tuviesen que escapar en la noche de su casa porque afuera están cayendo bobas y nadie dice… una foto de la bomba cayendo (risas), el diario de ese día [i1: ni el carné del lectoral…] nadie dice eso. Imagínate de personas que son perseguidas que les mandan cartas a su casa y quién se las va a llevar ¡NADA! Ni las denuncias… ¡nada! La gente agarra la maleta y se vá. Entonces es bien interesante eeh… cómo funciona la credibilidad en ese sentido. Y… yo creo que a veces mal entendió por parte de la autoridad [I1: mm].
En los casos que son reconocidos… que hemos podido ver nosotros, tampoco es que la persona en todo caso haya tenido que aportar tanta prueba [I1: ya] pero mientras más prueba tiene uno piensa que mejor le va a ir a la persona y lamentablemente cuando una persona, por ejemplo, de Colombia viene tiene toda la historia de asilo fuerte y no tiene ningún documento… uno vé que no está... ósea yo pienso que las posibilidades de que la reconozcan en cincuenta y cincuenta [I1: mm], aunque sean casos de libro [I1: mm]. Eso

I3: ósea que las pruebas están jugando un rol importante en la construcción de esta credibilidad

E: yo en mi opinión personal juega un rol importante y obviamente espero que… que sea solo como en la dureza [I1: mm] de las preguntas peor que no tenga un factor tan relevante o que no sea un factor tal relevante en cuanto a reconocimiento no [I1: mm]. Pero, de nuevo, las cifras son… ósea… hay tanto… voy a volver al concepto del oscurantismo [I1 y 2: mm] es tan poco lo que uno sabe. Yo no sé cómo falla, yo no sé cuáles son los criterios, yo no sé si cuando yo entiendo opinión política una cosa… yo no sé si el estado entienda lo mismo que yo [I1: mm]. Porque la gente cuando va bien no te viene a mostrar sus resoluciones [I1: mm] te dice “me fue bien” un punto. Tenemos un caso, por ejemplo, de un patrocinado cubano homosexual perseguido en su país de origen hay una norma específica que le prohíbe ser homosexual tiene así…. un alto de documento. Yo lo acompañe en su entrevista de legibilidad, yo solo he acompañado dos veces me han dejado IR a entrevista de legibilidad, el caso que les conté antes y este. Para mi este señor si o sí debería ser reconocido, lleva tres años en su proceso, y llego el otro día y me dijo “me inscribí en el proceso de regularización” [I1 Y 2: mm] (en tono de conmoción). Eso ¿qué significa? Que le pausaron su proceso [I1: claro].

Legalmente… yo tengo mi discrepancia de que se o deban pausar etcétera pero que pasa que… y no sé qué hubiese pasado con su caso [I1: mm] porque por el tiempo decidió… o al menos… está pensando [I3: en…] en salirse. Y si le llega ir bien yo no sé si va a venir y me va a mostrar su resolución.

I1: claro

E: ósea es complicado saber cómo funciona al final [I1: mm], cuáles son los números…

I1: Me pasa que cuando entrevisto a solicitantes [E: si] está siempre esa duda. Ósea, por ejemplo, seguramente tú tienes mucho más creencia en estas entrevistas pero… em… personas que está… que su historia de vida está trazada por el tema que rige ejército en Colombia, pero claro te lo cuentan con una naturalidad porque es la historia de su infancia así como en dictadura acá la historia de la infancia esta cruzada por esa (no se entiende la palabra que dice 1 hora 10´28´´) fuerza… entonces en su historia efectivamente no aparecen estos elementos como de una persecución especifica constante ¿no? en donde yo puedo entender desde aquí palante me transformé en una perseguida. Si no que hay una normalización…

E: exacto

I1: ¿Cachai? De estas condiciones. Entonces claro… ó por ejemplo tu historia venezolana que también eem… las extorciones, la pobreza, la imposibilidad de seguir trabajando… eran todos temas de seguir acrecentándose pero ellos nos decían “nosotros no somos so... eramos solo... no… no tan participantes del chavismo, pero nunca fuimos opositores explícitos ni nada” y claro en el fondo se fueron cerrando las posibilidades de trabajar em… sobrevivir y esa es su historia de refugio [E: claro] entonces yo les decía “nosé” porque si m… Realmente ellos me preguntaban ¿usted cree que me lo van a dar? Y era muy fuerte porque, claro, como no sabía realmente, porque en el fondo también puede ser ,por eso te decía, la historia… una historia normalizada dentro de la situación eem… en situaciones como lo que pasaba en Venezuela o en Colombia [E: exacto] porque pa mucha gente es una normalidad todo esto.

E: no y porque también en Chile, volviendo al tema legal de que no sabemos cómo se falla [I1: mm] hay dos definiciones de refugiados [I1: mm] la segunda tiene que ver con situaciones de contexto, ósea [I1 y 2: mm] el ejemplo cuando yo digo ya qué ejemplo, les digo a los alumnos, qué ejemplo se les ocurre ¡Venezuela! Es la primera cuestión que dicen [I1: mm] entonces decir… [I1: CLARO] entonces unos dice como pucha desde la información oficial que yo tuve alguna vez con la jefa anterior que una vez en una… reunión ella dijo abiertamente que en Chile no se habían reconocido nunca personas por definición ampliada [E1: que están en contexto] eeh… yo digo… y que va a pasar con las personas de Venezuela… las van a reconocer. Entonces frente a la pregunta que le hace a la persona mi respuesta seria “no sé tampoco. Legalmente bajo mi perspectiva usted cabe en la definición de refugiado y deberían reconocérselo” pero no sé si en la práctica van a practicar la definición, cuales son los estándares [I1 Y 2: mm], además que esto es una cosa que claro, yo no tengo una certeza del cien por ciento ero personas que trabajaban ahí antes y que ahora se fueron a mí me han ahora dicho cosas tan terribles como, por ejemplo, uno eeh… que hay una directriz interna que si no hay persecución directa la respuesta es no [I1: mm] ósea definición ampliada

¡chao! No existe. Segundo eeh… que había eeh… la directriz es una persona que, a ver, cómo decirlo… esto me lo contó una persona que estuvo y ya no está pero que [I1: mhm] tiene amistades dentro todavía y esta persona me conto que una de sus amistades era oficial de legibilidad [I1: mhm] ósea que trabajaba haciendo informes [I1 y 3: mhm] y que a esta persona tenia informe para reconocimiento y informes para rechazi [I1: mhm] y que cuando llego la nueva jefa le dijo "cuales son tus informes de reconocimiento" "estos" "transfórmalos todos a rechazo" [I2: mm] y que esta persona había investigado y que por eso había llegado a la conclusión de que sí.

I2: claro porque esos informes de reconocimiento tenían un sustento

E: tenían… ósea INFORMES po, ósea desde las directrices, desde información de país de origen y tuvo que cambiar a no y entonces su respuesta fue: "pero yo llegue a la convicción de que sí, me estay pidiendo de que cambie mi convicción" bueno esas son las reglas. Entonces yo no sé si esto será verdad o no. Pero no me lo ha dicho una persona, me lo han dicho varias desde distintas aristas. Tiendo a pensar que algo de eso HAY aunque no sea todo cierto [I1: claro] algo hay y me [I1: hay algunos reconocimientos ¿no?] preocupa. Y me preocupa. Y si a eso uno le va añadiendo como la vulnerabilidad que trae la persona, la poca asistencia jurídica y social que existe porque al final es fasic… [I1: si] eeh le vas añadiendo eeh digamos la lo el oscurantismo, el hecho de que las personas con las que tu te erelacionas tampoco saben [I2: mhm] la persona de afuera, alguien con la mejor voluntad del mundo, no sabe la diferencia legal entre un migrante y un refugiado entonces le dice "no, pídete la definitiva" y la persona, con todo respeto a la grabación… ¡CAGÓ! Porque al frente al dem hay una solicitud de visa entonces la solicitud es como "aah no… usted pide la visa entonces se vá" [i1: claro] también hay una actitud por los redactos de las personas, una actitud de… como frente a cualquier piedrecita… "a entonces terminamos el proceso entonces sory, [I1: mm] estamos tan colapsados que la primera [I3: es un alivio] yo me me desligo" ¿cachai? [I1: mm] terrible po. Ósea hay una serie problemática. Ahora… estamos hablando muy desde lo negativo (entre sonrisa) yo creo que igual hay cosas positivas y cosas que van a mejorar pero hay esta nuestro trabajo también po de todos y todas [I1: mm]. Sacar esto a la luz igual [I1: mm]

I2: en algún minuto dijiste que finalmente ahora como ya está todo el oscurantismo y también como nuevas barreras casi como los portonazos casi que están viviendo [E: claro] entonces como tenían vías vías jurídicas distintas al dialogo

E: ¿nosotros?

I2: mm

E: claro, las acciones (no se entiende lo que dice I2) judiciales [I2: aaah], las acciones judiciales [I2: ¿y cómo son estas cosas?]. Ósea por ejemplo ahora para el ingreso al procedimiento… presentamos una acción de protección por un to… bueno presentamos dos por un total de veinte personas de diversas nacionales pidiéndole a la corte de apelaciones y, eventualmente, a la corte suprema que ordene el departamento de extranjería ingresar a estas personas al procedimiento ósea… (Silencio 1 hra 15´51´´ a 1 hra 15´54´´) sory (risas), ingresar a las personas al procedimiento. Eso por una parte y lo otro eeh… le pedimos ahí a la corte di digamos que busquen la forme de… de… de digamos, a

ver, cómo decirlo de… de ordenar a l autoridad o de licitarle a la autoridad que termine con estas prácticas porque vamos a estar presentando protecciones de a veinte [I1: mm]. Ósea presentamos dos por un total de veinte personas, ahora ahora que ustedes se vayan tengo que terminar el listado con los alumnos que me mandaron ya, tengo los mail ahí, entonces tengo que abrirlo y anotar… tenemos como seis casos nuevos y hoy día sume uno séptimo en la mañana hablando al almuerzo con la Fernanda, ella tenía otro. Entonces vamos juntar pa hacer otra protección y después otra protección y vamos seguir presentando protecciones, ósea vamos a ver como se falla en esta. Dios quiera que nos vaya bien y que entonces el gobierno diga "bueno tengo que hacer algo porque no puedo seguir…"

I!: generando nuevas…

E: ¡no! Y dándole a los abogados [I1: mhm] ir alegar, presentar informes [I1: claro], nosotros no nos vamos a cansar de hacerlo. Entonces al final pierden ellos [I2: mm] ¿te das cuenta? (I3 carraspea)

I1: el seguro (no se entiende la palabra que utiliza en 1 hra 17´03´´)

E: ósea no es exigible que una persona tenga, para ingresar a un procedimiento de protección, tenga que encontrar asistencia jurídica, que lo atiendan porque nosotros hay meses y meses. Ahora estamos dando hora, alguien que llama hoy por teléfono le damos hora pa el once ósea pa la otra semana [I3: ya] eso es muy normal. Pero si hubiésemos tenido esta conversación este mes el año pasado… dos meses más o tres meses más. Una vez en Enero estábamos dando hora pa Abril.

I2: qué pasa en vacaciones con la clínica…

E: en Febrero cerramos. En febrero no hay atención. Pero todos los casos abiertos… los alumnos parten en Enero [I1 Y 2: mm], entonces de Enero a Julio es un semestre los alumnos llevan los casos y el Febrero tienen responsabilidad sobre los casos igual [I2: mhm]. Nosotros el equipo docente tenemos un sistema de turno, entonces una semana estoy yo, después la Fernanda, Victor Hugo, la Claudia y estamos ahí como atento y atentas a que el alumno nos pueda llamar como con una urgencia. Eeh… y en Julio hasta mediado de Julio parten los nuevos, entonces se hace el traspaso de carpeta. La carpeta siempre está con alguien [I1: mm] ese es el punto [I1: ya] [I2: mm]. Eeh pero no hay orientación jurídica en Febrero, eso no hay. Ni aquí ni en fasic. (Se deja algo como un papel sobre la mesa y hay silencio desde 1 hra 18´11´´ a 1 hra 18´16´´)

I1: dale, yo creo que…

I2: no… creo que ya…

I1: porque…

I2: si sí
I1: ya lleva mucho tiempo

I2: si peor muchísimas gracias…

I3: oye muchas gracias de verdad e... Es increíble cómo se cruzan los temas. Ósea lo que habíamos percibido allá trabajando con los mismo solicitantes y con otras instituciones ya lo refuerzas tú da una idea bien clara como lo que está pasando y… realmente GRAVE la manera en la que no se está cumpliendo todo este marco legal que se… que se logró digamos

E: exacto [I3: mm]. Yo de pronto, bueno no se si alcanzamos por temas de tiempo, pe por eem… cuando mi alumna que está haciendo su tesis sobre em… sobre refugiados [I2 mhm] yo igual se las puedo mandar [I2: mhm]. Ahora estamos ella me mando el borrador y ya lo corregí… debería mandarme la versión final pronto y deberíamos tener la lista pero ella también hizo entrevista a instituciones [I2: mhm]

I1: aah sería súper

E: sí

I1: mira nosotros la idea es que, bueno aparte que este pequeño proyecto como producto digamos de la informe y eso, salga también un pequeño conversatorio en fasic del tema [E: súper] em… tratando de involucrar sobre todo, no solo a la gente que está en contacto que ya sabe sobre todas estas cosas, sino que sobre todo a las personas que están contacto y NO SABEN cómo lo los servicios de salud. Entonces nada en esas ocasiones seguramente te vamos a contactar para invitarte y si no puedes ir tus alumnos tu equipo seria súper bueno [E: sipo] seria súper bueno [E: súper] que estuviéramos también pa poder tratar de externalizar todo este conocimiento a todo este universo de funcionarios que no… que no están en contacto con… [I2: mm]

E: súper… me parece excelente
I1: asique eso
E: Yapue…
I1: yapo muchas gracias
I2: Muchas gracias
E: ya…

SALUD MUNICIPAL

PP 37-52

P: A ver… Yo pienso que… lo que en parte te voy a… a transmitir tiene que ver con una línea, que tiene que ver directamente con mi responsabilidad y mi trabajo eeeh… En la atención primaria ¿Ya?

I: Mhm

P: ¿Sí? Y parte también de lo que son mis propias búsquedas, inquietudes y lo que hago despúes de las cinco de la tarde ¿ya?

I: Claro

P: Por tanto, también voy a manifestar una de mis posiciones respecto al tema ¿ya?

I: Claro

P: Y… entonces, y es difícil conjugar, que yo te pueda decir (ininteligible 0:35) de la pega, pero de un modo, parte de lo que hemos montado también tiene que ver con… con… como con algunas compañeras estamos mirando el… el tema hace unos años atrás. ¿Ya?

I: Ok

P: Yo, bueno, mi nombre es YY, de formación inicial yo soy licenciado en filosofía, soy magíster en psicología social. Me he dedicado… chao chao (se despide de otra persona). Me he dedicado a temas de migración hace ya… ya seis años, desde distintas perspectivas, pero ciertamente más desde la intervención. Y… Mmm… Y mi línea, como desde donde yo estoy mirando el tema eeeh… se inserta lo que tu vas a poder encontrar en las investigaciones de YY, que es profesora de la Javeriana de Colombia…

I: Mhm

P: Eeeh… Quién, cuando la cono… cuando me licencié me cambió la perspectiva de donde estábamos mirando el tema de la migración y un poquito donde yo hoy me posiciono. ¿Ya? Tiene una investigación que se llama eeeh… que es sobre los procesos de racializaicón de la población afro… afrocolombiana en Antofagasta. Eeeh… y donde de una, desde un enfoque trasnacional de los estudios migratorios ella logra desmontar el.. como muy pocos autores lo han hecho aquí en Chile. Eeeh… Como se está mirando el tema de eeeh… de la situación del colombiano y colombiana en el norte de Chile ¿ya?

I: Mmm si

P: Entonces, dicho eso, yo te diría a mí me confun… me confunde un poquito la entrada al tema de hablar de refugiados…

I: Mhm

P: Y solicitantes de refugio, que, si bien yo entiendo que es una distinción jurídica, parte, bueno de la convención… internacional para la (ininteligible 02:28) de los refugiados y la ley… la ley del 2010 lo estipula… A mi me complica, porque en la práctica, la lógica en como el Estado gestiona el refugio eeeh… y cuando hablo, tienen muchas… muchas vías, pero una de estas es lo que tú dices, que, en el fondo, que hay una vía de reasentamiento institucional que tiene alguna experiencia… súper concreta con los sirios, los palestinos eeeh… La situación de los solicitantes de refugio, especialmente, particularmente los colombianos y colombianas es súper… super… compleja, porque tenemos en la práctica muchas personas que son víctimas del conflicto armado… Que… han sufrido desplazamiento forzado interno en su país de origen… Que están inscritos en el registro único de víctimas de la ley 1448, eeeh… Tienen su certificado de víctima, pero aún así el Estado de Chile les ha negado el refugio o ellos por las dificultades y arbitrariedades de la gestión cotidiana desde la frontera hasta la Gobernación Provincial de Antofagasta…

I: Mhm…

P: Eeeh… Nos piden, terminan (ininteligible 03:47-03:52) Migrantes morales. Entonces yo me he encontrado con algunas investigaciones, y te puedo abordar el tema acá, es que… como… si pudiese ser factible una división entre los migrantes laborales y los… los solicitantes de refugio y… refugiados, y cuando en la práctica, lo que nosotros nos hemos encontrado es que… Sobre todo, en la migración colombiana del pacífico… eeeh… Tengo montón de chicos y chicas que son víctimas del conflicto armado y no han podido el (ininteligible) de refugiado jurídico si se quiere o simplemente por desinformación o negación, o por arbitrariedades de tipo de… de análisis de preadmisibilidad…

I: Mmm

P: De... de posibilidades de solicitud del refugio, simplemente lo desincentivan… Y por qué comento esto que es importante, porque en la práctica tenemos personas que llegan a los centros de salud con… las… con una situación de vulnerabilidad social, experiencia traumática del conflicto armado… Y que... por no tener el estatus de refugiado lo vemos como migrantes normales…

I: Mmm

P: Entre comillas…

I: Si…

P: Como migrantes laborales… Y ellos son los que en definitiva están en una situación de vulnerabilidad eh… mucho mayor que inclusive los solicitantes de refugio… En la práctica nosotros lo que… lo que hemos ido haciendo y entendiendo, no de manera muy extendida, sino en algunos centros de atención primaria, es coordinarnos con la oficina de refugio de FASIC…

I: Mmm

P: Porque… (ininteligible) FASIC nos derivaba a todos los solicitantes de refugio, entonces la persona iba a la gobernación, formalizaba su solicitud de refugio, iba a FASIC y FASIC nos (tengo duda si dice no o nos min 05:38) los derivaba a los centros de salud. Entonces yo fui donde X… XX… la persona encargada de la oficina del FASIC acá… pero tú para que los derivas…

I: Mhm

P: Entonces, el único interés de X era derivarlo, era para que le hicieran un análisis… general…

I: Mhm

P: ¿Sí? Eeeh… que les sirviera a las personas solicitantes de refugio para presentarlo ante la comisión al momento de la entrevista.

I: Mmm

P: en el fondo, que hubiese un profesional del centro de la salud, ya sea médico, psicólogo… dependiendo de… de la situación, que pudiese ver a la persona y después emitir un certificado con lo que encontró y que eso lo (ininteligible) la persona al momento de la inscripción.

I: Mmm

P: Y yo le decía que eso era muy difícil y que, te estoy hablando conversaciones que se dieron en torno al año 2016…

I: Ok…

P: (Ininteligible) Que eso era muy difícil de hacer para nosotros en los centros de salud a términos de que en ningún documento se nos manifiesta la obligatoriedad…

(Suena teléfono 06:43)

I: Disculpa

P: Sabe que usted tiene que hacer como parte de este procedimiento o tal procedimiento…

I: Ok

P: Es lo que se mostraba. Y lo que yo te puedo garantizar es que a todos lo solicitantes de refugio los van a atender a los centros de salud y van a ingresar al programa eeeh… conforme a su… a… al programa de salud del niño... salud mental, del programa de la mujer, del adulto, del adulto mayor… conforme a esa necesidad, eso va a ser a parir del primer control. Y yo, cuando lleguen estas personas… las vamos a afiliar a FONASA, les vamos a dar una atención y… y los vamos a hacer usuarios nuestros. Pero lo que yo no te puedo garantizar o… o decir que yo lo… (ininteligible) puedo comprometer, a que nosotros desde los centros de salud vamos a emitir esos certificados que tú estai interesado. Y… dado esa situación, así hemos venido trabajando, entonces en la práctica un solicitante de refugio, después que ha ido a la oficina de… de FASIC pa recibir la orientación inicial y el acompañamiento X dentro de las múltiples derivaciones que hace, lo deriva a los centros de salud. Y ahí empieza una… una vuelta… eeeh… yo trabajo con un usuario nuestro donde la mayor singularidad… oooh… si se quiere, el mayor énfasis que hemos tenido ha venido por dos líneas. Una, ciertamente con las personas donde el desplazamiento forzado eeeh… ha sido muy reciente…

I: Mhm…

P: por ejemplo, chao chao (se despide de un tercero) personas que han tenido que salir de… de Colombia, en n plazo de uno o dos días, muchos de ellos arranca… nos han tocado algunos casos de algunas personas por ejemplo que vienen con heridas de bala muy recientes…

I: Mhm

P: Y que por tanto necesitan curaciones eeeh… Necesitan eeeh… amputaciones o necesitan puntos, o sea, te estoy hablando a ese nivel de…

I: Claro

P: Eeeh…

I: De emergencia

P: de complejidad, de emergencia, claro, tú lo dices bien, de emergencia eeeh… Ya, esas respuestas las damos y… un examen físico, una consulta de morbilidad al inicio de su llegada y eso nosotros lo otorgamos, pero la otra línea fuerte es la de salud mental…

I: Claro

P: Eeeh… y en la de salud mental ahí hay… un tema que los psicólogos trabajan en… como pueden y van abordando las situaciones en la medida de que sus herramientas de trabajo de salud mental… le permiten. Porque al no haber una formación… eeeh… profunda para abordaje de… en salud mental para personas que han sido desplazadas o que… estén refugiados (ininteligible 09:50) con experiencias super complejas en sus países de origen, de la inexistencia de eso…

I: Mmm

P: Como una metodología de trabajo para abordarlo, es súper autodidacta y la primera pega que hacen los equipos de salud mental es la contención, o sea, son personas que llegan muy cargadas…

I: Mmm

P: Con datos muy… casi eeeh… increíbles de cómo se dio todo su proceso de desplazamiento y que ellos… después de esa… esa, esa construcción inicial eeeh… comienzan a hacer el proceso ya sea de derivación a COSAM o siguiendo en controles en atención primaria. Pero ese proceso de trabajo, yo diría, administrativamente nosotros lo resolvimos con FASIC…

I: Mhm

P: Cuando nos pusimos de acuerdo

I: Claro

P: pero todo solicitante de refugio llega a nuestro centro y nosotros (ininteligible 10:41) Ahora, el abordaje técnico si… eeeh… para el trabajo con solicitantes de refugio o refugiado ha sido autodidacta total…

I: Claro

P: Por que no existe… tampoco hay interés, no de parte de los equipos, sino digo como de las autoridades por ir abordando eeeh… esa temática de manera más… integral o... de manera más rigurosa. Hay más bien como una omisión que te deja en zona gris de que hagas lo que puedas ¿no?

I: Claro… claro. Y respecto a esta solicitud qué hacía el FASIC emmm… ¿De emitir como un certificado médico o digamos una… emmm… un certificado que pudiera servir para las solicitudes?

P: Ahá

I: ¿Eso es una… petición que te hace… está persona X que me cuenta, espontáneamente o es que efectivamente en los procesos de solicitud de asilo emmm… vale algo digamos emmm… lo que pueda decir o no decir un médico o un centro de salud chileno?

P: Eeeh… yo creo que la intención de X eeeh… es ir ayudando, abordando… ayudando a los (ininteligibles 12:00) prófugos que puedan dar la mayor cantidad de información posible…

I: Mmm

P: Que… aumente las escasas posibilidades que tienen de que le otorguen el refugio

I: Mhm

P: O sea, las… hubo el diez por ciento de los casos que ha tenido X en los últimos cuatro años… han obtenido el refugio… En Antofagasta no se está consiguiendo el refugio…

I: Claro…

P: Eeeh…en el anterior gobierno de… de Piñera y al inicio del gobierno de Bachelet se generó hasta un examen de preadmisibilidad que no está en la ley, o sea, a los solicitantes de refugio antes de acoger su solicitud de refugio…

I: Si…

P: Le hacían una preentrevista

I: ¡Mmm! Y ahí ya se descartaba la posibilidad del refugio…

P: ¡Claaaro! Entonces, en el fondo hay una primera traba para no visibilizarlos. Entonces estas personas terminaban así… por eso yo te digo que a mi me complica, es súper complejo la… esta distinción entre refugiados y solicitantes de refugio y migrantes laborales como lo primero que yo pongo en cuestión…

I: Sí

P: Cuando hablamos de… de personas que… vienen al país comilla por motivos laborales, están todos estos motivos… y el desplazamiento es clarísimo después de cuando empiezan las entrevistas o un trabajo más en profundidad…

I: Mmm

P: A la raíz de muchos de los migrantes, y yo te estoy hablando específicamente de los colombianos, que está invisibilizado como una migración laboral en el fondo… eeeeh… una raíz humanitaria brutal…

I: Claro

P: O sea, tú ves en la medida en que se empieza a caldear el conflicto armado Colombia… en el pacífico digo, en Ariño, en el Valle en el… en valle… en (ininteligible) mismo y en el Chocó tú ves como va aumentando la migración al norte de Iquique y… O sea, eeeh… y el 2003, siempre lo pongo como año importante, sobre todo en Buena Aventura que… un grupo importante de personas de Buena Aventura vive acá, se generaron trece mil procesos de desplazamientos forzados eeeh… entonces la migración tiene un tinte humanitario invisibilizado…

I: Mmm

P: Eeeh… socialmente y además, con autoridades que ponen… que han puesto otros requisitos para impedir que la persona llegue a solicitar el refugio… Entonces X lo que intenta… es… que a… acumulen la mayor cantidad de información posible de documentación, para aumentar un poquito sus posibilidades de que puedan eeeh… puedan ser… acogidos como refugiados. Y en la práctica… eso no te asegura nada… es lo que te decía de un inicio ¿no? Tenemos personas que son víctimas del conflicto armado, eh tienen su certificado de desplazados, tienen los relatos de la víctima, pero el Estado chileno aún con esos antecedentes le niega el refugio.

I: ¿Y sobre que se basa este… ese diniego, esa negación del refugio?

P: Ya, por ejemplo, tenemos el ejemplo de niños que han sido reclutados… por… hay casos distintos, no voy a generalizar…

I: Claro

P: Te cuento algunos casos que yo he tenido noticias…

I: Si…

P: Eeeh… chicos que fueron reclutados en su infancia o en su adolescencia por los (ininteligible) o los (initeligible)

I: Sí

P: Que llegan (ininteligible) después y que les niegan el refugio porque antes de víctimas fueron victimarios aún cuando… eeeh… internacionalmente se ha resuelto que los niños que fueron reclutados… o sea, que las personas que fueron reclutadas en su infancia y adolescencia también son víctimas…

I: Claro…

P: El estado chileno no los reconoce como tal (tengo la duda en estado chileno min 15:45) Eeeh… lo otro es que… en procesos de entrevista eeeh… cuando… ellos se quiebran, esto es lo que relatan algunos refugiados solicitantes ¿no?

I: Si…

P: Cuando se equivocan en las respuestas o… se equivocan en las fechas o… hay algo que revele una inconsistencia, ya de frentón… se le… menos posibilidades…

I: Claro…

P: Menos posibilidades, entonces la… la verdad… es que… no hay un criterio exacto… determinado… visible, por el cual a las personas les niegan el refugio, lo que sí tenemos que hay personas que han sido víctimas, pero en algún momento han sido victimarios, a esas personas de frentón no se les, no les dan el refugio. Eeeh… pero nos han pasado casos que… tenemos… noticias de uno o dos casos que han sido… o sea, que fueron reclutados en su niñez…

I: Ya…

P: ¿Te fijai?

I: Mmm

P: Y aun así… (ininteligible 16:44) y aún así eeeh… es súper discrecional el último término de la obtención del refugio nooo… pufff…

I: Eso te iba a preguntar, por ejemplo, me estoy imaginando los… o sea, los niveles de experticia o… o en el fondo como se constituyen estas comisiones de emmm… extranjería, que me imagino es quién… que constituye la comisión para la… la aprobación o la admisibilidad de las solicitudes. ¿Quiénes están ahí? ¿qué es lo que buscan esas entrevistas emmm…? ¿Cuánta experticia está en el entender? además también… ¿Cuánto se cumple o no se cumple la ley? Porque se supone que a nivel de ley Chile adscribe digamos a toda la… el derecho internacional en toro a protección de… de refugiados… Entonces, por lo que tu me estás contando hay un nivel de incumplimiento de la ley importante en este proceso.

P: Eeeh… sí y no…

I: Mhm

P: O sea, eeeh… sí, pero invisibilizado…

I: Claro…

P: O con ciertos argumentos que permitirían o avalarían esa discreción ampliada, pero yo estoy de acuerdo contigo, o sea lo… lo que hay aquí… es por parte del estado… eeeh... A ver, es que el estado chileno tenía esas mismas prácticas y las vuelve a repetir ahora, o sea, ¿qué es lo que hizo el estado pa parar las solicitudes de refugio de los venezolanos?

I: Mhm

P: Puso la visa de responsabilidad democrática, la visa de responsabilidad democrática actúa como un dispositivo para anular las solicitudes de refugio y pararlas, es una forma de gestionar en el fondo esta crisis humanitaria…

I: Mmm

P: Entonces, el Estado, lo que hace es arbitrar discrecionalmente o aprovechando, no las negaciones, sino los vacíos o los intersticios que deja la ley eeh je…

I: Mhm

P: Para generar practicas arbitrarias…

I: Claro

P: O sea, claro, el Estado Chileno, lo que te puede… eeeh… y si uno lee el discurso del nueve de abril del presidente Piñera…

I: Mhm…

P: Eeeh… La visa para los venezolanos se llama visa de responsabilidad democrática, se elabora toda una condición discursiva respecto a porque el estado chileno está en (ininteligible min 19:00) en torno a reciprocidad con las personas colombianas, pero cuando tu ves en la práctica de que… impli… las consecuencias de esos discursos o de ese dispositivo…

I: Mhm…

P: Es que, en el mismo término, lo que están haciendo es… una forma legal de negar la posibilidad de que una persona llegue a solicitar el refugio, que una persona venezolana pueda solicitar el refugio…

I: Claro

P: Y además, como están desesperados a nivel local, y de esto yo no tengo certeza de lo que te voy a comentar, estos son relatos que yo he construido con las personas de la sociedad civil…

I: Si…

P: Eeeh... las personas… a las personas que están solicitando el refugio les metieron en el proceso (ininteligible min 19:38) de regularización…

I: Mmm

P: ¿Qué quiere decir? Que las van a meter en (ininteligible 19:42) les van a negar el refugio, por tanto, son migrantes laborales…

I: Mmm…

P: Discursivamente, cual es el efecto que eso provoca, estamos llenos de migrantes laborales y estamos escondiendo una y otra vez… sistemáticamente la crisis humanitaria que hay detrás de la comunidad de migración latinoamericana…

I: Mmm

P: Principalmente en el caso colombiano, que es el que yo más me he dedicado a… a profundizar y estudiar, pero eso en el caso de venezolanos está clarísimo…

I: Sí…

P: Eeeh… entonces sí, yo coincido contigo a raíz de tu… de tu pregunta, de que… de que hay negaciones eeeh… y arbitrariedades que (ininteligible 20:16) el derecho internacional en materia

de derechos humanos, pero ojo, eso si lo leemos en clave de un enfoque trasnacional es... algo que se mueve entre origen y destino…

I: Mmm

P: Está construido así, o sea, la invisibilización de… de… del conflicto y… la negación de la crisis humanitaria… son discursos y prácticas que parten en los estados de origen…

I: Claro

P: Eeeh… y eso es brutal… eso es brutal, eeeh… porque son personas que…

I: (…) Claro, porque de alguna manera… emmm… invisibilizar el refugio colombiano, por ejemplo, o los niveles y los números…

P: Ahá

I: Emmm… de alguna manera tiene el efecto también de negar el estado de conflicto que hay en Colombia.

P: Es... total, mira yo te voy a contar una infidencia…

I: Mhm

P: El estado chileno con el estado colombiano… fijaron, hicieron, con el (ininteligible 21:13) de la OIM, gestaron un estudio de la red migratoria de Chile y Colombia…

I: Mmm

P: ¿Sí?

I: Sí

P: Y de hecho, si tú te metes a la página de la OIM está, que… está la información de cuando se iba a lanzar ese estudio…

I: Ya…

P: Pero ese estudio nunca lo publicaron…

I: Mhm

P: Porque lo que había, era manifestar brutalmente que detrás de la migración del Pacífico Colombiano, que es principalmente (ininteligible) a Chile, está el conflicto armado.

I: Claro

P: Y lo que hace la cancillería cuando se juntan ambas comisiones en Bogotá, dejan afuera a la OIM… entra y acuerda no publicar ese informe y ese informe nunca se publicó…

I: Mmm…

P: Hay una invisibilización por parte de los estados de esta cuestión

I: Mmm

P: Y es clarísimo, es… es… o sea, es que… disculpa con la pasión que te hablo de esto, es que es indignante…

(se traslapa el discurso entre participante e investigadora min 22:08)

I: Si, no, es que estoy pensando que efectivamente eso resuena con lo que nosotros logramos mirar acá… Porqué emmm… a nivel de política pública la… el refugio se ha centrado en una preocupación por los reasentados…

P: Exacto…

I: Entonces hay… además es que son mucho más fáciles de justificar en términos de… necesidades… cursos de lengua, ¿no cierto?, tratamiento del trauma… porqué estamos hablando de sirios, entonces, estamos… reconocemos que hay un conflicto en Siria… y qué se yo… Pero, para los solicitantes latinoamericanos hay una…forma de evitar este reconocimiento de un estatus de refugiados para ellos…

P: Claro… si… no… y es brutal po… es brutal… Tú después escuchas a… no sé… a una… una compañera colombiana afro… ¿no?... qué además… disculpa la dispersión, pero…

I: Mhm

P: Es muy difícil además en el propio… esta invisibilización… o los discursos o prácticas que invisibilizan este tema, porque tiene un efecto también en la vida de los propios migrantes, bueno que eso… ese es mi rol ¿cachai? Eso produce subjetividades…

I: Sí… si, te iba a pregunta también de eso ¿cuáles son los efectos sobre las personas?

(se traslapan discursos participante e investigadora min 23:24)

P: ¡Sí! Bueno… esa es mi pregunta también… jajaja, esa de hecho es la pregunta que estoy elaborando ahora en el fondo… cuáles son los efectos que producen esos discursos y esas prácticas a nivel cultural, a nivel estatal y esas invisibilizaciones, eeeh…. Y… entonces, una de las cosas es… es la negación y el silencio del conflicto por parte de los propios migrantes, entonces no es fácil acceder a un comillas migrante que tu… que uno ve como comillas un migrante laboral…

I: Mmm

P: Eeeh… que te suelte el rollo…

I: Claro

P: Que te diga ¿Sabís qué? En verdad… en verdad, en verdad, detrás de toda mi inmigración está este rollo…

I: Claro…

P: Eso es muy difícil acceder, por parte de un… yo… yo pienso que es muy difícil de acceder por parte de los investigadores chilenos…

I: Mmm

P: Porqué… ummm. Si se hace una revisión de como se ha visto la migración colombiana los últimos…diez años, eeeh… yo me he encontrado, con que por una parte hay investigaciones que no toman en cuenta las condiciones de origen o otros solo la usan para la contextualización, pero no exploran en las consecuencias o cuando hacen el análisis de los resultados obvian la situación del conflicto armado, entonces no logran dar con el… hay una… hay una investigación de la (ininteligible min 24:53) Liberona…

I: Mhm…

P: Eeeh… que… en la frontera… se da eso y el (ininteligible min 24:57) como aliado, dan cuenta y relata… y dice las personas son víctimas del conflicto armado, contextualiza la migración colombiana y lo que pasa en la frontera del conflicto armado. Pero después, publica un resultado, y ¡una mujer le está diciendo ahí mismo en el texto le dice! ¡¡Tuve que salir en cinco días de Colombia y tatatata!! (Da énfasis y se escucha que aplaude acompañando las palabras) Y no lo problematiza…

I: Mmm

P: Y yo digo… ahí te está diciendo… que ella tuvo que salir, porque probablemente tuvo que salir por un desplazamiento forzado… Pero los investigadores no han mirado eso…

I: Mmm

P: ¿Cachai? Entonces, vemos… no logramos dar en el resultado por… a mi modo de ver… por como los investigadores están abordando el tema y por otro… no logramos dar en el clavo pienso yo, o … o es difícil acceder, porque… la invisibilización histórica tiene… va… va teniendo efectos y resultados. Y bueno, y esto… ahora si que te cuento los dos relatos que te quería comentar…

I: Mmm

P: Uno… un… eeeh… un chico… que lo conozco hace tres años… cuatro años quizás, eeeh… de tardes largas sentado en… en un campamento, sentado en su casa… (ininteligible min 26:04) que es dirigente, entonces, haciendo talleres con personas migrantes en temas de migración y salud. Después yo me iba a su casa… o sea, una amistad una cercanía… a tal punto que fue a mi matri… fue a mi matrimonio… o sea, una relación de mucha cercanía, que a mí siempre había cosas que no me terminaban de cuajar en sus relatos, pero era un migrante laboral… una persona excelente acá... Resulta que viene Y… y vamos a hacer trabajo de campo y yo le digo, te quiero comentar Z… y el rollo que Z no me tiró en tres o cuatro años de amistad, se lo tiró a ella en una conversación donde yo quedé… totalmente fuera… Y el loco contó la firme y le contó toda su trayectoria desde porqué tuvo que salir en dos días de Colombia, lo que sufrió en el aeropuerto de (ininteligible min 26:50) tuvo que salir disparado tenia recursos… tenía en Panamá, en Panamá pens… eeeh…. Estuvo muchas horas en escala… sufrió unos vejámenes así horribles… y hasta que llegó a Chile y toda la historia.

I: Mmm

P: Pero… no… no… yo no te podría decir que pasó que esta persona termina… enganchando y mostrando… esto pasó, esta es mi migración… mi… yo tuve que salir forzadamente en un día porque iba con mi guagua eeeh… ah…. Y en el fondo al… al narco… para el cual el trabajaba… se

vio visto en una balacera, él saca la pistola del coche de la guagua ¿no? Y le empieza a disparar y entonces después lo andaban buscando pa matarlo y la mamá hace que salga en… dos días…

I: Mmmm

P: Lo fondearon y lo (ininteligible min 27:46)

I: Mmm

P: A ese relato yo puedo acceder al… aaah… a esa dimensión de su historia después de cuatro años…

I: Sí…

P: Respecto a la W que es otra compañera… amiga mía nuestra, pasó lo mismo, dos tres años y… y… ¿de dónde eres tú W? No, yo soy de (ininteligible min 28:05) soy de… soy de… soy de… todos sus relatos que ella venía o provenía de (ininteligible) Y resultó que después de dos años en una conversación… eeeh… junto con… con Y que… en una entrevista… que… hicimos larga… ella viene y nos dice… no, que ella tuvo que… ella claro, (ininteligible min 28:30) fue el último lugar donde vivió después de un desplazamiento…

I: Mm

P: Porque… eeeh… un paramilitar la quería… a ella como mujer y su papá la tuvo que… así relato de ella, sacar escondida en un saco en la noche porque sino se la iban a robar… a ella…

I: Mmm

P: Para casarla con un paramilitar en Buenaventura. Y después de (Ininteligible min 28:52) ella (no se escucha min 28:55) procesos desplazamientos forzados internos que después gatillan en procesos de migración… internacional…

I: Internacional…

P: Y quien era W para el mundo, una migrante laboral que viene a buscar pega y que más encima no puede obtener la definitiva después de nueve años… Con una (ininteligible min 29:09) horrible… Entonces si, eso… eso se invisibiliza y es muy difícil (no se entiende min20:15) esas historias esos relatos… mirar… estos movimientos y las cosas que están pasando… entonces sí, no es nada fácil. Y yo creo que esto es producto también de una cuestión de clases… de una invisibilización histórica… Eeeh… los imaginarios también entorno al desplazamiento dentro de los propios colombianos tampoco… es bien visto, me encontré con un colombiano blanco de acá que… que… es dirigente social y dice “yo tuve que desplazarme forzadamente cuatro veces, porque me querían matar” como dirigente… Entonces, la pregunta (ininteligible min 20:57) ¿entonces tu te acogiste en la ley 1448? Eeeh… ¿Estás inscrito en el registro único de víctimas? Y me dice “nooo”… “Es que eso es para los que necesitan más”

I: Claro… claro que sí

P: Entonces hay un tema de clases y también que… esas propias personas eeeh… ocultan o se niegan a asumir su estatus de desplazado… de víctima, también por temas de clase…

(Se interponen discursos investigadora y participante)

I: Claro, también estaba pensando que…

P: (…) sociedad receptoras…

I: (…) Es un discurso también naturalizado el de la violencia ¿no? O sea, como el sentir que es una situación estructural de todos y entonces eeeh… como se juega quien es realmente víctima… y quién no ¿no? Es un… límite muy… sutil…

P: Mmm… Yo pienso que está la… con… sí. No, pero yo creo que ellos tienen… tienen tal hervidero…

I: Mmm

P: Que ellos tienen clarísimo su… o sea, claro, cuando tu dices hay siete millones de desplazados… tu dices… es que ella… ya… si se sabe que son víctimas y que el desplazamiento es una de las experiencias más traumáticas que… que hay dentro del conflicto armado digamos…

I: Mmm

P: Y las personas del pacífico lo están viviendo por montón y ellos lo saben… El tema es que eeeh… por un montón de motivos terminan siendo migrantes laborales…

I: Mmm

P: En el país de destino… cuando son víctimas y podrían perfectamente solicitar el refugio. Este… el problema es que cuando lo solicitan se los niegan…. Jaja…

I: Claro…

P: Y entonces ahí y pa… pa terminar… el... el punto ¿no?

I: Mmm

P: Cuando… quiero profundizar en lo que te digo, cuando llegan si se los niegan, y ahí se produce un problema… un problemón que tenemos en destino y es este… Esto es una caricatura que te voy a hacer…

I: Sí…

P:Entonces tenemos personas víctimas del conflicto armado… que han sido desplazadas, que tienen como certificarlo, que llegan a la frontera, que no lo dejan entrar ni como turista ni como solicitante de asilo… que (ininteligible min 32:04) una, dos, tres, cuatro veces… cruza a (ininteligible) y (ininteligible min 32:10) y les vuelven a negar el ingreso, entran por paso no habilitado, muchos de ellos han consultado por redes de coyote en fronteras…

I: Mhm…

P: Eeeh… Solicitan el refugio de la gobernación provincial, pero tienen ingreso por paso no habilitado…

I: Mhm…

P: A esas personas entran por paso no habilitado… eeeh… van a la gobernación… finalmente le aceptan la solicitud de refugio… se las niegan… se las terminan negando porque al noventa por ciento le niegan el refugio y… aquí aparece el fantasma del ingreso por paso no habilitado…

I: Mmm

P: ¿Qué hace una persona que le niegan el refugio, que es solicitante de refugio y que ingresó por paso no habilitado? Las posibilidades ahí se complejizan mucho, porque se viene ahí es la posibilidad de la orden de expulsión…

I: Claro, de la expulsión…

P: Y por tanto, de volver a vivir en situación irregular…

I: Mmm

P: Entonces… es súper compleja la situación que terminan viviendo las personas que solicitan el refugio, que se los niegan y han entrado por paso no habilitado…

I: Mmm… O sea, es aún más… en el fondo la… la ley los pone en una situación de reproducción de su propia vulnerabilidad, porque vuelven a… a…

P: Sí…

I: A estar en una situación por la que son expulsados, luego vuelven a… a… arriesgar a tener que pasar de nuevo por paso no habilitado…

P: Sí…

I: Es como…

P: Y entonces…y esos son muchos de nuestros usuarios que llegan a los centros de salud po… o sea, como te digo… los que llegan son con muchas necesidades físicas…

I: Mmm

P: …Muchas veces necesidades de querer contar…

I: Sí…

P: Y de hablar… y de sesiones… y de… (ininteligible min 33:52) que… si te interesa te puedo mandar el dato de una compañera que es de la dirección de salud… que es la referente de salud mental, ella trabaja como psicóloga en el CESFAM Centro Sur…

I: Mhm

P: Donde… fue donde empezó a sistematizar esta práctica… de... de atención a solicitantes de refugio y refugiados… Y… y… y nada po, la Q me cuenta que… que era..heavy porque… no había rendimiento que soportara…

I: Mmm

P: Un ingreso a salud mental de un solicitante de refugio porque era…

I: … Claro…

P: … Una hora… una hora y media, y que no podí parar porque... ejé, es tal lo que se quiere mostrar, es tal lo que se quiere… contar… que… que la contención se hace imposible en los espacios de rendimiento normal… No tenemos ni orientaciones técnicas… tenemos demanda y tenemos… más encima estamos apretadísimos por rendimiento que no nos alcanza para resolver con pertinencia… ese caso, todo lo que hacemos es autodidacta… Lo que… lo único que podemos resolver fue el ingreso administrativo. Yo te compartí… el borrador…

I: Mhm

P: De un… a ver… creo que te lo mandé ya… no te lo mandé, te lo voy a mandar, un… eeeh… de atención… un flujograma…y un borrador de un protocolo para atención de los solicitantes de refugio…

I: Mmm O sea, ustedes han podido en el fondo…

(Se interrumpen ambos discursos 35:13 min)

P: En el fondo…

I: … coordinarse dentro de lo posible...

P: Exacto… Sí.

I: No, es increíble, porque eso además de tener, seguramente efectos sobre los equipos de salud, o sea esta carga, esta imposibilidad de tener las herramientas… el tiempo, los recursos… pa poder dar un… un servicio que… el equipo considere que… que es el que se merece la población que está llegando. Emmm… también tiene un efecto sobre los propios equipos de salud, en términos de burn out, de frustración y de… de también sentir que no… que no pueden hacerse cargo ¿no?

P: Sí… así es… Está… está súper pelúo…

I: Está súper pelúo… sí… realmente está súper pelúo… Oye te agradezco montones, en verdad me interesaba mucho como… esta primera… casi exploración de esta experiencia increíble que tienen en el Norte y que… son pocos digamos los que tienen como tan pensado… también… están digamos arriba de la ola, a pesar de este estado como emergencial total que existe en el tema. Y… mmm… Y nada po… me… me confirma lo importante que es empezar a visibilizarlo también por los niveles de violencia que se están generando a nivel burocrático, institucional y de incumplimiento también de leyes… en cuanto… en esta materia ¿no? Porque al final está generando una población profundamente vulnerable…

P: Si po…

I: Y… no se está… como decíamos, visibilizando de ninguna manera, porque en el fondo todo lo que se hace es visibilizar lo que… lo que se ha hecho como a nivel de política institucional…

P: Sí… ahí yo… como te comentaba en un inicio, te recomiendo que le podai echar una miradita a lo que… a lo… eeeh… a lo que Margarita a…

I: Absolutamente y si, muchas gracias por ese… por ese dato. Creo que lo vi… ¿Estará también publicado en inglés? Porque hay un texto en inglés que no me acuerdo en que revista…

No… sí, no sé. Lo que pasa es que lo primero que empezó a hacer fue llevar el trabajo de campo a un congreso, y parece que de ahí tiraron uno, per después ella escribió, en todo caso lo sistematizó en un… en un texto. Yo te lo voy… en el correo que te estoy mandando ahora te lo voy a mandar, lo que yo tengo de la Margarita. Pero respecto a la situación de los afrodescendientes en Antofagasta hay… una… publicación y después ella en otro texto sobre la migración de jóvenes hace alusión también a la… a la situación de las personas migrantes colombianas en el norte de Chile. Te lo voy a compartir.

I: Ya po… muchas gracias.

P: Y… hay que estar atento por que la Margarita parece que va a venir como conferencista a la... la conferencia de… de psicología comunitaria en octubre en la Chile…

I: Una grande que hay, parece ¿no? Una cosa media internacional… si

P: Sí… si, yo no he confirmado con ella, pero me tinca que va a venir y vale la pena igual escucharla…

I: Ya… perfecto. Pucha, súper buenos datos.

P: ¿Ya?

(Termina entrevista, se despiden y se termina conversación en torno a tema de entrevista min 38:33)

SALUD MIGRANTES RECOLETA

I: Investigador E: Entrevistado

- I: Bueno en la entrevista principalmente es para conocer como cuál es la experiencia de trabajo de ustedes como APS y como es el departamento de salud, con la población migrante y específicamente con la población refugiada. Entonces cuáles son desde el departamento de

- E: Sí, mira desde el departamento eh, desde el departamento de salud, el tema migrante es todo planteado desde un inicio de esta gestión como una , como una prioridad, como un tema relevante, desde el 2013. Si bien, eh la cara institucional ,digamos que aborda el tema migrantes, de la municipalidad no, no hace Dideco?, desde salud se plantea como un eje , teniendo en cuenta que en esta comuna, eh había un este , cuando recién esta gestión asumió , había una gran cantidad de , digamos un porcentaje que no era menor , comparado con otras comunas de , de migrantes eh , entonces se plantea más bien como eh como una , desde el acceso ya? [I: desde el acceso] Si, era una de las preocupaciones es decir si los ,eh los extranjeros viven en la comuna de Recoleta, realmente accedian a los al a los , a salud no ? ese fue como la mayor preocupación , por un lado el acceso y por otro lado , el , el eh , entendiendo las barreras , que habían para el acceso, cierto, es decir , porque eh los extranjeros que vienen a la comuna no acudían a el consultorio , Mmm se entiende que también había un problema desde, la información , no cierto ? de si la salud era o no un derecho , si podían acceder o no

- -E: conteste nomas , no hay ningún problema . yo le pongo stop.

- I: Entonces, las preguntas bueno, en estas como que empiezan, desde el 2013

- E:Si

- I: Empiezan con esto de el acceso y las barreras y ,en estas épocas como ven el tema de refugio como o se van o se dejan como se empieza a gestar?

- E: O sea el tema de refugio específicamente no no es un tema que nosotros lo abordemos ponte desde salud[I: Mmhh] ,no nunca fue planteado así [I: Mmhh] era más bien desde la oficina migrantes no cierto? en ese momento la oficina migrantes se Mm trabaja también lo

del ehh lo de la municipalidad sobre todo la mayor preocupación era en dar brindar información sobre el temas migratorios y también me imagino que temas temas re relacionado con refugio no? porque en ese entonces la XX que también era la encargada del estaba en esa condición [I: Mmhh] entonces es esa oficina que quien maneja más el tema [I: claro] desde salud más bien la preocupación estaba centrada en cómo brindar información a los, a los migrantes pa que pudieran acceder a la atención de salud y por otro lado desde eh desde los funcionarios de salud si tenían Mmm ,si conocían la norma las normas existentes en ese momento para poder Mmm para poder atender a la a la población migrante no? [E: claro] porque hasta ese momento lo que había era más bien una Mmm ah una constatación de que Mmm a pesar de que la norma por ejemplo Eh eh permitía la atención a gestantes a niños, a niños de hasta menor ,hasta 18 años se sabía que Mmm que no que se rechazaba que no se atendían sobre todo la las los extranjeros que estaban con pasaporte o que estaban solamente con su documento de identidad entonces a se rechazaba simplemente se les decía que si no se tenía un rut no se les atendía y sin embargo la norma contemplaba en ese momento el derecho a la atención a estos grupos. Entonces trabajamos más bien en ese momento como por, porque la gente los funcionarios conozcan por lo menos cuales eran la la la la norma en relación con la atención .Ahora desde un principio esta gestión se planteó eh la atención a todos los migrantes incluso los migrantes incluso los que estaban en situación irregular SI entonces porque es un derecho y ahí es como se Mmm eso por un lado por otro lado ehh era conocer un poco sus su las razones de los motivos por los cuales no accedía a los centros de atención eso fue como en un inicio y a partir del 2014 se empiezan a plantear algunas medidas para facilitar ponte para facilitar la atención no habían muchos haitianos por ejemplo en ese momento en la comuna pero ya habían algunos, desde el 2014 ya nos planteamos la contratación de una una facilitadora, facilitadora, traductora para la comunidad haitiana en el entendido para las matronas por ejemplo era un poco sobre todo para las matronas, por el tema de las de los embarazos, para manejarla entonces se empiezan a plantear medidas de esa naturaleza más específica para resolver barreras no ? eh mmm nosotros fuimos pilotos desde el 2014 no cierto? y con ehh con con el esos recursos empezamos a direccionar un poco el trabajo darle prioridad al a la difusión en espacios no formales para que la eh la población extranjera pudiera acceder no ? al a la atención de salud y bueno se aprovechaban todos los espacios porque en esta comuna en ese momento se inscribía, o sea se adscribían a los Mmm, a los extranjeros para recibir atención en ese momento todavía no, no había esta normativa de facilidad ,de facilidad de Mmm de inscripción sin, eh digamos sin documento no? pero

- I : Entonces se inscribían más que, más que solo a la comuna

- E: O sea , aquí en Recoleta era la única comuna que incribían con pasaporte entonces eh Mmm el gran problema que teníamos era que podíamos inscribir y dar la atención a nivel de la APS pero no podíamos derivar a la atención especializada [I: claro] entonces desde salud se ha trabajado básicamente el tema desde acceso y de de eliminar un poco disminuir barreras no? pero refugio en particular eh no lo hemos

- I: Por que ,pero ha destacado…

- E: O sea emm no lo hemos trabajado de manera directa en salud pero si hemos participado ponte tu a para algunos eventos algunas acciones que se han hecho como para entender el tema del refugio

- I: Se han hecho capacitaciones en el, el

- E: O sea yo se que XX en alguna oportunidad desde la oficina migrantes han habido algunas actividades [I ; específicamente] a lo largo de estos años no? [I: para para] Claro, como para dar a difu difundir tanto refugio y trata por ejemplo son dos temas que se han [I: si] se han estado eh que se se han hecho algunas acciones no? es que haya sido una línea de trabajo específica no ? más bien desde la oficina migrantes era como pesquisar identificar personas que

- I : Y en estas mmm por ejemplo nose eh el temas de las visa porque igual el tema de las visas de del solicitante refugiado que no ha reconocido su condición mm ta cambiando o sea su sumale 8 meses con uno, como mm 8 meses desde que solicita ,luego, luego se le acaba como el los tiempos de espera son de más que esos 8 meses vuelve con otra visa temporaria de 8 meses asi hasta que tiene respuesta

- E: Si lo que yo haber mira yo personalmente [I: Mm] no conozco más allá de lo que conoce el común de la gente o sea tal vez desde desde esa postura yo lo que puedo decir es tal vez falta falta mayor información sobre sobre el significado del refugio, las razones, las motivaciones, como son los mecanismo más allá ponte tu de la información que uno tiene por extranjería o por cierto por ,página web entonces no no mmm en general cuando ,cuando eh como el común de la gente [I: Mmhh] no cierto? pregunta o le preguntas que que sabe sobre el refugio ,generalmente uno eh asocia el refugio a temas políticos [I: claro] no cierto ? es decir : ah sí es una forma de , Mmm no cierto? es una forma de de facilitarle a algunas personas cuando hay problemas políticos en algun lado. Entonces no hay yo lo que creo en salud. Tu le preguntas a cualquier persona no no hay mayor información sobre eso o sea, la información es insuficiente como para que uno pueda manejar elementos vinculado a temas refugio no ? [I:claro y] Entonces si tu me hablas de los de los plazos yo lo escuchado pero nose exactamente no ?

- I: Y cómo se relaciona la inscripción o si igual aca no hacen distinción

- E: No, no para nosotros no hay ninguna distinción, o sea para cualquier persona que viene en este momento por ejemplo, sin incluso habiendo pasado por Mm por paso no habilitados [I: claro] y si se encuentra incluso sin documentos no no hay eh el refugio no es una en el caso de salud por lo menos en esta comuna mm no es una una limitación para acceder a la

atención de salud. [I: mm ya] en términos generales ah? . ahora lo que yo sí tengo entendido es que hay algunas personas que solicitan refugio y que se le es negado porque porque no como que no acreditan necesariamente que son perseguidos, entran por persecución política por ejemplo no ? eso sí he escuchado unas personas, no como que, que una modalidad ponte para quedarte en un país puede ser el refugio pero no, no, no necesariamente aplicas porque no, no es tan fácil me parece conseguir una no?

- I: Al parecer no, no es tan fácil. Eh y no ha salido por niuna a colación algún caso de salud, la temática de refugio así como en una población que esté solicitando más nose, en servicios de salud mental así por decir algo

- E: Que yo sepa no . Yo por lo menos no conozco, o sea yo he conocido de algunos pocos casos eh vía la oficina ,de migrantes [I : Mmhh claro] cierto que en algún momento nose por ejemplo un colombiano que estaba con otras dificultades y de repente nos dimos cuenta que era desplazado no?

- I: Ah y se percatan ahí

- E: O sea claro un poco en el relato entonces ahí la XX ehh lo derivó para que pudieramos este eh brindar atencion no' pero salvo esos casos puntuales que después no es un tema no ha sido un tema que yo haya escuchado de las referentes o de las personas que trabajan los temas de migración como una como una preocupación, o sea como un tema que uno conversa que uno expresa o que identificas es un poco asi como lo del trata no? [I: Mmhh] en el sentido que todavía es un tema que no, no, no se conversa de manera más amplia no ? [I: Mmhh] por lo menos yo no he percibido no

- I: Y porque asocias esto con algo tan demandante estos

- E: Debe ser porque no son muchos los casos tampoco. Deben ser pocos los casos de eh refugiados en Recoleta. yo no sabría por ejemplo cuántas personas eh desde salud no cierto eeh cuantas tienen refugio, me pongo a pensar ahora nose de repente si alguien va claro y se inscribe en algún consultorio, mmm tampoco no es una pregunta [I: no no] nadie va decir que es un refugiado

- I : Es una con condición que está mientras que es un derecho de la persona a de velar o no develar [E: no, no, claro] ninguna obligación

- E: Claro entonces eh entonces no no aparece [I : no aparece] no aparece

- I: Y cuales creerías que fueran las principales necesidades de los solicitantes así pero especulando, de los solicitantes refugiados

- E: Mira yo , yo creo que en general eh a mi me da la impresión no ? uno de los uno de los temas que yo creo eh es importante para todo aquel migrante o extranjero que migra eeh presionado bueno voluntariamente no? pero presionado por circunstancias diversas ya sea económicas o que son básicamente económicas yo creo que el extrañamiento no cierto ? es el tema de la familia eeh sus procesos de de incert de insertarse de adaptación en un país nuevo con todo lo que eso implica para el proceso para la visa , el trabajo , o el tener poco recursos, o vivir de manera hacinada, todo esos elementos creo que hacen que haya un un algún tipo de dificultad a nivel de su salud emocional mental [I: Mmm] A mi me parece que es ese uno de los temas que no está muy trabajado y que uno se afecta ya ? se afecta bastante , depresión este nose tristeza no? mmm y en el caso de los refugiados yo me imagino que igual porque un refugiado generalmente deja deja familia detrás a veces sale arrancando eeh o algunos incluso con persecu persecución entonces se añaden elementos que son que son mmm mas mm más complejos desde arriesgar la vida también no cierto ? [I: Mmhh] tener temor , sentirse sentirse que probablemente te pueda pasar algo entonces para mi la salud mental para mi a mi me parece que la salud mental es una de las cosa ehh que es aveces mas dificil de llegar porque uno va a a la atención primaria atender generalmente [Mmm] un control de salud, tu examen preventivo o algún problema de mayor urgencia [I: O físico] mas fisico nose pues te duele las muelas entonces puedes eh solicitar pero pero llegar al o sea pedir hora para el psicólogo es como una ruta un poco más difícil y que

- I: Y como es esa ruta en Recoleta como pa entenderla uno tiene que pedir a generales y de ahí te derivan porque

- E: No pero yo te estoy hablando un poco desde desde la cultura [I: ah ok] o sea probablemente no es difícil si tu llegas al consultorio y te inscribes y pides una hora con psicólogo porque tu estas conciente de que [I: de que es lo que te afecta] claro de que estás afectado por eso que tienes tristeza, te pueden dar la orden dentro de un mes o de dos meses por último, uno se puede demorar un poco en eso porque no hay suficientes psicólogos en fin pero eso es porque tu ya estas conciente de que hay ese de que tienes que hacer es proceso

- I: Pero los migrantes no están tan conciente

- E: No y además uno minimiza eso no no [I: Mmm] es decir es como

- I : No es pa tanto

- E : Claro yo o sea yo no me voy a no voy a perder mi tiempo esperando la hora al psicologo y además el psicólogo ,nose pues hay un estigma con la relación a la atención del psicólogo,y el psiquiatra y pasa a segundo plano, generalmente pasa a segundo plano , entonces culturalmente no hay una prioridad o una importancia de que mi salud emocional debo de atenderla , voy por otra urgencia , voy porque me duele estomago porque tengo algún ina bronquitis , alguna afección ehh pulmonar o cualquier otra cosa que sea como más urgente o visible pero si estoy un poco triste o estoy un poco apenado o si no necesariamente y no le voy a dar importancia por eso desde mi perspectiva considero que ese es un tema que eh que uno tiene que mirar con un poquito más de de importancia , tanto para aquellos que son solicitantes de refugio o en general los extranjeros que llegan a un país

- I; So, solicitan, están solicitando menos o sea demandan menos salud mental?

- E: Solicitan menos salud mental, no se les da mayor importancia , eso de manera general

- I: Incluyendo al chileno o hay un contraste?

- E : No yo creo que hay una diferencia cultural en eso , que tiene que ver un poco con con de dónde un viene no? te estoy hablando , yo que soy de peru o sea le puedo dar relativa importancia pero si lo que yo veo de de algunos haitianos no tiene ningún sentido ir al psicólogo o ir al psiquiatra porque todos estos temas los puedes resolver de otra forma, no cierto ? [Mmhh] vas a la iglesia o al culto o a través de la espiritualidad se pueden resolver esos problemas no cierto ? o sea no no se le encuentra mucho sentido , por lo menos algunos expresan que no le encuentran tanto sentido a la atención de un psicólogo o la de un psiquiatra

- I: Claro hay una diferencia cultural. y esto de la población colombiana por ejemplo, cómo se da esto del acercamiento de la población

- E: O sea yo creo que en general en américa latina es, es un poco más común hay una diferencia con los haitianos por la barrera idiomática [I: Mmhh] pero a pesar que cada país tiene su expresión [i: si] cultural , en relación con eso yo creo que en el caso de colombia a mi me da impresión de que que sus vivencias son mucho más Mmm o sea tanta, tantos años de, de, de violencia en Colombia [I: Mmhh] de, de ,de vivir en confrontación en guerra [I: Mmhh] o sea en Perú también han habido varios años pero es distinto, ha sido distinto , distinta la lucha contra el terrorismo , en fin [I: distinto como]. En el caso de colombia son muchos más años . son nose , 30 años creo que de violencia, entonces eeh a habido mucha gente desplazada, la confrontación [I: Mmhh], la la lucha armada que tiene otra [I: otra connotación] otra connotación [I: la violencia] Entonces Mmm [I: eso también se mm] pero de como lo vive la gente en general que viene desde fuera , como digamos por

refugio o por o por cualquier otro motivo ehh viven estos proceso que tienen que ver con el fenómeno este de lo que implica los efectos de la migración , y todo lo que es dejar tu país, dejar tu, tu familia, tus amigos y entonces eso genera procesos

- I: Pero ,pero bueno esta población colombiana quería pesqui, pesquitar , pesquisar como , si esta como violencia finalmente como, como se relacionan ellos con la salud mental, como ha pesqui como ha pesquisado

- E: Es que yo creo que no no [I: no no] no es un tema que se haya trabajado

- I: Ellos no demandan

- E: No, yo creo que en general los extranjeros no, no ,nose de repente XY te puede dar

- I: XY, le vamos a

- E; Precisar el tema

- I : Precisar

- E: Ya, porque el trabaja con salud mental, y seguramente tiene informacion un poco mas sobre cuales son los mayores problemas que se presentan en, en personas que extranjeras que atienden la salud mental, ya ? con él , con él yo creo que puedes, conversar más en detalle

- I: Mmm... Y has sabido algo de sobre las dificultades que enfrentan sobre el proceso de solicitud

- E: Mmm no, no de manera ehh no de manera amplia ah ? yo he conocido un poquito esto a través de algunas personas , que , que , que he conocido y he visto el proceso , no , no soy una persona conocedora de este tema ya ? del tema del refugio. Se que una de las dificultades siempre es el trabajo o sea por lo menos mmm es como insertarse y buscar un trabajo no cierto ? [I : Mmhh] el caso de los profesionales , igual si no, si no, si no válidas el título dependiendo de, de , de la ubicación que tengas no cierto ? entonce ehh claro termina siempre trabajando en en, haciendo tareas que no están relacionadas necesariamente con la profesión no? entonces de las pocas personas que yo he conocido , el trabajo ha sido un tema desde un comienzo [I:] o sea la salud emocional, la familia , más aún ponte si eh si no puedes regresar al país [I: Mmhh] no cierto ? si tienes , entonces esos

elementos son los que, los que creo que juegan no ? pero se que tiene que , el trabajo , el trabajo no cierto ? pasa un proceso de inserción

- I: Y en relación a lo que ellos hacian alla

- E: Claro

- I: Y lo que tienen que hacer acá, y si tienen que salir arrancando

- E: Claro , o sea por ejemplo si eres una médica que esta , que está exiliada aquí , mmm te cuesta no? conseguir fácilmente el trabajo como médica [I: claro , el proceso] no cierto ? vas a trabajar seguramente atendiendo enfermos o atendiendo a personas mayores pero no corresponden ehh o sea el titulo que tu tienes, si en tu país estás trabajando como médico o como médica aquí no , no [I: no es tan rápido] no lo vas a poder hacer tan rápido no ? entonces demora, estos procesos son muy largos no ? [I : Mmhh] llevaban dos , tres años dependiendo de lo que hacías y si ponte si pues si trabajas por ahí por como sociólogo, como antropólogo y si no has validado aqui el titulo, es como , no no difícil no? entonces tienes que hacer tu propio camino [I :Mmhh] es estudiar algo que te pueda , permitir conectar , conectarte, trabajar en.

- I: Y hablando ya un poco más de las comunidades ya , ya no , no volver a pre preguntar sobre refugio , me puedes hablar de estas comunidades que son principalmente son las que mas bien piden refugio que son los colombianos , como ves la inserción de los colombianos , hacen, son como mas sociales , tienen confianza entre ellos

- E: O sea mi percepción, o sea mi percepción, porque claro no, no, no, mi percepción es que ellos logran establecer como , ehh , digamos por la forma de ser también [I: Mmhh] por la idiosincrasia de los colombianos que son , ellos son como mas alegres, mas acogedores, persona que están más acostumbradas a, a que tienen, son más expresivas , les gusta la musica no cierto?, les gusta el baile , entonces yo lo que veo , es que , hay comunidade colombianas que se que se juntan que propician no? [I: Mmhh] los peruanos por ejemplo no, no tanto así , los peruanos somos como un poquito mas , Mmm a ver, nose si la palabra es como más apagado, no apagado porque igual , hay la música y otras cosas, pero en el caso de los colombianos , depende de donde pro procede, pero de los lugares que yo he visto

- I : Sí, porque hay como unos regionalismos, eso es importante

- E: Si en general en américa latina, está presente el regionalismo [I:Mmhh] pero en el caso de los colombianos , es de donde no se pues si es de Cali , si es del Cauca , si es del

Quindio , o sea [I: Antioca, La Gira] son, son mmm eh son personas así como eh acogedoras, alegres [I: y desde que se , ya] entonces eso yo creo que , que ayuda , ayuda a generar comunidad , entre sus propios compatriotas no ? Mmm

- I: Mmm sip y la inserción, que es el tema

- E: Me imaginos que también ayuda un poco en los procesos de inserción

- I : Y los venezolanos que están llegando como a visto este proceso, porque igual ha sido masivo el último tiempo

- E: Si , o sea nosotros , o sea aquí en la la comuna no , han ingresado bastantes pero nunca tanto tampoco , los números son relativos pero la comunidad venezolana es la que ha crecido enormemente en los ultimos ,los ultimos, el último año diría , no los mmm no igual igual yo diría que los venezolanos son , eeh son gente aperrada , como dicen , [I: Que vienen a] si [I: hacer lo que tienen que hacer] de todo [I: independiente de lo que hacian alla] si , si si o sea la sobrevivencia pasa por no importa lo que , son como, lo que yo he visto por ejemplo ellos son como bien conscientes de sus derechos ah, reclaman o sea lo reclaman en el buen sentido de la palabra , es decir, si esto me corresponde yo lo hago [I: demandante] son menos sumiso diría yo

- I: Mmm, que mas poblaciones más andinas quizás ?

- E : Mmm si pues que otras comunidades, no cierto ? son como mucho mas , averiguan, están más informados, es una comunidad también más , por lo menos de los que han llegado aquí, la gran parte son Mmm son gente que ha estudiado, que tiene nivel no? Entonces me imagino que eso tambien influye como para conocer mas tus derechos

- I: Claro , y como ha visto como que son parte , el nivel de participación

- E: Eh no a ese nivel yo creo , o que encuentro

- I: O son más individuos que grupos

- E: Si , a los colombianos yo los encuentro más gregarios no cierto? como más cercanos entre ellos , no , a los venezolanos los veo más de família[I: Mmhh] como de comunidades más chicas [I: claro] o de amigos o de familia Mm así los veo como diferentes pero igual en la misma lógica de, de , pucha a pesar de que estaban, la están pasando mal eh, con ánimo no? alegres [I: Mmhh] eso me impresiona , es como son una comunidad mucho más

Mmm como con una actitud más positiva no cierto? , más optimista eso lo que yo encuentro , menos sumiso

- I: Eeh qué recursos cree que necesita esta institución para mejorar la atención a este tipo de población, volviendo al tema de los refugiados

- E: A qué institución te refieres ?

- I: Ah Recoleta

- E: Ah

- I: Como municipalidad y en el servicio de salud

- E: De repente el primer paso sería cómo conocer, tener más un diagnóstico sobre eso, cuántos refugiados viven en la comuna, no cierto ?, este eeh y o sea , cuantos van en el sentido pa saber pos nose de la cantidad de población, si hay cien ,si hay cincuenta , si hay quinientos , eh de repente poder promover un poquito mas Mm intercambios no cierto ? como pa saber exactamente también como ha sido para como ara refugiados si saber que problemas tienen también

- I: O levantar demanda

- E: Claro, levantar demanda o sea que problemas , que necesidades más que , o sea que problemas pero también qué necesidades no ? o por último compartir un poco historias no ? la gente por ejemplo que ya lleva muchos años aquí , que entró como refugiado, eh tal vez está dispuesto a compartir un poco más la experiencia

- I: La vivencia que son como super

- E: Sí

- I: Son fuerte como pa

- E: o sea no me refiero a que cuente el detalle [I: no] el motivo, la razón sino, a ver , que dificultades como o sea si tu eres refugiado y estás viviendo en este país durante tantos años, cuales serian , las sugerencias , las recomendaciones , en fin , conocer un poco más desde su , desde su perspectiva [I: Mmhh] me entiendes, yo no puedo hablar ,o sea yo

puedo hablar de la experiencia in, in , como se dice, no directa ,sino más bien indirecta [I : indirecta] de lo poco que he escuchado

- I: Si, tan poco , por que son procesos cada ,muy compartido ,pero sí directamente

- E: Mmhh y lo otro , es como la información no? sobre el tema , que yo creo que , que no es , no ,no es un tema que esté debidamente informado , que esté posicionado

- I : Entonces , esta es una pregunta que estoy cambiando. Como no se ha dado una especi, especificidad en cuanto al refugio, cierto, no

- E: Creo que podria hacerse mas, mas cosas no?, no ha sido ,no, no ha sido un tema Mmm , a ver , no es, es un tema relevante no , a ver , tal vez , yo que no conozco mucho , pero yo creo que hay una voluntad política de esta municipalidad ,ya no solamente , con el tema de los latinos por ejemplo, el refugio es un tema que está presente con los otros pueblos , con palestina, con los pueblos que están en guerra , entonces a propósito de eso , estas pocas actividades que se han hecho es como que difunde [I: Mmhh] el derecho al asilo. en fin , todos estos temas que están relacionados con eso , Recoleta en ese sentido es una comuna, es una comuna que acoge, de hecho eh ponte de los eh de los sirios que llegaron algunos sirios que han llegado en algún momento yo se que algunos eh han venido aquí a Recoleta y han tenido algún tipo de , de atención ya , eh o sea es un tema que está presente , como comuna o sea , como alcaldía , como hay voluntad política de que este sea un tema por que el asilo , el asilo , el refugio , es un tema que tiene que ver con, con los derechos humanos , con la democracia, en fin , o sea es un tema que está , que cruza [I: Mmhh] de hecho ponte tu el programa migrante , en la oficina migrantes, eh yo cuando llegué aquí era la oficina migrantes pero yo se que después el nombre se a hecho más visible , por que es la oficina migrantes y refugiados , por que es una línea que está incorporado municipalmente a su trabajo [I: Mmhh] ya, pero yo creo que no ha sido , es insuficiente, porque por ejemplo el conflicto o conflicto de otros pueblos , o situaciones como, como la del pueblo palestino, o sea se han hecho, se han hecho actividades desde la cultura, o sea ha estado presente el tema.

- I: Pero una pregunta como , no han tenido ninguna experiencia ? como intracomunal jaja como con otras comunas que están como macul

- E: Yo por lo menos desde salud no

- I: Desde salud no

- E: Desde salud no , yo desde salud no [I: claro] pero si yo se que hay un espacio ponte hay un espacio de las mesas migrantes, eeh que es como una mesa , es una mesa migrantes donde, donde participan diferentes representantes de las comunas, en las que asiste XX, yo creo que

- I: Ahi como tal vez hay que

- E: O sea, por lo menos conversatorios , o diálogos

- I: Y usualmente migrantes de puros municipios

- E: Si , y tengo entendido que en alguna oportunidad se hace el dia del refugiado por ejemplo [I: Mmm] , se hace actividades , o sea se han hecho alguna cosas ya, solo que yo desde salud , no he participado directamente, no me he involucrado. Más allá ponte tu de la solidaridad , el apoyo, no cierto ? [I: Mmhh] el tema que está más vinculada la conciencia, pero no , no , nose con detalles , de repente se han hecho mucho más actividades y yo estoy desinformada

- I: Claro , volviendo a la salud mental que es un tema , ya como independiente de que si sea o no sean, com ha sido la adherencia de estas distintas poblaciones al respecto

- E: No sabría decirte , no manejo esa información

- I: No , de, de refugiados, sino que

- E: En general

- I: O le pregunto a XY

- E: Sí , yo prefiero por que el conoce un poco mas , nose si sobre ese tema en particular, tal vez eh , yo lo que se por ejemplo es bajo todavía , son bajo , bajo la adherencia de extranjeros, extranjeros eso lo he escuchado pero nose si es real , no podría afirmarte con certeza no? se que cuando se inician procesos, tratamientos ponte tu [Mmhh] de atención psicológica, como que, como que se abandona , se deja tratamiento , son o simplemente van una vez y después ya no

- I: Ya

- E: No hay continuidad

- I : No hay continuidad, y cuando se necesitan por ejemplo a los centros de atención primaria cuando se necesitan como atencion ya más especializada por que me imagino que ya , un adulto migrante que necesita atención, va al psicólogo [E: Mmhh] también hay psiquiatras en?

- E: En el COSAM

- I: En el COSAM

- E: Se deriva al COSAM

- I: Que se deriva al COSAM

- E: Cuando la atención psicológica eh requiere una atención un poco más especializada se deriva al COSAM, porque en el COSAM si hay psiquiatra

- I: Como se llama esa cosa

- E: El COSAM?

- I: Si

- E: Centro Comunitario de Salud Mental

- I: Ah, ya sí

- E: Sí, los COSAM

- I:Si, no, pero no ,no tiene el COSAM de Recoleta ningún nombre en particular

- E: Sí , no

- I: Bueno , ahí lo estaré buscando

- E: COSAM

- I: Y se conectan cuando ya necesitan más ayuda , con los hospitales Horwitz Barak,[E: sipo] vínculo[E: sí, claro que sí] o claro

- E: Si , o sea cuando se requiere hacer una derivación al psiquiatrico, se manda no? se , o sea hay mecanismos de [I: Mmhh] de comunicación entre, entre ellos tengo entendido

- I: Bueno , muchas gracias

- E: Espero haber apoyado en algo

- I: Siempre

- E : Bueno este es un tema que yo, yo no manejo mucho ,no

- I: Pero siempre , siempre todo es informacion , muchas gracias

- E: Ya de que jaja, ya pues

muletillas : [I: claro] [I: claro]

PSICOLOGA FASIC

A: Entrevistadora
B: Entrevistada

A: Entonces nada, era conversar un poquito sobre tu experiencia de trabajo acá en Fasic con población refugiada, primero como cuánto tiempo llevas, qué experiencia tienes con…

B: Bueno, yo estoy trabajando recién este año aquí, desde abril empecé a trabajar y estoy re pocas horas, estoy doce horas, digamos

A: Ya

B: Estoy tres medias jornadas aquí ¿ya? Así que he estado aquí, bueno, obviamente como conociendo el trabajo, conociendo la experiencia, y he estado atendiendo hartas personas igual que están llegando, que solicitan como por demanda espontánea y muchas personas que desde la sección de migraciones piden una evaluación

A: Ya

B: Porque hay dudas como de antecedentes psiquiátricos, ese tipo de cosas. En eso he estado como principalmente. Bueno, y mi trabajo consiste en, como te decía, hacer procesos como de contención, de apoyo en temas generalmente de dificultades de adaptación; también muchas personas, bueno, la mayoría, los colombianos, vienen y traen muchos temas de trauma, mucha violencia que han sufrido, entonces ahí hay muchas tensiones por estrés post traumático y situaciones así como de traumas muy fuertes. Y bueno, y otros temas como de adaptación principalmente ¿ya?, como la sensación de desarraigo, ese tipo de cosas, y he hecho hartas evaluaciones también, como te decía, como que me piden desde la sección

A: Entonces, generalmente las personas llegan, o sea, el primer contacto ¿con quién lo hacen?

B: Generalmente los atiende primero la asistente social

A: Ya

B: Y ahí me los derivan pa' acá cuando ven que están como muy mal y todo, y otras personas piden directamente ahí en recepción atención con psicóloga

A: Con psicóloga

B: Y también, bueno, he coordinado estos talleres, como lo que te decía, sobre trata, sobre violencia de género. Estamos también, ahora incorporamos unas clases de yoga para las personas, para los refugiados, y después de las clases siempre se da una conversación, salen hartos temas personales y todo. Así que eso es semanalmente

A: Sí, si me comentaron… una señora venezolana me comentó, sí

B: Claro. Eso, no sé qué…

A: Y específicamente, en el fondo, ¿cuánta...?, el tema de la atención psicológica es uno de los, digamos, servicios que da Fasic

B: Claro

A: ¿Tú eres la única que está o hay más personas que están con eso? Cuántas horas, digamos, le pueden dedicar a la atención psicológica

B: O sea, ahora estoy yo po, antes había una psicóloga, no sé si tenía más horas, creo que estaba más o menos en el mismo horario

A: Ya

B: Así que estoy yo sola, básicamente, en la parte de salud mental, claro

A: Ya

B: Sí. Lo otro es como el apoyo desde lo social

A: Ah, okey. Ya, porque me habían hablado otras personas, pero como que me decían "el otro psicólogo", pero yo no sabía si habían más psicólogos

B: No, claro, es que a veces se confunden…

A: Sí

B: Claro, están los asistentes sociales y la Pamela que es la encargada de coordinar como temas laborales

A: Ya

B: Buscar posibilidades de trabajo, orientar para que se inscriban en las OMIL y ese tipo de cosas

A: Claro, ya, ver los temas más como de inserción

B: Claro

A: Integración, okey. Porque, en el fondo, la opción de atención psicológica la tienen principalmente acá, ¿o también tú ves qué buscan o usan servicios de atención de salud mental afuera?

B: Ah, bueno, claro. Es que eso es, generalmente, es parte de la orientación que se hace porque no tienen, por ejemplo, información de la inscripción en los consultorios y eso, entonces cuando yo veo que necesitan una atención más permanente y como más estable, a lo mejor, en el tiempo, más accesible para ellos, también les hago una derivación a los consultorios para que puedan ser…

A: Ah, tú los derivas

B: Atendidos por psicólogos, claro. O cuando necesitan, hemos coordinado un par de atenciones psiquiátricas… es difícil igual porque hay muy pocos psiquiatras que puedan atender, o sea, digamos, que tengan valores accesibles para lo que se pueda pagar desde aquí ¿ya? Pero se ha apoyado en algunas atenciones psiquiátricas. Y si no, yo también les sugiero que pidan en sus consultorios

A: Ya, y de ahí de los consultorios los deberían derivar a COSAM en caso de tener alguna necesidad permanente de terapia

B: Claro, sí

A: ¿Y tú has visto que eso se realiza?, porque en la investigación hemos estado como visibilizando que, en el fondo, en el sector salud está bien invisibilizado el tema de refugio

B: Sí… eh, mira, yo no he tenido muy buena experiencia. Por lo que me relata la gente, han tenido hartas dificultades en los consultorios para las inscripciones, de hecho, atención psiquiátrica, les dan hora como pa' tres meses después. Entonces, cuando están en crisis y necesitan urgente, obviamente hay que movilizarse como rápido. Bueno, esas son las falencias propias del sistema de salud yo creo, pero sí muchas personas han sufrido mucha discriminación, así como un trato bien malo en los consultorios. La mayoría, hay otros que tienen buenas experiencias; una señora venezolana que tuvo un problema al corazón y ha estado súper contenta con la atención que recibió y todo. Pero, en general, por lo menos los relatos que he recibido yo no han sido muy positivos. Entonces, quedan como ahí medios a la deriva, por lo menos en las atenciones de lo que es salud mental

A: En salud mental específicamente

B: Claro

A: Porque también en salud en general, son considerados como migrantes más no más

B: Sí, claro

A: Están en la misma… no hay una visibilización de su condición de refugiado

B: Claro po, y hay poca información sobre el tema de refugio. Entonces, nosotros aquí les entregamos un certificado que dice que son solicitantes de refugio y que, en el fondo, tienen derecho a acceder a la salud, a la educación, al trabajo igual que cualquier chileno ¿ya? Porque es una condición especial de migración

A: Claro

B: Pero la gente como que no, desconoce eso en realidad. Y desconoce las leyes, los derechos que puedan tener los solicitantes de refugio o refugiados

A: Entonces eso se está transformando como en una barrera específica también pa' salud mental

B: Sí po, sí

A: Y en el tema de la atención, como de la especificidad de sus necesidades, o sea, como situaciones de violencia o estrés postraumático ¿cómo se trabaja en el sector público esos temas?

B: En el sector público yo creo que se trabaja como con el estrés postraumático más específicamente, pero desconozco en realidad cómo puedan abordar el… así como en los COSAM esos temas. No sé si hay una especialización, digamos, como en temas tan concretos como los que traen, que son temas bien específicos ¿me entiendes? Bueno, lo otro que nosotros hacemos, eso se me olvidó comentarte, que en los casos de personas que han sufrido violencia sexual los derivamos a la Fundación León Bloy, que tienen especialistas en eso

A: Ah, okey, ¿de qué se trata esa fundación?, no la conozco

B: Es una fundación que trabaja justamente con temas de abuso sexual, claro, temas de violencia sexual en general. La mayoría son mujeres, pero también hay hombres, y ahí hemos derivado y han recibido su proceso de terapia específicamente en esos temas ¿cachay?, entonces ahí se van como en paralelo, los voy, vienen para acá, se hace un seguimiento y se va como conteniendo, digamos, en eso. Pero el tema específico de lo sexual se aborda ahí, se trabaja ahí

A: Se aborda desde ahí

B: Claro

A: Por lo menos ahí se gesta, está ese espacio como para… hacer una (no se entiende 07:33) específica, digamos, de la migración

B: Sí

A: ¿Y tú, en cambio, habías tenido trabajo antes como con refugiados, con migraciones como desde la psicología?

B: Anteriormente no, o sea, he trabajado con niños y jóvenes en colegios que son migrantes ¿ya?, en trabajos grupales, talleres, talleres de identidad y todo. Pero así específicamente en atención directa de casos, no. Así que ha sido una experiencia bien buena de aprendizaje y de conocer más en profundidad todo lo que traen porque uno también desconoce muchas veces cómo… o sea, uno tiene nociones, qué se yo, porque se informa

A: Claro

B: Pero es distinto estar trabajando, digamos, con la gente

A: ¿Y has sentido la necesidad de instrumentos específicos para trabajar con esta población?

B: Sí, igual he sentido… bueno, yo como que por mi cuenta he estado leyendo, informándome más, pero sí yo creo que es necesario como, a lo mejor buscar alguna especialización en los temas porque son temas distintos a los que traen otra gente. Como te

decía, el tema del desarraigo, el tema de la identidad sí, que llegan aquí y empiezan a surgir fuertes las crisis de identidad, la discriminación que sufren. Y todo eso se mezcla además cuando hay, además, porque como son refugiados obviamente traen temas de las salidas de sus países que son muy bruscas, muy rápidas, producto de una emergencia que tuvieron que salir, dejar todo y salir… la soledad, porque salen muchas veces solos, dejando a la familia. Entonces, son temas como bien específicos

A: Claro, y que a veces no da como los instrumentos más estándar como para poder tratarlos

B: Claro. O técnicas más que instrumentos. Quizás como conocer más de técnicas específicas para abordar eso. Yo a partir de como mi experiencia y de lo que he leído he podido como entender y trabajar el tema de la inserción, los códigos también, orientar un poco como el lenguaje, la forma de ser de las personas. Porque para muchos extranjeros como que ven a los chilenos como medios prepotentes, como muy fríos, ya las formas de relacionarse con muy distintas. Son temas que se han trabajado harto

A: ¿Cuáles son los temas, a propósito de eso, qué es lo que más emerge como las primeras demandas como de parte de los refugiados? ¿Cuáles son sus temas como más urgentes?

B: Claro… bueno, lo más urgente es, bueno, lo que te decía. Las sensaciones de soledad, los sentimientos de soledad, de desarraigo, de sentirse como totalmente desorientado y desadaptado en una sociedad que es distinta, que desde el discurso como que los acoge y todo, pero en la concreta, en la vida cotidiana, sufren un montón de discriminación ¿sí?, y rechazo, e historias así de maltrato…

A: ¿En qué lo perciben, por ejemplo?

B: O sea, por ejemplo, el racismo con los negros es *heavy* ¿cachay?; que les dicen cosas, que les gritan cosas, que las instituciones también los discriminan ¿ya?, que los mandan pa' allá y pa' acá y bueno, que obviamente hay mucha burocracia, pero también como hay desconocimiento, muchas veces como que dicen: "No, usted no tiene derecho a inscribirse en Fonasa. No tiene derecho a esto", y se les trata mal. O sea, y yo creo que no dudo de que efectivamente sea así, que hay funcionarios que son muy discriminadores, y que les dicen: "Ya, mejor ándate a tu país, negro" y "¿Pa' qué vienes aquí a quitar los trabajos?", qué se yo, o: "¿Pa' qué vienen aquí a enfermarse? ¿o vienen aquí a tener hijos", qué se yo. Comentarios como esos que me han comentado que reciben

A: Qué fuerte

B: Sí po. Y bueno, el tema de la desadaptación, conocer como los sistemas de los colegios, de los estudios… y los traumas po. No sé, como las manifestaciones de los estrés post traumáticos, que no duermen, que hay muchas pesadillas, que hay muchas personas que están muy paranoicas, que sienten que están aquí y que todavía los siguen y eso. Bueno, que son los síntomas propios del estrés, como de sentirse perseguido, sentirse amenazados también

A: ¿Y tú sientes que con los instrumentos que tienes o con el tiempo que tienes con, digamos, recursos que tienes acá, puedes generar, digamos, una incidencia, una contención, un apoyo a las personas que encuentras? ¿Qué sensación tienes de ese trabajo?

B: Bueno, con las personas que he trabajado hasta ahora sí, yo creo que he logrado contenerlos, porque finalmente es eso po; se hace mucha contención, escucharlos, como están la mayoría de las veces las personas muy solas, el venir a desahogarse y a conversar con alguien cercano, digamos, que lo sienta cercano ya es un gran alivio para ellos

A: Claro

B: Ahora sí, igual yo sí he sentido como que se me hace poco el tiempo de repente, porque podría como… o sea, es más difícil poder desarrollar un proceso terapéutico más en profundidad ¿sí?, entonces, generalmente se hacen algunas atenciones seguidas, después ya empiezan a conseguir trabajo, los horarios entonces como que ya son más difíciles para ellos y todo, pero sí…

A: Entonces como que dejan de venir espontáneamente

B: Claro. Entonces, ya después se hace un seguimiento una vez al mes o qué se yo, pero hacer un proceso, una terapia como propiamente tal es más difícil, han sido los menos los casos con los que lo he podido hacer, así de semana a semana y todo

A: ¿Y casos más agudos?, como me decías que hubo derivaciones psiquiátricas, ¿qué pasa ahí cuando, en el fondo, necesitan una intervención más a largo plazo o, en el fondo, ya no es contenible con estos encuentros semanales, digamos, con la persona?

B: Claro, bueno, ahí hemos mandado, hemos hecho la coordinación con psiquiatra y desde aquí se les paga dos o tres atenciones

A: Con psiquiatras privados

B: Claro, claro. Y claro, a ellos se les da hora, no es atención semanal po, como dos veces al mes si es que, una vez al mes en general, y después se va controlando el tema de los medicamentos, cuánto les da. Y, como te decía, en paralelo yo voy haciendo una contención más como terapéutica

A: Claro

B: ¿Entiendes? Y cuando ya requieren algo como más estable, ya se tendría que derivar a COSAM

A: Claro, para que estén ahí…

B: Claro, sí

A: Te iba a preguntar en torno al proceso de refugio, ¿cómo ves tú que se está realizando en Chile? Cuántas facilidades, cómo se están cumpliendo las leyes, cuántas dificultades u obstáculos se están poniendo, de las experiencias con las que tú estás trabajando

B: Claro. Bueno, es un proceso bien difícil, más ahora como con este gobierno que han cambiado las leyes y todo, y hay muchas más limitaciones para aprobar la solicitud de refugio, lo que es… bueno, lo hemos visto aquí en el programa y también con las personas que vienen, que se están aprobando mucho menos solicitudes. Sí se le está dando como cierta prioridad a los venezolanos, que parece que se va a implementar como todo un programa especial y un plan de contingencia para acogerlos, pero…

A: Pero en calidad de refugio, no por lo de la visa democrática

B: Claro

A: Es la gente que está en solicitud de refugio

B: Sí, los solicitantes de refugio. Y ellos tienen un trato distinto en general, eso como que se está viendo que también tiene que ver con las políticas de gobierno ¿sí? Pero, en general, para personas de otros países, bueno, hoy día estamos viendo cómo se están llevando a un montón de haitianos y todo que dicen que es… ¿cómo es que le pusieron?

A: Sí, como retorno… democrático

B: Democrático, sí… como que están… los nombres que usan, bueno…

A: Sí, el eufemismo

B: Sí. Entonces, yo creo que hoy en día, hasta donde yo lo percibo hay mucha más dificultad y más trancas, digamos, para los solicitantes de refugio. Mucho más trabas, digamos, para poder ser refugiado. Entonces, se promueve que finalmente desistan de la solicitud y que sea como un migrante cualquiera, o que retornen a sus países, lo que finalmente termina… esas son como las opciones

A: ¿Y la gente renuncia a la solicitud de refugio o…? porque yo en las conversaciones que tuve, me planteaban esa duda. O sea, como, me han dicho que se puede demorar tanto este proceso que pensaban en (no se entiende 16:11)

B: Claro, , es que es muy demoroso, o sea, están esperando a veces tres, cuatro años, hasta cinco años hay algunas personas que están esperando. Y claro, a veces como que, justamente lo que te decía, que hay tanto desconocimiento de lo que implica el tema del refugio que finalmente optan por renunciar. Yo creo que igual son menos los casos los que renuncian a la solicitud de refugio, hasta donde yo he visto ahora el poco tiempo que llevo. Pero yo creo que se ha dado en algunos casos, y también yo he sabido de algunas personas que, finalmente, han retornado, porque les ha costado tanto aquí conseguir trabajo, insertarse y adaptarse; personas que, a lo mejor, tienen más vulnerabilidad que finalmente optan por retornar al país

A: A su propio país

B: Claro, claro. Y buscan opciones ahí en sus países, o buscan otros países ahí después para… para solicitar refugio

A: Entonces, en general ¿cómo ves tú que están actuando las instituciones en torno al refugio?

B: En torno al refugio… bueno, yo creo que, como te decía, como hay desconocimiento, finalmente se les trata igual que como cualquier migrante ¿sí? Yo creo que en muy pocas instituciones está la información de qué implica el ser refugiado, porque no entiende, no está la noción de que si es refugiado tuvo una situación de urgencia en su país por la que tuvo que salir, sino que en general para la gente y para las instituciones son migrantes cualquiera no más ¿cachay? Entonces no hay un trato distinto, no hay como una… bueno, eso, no hay un trato

A: Claro. Porque parecía, este tema lo conversamos también, por ejemplo, en los municipios, Municipalidad de Recoleta, de Quilicura, que tienen oficinas de migrantes y refugiados, y sin embargo, claro, nos decían que efectivamente pa' ellos era complicado porque veían que, a veces, la misma gente no quiere visibilizar su condición de refugio, como que existe un poco de vergüenza, un poco, un sentir un poco… entonces como que tampoco creían que el visibilizar o, en el fondo, que las personas supieran que por un rut o por, no sé, alguna marca en la solicitud o en los papeles se supieran que eran refugiados, tuvieran que tener un trato distinto

B: No sé si será tan así eso ah

A: Eso te quería preguntar, ¿cómo lo ves tú?

B: Porque, por lo menos, en mi experiencia la gente tiene muy clara su solicitud y que son refugiados y por las razones que están solicitando eso. Y, en ese sentido, nosotros también tratamos de empoderarlos en que ellos sepan sus derechos y que también puedan exigir sus derechos ¿sí?, sus derechos a los accesos de servicios, acceso a la salud, acceso a la educación y todo… entonces, justamente hay que empoderarlos y es lo que también, uno de los temas que abordo en que ellos sepan que son refugiados, que tienen derecho ¿sí?, que tienen derecho también a la protección por el estado chileno porque por algo están solicitando refugio ¿sí?

A: O sea, como mirarlo, instalarlo en las personas como desde la perspectiva de derecho

B: Claro

A: Porque claro, en el fondo, en los servicios, toda esa dimensión no existe. O sea, sobre todo en los servicios de salud que es donde estamos mirando

B: Sí. No po, para nada, no existe eso. Y no existe para nadie, digamos; para los migrantes tampoco existe, para los pobres tampoco

A: Claro

B: Sí

A: Y la decisión de solicitar refugio… la hacen las personas, las personas llegan informadas a pedir refugio, o en el fondo es una contingencia que pasa… en el fondo, ¿cómo se orienta la solicitud de refugio?, el primer contacto, el darse cuenta que existe.

Porque algunos testimonios también nos decían: "yo no sabía que existía el refugio hasta que una amiga me habló"

B: Claro, claro. En general, la gente maneja algo de información como, claro, por amigos, por temas…

A: Por redes informales

B: Por redes, claro. Y aquí se les entrega más información de por qué, en qué situaciones podría ser solicitante de refugio y no ¿sí?, que sepan también como dar a conocer los hechos por los que podrían ser solicitantes de refugio, porque… pero en general la gente tiene nociones, saben que tienen que pedir refugio a frontera, o llegando tiene que ir a la sección de refugio y todo, tienen algunas nociones

A: Ya

B: Y porque además la gente que llega acá han vivido situaciones que son terribles po y que tienen que salir de su país o sino pone en riesgo su vida. Eso igual yo creo que les da una noción ya de que necesitan pedir protección ¿sí?

A: ¿Y qué pasa con contextos específicos, como por ejemplo, Colombia, donde el tema de la violencia está tan normalizado? ¿Cómo se diferencia, digamos, entre una persona que puede solicitar refugio por una situación de persecución específica y, en el fondo, un colombiano que ha normalizado también las múltiples formas de violencia que están en Colombia?

B: Claro, pero a ver, en qué sentido, a ver como…

A: No, entendiendo que, en el fondo, también como he conversado con, sobretodo también personal de salud ya como de autoridad o de las corporaciones, por ejemplo, me dicen: "bueno, pero en el fondo las situaciones de violencia o las situaciones de persecución están en todas partes", o sea, que te marquen la casa y te van a robar, o que la familia tal te agarró mala. Por ejemplo, esto era en una comuna, en Macul que me decían que, en el fondo, había rencillas en las poblaciones más complicadas, decían claro, entonces, la situación de ser perseguido por algo no es… o sea, puede ser normalizada en contextos de violencia, pobreza y como una situación más estructural para todos. Entonces, en el fondo, ¿cómo diferenciar la condición de refugio de esta condición como de violencia generalizada?

B: Ah, claro. Sí, es que yo creo que en el caso de los colombianos es, claro, ellos han vivido en una situación de guerra, y las situaciones de violencia y tortura y matanza y todo yo creo que son muy extremas, o sea, una cosa es la violencia porque sabemos que la sociedad es mucho más violenta y todo, o cosas que pasan en Venezuela, no sé, o en otros países, pero lo que ocurre en Colombia, los testimonios que yo he escuchado son terribles, o sea… es una guerra po ¿cachay? Entonces hay una guerra que, en el fondo, yo creo que pa'l resto de Latinoamérica y del mundo es una información que no se maneja, que ha estado muy oculta, entonces yo creo que… por lo menos a mí me ha pasado de tomar conciencia, porque no es

que se hable de la paz en Colombia, que no existe eso, o sea, y es una guerra terrible donde la violencia es extrema, extrema. Hay una matanza, hay persecución, hay tortura, hay violencia sexual para niños, para jóvenes. Es como muy extremo. Entonces, yo creo que no se puede hablar como se una violencia más como en el resto del mundo, no sé si me explico

A: Sí

B: ¿Cachay?, porque es mucho más extremo, es terrible. Y viven en ese contexto hace muchos años, o sea, décadas de años que está sucediendo eso y ha estado muy oculto, o sea, manejado así de una manera muy solapada en cuanto a los medios informativos, entonces…

A: Sí, porque eso es como el elemento clave en el sentido de que como no se conoce, en el fondo, uno piensa en Colombia, en un gran país con conflictos internos, pero no puede, no pasa realmente imaginarse qué dimensiones tienen esos conflictos

B: Claro po, y uno… y aparece el país como este país turístico, que ahora ya quedó todo atrás, qué se yo, y va a quedar cierta noción de que hubo algo malo, digamos, de que el tema de narcos y qué se yo, pero ahí lo que hubo fue una guerra. O sea, el pueblo estaba perseguido, el pueblo estaba atemorizado; se les quitaron sus negocios, sus tierras, sus casas, todo. Ha sido terrible po, ha sido muy extremo

A: Y eso, en el fondo, tú lo has ido recuperando ahora que tienes contacto con personas que han estado en eso

B: Claro, claro. Con la gente que estaba atendiendo, que hay testimonios terribles y cosas que suceden hasta el día de hoy po ¿sí?, esto de reclutar a niños como soldados, que allanan las casas, que violan a las mujeres, que matan y torturan a los hombres, todo. O sea, está pasando hasta el día de hoy, yo creo que a lo mejor ha disminuido mínimamente en algo, pero por algo siguen llegando colombianos

A: Pero la gente viene con esa experiencia

B: Sí, y siguen solicitando refugio, y siguen asustados y siguen atemorizados, gente que llega para acá por las familias que quedaron allá, mujeres que han hecho denuncias de las situaciones y que les han matado a toda su familia ¿cachay?, o sea, terrible

A: Claro porque, en el fondo, el nivel de desinformación respecto a esto es súper alto

B: Sí po

A: O sea, en el fondo, como que se habla de los colombianos, digamos, como una gran masa de personas que están viniendo y sin ninguna noción realmente de las diferencias también locales que tienen y todo, en el fondo, los orígenes de toda esta migración tan masiva

B: Sí. Claro po, o sea, por algo siguen llegando personas colombianas y solicitando refugio

A: ¿Siguen siendo los colombianos y venezolanos como los más numerosos en…?

B: Sí, sí

A: ¿Lo han seguido viendo?, porque colombianos fue como una constante los últimos años ¿no?

B: Sí. Bueno, ahora último los venezolanos ya mucho más. Pero sí, son los dos países como con más, que llegan más personas

A: ¿Y en el proceso de solicitud tú les das también apoyo como para esta…?, en el fondo, como para construir, reconstruir su historia, para que sea una historia que sea, digamos, plausible de que le den el refugio

B: Sí, claro. Bueno, eso, se les entrega harta orientación también con los asistentes sociales

A: Ya

B; Pero sí yo también, al indagar de las razones de por qué salieron y todo, que sepan fundamentarlo y presentarlo bien y con pruebas que siempre se pide, como recortes de diarios tipo, no sé, diferentes pruebas que puedan haber. Pero claro po, que puedan reconstruir, digamos, todas sus vivencias para que justifiquen, digamos, la solicitud de refugio

A: Y ahí, por ejemplo, ¿qué indicaciones les das? ¿Cuáles son las formas, digamos, de ayudarlos a construir, digamos, una historia que sea candidateable? No sé cómo decirlo

B: Claro. Más que nada ayudar a ordenar el relato ¿sí? Hay gente que tiene todo muy claro, muy lineal, pero hay otras personas, lo que tú decías, que está normalizado, entonces como que no dimensionan que lo que les pasó realmente es grave, es algo que puso en riesgo tu vida y todo, y por lo que mereces, digamos, tener protección por un Estado ¿sí? Entonces, más que nada es orientar, o sea, ordenar el relato, enfatizar en las situaciones, que le tomen el peso, digamos, que tomen conciencia de la gravedad como de lo que han vivido ¿sí? Y ordenar la información que tengan, no sé, porque a veces tienen recortes de diario o programas, qué se yo, las denuncias que han hecho, como la documentación que esté ordenada, que esté clara

A: ¿Y llegan con esos documentos, llegan con esas pruebas? Digamos, porque ese es un tema también en la solicitud, el probar tu condición de víctima

B: Claro. Sí, en general hay muchas personas que llegan con hartos documentos, sí

A: Ya. O sea, no es tan difícil la construcción, como la reconstrucción de la historia

B: No. Y claro po, bueno, ahí lo que se enfatiza también es que ellos puedan explicar y expresar claramente que si ellos regresan a su país su vida corre riesgo

A: Claro

B: ¿Ya? Y que su familia también está en peligro ¿cachay?, que esa es una de las condiciones, digamos, para el refugio

A: ¿Y qué pasa ahí con la gente, por ejemplo, que piensa en retornar?, como que se desestabiliza esta idea de que pueden poner en riesgo sus vidas

B: Sí po. Claro, es que ahí se les confronta un poco porque, en el fondo, dice: "¿Cómo estás solicitando refugio si piensas en retornar?", claro, esos han sido los menos casos y casos extremos en que ya no logran adaptarse y no logran, digamos, insertarse en ninguna red, ni trabajo, ni estudio, y que están solos… claro, se confrontan a esta idea de regresar, y se entiende igual porque también el desarraigo, la desadaptación ¿cachay?, es parte de los procesos, y como el extrañar su lugar, su país po. Sus orígenes

A: O sea, se confronta esta dimensión más como racional de saber qué es lo que está pasando

B: Claro, claro. Sí, como bajar la ansiedad y como: "piénsalo bien, evalúa los riesgos, evalúa los pro y los contra"

A: Y esta cosa muy afectiva de sentir que no podí más y que necesitay…

B: Claro, entonces ahí se hace la contención para que también evalúen y vean los pro y los contra de tomar una decisión, claro

A: ¿Te ha tocado estar como dentro de un proceso de solicitud? O sea, como acompañarlos desde más allá, como ir a las entrevistas o…

B: No, no. Sí me ha tocado como acompañar el proceso, bueno, desde acá, cuando me dicen: "bueno, mañana tengo la entrevista" y qué se yo, entonces trabajando para que no se pongan nerviosos, para que puedan estar tranquilos, para que puedan expresarse con claridad y todo, no enredarse con esto que, a veces, como que la ansiedad se los come y…

A: ¿Y cómo son las entrevistas? ¿Te las cuentan o te las relatan como pa' ellos…?, son entrevistas donde, no sé, les piden que les recuenten la historia de nuevo

B: Claro, claro. Algunas personas han dicho que, pucha, que les preguntan como varias veces lo mismo y que ya no quieren volver a hablar de eso porque son situaciones súper duras para ellos y todo. Pero, en general, parece que los atienden… no sé si es psicólogo o qué profesión, pero en general son personas como, por lo menos lo que han contado, como bastante amables y que se sienten contenidos también

A: Ya

B: Sí

A: No hay como una revictimización en ese proceso

B: O sea yo creo que igual se da, en esto de tener que relatar más de una vez todo lo que han vivido y todo. Sí, es inevitable que se dé. Por lo menos, en ese sentido, aquí conmigo, bueno, ellos toman, ven como hasta dónde quieren llegar con sus relatos y todo

A: Claro. Y ustedes como Fasic ¿son interpelados como por el ministerio o por extranjería? En el fondo, cuando se trata de temas de refugio, ¿hay una consulta, hay reuniones con ustedes o…?

B: Sí, hay una relación permanente. Bueno, con Elizabeth, ella está en permanente comunicación con las personas de la sección. Y sí, se trabaja bastante en conjunto, como ellos nos mandan personas como con indicaciones especificas en cuanto a solicitudes de trabajo, solicitud de atención…

A: Desde la dirección de extranjería

B: Claro, u orientación en otras cosas, qué se yo. O personas cuando tienen dudas, por ejemplo, en temas psiquiátricos, que ha habido un par de casos de personas con, no sé po, esquizofrenia y todo, claro, lo mandan así conmigo para que yo pueda formar una opinión de, no sé, esta persona está fuera de la realidad, es otra la atención que necesita. Sí, pero se trabaja muy en conjunto, digamos, con bastante retroalimentaciones con los casos, sí

A: Ah, mira. Porque, en el fondo, Fasic es como la única institución más autorizada, con una experiencia larga en temas de refugio, entonces, como las políticas de refugio, si los consideran o no a ustedes ¿no?, como actores…

B: Sí, sí po. Se trabaja muy a la mano

A: Bueno, eso más o menos. En temas de salud, así solamente como más específico, ¿qué temas has visto más amplio?, más que salud mental en general. En salud, ¿Cómo cambian sus condiciones de salud o no cambian? Cuando, respecto a lo que relatan, qué pasa con enfermedades que pudieron tener antes, después, o temas de salud que se vayan produciendo después del proceso

B: Sí, yo creo que hay temas que… bueno, como que las defensas ahí obviamente como que disminuyen por los temas emocionales, por una parte; por otra parte, el clima se les hace muy difícil. Si en invierno lo han vivido, la mayoría de las personas que yo he visto aquí, como muy difícil, muy duro, se les ha hecho aquí… hicimos, les juntamos ropa, abrigo y todo, porque además…

A: Para el invierno

B: Claro, porque andan súper desabrigados, las enfermedades como respiratorias y ese tipo de cosas son muy frecuentes. En invierno andaban todos enfermos aquí, en ese sentido. Entonces, la alimentación y el clima como que les afecta mucho, les incide en su salud como física ¿cachay?

A: ¿Y condiciones de vida? Vivienda, trabajo, educación, ¿cómo se activa o cómo se insertan todos esos procesos, o sea, en todos esos temas, cómo los van resolviendo?

B: Bueno, desde aquí ahora se les está apoyando a muchas personas con arriendo ¿sí?, y se les orienta como un poco en la búsqueda, qué sectores buscar. Bueno, hay muchos que viven casi toda la familia en una habitación, en condiciones bien precarias de vivienda. Así que se orienta, también se orienta en temas, que tengan ojo con los arrendatarios, con los arrendadores, digamos, que siempre tienden a abusar, a cobrar precios como excesivos y todo, entonces se entrega harta orientación en eso. Pero, en general, las viviendas y los

lugares donde viven y donde consiguen arrendar a precios más o menos razonables son bien precarios

A: Ya

B: Sí, como espacios mínimos compartidos y todo

A: ¿Y ahí aparece este tema como de las descalificaciones que produce la migración? O sea, el hecho de que se insertan en condiciones de pobreza o de clase social mucho más baja de la que estaban acostumbrados, ¿has visto eso?

B: Claro, sí. Claro po, y muchas personas, hay muchos profesionales y todo que están trabajando haciendo aseo o en trabajos así como de oficios y cuesta adaptarse como a esta nueva realidad. Y claro, es lo que tú dices, de cambiar de estatus social, digamos, de forma de vida, es duro igual. Porque llegan a una situación de pobreza efectivamente. Y ahí se contiene, bueno, de que es parte del proceso, que es parte de la adaptación, de la inserción, que lo vean como algo temporal, que ya va a haber oportunidades de ir surgiendo y qué se yo po. Pero son procesos duros para las personas, y suman ahí dificultades también en lo emocional y todo po

A: Claro

B: Estar viviendo en condiciones así como bien precarias

A: Sí. Conversando también con dos personas venezolanas, ¿te acuerdas, la mamá y el hijo?

B: Ah, sí

A: Y claro po, ellos tenían ese tema, como que estaban, habían bajado de clase, que nunca se habían imaginado perder tanto, en el fondo, de seguridades que tenían

B: Claro po, porque estuvieron viviendo de allegados, y ellos personas profesionales, con muy buen pasar allá en Venezuela y todo

A: Claro

B: A estar así, viviendo como de prestado y todo. Ahora ellos arrendaron un departamentito y claro, entonces ellos me decían: “Pucha, es como volver a empezar” y todo de cero, y estar como… ellos llevan casi un año, más de un año aquí, y era como esa sensación de tener que estar todo el tiempo partiendo de cero ¿sí? Partían de cero y cuando llegaban y… y varias veces partiendo de cero. Eso es súper desgastante po. Es como agotador

A: Claro. Y las condiciones de pobreza que pone Chile para las personas que están en migración, o sea, como vivir en una pobreza que igual implica también todas estas cosas de, condiciones de… no sé, no alcanza para calefaccionarse, o las poblaciones que son una realidad distinta a la que pueden, a las poblaciones que pudieron haber venido, quizás venían de sectores rurales o, en el fondo, donde no estaban estas condiciones de hacinamiento

B: Sí pues, y la violencia que se ha dado en las poblaciones y todo. Y es como que dicen: "Claro, me fui de Colombia donde estaba viviendo algo terrible y llego…" claro, decían "no es tan terrible como allá", pero igual han…

A: O les da miedo salir a la calle

B: Claro po, salir en la noche, que hay balaceras, cosas así que es complicado. O sea, en el fondo, eso también es parte de la revictimización ¿cachay?

A: Claro, en el fondo, nosotros queremos un poco como relevar esta idea de que las condiciones que pone Chile, en el fondo, aumentan la vulnerabilidad de estas personas

B: Sí, totalmente

A: También en términos de salud, o sea, el hecho que no está, se logró que tuvieran acceso, tienen todo en términos legales, una legislación que debería proteger, pero, en el fondo, en la práctica estamos viendo que no lo hacen

B: Sí po. O sea, es que en la práctica no, es muy… no se da po, no se da el acceso a los servicios tan fluidamente como se plantea ahí, digamos, en el papel

A: Claro, sí. No, y de hecho, ya todas las restricciones que se están poniendo en el mismo refugio parece que, en el fondo, están poniendo como una salida más difícil en la posibilidad de, efectivamente, de tener refugio

B: Exactamente

A: ¿Y en temas laborales? ¿Ellos logran como…?

B: Eso también es todo un tema porque bueno, a la mayoría les piden la…

A: La definitiva

B: Definitiva, claro, la permanencia definitiva, y nadie lo tiene po, entonces… y aunque se les entrega este certificado que dice que es refugiado, que tiene que tener derecho a poder trabajar como cualquier chileno y todo, que es una condición distinta a cualquier migrante, de todas formas no se cumple, la gente pide la definitiva y les cuesta un montón conseguir trabajo así como formal. Entonces, terminan muchas veces vendiendo cosas en la calle, o negocios así como más informales, o haciendo aseo, qué se yo, trabajos, oficios informales. Son muy poca la gente que logra un trabajo con contrato, así como con todos los derechos y condiciones laborales mínimas…

A: ¿Y ustedes tienen algún otro…?

B: Y hay otros abusos también po, que se les paga…

A: Claro, eso te iba a preguntar

B: Trabajan así jornadas extensas y se les paga menos del mínimo por lo mismo: "Ah, no tienes papeles, ya". No se paga ni siquiera el mínimo legal ¿cachay? Entonces…

A: Están sujetos a todos los abusos de la…

B: Sí, totalmente. Por eso hacemos este taller de trata

A: Claro, porque están como en el límite, en el limbo

B: Claro, sí

A: ¿Y ustedes tienen colaboraciones como con instituciones que puedan, digamos, insertarlos laboralmente? O con algunas empresas, no sé, como que estén más en este como…

B: Claro. Bueno, Pamela que es la que trabaja medios de vida, trabaja en coordinación con varias OMIL de diferentes comunas; con algunas ha hecho un trabajo más fluido y que hay más apertura, digamos, a recibir, a poder insertar a personas laboralmente. Y también tiene contactos y convenios con varias empresas

A: Ya

B: Con Castaño, no sé, y otras empresas como fábricas…

A: Que tienen un perfil un poco más como de protección, no sé

B: Claro, claro. Claro, como de derechos en el fondo

A: De garantía de derechos de los trabajadores

B: Que insertan a personas extranjeras, y que no les exigen esto de la definitiva

A: Y en cambio, en temas como más informales de abusos ¿en qué lo han visto? Porque todo esto, por ejemplo, de ver, no sé, a los haitianos vendiendo Súper8, vendiendo zapatillas en la calle y bueno, todas las redes que se han empezado a mirar, a ver en términos de abusos en vivienda. En el fondo, ¿qué es lo que está actuando en torno a estas, digamos, como zonas descubiertas que están en la protección de los migrantes?

B: ¿Cómo a qué te refieres?

A: Cómo han visto, digamos, todas estas cosas informales, o quizás, no sé, como redes más de abuso o incluso como medias criminales en las que están cayendo los migrantes

B: O sea, es que hay muchas de esas redes po; en lo laboral, en mafias, verdaderas mafias que captan a las personas y los hacen trabajar, no sé, doce horas todos los días, sin días de descanso, y como que o te sometes o sino chao, hay cincuenta personas más esperando este puesto de trabajo

A: Claro

B: Entonces se ven muchas veces como…

A: ¿Y en qué sectores específicos, o se encuentra…?

B: En construcción he visto mucho, sí, en ventas también, como esto de captarlos para las ventas informales, para comercio callejero… eso principalmente. En construcción es mucho. Bueno, y en temas de trabajos como de aseo en casas o en empresas de aseo, que contratan personas y los mandan a trabajar a diferentes, o a casas o a instituciones, hospitales, ese tipo de cosas. Y son jornadas, unos turnos así pero terribles igual, así súper excesivos, y les pagan menos del mínimo, no sé, ciento cincuenta lucas ¿cachay?, por trabajar todo el día, todos los días, diez horas diarias y con un día libre, así en condiciones…

A: ¿Y en temas de vivienda también lo han visto?

B: Sí, claro. Cobros así excesivos, cobran trescientas lucas por una habitación que con suerte tiene una ventanita, también en los cobros excesivos y condiciones mínimas po, o sea, baños compartidos ¿cachay?, cocinas compartidas, qué se yo

A: Claro

B: Casos de personas que tienen casa y subarriendan piezas, pero a precios así súper excesivos

A: Y el tema, volviendo al tema de salud, ¿tú ves que le gente tiene una demanda de salud?, o sea, les interesa inscribirse en Fonasa, ir al consultorio

B: Sí po, sí

A: Hay un interés propio de las personas

B: Sí po, hay una necesidad en la mayoría de las personas, sobre todo de familias con niños y todo. Y en invierno yo lo vi mucho po, como te decía, acá mucha gente muy enferma y necesitaba acceder a salud y no tenían la posibilidad, se les hacía muy difícil, o sea, tendrían que irse, siempre iban como, terminaban yendo a urgencias, a los SAPU, donde ahí sí te hacían la atención como de contingencia, digamos, para resolver una emergencia

A: Pero en los consultorios se les hacía difícil por eso que tú dices que la gente, en el fondo, no reconocía el hecho de que el solicitante tuviera derecho

B: Sí po

A: Eso ha aparecido como…

B: Eso es como lo más frecuente

A: Ya

B: Y la inscripción a Fonasa también. En Fonasa, tuve por ahí un par de casos, que no pudieron inscribirse finalmente, que los mandaban a uno y otro lado, y como que sacar trámites y que el carnet no sé qué, y no podían finalmente, no lograban inscribirse

A: Porque ellos lograron carnet luego de la solicitud ¿no cierto?, durante la… o sea, en el fondo, el rut ¿lo obtiene?

B: Claro, lo obtienen más o menos rápido, como a las pocas semanas parece que lo…

A: Ya, o sea, ellos pueden, o sea, ellos se presentan igual con un rut, el rollo es como…

B: Sí po. La mayoría de la gente ya tiene su rut y con eso ya pueden hacer todos los trámites

A: Entonces ya es como de desconocimiento

B: Sí po… no, y yo creo que cae muchas veces en el criterio de cada funcionario; hay personas que les ha tocado experiencias y que les facilitan todo y hacen todos los trámites súper expedito y todo, y otros que les ponen tantas trabas y todo que no logran inscribirse po

A: Pucha, yo tengo mi marido que es extranjero, y al principio no le querían dar Fonasa porque no tenía previsión

B: Claro

A: Entonces eso fue algo que se inventó la persona que… y cambiamos de oficina y encontró otra persona que lo inscribió sin preguntarle nada

B: Claro po, si queda muchas veces a criterio

A: A criterio. Qué fuerte. Bueno, gracias Inés, con esto estamos más o menos.

SOME

RECOLETA

A: Entrevistador
B: Entrevistado

B: Todo tiene que ver con los refugiados, ya estamos… acá al menos no identificamos cuando son refugiados o no porque son muy pocas las personas que…

A: Lo identifican

B: No

A: No

B: No hay una, por ejemplo, una pestaña que nosotros, porque nosotros trabajamos con el sistema rayen

A: Rayen, ya

B: Que aquí está el sistema rayen. Entonces, en ninguna parte acá nos dice si son refugiados o no, solamente nosotros colocamos el país de origen

A: País de origen, ya. ¿Y ahí tampoco sale nada como de la visa?, que cuántos meses tiene la visa, nada

B: No porque hay gente, distintas condiciones po, si llegan distintos tipos de usuarios extranjeros

A: Ya, y eso, ¿no pesquisan distintos tipos de visa?, por qué te lo pregunto, porque otra forma de, sin que la persona lo diga, de identificar a un refugiado, es que tenga una visa de ocho meses. Es como algo súper particular

B: Claro, pero acá por lo general la gente llega con pasaporte

A: Claro

B: Una vez que tengan el pasaporte, y después traigan, cuando ya hacen el trámite de la visa y se regulariza, ahí ya nosotros ponemos… lo que se coloca acá es más que nada si son, tienen visa temporal, definitiva. Pero yo creo que cuando pasan a consulta más adelante, con la asistente social, yo creo que ahí se puede identificar. En mi caso, que yo comencé a atender ahora como asistente social, yo no me he encontrado a nadie de refugiado ¿cachay?, pero no lo hemos visto en eso. Por eso yo te puedo hablar del tema como de salud, cómo llegan, cómo se integran… yo sé que Fonasa también tiene una parte para refugiados y que tienen asilo, que le piden un documento especial

A: Ya, y ¿cómo das esa información?

B: A ver

A: O sea, ¿ha pasado el caso de que soliciten información o revelen la condición?

B: Mira, yo en un momento me encontré con una refugiada, ahora que me acuerdo, que me señaló, me dijo: "yo tengo…" no sé si fue asilo o estaba refugiada, eso no recuerdo

A: Entonces, ¿qué pasó en esa situación?

B: Lo que pasa es que cuando la chica me dice que era refugiada, porque estábamos viendo el tema de Fonasa, no me acuerdo si era… con asilo político me parece. Entonces, como nosotros tramitamos el número provisorio de Fonasa y que tiene que ver con el decreto y que se dictó del 2016, ella me dijo a mí po, me dijo: "oye, sabes que yo tengo asilo, yo tengo asilo político ¿cómo lo hago?", y yo ahí me comuniqué con Fonasa y me decía que la gente que tiene un asilo de refugio y viene con una pensión, que si era con asilo político venía con pensión desde el país de origen, y que si era así ella tendría que traerme el tema del documento de la pensión, que no es la palabra pensión, pero tiene relación con algún tipo de…

A: Remuneración

B: De remuneración por ser asilo, y ahí se le daba no el Fonasa A, sino el Fonasa B

A: Fonasa B

B: No sé si por Derechos Humanos, ya no me acuerdo, fue hace como un año. Entonces, la chica me dijo que ella tenía asilo y que desconocía que tuviera algún tipo de pensión o algo así ¿cachay? Y ese yo lo he encontrado bajo al menos, pero nosotros… yo al menos cuando he trabajado en OIRS, porque ahora trabajo menos horas en OIRS, se le da la información completa, y yo trato de preguntar igual en qué condición vienen, pero en ningún momento revelan, por ejemplo, que vienen refugiados

A: No lo dicen, ¿qué dicen?

B: No porque, es que hay mucha gente que llega ya desfasada, que llegó al país hace ocho meses y se vino a inscribir tardíamente al Cesfam ¿cachay?, entonces…

A: Cuando les pasó algo

B: Cuando les pasó algo, yo siempre le digo a la gente extranjera que si está en un país desconocido, lo primero que tienen que hacer es asegurar la salud porque si vienen a trabajar o vienen a echar raíces, si no tiene salud o desconoce cómo es el sistema, sobre todo cuando quedan embarazadas, por ejemplo… etcétera, etcétera. Pero tiene que ver con eso porque depende como uno se presente con la persona, que te vaya revelando si viene arrancando o algo por el estilo, pero yo creo que…

A: Como la relación que establecen

B: Claro

A: Ya, y en esas circunstancias ¿te cuentan…?

B: Por eso te digo, si ya entramos en confianza, por ejemplo, ahora con los venezolanos que vienen con un poquito más de sentimientos encontrados porque dejaron su país, porque ellos llegan en familia, no llegan solos como los haitianos, por ejemplo, ellos igual te informan del tema político, que están súper complejos, que tienen que venirse sí o sí y dejar todo. Entonces, vienen con harta amargura. De hecho, conocí a un caballero que le dio un infarto cuando llegó acá, pasó un mes y le dio un infarto, no podía creer cómo estaba su país

A: Claro

B: En eso va. Va a depender de la comunicación que yo pueda entablar con la persona. Por eso cuando esta chica me dice que tenía asilo político, me dijo: "no, sabí que yo vengo por esta condición", y ahí yo la mandé a Fonasa en realidad, pero no me acuerdo ya en qué habrá quedado esa situación

A: Se inscribió y no la volviste a ver

B: O sea, se inscribió, hicimos los trámites correspondientes que nos corresponde a la OIRS en este caso, y después ya ella tendrá que, no sé, haber ido a control, por ejemplo. Pero yo creo que tampoco, es un tema porque hay mucha, una ola de migrantes tremenda, entonces yo creo que la gente no anda diciendo: "no, yo soy refugiado, yo soy… yo tengo asilo político". Yo creo que no está como en el consciente de las personas. Yo creo que más de alguna persona viene con refugio, pero no lo anda diciendo

A: Pero acá, en el fondo, igual se les da la atención. Por lo que comprendo, por Recoleta que está sensibilizado, como independientemente, solo con el pasaporte, no tiene que…

B: Lo que pasa es que todas las personas extranjeras se pueden inscribir

A: Todas

B: Con el pasaporte. O con cualquier documento de identificación, ya sea pasaporte, el DNI, el certificado de nacimiento en el caso de los niños del país que corresponde. Tiene que tener algún documento, algún número como yo digo, porque acá en Chile todo se tiene que relacionar con algún número, entonces tiene que tener algún número pa' poder meterlo al sistema ¿Qué es lo que pasaba antes?, que como no estaba el tema de Fonasa, nadie pagaba esa atención, entonces era, se inscribía mucho extranjero, pero el per capita era solamente para los chilenos

A: Lo del año pasado

B: Entonces, bueno, ya con el tema de Fonasa, ya se arregló un poco, ya toda la gente puede acceder a las prestaciones de la atención secundaria. Porque solamente los podíamos atender acá, pero en el hospital ¿quién le pagaba al cirujano, el parto?, nadie cubría eso

A: Oye, y entonces usted me contaba que vienen mucho y, en el fondo, no es necesario… tampoco necesitan develar su condición

B: No. No porque igual lo van a atender acá. Si, independiente de que haya escapado, arrancado, no, lo van a atender igual. Yo creo que la ayuda que ellos tienen va por otra parte, por otra área, por lo social, por ejemplo. Yo, en algún momento, había escuchado que iban a llegar familias refugiadas; hace como dos años escuché que estaban preparando, que iban a llegar no sé, como a Patronato, no sé, pero no sé en qué habrá quedado eso… que eran como de Irán, no me acuerdo de qué parte iban a llegar como cinco familias. Ah, iban a llegar y después al final no llegaron acá, se fueron a otra comuna. Pero ellos era porque venían como diez, veinte personas

A: Claro, sí. Oye, y el tema de… no sé po, con Inga o de ella particularmente, ¿ves que ella pesquisa algo?, como ella está en el área social de la municipalidad

B: Yo creo que a ella le llegan más casos así que a nosotros

A: ¿No se contacta con ustedes? "oye, sabes qué, tengo una chica que necesita algo, denle más prioridad"

B: Inga me llama. Yo soy el contacto con Inga, por ejemplo, dice: "Sergio, me llegó esta chica colombiana, podí revisar que se inscriba", pero siempre hay comunicación en ese sentido. Porque, a veces, la gente llega a la muni en vez de llegar acá, entonces se dan una vuelta más larga, entonces después la Inga me llama y me dice: "oye, hay esta persona…" o Delia también me dice: "Sergio, ¿Qué pasa con esta chica? ¿Está inscrita o no está inscrita?" y de ahí viene para acá

A: Claro

B: Mucha gente llega allá directo, obviamente por desconocimiento ¿cachay? Eso…

A: Oye, y Delia o Inga no te han nunca revelado como: "oye, este caso es refugiado, tiene tal problemática"

B: No, no. Que por eso yo te digo, yo tratando de, cuando leía la investigación…

A: Trataste de reflexionar

B: Yo estaba tratando de hacer memoria, de ver si había personas, porque por eso me acordé de esta chica. Pero, como te digo, yo creo que la gente, si viene refugiada o con asilo, yo creo que viene con temor. Entonces, tampoco anda hablando de que somos refugiados, a no ser que sea como una comunidad, como te daba el ejemplo de que, estos eran como cinco familias y que todo el mundo sabía que venían refugiados, no sé qué

A: Sí, como mediático, la foto con la presidenta

B: Sí, sí, entonces…

A: Pero esos son los reasentados, que son distintos. Los refugiados se diferencian del reasentado, que el refugiado es espontáneo y el reasentado es un programa, un estado se compromete con esta (no se entiende 11:07). Entonces, como que ahí…

B: Claro, yo me acuerdo que ese tema de las familias también fue abullado porque que no querían llegar acá, que se iban entre Argentina y Chile…

A: Oye, y volviendo a la temática de refugio, yo sé que como que las capacitaciones son concursables, y ¿han tenido capacitaciones con temáticas de refugio?

B: No, no. En el último tiempo, no. Yo he ido como a charlas, pero eso fue como el año pasado

A: Charlas de…

B: De refugio, pero fui como a una

A: ¿De qué? ¿de salud?

B: Sí po, de salud. Pero las temáticas igual se van repitiendo porque, al final, más que la charla o la atención de salud que se tiene que brindar ¿cachay?, que son temas que son específicos

A: Ya

B: Ahora mi colega, la otra vez, fue a otra charla, pero tiene que ver con la trata que también se estaba dando en el último tiempo, que tampoco lo estamos identificando y que también salió por un caso de trata y que acá en salud no estábamos preparados para recibir gente que te dice que no la dejan salir, que está trabajando todo el día, entonces ahí uno puede…

A: Usted puede hacer como esas preguntas indicadoras

B: Sí po, entonces yo creo que cuando uno está en consulta recién ya uno puede entrar en otra área porque abajo llegan a conversar y: "tiene que inscribirse, necesita esto" ya, démosle

A: Claro po, en el fondo, como que hay… están esas como preguntas pa' identificar a una persona en trata, pero una pregunta para identificar a un solicitante de refugio es cuánto dura su visa

B: Claro

A: Si te dice ocho meses, es refugiado

B: Claro

A: O están solicitando

B: Claro. Por eso te digo, que ese tema salió a raíz de eso y que se hizo una mesa de trabajo en el servicio, y mi compañero dice sí, me parece interesante porque nosotros de salud igual estamos súper limitados; que llegan con muchas problemáticas y nosotros no podemos resolver, si nosotros somos salud, tenemos que ver primero la prevención, la promoción de la salud, pero no… por ejemplo, ¿cómo ayudamos a una persona que tiene trata más que hacer una visita y ponerlo en la denuncia no más po?, y nosotros hasta ahí no más llegamos

A: Oye y tú me mencionabas que algunos temas se repetían en esta capacitación

B: O sea el tema del acceso a la salud, temas de la atención con el extranjero

A: ¿Y en atención qué temas…?

B: O sea, que el acceso, como que se tiene que atenderlo, pero yo creo que, mi crítica es que hacen temas como muy generales. El año antepasado fui a una capacitación que decía de… de extranjero, salud, sexualidad… no me acuerdo cómo era el tema, que era así como bien rimbombante salió, era como salud, sexual, no sé qué, no sé qué, del extranjero, y hablando de lo mismo de cómo tenía que atender, cómo llegaban, de qué parte vienen, que tienen que… el tema es que no se ha sabido trabajar bien en el tema, o sea, tiene que ver que hay mucha gente que es extranjera y cómo las culturas se van mezclando ¿cachay?, no hay una capacitación que diga: "ya, la gente haitiana tiene esta cultura, la gente venezolana tiene esto, tiene esto, los chilenos somos así", y cómo nosotros esos conocimientos los abrazamos y hacemos una atención general po porque, por ejemplo, lo mismo que hablábamos la otra vez, que las haitianas no vienen a los controles porque no saben, porque ellos tienen otra cultura, las peruanas llegan a los ocho meses porque allá en Perú recién se empieza a controlar al séptimo mes ¿cachay?

A: Como, sí po, no se da la atención como integrando a la gente

B: Sí porque nosotros, llegan los extranjeros, pero nadie nos dice: "oye, a este hay que tratarlo de esta forma, que el haitiano, que el hombre es más machista" ¿cachay?, uno lo sabe porque lo ha vivido no más po, pero tampoco hay un tema que se diga: "ya, vamos a atender así, esta es la lógica, estamos trabajando de esta forma"

A: Se recomienda hablar con estas personas de esta forma

B: Claro po, los mismos temas de la…

A: Nada especifica

B: Los mismos temas de la nutricionista que yo, a veces, cuando yo hice la práctica profesional, porque estuve en Peñalolén, hicieron con la nutricionista una pauta de alimentación y mezclaron alimentación de varios países. Entonces, le ponían palta y entre paréntesis le ponían como le decían en México, en Colombia, no sé qué, aguacate, no sé qué, le ponían la medida en onza más la medida en gramos ¿cachay?

A: Claro, como integrante, como que las capacitaciones no van al grano

B: No po, solamente son generales. Ahora, hace poco, la referente de migrantes que tenemos, que es Maira, que también trabajaba con Inga, se hizo con un médico haitiano y hablaron del Vudú, por ejemplo, cómo es la creencia que tenían ellos y eso también es interesante saberlo

A: Más profundo

B: Sí po

A: Además que ya entra todo el acceso de (no se entiende 16:23)

B: Sí po, por ejemplo, yo siempre he dicho que las haitianas que son más yerbateras para todo lo de la salud, debería haber una instancia en que juntemos a todas las haitianas y que hagan su trabajo como en el sur que hay una ruca mapuche ¿cachay?, y acá hay harto haitiano, y ellas tengan la posibilidad de tener una medicina alternativa porque ellos son de mucha yerba

A: Sí po, porque… como, sí po, hay como consultorios policulturales donde están como la (no se entiende 16:52) y está la Jessi y como que conviven

B: Sí po

A: Pero acá no está

B: Entonces, si bien acá en Recoleta si bien se abren las puertas, pero se abren las puertas no más po, o sea, atendamos no más, pero no ven más allá

A: No conozcamos profundamente. Y entonces, del refugio, de esta charla que fuiste del refugio ¿no te acuerdas de algo así como que…?

B: Es que era como parte del tema, era como migrante, refugiado, pero era como qué eran los refugiados, pero más allá…

A: Más como, te explican algo del refugio, pero así como a grandes rasgos

B: A grandes rasgos, sí

A: Una pasadita rápida

B: Sí

A: Bueno, pasando a otro… qué, ¿tú sabrías diferencias entre un migrante y un refugiado?

B: Así ¿a la vista? Jajaja

A: No, no, pero así como… ¿sabes la diferencia?

B: No. Por eso te digo, tendría que hablar con ella, si por eso te digo, cuando yo converso con…

A: No, pero como conociendo, "ah, este es…"

B: Sí, pero igual depende de la postura con que llegue la persona ¿cachay?, porque hay gente que le interesa su salud, así como tratando de hilar un poco, y uno dice: "ah claro, él…", es que nos pasa mucho con la venezolana, como ya sabemos el contexto, pero hay venezolanos que llegan súper tranquilos, pero hay otros que yo diría que sí vienen arrancando porque vienen con otra, cómo te puedo decir, con otra postura ¿cachay?, con otro ánimo, desconcertado, a veces sin siquiera querer atenderse, así como…

A: Como susto

B: Sí, como con susto. Yo creo que ahí nos ponemos de acuer… es que ese es claro ejemplo, porque si mañana llegan puros, no sé, suecos, también les vamos a decir “ohh…” también están llegando así. Pero igual hay distintos tipos de venezolanos que llegan, o sea, está esta gente que ya viene resignada “ya, nos tenemos que mudar porque nos está yendo mal”, pero hay otra gente que yo creo que sí viene arrancando porque quería salir ya, quizás, por temas políticos. Pero igual ellos ponen como una barrera, porque yo también les he preguntado: “oye, pero ¿te pasó algo? ¿tú estás en alguna organización?”, me dijo: “sí, pero me tuve que venir”. Así como “sí, pero me tuve que venir porque ya no da más, ya no da más”

A: Ya no da más, así como…

B: Sí, pero de hecho yo le pregunto a la gente, o sea, a los venezolanos en este caso que es la moda, de hecho, ahora estamos con más venezolanos que haitianos

A: Los superaron

B: ¿Cachay?, las tres tendencias, yo he estado haciendo números provisorios y los voy clasificando por nacionalidad, y las tres tendencias son haitianos, venezolanos y los peruanos

A: Ah, ¿y colombianos no tanto?

B: No

A: Ah…

B: No, es poco porque va a depender del contexto po. Pero la gente dice: “ah, están llenos de peruanos” no es así, estamos llenos de venezolanos ahora porque los venezolanos llegan en familia

A: Las familias, las hijas…

B: Sí po, llegan al tiro las hijas y las chicas llegan con tres hijos, cuatro hijos

A: Oye, y entonces como que este ánimo de, como que hemos identificado dos perfiles de venezolanos; uno como que viene más asustado, pero tampoco revela mucho, y el otro como que es más sencillo

B: Sí. Y yo por lo que me puedo dar cuenta abajo

A: Sí, de lo poquito, que es como a la rápida

B: Sí po, a la rápida. Yo una vez le dije a un caballero: “¿pero usted volvería?”, me dijo: “no, de aquí esto tiene pa’ rato. Y yo ya estuve ahí, entonces yo ya sé, y estuve ahí en algo –me decía-, pero esto aquí si no hay un cambio drástico… no”. Porque a mí igual me interesa saber por qué po porque yo igual lo encuentro valiente ¿cachay?, empezar de cero, empezar de cero es súper complejo. Entonces, porque hay mucha gente que es médico, que es ingeniero, que son abogados, entonces partir de cero y tener que… yo conocí un abogado que estaba limpiando autos, por ejemplo. Entonces, me decía que no…

A: Bueno, las cosas que han pasado con los colombianos, o sea, venezolanos, es como que ha salido el tema como que al venezolano como que le cuesta asumir que está mal

B: Sí

A: ¿Lo has visto tú?, que le cueste asumir que necesita ayuda, como que es como orgulloso

B: Yo creo que vienen con mucha expectativa, yo creo que… hubo una vez que yo le pregunté, no sé si fue a un haitiano, a un boliviano que vendió todo y se vino, y yo le dije: "pero ¿y por qué se vino usted? –no, es que me dijeron que aquí era súper bueno. Y vendimos la tierra y todo", entonces yo creo que se hacen muchas expectativas, y cuando llegan acá llegan a una parte específica, por ejemplo, Recoleta, que hay muchas otras comunas y aquí vienen y… lo que sí me he fijado es que vienen con dinero y que pagan el alquiler por tres meses, seis meses

A: Los venezolanos

B: Y después de eso ya comienzan así como "no encuentro trabajo, ¿qué es lo que vamos a hacer?" y empiezan con la desesperación. Pero sí ellos, yo he visto como que arriendan departamentos, casas, no así como los haitianos que llegan directo a la pieza

A: Claro

B: Como que tienen otro estatus también, porque no son cualquiera, no es un venezolano campesino el que se viene po, el venezolano campesino se queda allá, no tiene los medios

A: Sí po, viene como la Recoleta (no se entiende 22:50)

B: Entonces sí po, porque en esa consulta, por ejemplo, ese que te contaba yo que es abogado y vino a pedir ayuda para pagar el arriendo. Entonces, mi colega le dijo: "pero búscate algo más barato, no tenis pega pero veni a pedir ayuda para ayudar a pagar arriendo" porque era abogado, qué se yo, y no podía estar viviendo en otras condiciones ¿cachay? Pero la última gente… yo creo, igual son súper amables

A: Oye, pero el venezolano como, no sé, como… vuelvo a insistir con esta pregunta que, como de su carácter, de contar que está mal ¿le cuesta pedir ayuda o no has percatado eso?

B: O sea no, yo creo que la gente más adulta viene con, es más resistente. Ya los más jóvenes ya vienen dispuestos. Porque, como te decía po…

A: Indiferente de la nacionalidad

B: Indiferente, pero la, o sea, la mujer venezolana, o sea, yo lo encuentro muy amable en todo sentido, porque están recién llegando, pero ya cuando se empiezan a dar cuenta que la hora se demora, que no sé qué, que la cuestión, como ahí empieza como la resistencia. Pero yo creo que, no sé si les costará, porque yo creo que lo hacen más por los niños, me he fijado que son súper preocupados en ese sentido

A: Ya

B: Súper preocupados

A: Oye y pasando a otro tema, ¿cómo es la atención de salud mental de migrantes?

B: ¿Cómo?

A: Que, si pasan… ¿cómo se da, son demandantes de atención mental, de salud mental?

B: Son… a ver

A: ¿Piden atención pa' eso?

B: Es que todo va a depender de la atención, si la primera atención que tengan. Por ejemplo, ideal es que cuando llegue un extranjero se inscriba, pase al tiro por asistente social para que pueda identificar si hay alguna patología o algo que ve la asistente social, lo manda al tiro al psicólogo, por ejemplo

A: Ahí lo deriva

B: Ahí lo deriva. O las gestantes también, porque tienen una escala acá cuando llegan, o sea, la matrona le aplica un instrumento y ve que, no sé, que hay una depresión leve, también lo mandan al psicólogo. Pero nosotros igual conversamos en esa oportunidad, que Boris también exponía que la depresión que nosotros tenemos es muy distinta a la otra persona po, porque nosotros ya estamos aquí estresados porque, no sé po, los viajes qué se yo, y la otra persona extranjera viene con otro tipo de estrés

A: Claro

B: Que no entienden la gente. Que, por ejemplo, esa vez yo me acuerdo en un focus group hablamos del tema del dominicano, que no entendían, que acá recién conocen la depresión

A: Claro

B: Acá recién conocen y se empiezan a…

A: Como que acá tiene el nombre

B: Claro. Allá lo viven no más y están muertos de la risa, a pesar de todo, le dan, le dan, acá no. Yo creo que Chile es moda, todo es moda

A: Todo es moda

B: Sí, antes era el estrés, ansiedad, depresión, ahora todo el mundo es asperger. Yo muchas veces he escuchado: "no, es que ella es asperger", todos son asperger, todos tienen…

A: Bueno, pero y volviendo al tema de la salud mental y, ¿qué pasa cuando ya este…?, ya, lo derivan, en esta primera atención lo derivan, ¿cómo es el tránsito de esa persona? Pongamos el caso equis que esa persona lo derivan y digan: "no oye, tiene que venir con la psicóloga una vez al mes o dos veces por semana" cualquier cosa, ¿vienen?

B: Sí po, vienen

A: Como son adherentes, no son adherentes

B: La gran mayoría es adherente, la gran mayoría. Pero también ocurre que el mismo sistema los va asfixiando porque ya una vez al mes ¿cachay? Ya, a veces, la psicóloga no viene y se le atrasa la hora, pero sí yo me he fijado que ellos cumplen harto

A: Adhieren a salud mental

B: Vienen, vienen

A: O adhieren a todo

B: Adhiere. Lo que sí me he encontrado, por ejemplo, que como cinco casos que hay gente que se ha ido al Horwitz, al psiquiátrico. Porque es tanto, porque se le declaró una esquizofrenia ¿cachay?, se les declaró otras patologías de salud mental. Porque me vienen a pedir el tema de Fonasa, entonces yo les digo: "Uy, ¿qué le pasó? – No, es que está en el psiquiátrico, o le dio una crisis" ¿cachay?, o les da algún tipo de enfermedad y tienen que ir al San José. Ahí me encuentro con harto… harto venezolano con dolor de estómago, por ejemplo, que pa' ellos es raro ese tema del estómago, tiene que ver también con otros factores de alimentación ¿cachay?, de los nervios, no sé. Pero adhieren la gran mayoría, pero va a depender de la, como te digo, cómo ellos vean el problema que tienen po. Por eso te digo, en temas de la depresión ellos, depende de cómo lo vaya a tratar la psicóloga

A: Cómo se lo explique

B: Sí, cómo se lo explique, cómo él lo manifieste también

A: Las dos cosas

B: Claro, porque a lo mejor para la psicóloga chilena eso es estrés y para el, no sé, haitiano, eso es pena no más ¿cachay? Pero sí me he encontrado con varia gente que ha ido a parar al psiquiátrico

A: ¿Y desde qué particularidades, o qué más podrías profundizar de eso?

B: No po, yo… el que te conté fue el tema de esquizofrenia que a mí me llamó mucho la atención porque era un jovencito venezolano y que venía con los hermanos, entonces yo le pregunte, le dije: "oye, ¿pero qué le pasó? – No- dijo. Si en Venezuela estaba bien, llegamos acá, pasó un mes y se le declaró esto, una esquizofrenia", que empezó a transmitir cosas y yo creo que también el mismo tema tiene que ver con el cambio po

A: Claro

B: Y yo dije: "pero era súper sano", me dijo: "sí, si era súper bien. Llegamos acá y lo perdimos". Como a tres personas les ha ocurrido eso, como con crisis que les ocurren

A: Como les desata

B: Sí

A: ¿Cómo se trabaja el acompañamiento y apoyo en caso de riesgo bio-psicosocial?, como cuando ya son riesgos más…

B: Riesgos, es que hay varios tipos de riesgos. Yo, al menos, estoy comenzando a atender nuevamente, pero… depende del riesgo, te voy a dar el ejemplo de mi colega que ha… la haitiana en la última semana han tenido mucha violencia intrafamiliar

A: Ya

B: Y han llegado acá, por ejemplo, con moretones, qué se yo, con la asistente social y ella la asesora y la manda al Centro de la Mujer, porque ahí ya tienes que judicializar y todo. Entonces, ahí después le hacen seguimiento, después se les hace visita ¿cachay?, todo ese tipo de cosas. Pero ahí van con el traductor, de hecho, hubo un caso súper *heavy* que el traductor estuvo todo el día porque tuvo que ir al Centro de la Mujer y hacerle una contención, después ir a Carabineros a hacer la denuncia

A: Acompañamiento total

B: Total. Pero es cuando, en la medida que se pueda. En la medida que se pueda porque nosotros, al menos, y eso es lo que la gente no entiende, como asistentes sociales es igual que los médicos, los médicos especialistas y nosotros somos salud. Nosotros no podemos, por ejemplo, si llega alguien violentado e ir a hacer la denuncia porque nosotros tenemos agenda, ¿cómo seguimos atendiendo a la otra gente? ¿la dejamos botada? ¿atendemos a la gente que viene por violencia? Pero estos casos han sido fortuitos, al menos, la Tamara ha tenido la ventaja que no tenía agenda y ha podido hacer el trámite

A: Que es la traductora del consultorio

B: O sea la Rose es la traductora, pero la Tamara es la asistente social, entonces se ha dado el tiempo, y a ella le ha tocado como tres casos así

A: Ya

B: Pero de haitianas

A: De haitianas, específico

B: Sí, de haitiana. Sí porque son más machistas las haitianas, o sea, los haitianos son súper machistas. Aparte que tiene que ver con la convivencia familiar, quién manda más en la familia, entonces…

A: ¿Y otros casos se han dado?

B: Así como fuerte, no

A: ¿U otros riesgos?

B: Es que tenemos riesgos que son más comunes, por ejemplo, el hacinamiento ¿cachay?, que se van a dar siempre acá, los subarriendos, y eso desencadena que la guagua esté enferma porque no tiene ventilación, y así vamos sumando

A: Claro

B: Que son determinantes social ¿cachay?, que están enfermos, pero eso tiene que ver con todos los, con todo su contexto, con su entorno que hace que se enfermen

A: Claro

B: El mismo tema de que la hora, que en la mañana se van a las siete y media y están todos con las guaguas afuera esperando la…

A: Pero cambió eso

B: Sí po, a partir de ayer, ayer comenzó

A: Ah, octubre y como que la gente…como que salió el pajarito "ya no trasnoche, vecino" ¿y cómo ha aflorado eso?

B: Bien, o sea, estamos empezando po. Yo, al menos, es que no he estado mucho abajo, pero igual llega harta gente. Es que hay que ver qué es lo que va ocurriendo en el camino

A: Pa' mejorar

B: Porque se supone que eso se ha enfocado en la gente adulta, mayor, las que tienen guagua, las embarazadas, pero qué ocurre con el adulto que trabajo y tiene que pedir hora temprano porque tiene que ir a trabajar, ¿qué ocurre con esa población? O sea, la idea es buena, pero qué ocurre con esa… ¿le damos la hora no más, o que venga más tarde?, porque aquí se hace un triash y ahí se decide si se atiende hoy día o mañana

A: ¿Cuál es el triash?

B: El triash es que se colocan a unos profesionales y empieza "esto necesita médico ¿sí o no?" esto sí o no, porque mucha gente viene a consulta porque le duele esto no más y eso no necesita médico aún po

A: Claro. Es compleja la situación. Oiga ¿y qué más, como en torno a…?

B: Qué, qué

A: ¿Qué recursos o cosas siente que serían útiles para trabajar más con esta población? Dar como una atención más pertinente, volviendo al tema de la atención de migrantes, refugiados

B: Yo creo que hay que partir con información, por ejemplo, qué cantidad de personas, de refugiados vienen al país porque eso tampoco nosotros lo sabemos ¿cachay?, no lo manejamos. Por ejemplo, a la gente PRAIS se supone que se le debería dar una atención especial por ser PRAIS

A: Claro

B: Y se supone que la hora debería ser de otra forma, que ellos, los PRAIS no deberían hacer fila, por ejemplo, porque tienen un tratamiento especial. Debería ser así. El mismo tema de la interconsulta, ese es el ideal. Yo creo que a un refugiado o…

A: ¿Al PRAIS se le facilita la interconsulta?

B: Se le facilita

A: Porque sabemos que es de una dificultad en Fonasa…

B: Pero, ¿a quiénes? ¿de PRAIS?

A: O sea, no siendo, pero una persona Fonasa A con las demoras, es difícil la interconsulta

B: Es difícil

A: Entonces al PRAIS, como se asume que tiene una carga distinta

B: O sea, siempre y cuando él… lo que pasa es que yo también fui a una capacitación PRAIS, pero eso es cuando él va a Maruri, donde está la oficina PRAIS, y dice: "tengo que hacer esta interconsulta, ¿ustedes me pueden ayudar?", ellos hacen el tema

A: Ah, le activan las redes

B: Le activan las redes. Pero acá no, acá se atiende como persona normal. El ideal, o sea, lo teórico es que una persona PRAIS, te hablo de un PRAIS que sea original po, un PRAIS de ochenta años, de setenta años, porque hay nietos que también…

A: Son PRAIS

B: También son PRAIS, pero ellos no tuvieron consecuencias de, pero los que son más afectados son la gente adulta, entonces ellos se supone que debieran tener una atención especial y ya nosotros conocemos que son PRAIS, por ejemplo

A: Las arroja, ¿el sistema las arroja?

B: O sea, cuando vemos Fonasa, dice Fonasa PRAIS, Isapre PRAIS, todo PRAIS

A: Puede ser, ya

B: Y con los refugiados también se podría, como tú dices, un indicador que tienen la visa por ocho meses y nosotros no, nosotros en la inscripción no vemos por cuánto tiempo tienen la visa ¿cachay?

A: Pero una forma de no preguntar la condición

B: Claro, porque a nosotros nos interesa inscribirlo. Pero sí, quizás, ver qué medidas están llegando personas refugiadas, así como se sabe que son migrantes que vienen y que son quinientos, ¿cachay?

A: Como identificarlos dentro de la comuna

B: Identificarlos dentro de la comuna, identificarlos porque, por ejemplo, hay gente, los haitianos todos están por allá, y hay gente de acá más peruanos, acá más venezolanos, entonces uno sabe cuál es, allá hay más población adulta mayor, allá no tanto ¿cachay?, yo creo que esa es una buena medida, saber, o que la persona porque yo no sé si cuando vienen al país, en el pasaporte dice refugiado ¿cachay?, esas cosas ¿cachay?, como…

A: Informarse

B: Cómo sé que es refugiado y que viene con otras problemáticas emocionales, cómo yo lo puedo ayudar de mejor forma si no tengo, si el pasaporte dice normal, "ah ya", para mí es otra persona más no más "ya, inscríbase, vaya para allá, vaya para allá, haga esto y esto", siendo que si es refugiado le puede dar al tiro hora con asistente social y pa' que ahí se vayan armando las redes al tiro

A: Y pa' evitar…

B: Y ahí nosotros poder avisar, decirle: "oye, oye Delia, sabis que esta persona es refugiada ¿cómo lo hacemos? ¿tiene algún beneficio?" no sé, y ahí vamos ¿cachay?

A: Sí, "viene tan perturbada, quiere traerse a la hija y no sabe cómo, reunificación familiar"

B: Sí po. Entonces eso, la persona que es refugiada y que no sé, si llega la muni, decirle: "usted vaya y pide una asistente social para ver cómo lo podemos abordar". Pero si nosotros no sabemos cuántos son, no sé, si no vienen con un sello, o sea, tampoco van a estar marcados, me refiero a que "ah están marcados, ah tú eres refugiado"

A: Te refieres a como…

B: Pero sí debieran tener otro documento o en el mismo pasaporte, no sé

A: Indicado

B: Sí

A: Que sea reconocible

B: Sí po. Porque nosotros, yo no sé si es una gran cantidad de refugiados ¿cachay?, y un grupo que está en investigación también po

A: Sí po

B: Pa' poder identificar y también…

A: Como qué hacer

B: Sí po

A: ¿Quisieras agregar algo?

B: No. No sé, si esta era cortita yo creo

A: Ya, gracias Sergio, que estés muy bien, gracias

SEGUNDO AUDIO

A: Me estabas constando, Sergio, si lo puedes repetir

B: Que aquí debiera haber un sistema que la gente extranjera llegue y se le haga como una capacitación de todos los servicios que tenemos

A: Ya

B: Para que no anden dando vueltas por todas partes. Si de ahí salgan casos ¿cachay?, y se van repitiendo las mismas temáticas, que salud, que el carnet, y el jardín, qué se yo. Debería haber una forma de juntarlos a todos o aquí mismo, ya… aquí se están haciendo charlas, por ejemplo, para los extranjeros, de diferentes temas, por ejemplo, que tienen que ver con los problemas de migrantes que llegan ahí y qué se yo. Pero, nosotros tenemos que darles herramientas a la gente porque si no nunca van a aprender el sistema como de nosotros, porque si yo…

A: ¿Y cómo les das ahí herramientas si no con charlas?

B: ¿Ah?

A: Con charlas…

B: Con charlas, con talleres

A: ¿Pero las charlas no se están haciendo?

B: Se están haciendo charlas, pero recién… nosotros llevamos en Recoleta cuántos años y la comunidad sigue aumentando, aumentando, aumentando… estamos **(no se entiende 01:05)**. Pero recién ya, la Maira al menos está haciendo talleres, pero eso se podría hacer masivo, a nivel municipal ¿cachay?

A: La Maira es la asistente social de este consultorio

B: Sí, ella está viendo el programa migrante con la Inga, la Inga que ahora la tenemos acá. Pero si yo, por ejemplo, si llega una persona y yo empiezo a llamar por teléfono: “oye, consígueme esto. Oye, consígueme esto y eso”, al final no va a saber la señora cuál es la vuelta que ella también tiene que hacer en algún momento

A: Claro, como conocer los caminos

B: Sí po. Entonces, tengo que decirle: “mire, esto es así…”

A: ¿Y eso es lo que tú le llamas asistencialismo?, como entregar las cosas como…

B: Claro, oye si vamos a ver, está bien, todos tenemos un grado de urgencia, todos. Obviamente hay que ser criteriosos, no va a ser una persona que llega sin un brazo, que nunca me ha ocurrido, obviamente no vamos a esperar que la señora vaya. Hay que ayudarla,

obviamente. Hay criterio ¿cachay?, pero yo encuentro que todavía están ahí siendo una, una… que Recoleta es una comuna tan inclusiva, no sé qué y la cuestión, yo creo que igual nos falta, le falta empoderarse en ese sentido, tomar la batuta, ya el alcalde ha tomado la batuta en varios temas. Pero todavía siguen migrantes, todavía estamos en la… dando vueltas todavía

A: Todavía tienen población como repitiendo…

B: Sí po ¿cachay?, los problemas se siguen dando vueltas, se siguen dando vueltas

A: Como infinito

B: Como infinito, y no hay un centro, una casa de acogida de migrantes, por ejemplo, no hay. Donde la gente migrante llegue y sea especial para ellos, donde haya un plan, yo una vez decía, un plan que sea… o sea, igual se necesitan recursos, pero esto es una utopía, pero que haya como un consultorio de migrantes y diga ya: “aquí usted va a estar tres meses, le vamos a dar toda esta orientación completita. Listo, ahí está”

A: Como que pase por un programa y se egrese. Por ejemplo, hay mil programas donde la gente egresa

B: Sí po. Y ahí, tomar tres, otro grupo más: “ya, tenemos cincuenta cupos”, como el Programa Calle que toma cincuenta personas y los alimenta, les da las duchas, no sé qué, darles el empujoncito pa’ que digan ya

A: La información, “esto es tal así, tienes que venir hoy día, hoy día te vamos a enseñar cómo funciona el asunto…” no sé, educación. Algo así es tu idea

B: Sí

A: Como generarle un, como que…

B: Yo una vez le preguntaba a Orlando, que era un traductor, que por qué los haitianos tampoco se organizan y no se hacen, por ejemplo, un carnaval, porque él me contaba que en Haití hacían carnavales igual que en Brasil, pero que no habían televisado

A: Claro

B: Entonces, me mostraba imágenes y yo decía, pero si el carnaval la gente está… por qué ustedes, como comunidad haitiana

A: No lo hacen acá

B: No piden un día y lo hacen acá y muestran su cultura po. Pa’ nosotros saber que a ustedes les gusta bailar y nosotros también empezar a incorporar como los mismos, como el ejemplo que siempre digo de las colombianas que las uñas, que el peruano la comida ¿cachay? Y que nosotros los vemos así como tristes, qué se yo. Y él me decía: “si allá va una banda arriba de un carro alegórico, nosotros hacemos las máscaras”, entonces por qué no ellos también tomarse un espacio y ellos también hacer una corporación de haitianos, si aquí el poder está en la comunidad po, mientras… no sé po, hay tantos venezolanos que también están todos

dispersos; si yo me junto con otro, con otro y con otro, podemos ayudar a otro hermano venezolano también po, y así vamos generando

A: Oye, y el tema de los venezolanos ¿cómo lo has visto también, muy desgranados?

B: ¿Cómo?

A: Como muy aparte uno del otro

B: Sí

A: ¿Cómo se da? ¿Cómo se observa eso?

B: Es que esto es en general, o sea, toda la gente extranjera obviamente viene con su familia y va armando sus redes, pero yo creo que también falta; si yo me encuentro con otro venezolano que esté en la misma condición mía, ayudémonos. Si después encuentro a otra familia, vamos ayudándonos y vamos entre todos haciendo comunidad. Pero acá en Chile ya se perdió eso ¿cachay?, como que están todos por su lado, qué se yo. Pero la gente extranjera tiene otro trato, tiene otra disposición

A: Ellos se ayudan más

B: Sí. O sea, deberían ayudarse más yo encuentro.

A: Pero no sé po, compararlo, yo sé que ya no es como la tendencia, pero los colombianos ¿cómo eran? ¿Se ayudan, más del grupo, más…? Una señora traía a otra…

B: El colombiano trae a la otra, y la dominicana trae a la otra dominicana

A: Ah, y la otra viene y como no cachan, y como que las van integrando

B: Sí, ellos la van integrando, sí

A: ¿Y el haitiano trae a otro?

B: El haitiano sí, pero a su familia que llegó recién po, a la prima, a la hermana, no sé qué, porque tiene que ver con este tema cultural porque ellos son más cerrados, que no hablan mucho, que las mujeres tienen que recién aprender español después de un año pa' que no hablen con nadie porque vienen de un régimen también, un Haití de pobreza… son todos muy como clanes

A: Claro. Oye, y esto como que la dominicana trae a la otra

B: Sí, trae a la otra

A: ¿Es clásico?, ya, a la vecina… oye, pero ¿tú relacionas como…, y esto implica que vengan más informados?

B: Sí

A: Como que (no se entiende 06:30) ¿cuáles suelen ser los más perdidos?

B: ¿Quiénes son los más perdidos?

A: Los que dan más vueltas en el sistema

B: O sea, hoy en día no hay tanta gente perdida, pero yo creo que la información se va modificando de boca en boca, te van diciendo… la haitiana en un momento viene a pedir su casa gratis porque pensaba que le iban a dar casa

A: Ya, más o menos

B: Y se va modificando porque es una ayuda, nosotros los orientamos, qué se yo. Pero yo creo que la gente que más cuesta que entienda el sistema son los bolivianos, que yo creo que son más indígenas, les cuesta, hay que explicarles las cosas como cinco veces: "sí, ¿me entiende?", yo creo que hay gente que habla quechua, yo me imagino porque procesan primero la información: "¿ah? ¿ah?", y ahí vamos explicando

A: Y tú les dices: "¿entiende?"

B: No po, así, yo creo, porque la Maira también como que lo cachó, obviamente la Maira lleva diez años trabajando acá, entonces dijo: "¿usted habla quechua? – Sí. Ah ya, entonces le voy a hablar más lento para que usted procese bien su…"

A: Sí, o para que vaya traduciendo

B: Sí po. Pero no, los haitianos igual son perdidos, es que son barreras po, la barrera del idioma, *heavy*

A: ¿Los venezolanos?

B: No, los venezolanos… es que por eso te digo, ellos son profesionales y están atentos a las cosas

A: Ya po, gracias.

REFUGIDOS Y SOLICITANTES DE ASILO

PERU

PP1-13

Antes de comenzar la grabación:

Comenta que ella por su trabajo ya no es Fonasa, y debe operarse el ojo pero no confía en las clínicas.

I: Me contaba que pertenecía a varias organizaciones, como la de refugiados peruanos ¿desde cuándo participa en ella?

P: Ahora más o menos como cuatro años, casi desde que llegue. Bueno yo cuando llegue no conocía a nadie, y ya cuando llegue y estuve acá fui conociendo a los peruanos que habían, más que nada porque la vicaría prestaba un servicio a los refugiados, y a mí me recomendaron que fuera a la vicaría como extranjera y como solicitante de refugio. Que fuera a la vicaría para que ahí me orienten. Entonces en la vicaría me daban nombres de otros peruanos que también eran refugiados. Es así que en las reuniones de la vicaría me encontré con otros peruanos que estaban refugiados y que iban a la vicaría, y por ahí fui relacionando. Con colombianos también en la época que yo llegue había mucho colombiano.

I: ¿Hace cuatro años atrás?

P: Si. Más que peruanos colombianos.

I: Estábamos en el dos mil catorce. Y había más colombiano me dice.

P: Más que hace cuatro años, sipo, seis años. Si yo ya cuatro años ya estoy en mi trabajo.

I: ¿y cómo fue? , ¿Venia del mismo Perú?

P: Si vine de frente. Es que cundo yo salí de Perú tuve que salir muy rápido. Porque el problema que se creó judicial en mi caso, la persecución judicial que yo tenía era que si yo no salía me detenían, así de simple.

I: ¿Salió usted sola?

P: Desesperada.

I: Con lo puesto.

P: Claro, con lo minino. Porque mis abogados me avisaron, mi abogado de ese momento me aviso y me dijo sabes que tu caso va mal. Y estábamos esperando que la suprema se pronunciara y él me dijo mira la suprema eran cuatro vocales y me dijo ya sabemos que por lo menos tres van a botar en contra tuyo. Entonces yo ya estaba trabajando cuando paso eso, entonces ellos me llamaron y me dijeron no vuelvas a tu casa ándate no más. Entonces yo tuve que llamar a mi casa y decir sabe que prepáreme una maleta con lo mínimo a si de cosas, como decir una mochila y me voy a si salí.

I: ¿Sola?

P: Sola, más bien por una casualidad yo me viene, ¿en qué me vine? En bus por una causalidad en ese bus viajaba una peruana que venía a chile ya para devolverse y erra refugiada y ella venia digamos ya en afán de regresarse ya a vivir a Perú. Porque ya su caso ya había terminado, su problema judicial ya había terminado. Entonces yo ahí en el bus yo la conocí prácticamente conversamos y ella lo que me recomendó anda a la iglesia porque halla la vicaría te va apoyar. Y ella ya se volvía a Perú y se volvió definitivamente y se volvió ósea estuvo dos tres días y fue la única persona peruana que yo llegue con ella. Ella me llevo a una casa y me dijo yo acá yo conozco a este peruano es refugiado te puede ayudar y por lo menos te va tener ahí un tiempo corto no. Y bueno estuve yo ahí en esa casa que era de gente peruana pero que yo no conocía era peruano y también eran refugiado. Pero para esto cuando iba venir acá a Chile yo había conocido cuando estuve haciendo en Europa haciendo una pasantía, bueno una diplomatura por mi trabajo la hice en España y ahí conocí a un chileno. Y yo al chileno le comente mi caso no, es que mi caso ha sido de persecución política siempre, no po yo como médico en algún momento atendí a enfermos que estaban involucrado en la guerra interna que hubo en Perú, y en Perú bastaba que de alguna manera hubiera atendido a una persona que estaba implicada en la guerra para que ya te acusen que tú eres uno más. Entonces a mí me acusaron de eso y eso en Perú era ser terrorista. Entonces yo siendo medico resulte siendo terrorista cuando yo nunca había matado a nadie ni había peleado con nadie. Yo lo único que hice fue currar nada más a enfermos. Y así en Perú han acusado a abogados que también defendían, también por defender a un acusado de terrorismo de haber participado en la guerra también terminaba siendo terrorista y lo detuvieron ose habían presos profesores, enfermeras, médicos, abogados. Mucha gente fue presa en ese tiempo y a mí me abrieron un proceso por eso no. Entonces es por eso que cuando yo salgo es que estuve detenida, mi caso subió a la corte interamericana y la corte interamericana lo sentencio al estado peruano y dijo cómo es posible que tuvieran a un médico preso por atender enfermos eso no está prohibido en ningún lugar está penado. Entonces el estado peruano me tuvo que pagar una indemnización por haberme detenido por ese problema.

I: ¿Entonces en teoría usted puede volver hoy en día?

P: No... No porque eso fue una decisión de la corte, la corte hubo un juicio en la corte interamericana cuando yo estaba detenida mi familia llevo el caso a la corte, y el juicio fue favorable es por ese motivo que el estado peruano me tuvo que dejar libre. Pero ellos ósea el primer juicio que me hicieron lo anularon. Pero me abrieron otro proceso ya no por curar enfermos ahí me acusaron por pertenecía te das cuenta, por pertenecía a organizaciones terroristas. Entonces ya con ese segundo proceso la acusación al final, la corte como se llama el primer nivel de justicia como no había ninguna prueba de lo que ellos decían que yo partencia a alguna organización pero ahora la curación no lo tomaban ya ellos porque ya era el segundo proceso. En ese segundo proceso como ya nadie me acusaba y justo vino la sentencia de la corte contra el estado peruano ellos me tuvieron que dejar libre por que no había pruebas. Pero ese proceso siguió como cuatro años y cuando termine yo estaba trabajando de nuevo como me dejaron libre yo me regrese a mi trabajo a mi casa todo normal. Si yo no tenía por qué ocultarme si yo estaba… yo no había hecho nada que sea punible no. Ósea mi vida había sido totalmente trasparente entonces volví a todas mis actividades de antes, me costó volver a mi trabajo si po

pero me repusieron porque la corte interamericana había dado esa orden y dentro de su sentencia decía que me reponga el estado peruano donde yo estaba trabajando, que me cuenten los años de servicios y todos los años que yo estuve presa que me los devuelva como si yo hubiera trabajado. Ósea la sentencia fue muy buena.

I: Usted estuvo presa.

P: ¡Sipo!, me detuvieron no te digo que me acusaron de terrorista por haber curado enfermos, nunca se demostró nada pero igual me acusaron. Y bueno ese juicio fuese sin rostro y todo, entonces todo eso fue anulado y la sentencia de la corte fue que me devolvieran eee prácticamente que me paguen una indemnización por todos los años que estuve detenida, que me reponga a mi trabajo, que me consideren como si hubiera trabajado, que me dieran una capacitación porque de todas maneras yo había estado años sin trabajar no. Entonces todo eso muy buena era la sentencia… muy buena tú la puedes ver esta hay en los casos de la corte interamericana. Pero por eso salí pero estaba todavía en el segundo proceso al final la corte el primer nivel de justicia que es la corte penal de terrorismo que le llaman me… en ese caso me puso que yo la sentencia compurgada que quiere decir eso que los años que yo estuve eran los años que tenía que estar ósea de todas maneras estaba castigada por terrorismo ósea que ellos dijeron le damos la pena compurgada que quiere decir es que eso aquí ya termina, usted se va ya en libertad pero los años que usted estuvo eran los que merecían estar ya. Entonces eso fue ya después de cuatro años, te estoy hablando que el juicio siguió cuatro años yo en libertad.

P: La sentencia de la Corte Interamericana ya se había dado en contra del Estado peruano, pero en sus cuatros años ya yo estaba trabajando me vuelvo integrar a mi vida. Pero el juicio no terminaba era largo que lo anulaban alaban que lo volvían abrir ¡ahí me cambiaron la acusación fiscal po! Y ya no fue por colaborar porque así me habían acusado primero, por colaborar por curar enfermos. Esa era mi primer acusación por eso me detuvieron pero ahora ya no fue por eso, ahora fue porque pertenecía según ellos, la acusación fiscal fue por pertenencia. Pero eso tampoco lo pudieron probar. Entonces como un no hubo ninguna prueba, Entonces ahí es cuando la jaeza me dijo Bueno usted ya váyase, pero ya había salido la sentencia la corte cuando la jueza me dijo: Bueno usted pida su convalecencia, me dijo porque a usted nadie la acusa, Entonces yo por eso pedí mi convalecencia y salí. Bueno y la sentencia de la corte ya estaba ahí. Pero bueno este proceso duró 4 años más cuando se da la sentencia y esta sentencia el segundo proceso da la sentencia Y esa sentencia fue pena compurgada. Entonces yo apele porque yo no merecía estar ningún día, Entonces yo apele ¿y para que apele? Ahí la suprema decidió y demoró como 2 años en decidir y ahí decidió la suprema Cómo era un caso de terrorismo y la ley antiterrorista dice que mínimo 20 años ¿porque me habían dado solamente los que estuve yo? .yo estuve de 8 años no fue poco. O sea me faltaba cumplir 12 años. Porque eso es lo mínimo que se da en terrorismo. Eso dijo la suprema y una vez que dijo eso entonces ahí me tuve que ir po.

P: ir porque yo dije no voy a estar ni un día más presa, no po. Tuve que dejar mi trabajo, dejar mi familia, dejar todo y salir corriendo. Por eso mis abogados me dijeron: sabe que va a salir en contra la sentencia de la Corte Suprema. Ellos ya están metidos ahí ya salían que habían escuchado eso. Entonces usted tiene que irse ahorita porque si no la toman otra vez presa. Y yo dije no po otra vez La pesadilla no puede ser y por eso me vine. Así sin pensar donde irme ni nada.

P: Y sólo chile tenía la posibilidad de venirme solo con mi DNI. Porque no tuve tiempo de hacer trámites visa nada pues. Pero él los 4 años que yo estuve libre Qué hubo la convalecencia de que te digo una vez que me dieron la pena compurgada hubo como dos caños que la suprema no daba la sentencia. No anulada la sentencia que me había dado la superior la corte Superior. Bueno en este tiempo yo me fui a España hacer una diplomatura. Que estaba de acuerdo a las necesidades de trabajo. Porque a mí me han puesto en el sector del adulto mayor y yo antes había hecho pediatría. Te das cuenta o sea para fastidiarme en mi trabajo no me pusieron a pediatría me mandaron al adulto mayor. Al otro extremo de la vida Todo diferente (se ríe).

I: ¿y eso lo había hecho el estado?

P: Claro el estado. Pero no la vuelta cuando me repuso el estado acepto reponerme pero no me puso a pediatría, ni siquiera a medicina general me mandó al adulto mayor Qué es casi como usted un geriatra y yo nunca le he hecho a geriatría. Entonces yo misma decidí yo me voy a acabar citar en esto porque o si no voy a ser cualquier cosa acá. Entonces por eso hice mi pasantía Y esa pasantía la pague con lo que el estado me indemnizo por haberme detenido al tiempo que estuve detenida. Estuve siete meses en Barcelona en la Universidad Autónoma haciendo este curso de diplomatura del envejecimiento muy bueno excelentes profesores. Yo estuve muy o sea disfrute de su curso aparte que a mí me gusta estudiar.

Interrupción por cierre de caja.

P: yo creo que una vez lo dice el que quiera me hubiese imaginado nunca que iba a estar en Chile jamás en toda mi vida.

I: ¡y en qué parte viva en lima?

P: el Lima en surquillo.

I: bonito Lima.

P:¿en qué partes has estado tú?

I: Miraflores te recorrí estuve también en…

P: ¿la Molina?

I: ¿La Molina como el sector de bares?, las ruinas de huanchaco me perdí el que también hay ruinas están en medio de la ciudad.

P: si si si por Miraflores no po

I: fui al callao a ver El Fuerte.

P: el real Felipe Ese es el cómo se llama esto en mi castillo real Felipe.

I: bueno la plaza al centro que más po ¿Que más po? algunos museo que se me están olvidando donde quedaban.

P: ¿fuiste a la santa inquisición?

I: No no fui Me faltó. Después me encontré con una amiga que era sociólogo y me contó que era muy bonita la Universidad Católica de Perú, que tenía Parque y todo.

P: Claro. San Marcos conoces.

I: ¿San Marcos universidad?

P: sí la ciudad universitaria.

I: no también me faltó.

P: te faltaron muchas cosas. Hay grandes universidades haya, ahí está en la universidad de ingeniería, muy prestigiosas. Muy difícil de ingresar en San Marcos, La Católica. Y después hay otras particulares: Ricardo Palma, la pacífica del Sur, ahí estudio mi hijo. Universidades chicas pero particulares. Las nacionales más grandes en Lima: San Marcos, después está la agraria que es de la Molina, La de ingeniería que queda por la zona norte de Lima saliendo por la Panamericana Norte del cono Norte. Después particulares está Santa Anita ahora y varias.

I: ¿y usted en cuál estudio?

P: yo estudie en Cayetana, particular también. Lo que pasa es que San Marcos es una universidad muy buena pero está recontra politizada, y ha habido y hay cualquier cantidad es movimientos estudiantiles fuertísimos entonces hay huelgas paros y todos esas cosas. Entonces la carrera que dura 8 años dura 10 años.

I: ¿cómo la chile?

P: ¿así es la chile también?

I: no tanto.

P: no porque por ejemplo San Marcos cuando hubo la época de la guerra interna. Bastaba que uno fuera San Marquino para que le digan que era terrorista. Tú dices San Marcos ya arriba pa dentro. O sea si encontraban algún San Marquino en alguna movilización era terrorista. Imagínate Entonces cuando ya en la época de Fujimori en San Marcos metieron un pelotón de soldados. O sea, se volvió un reducto y no soldados en la puerta no dejaban entrar a nadie. Tú tenías que identificarte con carnet de estudiante para entrar. O sea ellos impidieron la entrada mi mente de cualquier persona ajena a la universidad. Antes por la universidad entrada y salida a todo el mundo o sea se había una huelga de Mineros entraban los Mineros ahí acampaban en la universidad. Ahora ya no entraba nadie. Después del... y ha estado años militarizada. Hasta hace poco han levantado eso hace poco.

I: ¿seguía militarizada?

P: militarizada o sea manejada por no te digo un Batallón viviendo ahí. Un batallón de soldados ósea y habían hecho tu reducto en este cómo se llama a esto su campamento y ahí todos los días qué terrible, custodiaban. Porque es grande la ciudad universitaria cada pabellón, cada facultad estaba custodiada por sus cuatro costados por soldados. Terrible años duro eso años. Yo diría hasta como el 2004 Que yo recuerde. Militarizada y nadie podía decir nada. Y los estudiantes tenían que ir bien derechitos ahí no sé permitía nada. Una vez que se des militarista empezaron los movimientos de nuevo.

I: ¿Y cómo fue tu proceso ya acá en Chile? ¿Se acogió a la vicaría?

P: pila me dio servicio psicológico, me apoyó económicamente me daba una cantidad mensual por unos meses; lo mínimo para sobrevivir para el pasar Cómo decir una propina.me apoyó mucho. La verdad es que yo le tengo tanto agradecimiento. Aparte que había reuniones donde reunión a todos los refugiados, y lo último que me dio fue un curso. En ese tiempo yo ya estaba trabajando yo cuidaba enfermos porque no estaba convalidada, entonces no podía ser médico. Entonces cuide enfermos como 2 años.

P: Entonces creo que en el segundo año hubo el curso ese de... el general del Refugio fue el último pero fue muy bueno. Con había todo un equipo de asistentes sociales, yo diría sociólogos también, que ellos nos dieron varias clases un curso que dura varios meses, creo que había que

ir dos veces a la semana y ahí no sean como una psicoterapia. En la cual donde a uno le hace empoderarse de lo que es. No o sea recuperar la autoestima. Porque uno llega acá no conoce a nadie, sin familia, la gente te mira mal porque dice a no estés de otro país peruano, o sea y discriminación y si no Uno no es Rubio no te aceptan, una cosa es tía y xenofobia, claro no es la mayoría pero hay gente así. Entonces a uno le duele esas cosas y yo andaba muy triste. Más deprimida yo estaba trabajando en algo que yo no me había preparado yo era médico y que hacía trabajando de auxiliar de técnico no. Y bueno si bien el sueldo no era tan bajo pero igual no me sentía bien. Entonces ahí ellos me dieron toda la Digamos como se dice la fuerza no para que yo me empieza a darme cuenta que caramba si he pasado tanta desgracia en mi vida y he sabido afrontar. Por ejemplo la misma carrera de Medicina no fue nada fácil No es difícil y he sabido salir adelante o sea uno... y todo con dibujos con pinturas bien bonito a muy bonito el curso.

I: ¿dónde lo impartían?

P: En la iglesia o sea en la vicaría. Muy bueno muy bueno excelente yo siempre he pensado que eso fue mi mejor terapia y con eso yo me sentí mejor me di cuenta que Caramba de verdad po ósea Yo tengo un potencial ¿y porque voy a estar deprimida? Entonces no y ellos me pusieron dentro de tus deseos cuál sería que no sé qué y yo puse pues: volver a trabajar como médico. ¿Cuál es tu mayor ambición acá en Chile? Volver a trabajar como médico, tener un departamentito vivir en un lugar donde yo pueda recibir a mi familia cuando venga, porque yo vivía en un cuarto y para mí tener un departamento era como un sueño. Pero era algo que yo quería hacerlo o sea mis metas inmediatas, Y bueno pues que venga mi hijo a vivir conmigo o mi hija. Pero no mi hija porque ya estaba en Europa, por lo menos mi hijo que estaba en Lima y yo no quería que se hieran allá solo que venga acá para qué acá estemos juntos no.

I: Perdón ¿Y cuántos hijos tiene?

P: tengo dos

I: Una que estaba en Europa

P: Sí y el otro está en Perú. Porque en Europa está el papá de mis hijos que también está exiliado allá.

I: antes que usted se fuera

P: mucho antes. Él estuvo como haber 2000 como 10 años antes. Yo me o sea yo me quedé con mis hijos años.

I: A ¿sola?

P: bueno vivía con mi mamá en casa de mis padres porque mi papá falleció. Entonteces yo me fui a vivir con mi mamá y me quedé con ella. Ahí fue que a él lo detuvieron después se tuvo que ir a Alemania, está en Alemania hasta ahora ya como 20 años más. Y cuando mis hijos Cuando yo estuve detenida llevaron a mis hijos pues ya la familia decidió que fueran con el papá. Porque él le podía dar allá por lo menos la educación que yo no podía darles pues. Si estaba presa Entonces se fueron los chicos pa ya. Pero cuando yo salí mi hijo regresó a Perú en cambio Ya mi hija no podía porque ya estaba en la universidad Entonces ella ya siguió y ha seguido allá a esta hora pero mi hijo está en Perú. O sea, vino Perú porque él recién terminaba su colegio allá en el Alemania, ingeniero 16 18 años tenía, no 16 y me faltaba completar algunos cursos que de allá pero le convalidaron en Perú entonces le faltó como un año y con esto terminó el colegio. Pero estuvo conmigo cuando yo salí pues o sea yo salí y justo el vino a

los meses y justo Yo decía cómo lo voy a mantener a este chico. Pero justo ahí ya me integraron al trabajo.

I: lo de geriatría

P: Empecé a trabajar y me reintegraron a mi trabajo y con eso ya lo mantenía. Pero aun así Los sueldos en Perú son muy bajos la verdad es que son muy bajos hasta ahora es que verdad hasta ahora está así. Y prácticamente los médicos es un trabajo más ganan poco y para pagarle una universidad particular a mi hijo se me iba casi todo el sueldo en eso no, así que tenía que hacer horas extras y todo para poder manejar mi casa no pero bueno en realidad yo no pensé nunca en venir a Chile la verdad nunca. si no hubiera sido porque tuve que salir así prácticamente con lo que tenía puesto y claro yo plante a mi hijo porque yo lo vi después a él me encontré con él y le dije mira hijo pasa esto no las cosas salieron en contra judicialmente Y si yo no me voy de acá me van a detener, Pero si tú quieres me lo pides y tú me dice Quédate mamá porque si quieres que yo me quedé y vea lo que pase no sé Lomas probable es que me detengan yo me quedo le dije. Y él me dijo: no mamá ándate no te quiero ver más una vez más detenida, porque él sufrió mucho cuando yo estuve detenida me dijo ándate. Entonces cuando él me dijo ya sí Entonces yo ya dije ya voy a hacer todo lo posible para que no te falte la economía. Por eso yo acá tenía que trabajar de lo que sea para poderle mandar a él porque él no tenía de otra persona.

I: no hay más familia alla

P: sí Pero mi mamá anciana ya estaba muy anciana mi madre. Mi madre Necesitaba una persona que en la tienda más bien. Y yo también desde acá tenía que mandar pa ya. Porque mis otros hermanos no están en buena situación lo que están en Lima por lo menos, hay otro que está en el norte que también es médico. Pero los médicos tampoco se están en tan buena en situación. En Perú el médico no es como acá, que por lo menos tienen un sueldo más o menos digno que uno puede vivir tranquilo. Hallan el médico que tiene dos hijos el universidad ya está arruinado. Porque necesitan los recursos para poder mantenerlo y mi hermano mayor mi hermano que es médico trabaja en el norte tiene cuatro hijos. Imagínate.

I: Fuerte. Coma una señora María Teresa

P: fuerte. Un psiquiatra acá el que me atendía pro la…a ver después que cerró la vicaría porque yo estaba en tratamiento psiquiátrico porque me daban para la depresión para el problema del él e estrés postraumático habían unos psiquiatras de la Universidad em.

I: ¿de Chile?

P: no de la universidad a ver...

I: ¿USACH?

P: no no no Alberto Hurtado.

I: ya

P: ahí crearon una clínica psicológica para refugiados y entonces del como a mí me atendían de la vicaría y ya cerraron esa tensión. A mí me mandaron para la clínica de refugiados pero también ha funcionado un tiempo pero ya después se la cerraron. En este tiempo me atendió. Un psiquiatra y una psicóloga, la psicóloga buenísima excelente y el psiquiatra este que me atendió ahí y escuchó toda mi narración, Él me dijo Cómo ha podido soportar tanto (se ríe) él se sorprendía que yo pudiera estar tranquila trabajando digamos y no me hubiera dado un

patatús pues no con todo lo que había pasado él se sorprendía y me decía que caramba con todo lo que a usted le ha pasado lo que tienes poco para lo que podría tener

I: Y eso mismo como que la levantada usted

P: sí.

P: bueno así fue la cosa. Ahora el refugio cuando yo solicité el refugio me lo dieron como 3 años después ¡mucho! Tiempo mi caso un caso de la Corte Interamericana. Por qué la corte después de que me dieron esa... anulo mi segunda sentencia en Perú, bueno en la suprema de Perú dio el fallo contra mío y dijeron que yo no podía estar solamente el tiempo que me habían dado sino que serías tan mínimo 20 años Presa. entonces me siguieron la pena y esa segunda sentencia mi abogada que llevó mi caso a la Corte Interamericana la presencia la Corte Interamericana con una especie de cumplimiento de Sentencia y la Corte Interamericana vieron este según proceso Bueno yo ya estaba en Chile cuando ya pasó todo eso. Y cuando la vieron dijeron que otra vez la corte peruana en Perú había cometido un error conmigo. Porque me estaba aplicando leyes antiterroristas que fueron mucho Después de los supuestos hechos de curación que yo había hecho, porque ellos no me podían acusar de nada. Me decían pertenencia pero para ellos la prueba de eso era que yo había curado que eso era la prueba que yo pertenecía. Pero esas curaciones eran antes del 2000 mucho antes que 90 no. porque terminó la guerra el año 1992 y ahí hicieron la ley antiterrorista y los hechos son del 89 de por ahí mucho antes que salga la ley antiterrorista como me iban a aplicado retrospectivamente a mí y acusaron a mí con esta ley. Que esta ley la pena mínima de 20 años entonces ahí cometió el estado peruano otro error. Entonces la Corte Interamericana le ordenó al Estado peruano: sabe que ustedes han vuelto a equivocar y tienen que anular ese proceso. Y el estado peruano dijo ya lo vamos a anular, los vamos a animar pero la vamos a jugar nuevamente con las leyes antiguas. Entonces mi caso no sea cerrado en Perú sigue y me llaman para un tercer proceso por lo mismo

I: un tercer proceso.

P: y por lo mismo, por los mismos hechos por lo mismo no hay nada nuevo. o sea es una persecución no acaba nunca. Además que Ellos están dolidos porque me han tenido que pagar la indemnización. Y lo otro es que ni defensa me pide siempre la Corte Interamericana que cumplan la sentencia no lo han cumplido. Cumplieron darme la indemnización pero eran como 5 puntos y no han cumplido pues: no me han dado capacitación, no me han dado atención médica como decía ahí en esto, no me han dado la cuestión del trabajo porque en mi trabajo no me querían reconocer los años que yo estuve detenida, no me lo quisieron reconocer nunca hasta ahora. Solamente los años que he trabajado menos han reconocido Y eso suma como 20 años. Pero hay como 8 años que están vacíos eso ellos no lo Quieren reconocer porque dicen no los ha trabajado y no los ha trabajado. Pero la corte ordenó que se reconozcan como trabajados. Entonces son varios puntos que no han cumplido el estado.

I: y a pesar de todos esos documentos.

P: y luego que dice el estado pero como es que ella está en proceso ella está llamada proceso A lo mejor en el proceso sale una sentencia y ya no tenemos que cumplir con nada de eso. Te das cuenta o sea ellos ponen de pretexto el estado peruano pone de pretexto para no cumplir la sentencia de la Corte Interamericana cabalidad: el decir que como no ha terminado mi caso no está cerrado entonces puede ser que cuando yo me presente e me sentencien. Mejor dicho Es que ya me están esperando con la sentencia lista eso es la verdad porque no quieren cumplir todo lo que me deben: me deben capacitación, miden los 8 años que supuestamente no los

trabajé y que ellos me tienen que reintegrar todo eso no lo quieres pagar el estado peruano por eso no le conviene terminar mi caso. Ahora Ellos dicen pero que venga la estamos esperando que venga pero apenas llegué la vamos a detener. Porque ella es una reo ausente. No ves que ni caso estaría nublado. El proceso judicial sea nulo pero no la detención. Entonces yo debería estar detenido hasta que se resuelva el caso. Imagínate Cuántos años estaría detenida en Perú.

I: infinito Pues no sé

P: No, pero ya se equivocaron 2 veces no voy a ir para que se vuelvan a equivocar no voy. Pero eso a mí me causó una tremenda pena: no voy a volver a poder a volver a mi patria, no poder estar con mi familia mis familiares. Pero ya me estoy adaptando a Chile mira que estoy comiendo comida Chile.

I: un chacarero pidió.

P: pero no es a .porque no me venía con porotos.

I: no, venía con porotos, ¿Pero qué clase chacarero viene sin porotos?, ¿y su mamá sigue ya allá?

P: ya murió, porque era muy mayor ella falleció el año pasado en marzo y no pude ir po no puedo ir a Perú. Y apenas pise tierra peruana me cargan. Y ojo que haya Ojalá no es procesos de terrorismo fueran rápidos duran años. Así que uno no sabe cuándo van a terminar y el segundo duro como 6 años.

I: ¿y durante el proceso seguiría en la cárcel?

P: claro tiene que estar preso.

I: Perdóname si es muy tonto lo que me decir: está en algo como punta peuco o cómo una colina 1 así como pensando en Chile.

P: punta peuco no hay como para casos de terrorismo, estaba en una prisión de altísima seguridad no te permiten que ni entre una guitarra, ni un instrumento musical, ni un libro.

I: ¿Nada? aislada total.

P: ahorita a los presos políticos que están desde la época del 90 tienen como 25 años presos y no les dejan pasar nada es tan sumamente afligidos. No les dejan pasar nada ósea es como la muerte en vida.

I: y usted estuvo 8 años así.

P: 8 años. Los primeros años con un aislamiento absoluto porque no nos dejaban ni tocar a la visita, no te dejaban nada más que te visita la mamá el papá el esposo o los hijos nadie más. Ningún amigo ningún primo pariente no. Ya cuando nosotros hicimos unas huelgas de hambre hemos hecho en prisión para qué te flexibilice las condiciones. Pero ahora otra vez se la han vuelto a poner bien rígidas. O sea para el Preso por terrorismo en Perú la aplican el derecho penal del enemigo. Qué quiere decir que para ellos el terrorista.

I: ¿es traición a la patria?

P: es una no persona, Qué quiere decir que no tiene Derechos Humanos. Entonces ellos tratan cómo les da su gana, es decir los encierran yo estuve en un encierro en el cuento la media hora de patio. En todo el día y te sacaban a la hora que ellos querían. O sea ni siquiera era la misma hora siempre sino que de repente a las 6 de la mañana, de repente a las 4 de la tarde. Estaban prohibidos los relojes, en lapicero estaba prohibido no podían escribir.

I: ¿no podía hacer nada?

P: no, y en ese tiempo jugábamos ajedrez de celda y con peones Y cómo se llama y todos los del ajedrez todas las figuras del ajedrez hechas con migas de pan, porque no te dejaban pasar.

I: ¿Pero habían barrotes y entre los barrotes y había espacio?

P: No tú no veías al otro. Eran filas así y todos estaban en un solo sentido las celdas. Y sólo a la hora del patio te dejaban encontrarte con la gente de tu ala. Tú no te veías con los demás sólo sacaban a esa ala. Media hora esa ala que tenía como 20 presos. Que eran como 8 celdas de a 2, de a 3, de a 4 a veces. Pero ese grupo está el día y no te dejaban que te juntes O sea tú tenías a máximo conversar de a dos. Pero la policía estaba parada ahí todo el tiempo y te decía no conversé, Sepárense, No nos dejaban ni conversar en grupo, a lo más se podría jugar voleibol. Eso aprendí a ser yo.

I: a jugar voleibol.

P: te cuento que yo empecé a hacer cosas que no hacía antes: por ejemplo yo salía y apenas me abrían la reja y me ponía a correr en el patio me daba vueltas y vueltas en el patio muchas vueltas en el patio. Es que la celda era chiquitita, era dos y medio por dos y medio. Mamá y a y a veces habíamos 3 personas ahí.

I : Tres

P: y ahí también en un pedacito estaba el baño en un costadito.

I: ¿y eran todos presos políticos?

P: Si po. Y más encima había que bañarse ahí, hacer todas tus necesidades era terrible, ósea no podías hacer en cualquier momento porque estaba otro A lo mejor comiendo era bien bravo. Entonces la primera oportunidad que había de salir. Por ejemplo había un taller de flores de repente ya yo me inscribía, cualquier taller qué hubiera yo me inscribía. Y eso me aconsejaron las chicas que estaban ahí, me decían Aunque no te guste sal porque es una manera de salir. Te romper este si era un pedacito hasta acá nomás sería.

I: Muy chiquitito

P: y las camas eran de cemento, te ponían un colchón de espuma y la tarima era de cemento todo era de cemento. Qué terrible era y te pasaba la comida por el piso y era una comida mala. Yo no sé cómo la verdad no soportaba todo eso. Bueno después del 2000 abrieron las rejas cuando salió el Fujimori y ahí ya podíamos estar afuera, pero antes del 2000 el encierro era total y ahí se hizo huelga de hambre mucha huelga de hambre se hizo.

I: ¿y usted participó de la huelga de hambre?

P: sí que me quedaba. En ese momento cuando me detuvieron la primera vez el juicio sin rostro, los juicios porque ellos tenían que tener juicios para justificar que estabas adentro. Cuando tus juicios eran rápidos y de frente 20 30 años te aplicaban así. Porque ya a mí me detuvieron el 96 y el 92 fue la ley antiterrorista entonces a todos aplicaron la ley antiterrorista. Aunque el caso no haya sido 92. Por eso es que la Corte Interamericana sentencia mi favor. Porque mis casos eran del 89 o sea, supuestamente la gente que yo había curado era de esa época, no era después del 90 ósea no había ley antiterrorista no me podían aplicar esta ley

pero me la aplicaron. Por eso me dieron 20 años po yo tenía 20 años para estar... la primera vez me dieron 20 años.

I: qué fuerte.

P: hasta ahorita hay presos en Perú.

I: los mismos compañeros suyos

P: claro detenidos Desde esa época y de antes. Gente que estaba detenida cuando yo llegue si ya detenida. Hay presos que ya tienen más de 25 años y se han cumplido sentencias y no los quieren dejar salir. Porque ellos te han sido dirigentes en la época de la guerra interna han tenido cargos políticos altos, ellos mismos han reconocido. Entonces están arruinados por qué no los quieren dejar salir Aunque ya hayan cumplido la sentencia de 25 años. A una pareja de presos los han metido 25 años más hasta ahora no lo quieren dejar salir les están inventando nuevos procesos, sobre los mismos hechos que ya fueron sentenciados e e incluso algunos hechos que son cuando ellos ya se estaban presos también de eso me están acusando. Imagínate cosas totalmente jalados de los pelos. Por eso se dice que en Perú aplican el derecho penal del enemigo, quiere decir que no es el derecho penal corriente digamos normal e internacional, sino que es ajustado a sus intereses políticos.

I: ¿eso se dice formalmente o entre la gente?

P: el derecho penal del enemigo existe

I: ¿institución es algo escrito?

P: Si po. Fue creado por los nazis en la época de Alemania porque crees que a los judíos los tuvieron como animales porque la aplicaron este derecho penal. Es un derecho penal nazi y es aplicado actualmente y no sólo ahora desde antes. O sea en el Perú desde los 80. Pero por qué crees que están los presos en Guantánamo algunos están sentenciados, y el derecho penal tiene fija una un término si tú estás detenido en tal terminó te tienen que sentenciar y si no si no te pueden acusar tienes que salir en libertad. Pero a los Guantánamo Qué son los que son los Afganistán cuántos años están presos desde el 2011 y algunos no están ni juzgados. ¿y cómo los tratan? como perros, peor que perros hasta perros los tratarían mejor. No humano po entonces eso se da. A los mapuches Qué les han hecho.

I: la ley antiterrorista.

P: este derecho penal del enemigo porque el enemigo es como decir no es un ser humano. Entonces no merece ningún derecho, pero si a esta señora mapuche en el hicieron en dar a luz en grillada, con el policía ahí trato inhumano total

I: Institucionalizado, como conversamos anteriormente.

P: Ese derecho penal del enemigo se usa en tiempo de guerra, actualmente se está aplicando en tiempo de no guerra ya es una expresión del endurecimiento el sistema penal pero me han entrado políticamente. Porqué las acusaciones ya ni siquiera son digamos hechos concretos sino son suposiciones, por ejemplo a estos dos que son dirigentes de la organización allá en Perú que dirigió la guerra y han estado 25 años no los quieren dejar salir y hay que ande usted Qué son impuestos son un peligro para la sociedad. Si sale en qué cosas podrán hacer, entonces mejor que no salgan entonces Hay que inventar cualquier cosa para que no salga.

I: Totalmente una maniobra política

P: Es una maniobra política por eso es el derecho penal utilizado políticamente por intereses de un grupo del grupo que está en el poder. O sea no se cumple la ley eso es que se van en contra de su propia institucionalidad porque se entiende que el derecho penal es un código reconocido internacionalmente. Sin embargo seda que no se cumple. Al pueblo mapuche no le aplican la ley cómo es tú has visto y se ha descubierto acá las crean le han creado pruebas falsas. La policía le ha creado prueba ¿cómo se llama ese operativo que hubo?

I: antorcha y todo eso testimonio en tortura condiciones muy infrahumanas.

P: pero no sólo eso le ha creado eso ¿cómo se llama esa?

I: Caso antorcha

P: no po hay otro último, se le descubrió que en la policía le había creado prueba informática con los celulares a los mapuches para demostrar que ellos habían hecho no sé qué quemas, hechos violentos allá en el sur y no era verdad. ¿Cómo se llama esta operación? ¿ Huracán?

I: huracán..

P: Huracán creo.

I: entonces acá presenta todos sus papeles de la Corte Interamericana como prueba me imagino ¿y a pesar de esto dura 4 años el proceso?

P: demora 3 años para que me den el refugio

I: el 2012 llega y presenta rápidamente me imagino

P: Si inmediatamente. No mira qué pasó yo el 2010 pero me regresé a Perú porque e... parece que la sala las vocales no sé porque los cambiaron, y dijeron que habían puesto otros vocales. Entonces había la posibilidad de que esa nueva sala me absolviera. Entonces por eso que yo me regresé a Perú. Porque ya había estado unos meses acá pero si me dijeron eso Hay una posibilidad de que usted sea absuelta. Entonces me regreso inmediatamente y me volví. Pero pasó unos meses y ojo que el trabajo me repuso porque habían pasado 3 meses no más yo había estado 3 meses acá. Entonces él 2011 A fines del 2010 en diciembre.

I: a ver si me estoy enchufando bien en la historia.

P: Yo pedí el refugio apenas llegué el 2010. Pero como en julio más o menos hubo la posibilidad de que proceso saliera bien, porque cambiaron a toda la sala suprema. Entonces mi abogado me dijo hay posibilidades que esto salga a favor porque creo que uno de ellos voto a favor no sé cómo fue pero la cosa es que eran como 5 y habían dos que se habían votado en contra, y habían posibilidades que los otros tres voten a favor no sé. Hubo un cambio pero la cosa que cambiaron a los vocales y entonces me dijeron parece que lo tuyo va a salir bueno. ¡Entonces dije hay me voy de mediatamente! y Bueno me fui a Perú yo para eso pedí permiso acá. Aparte mi mamá estaba delicada Entonces yo aproveché para ir. Porque les dije voy por la enfermedad de mi mamá y yo la verdad no pensaba quedarme tanto, yo le dije me voy a quedar un mes dos meses máximo. Pero cómo llegué y en mi trabajo me dijeron ya si tú vienes te podemos reponer no hay problema, me repusieron entonces yo dije Qué bueno que me repongan y volví a trabajar.

I: ¿volvió a Perú?

P: Si po en mi trabajo me aceptaron. Volví a trabajar y me olvide del asunto Refugio de acá pero todavía no salía la sentencia de la corte, hasta que en diciembre salió y ahí tuve que venirme al otro día. O sea me dijeron No no van a votar en contra entonces ahí tuve que venirme volando. Entonces en realidad es del 2011 yo llego y encuentro que en el refugio me habían denegado el refugio. Porque yo había pedido permiso para salir y no había vuelto.

I: Por medio del ¿DEM?

P: A todo el sistema de acá. O sea yo lo pedí a relaciones exteriores yo pedí permiso para viajar a Perú porque estaban solicitando refugio. Eso sí ahí mismo me respondieron esto salió a los meses salió que me denegaron el refugio. La primera vez que yo vine y me regresé a Perú ahí me salió volando la negación. Entonces yo cuando llegué, justo llegué y el mismo día fui corriendo porque yo no sabía que me habían denegado, Simplemente yo decía mí pasado de los días no que había quedado 2 meses y me pace 5 meses. Y cuando regresé ellos me dijeron apenas llegué Qué bueno que ha venido porque tenemos su respuesta firme que está recibiendo y me dieron la respuesta que está denegado. Tiene 48 horas y yo le dije no Pero si yo estoy volviendo porque mi situación estaba perón. Entonces me dijeron a tienen 48 horas para presentar una reconsideración. Entonces me fue ahí mismos en mi reconsideración y presente mi reconsideración y esta reconsideración me duró a mis 3 años que me respondan, como el 2014 me respondieron. Me aceptaron.

I: Con la ayuda esos años me imagino que con mi vicaria.

P: El primer año es normal la vicaría 1 año o 2 años quien sabe. No el primer año fue la vicaría, el segundo año no me acuerdo bien. Pero lo primero es año pero no fue más de 2. Porque en el resto ya Yo conseguí trabajo acá ya vino la Bachelet entró y ya vimos la posibilidad que los médicos extranjeros trabajen, empecé a trabajar en recoleta.

I : ¿hizo el eunacom?

P: claro

I: ¿cómo válido su título?

P: es que al comienzo yo no podía validarlo, yo cuando llegué Quise validarlo pero no me presente porque me fui a Perú. Al segundo año mi presente pero no me fue nada bien porque no me había preparado, Yo pensé que era fácil la cosa pero fue sumamente difícil. Entonces yo me decepcioné tanto eso también me golpeo no probarlo dije que pucha que mal estoy, no puedo trabajar así po definitivamente. Entonces yo estaba trabajando cuidando enfermos entonces Y además lo que yo ganaba me alcanzaba para mandar a Perú y Sobreviviré acá, pero también necesitaba para hacer un curso de preparación y ya yo me enteré que habían que hacer un curso para prepararse. Y ese curso cuesta como $800000 pesos entonces yo nunca iba a poder tener acceso a ese curso. Una vez que ya empecé a trabajar ahí tuve que ponerme las pilas y bueno ya me pagaban los suficientes como para poder hacer el curso ahí hice el curso.

I: Entonces empezó a trabajar y…

P: y después pude hacer el curso.

I: y después la prueba.

P: No pues yo llegando Ya di la prueba pero sin mayor preparación ahí me reprobaron. Ahí yo me decepcioné yo dije ya yo no he a poder nunca poder dar este examen así. Tengo que

prepararme llevando el curso no, no puedo dar así no más y trabajando más encima. Entonces dije yo ya estaba defraudada el curso Ya pensaba que no lo iba a dar. Pero se me presentó la oportunidad de trabajar en recoleta y ahí es donde yo dije Bueno ya si tengo un sueldo ahí puedo pagar el curso, y eso fue lo que hice pagué el curso y vuelve a dar en el examen. pero ya en esas condiciones Si no hubiese trabajado como médico nunca hubiera podido pagar ese curso así de simple.

COLOMBIA
PP 14-38

P: 11 años ya van a ser 12.
I: ¿usted llegó pidiendo solicitar refugio?
P: Llegamos como solicitantes de Refugio todo el grupo familiar mi esposo y 3 hijas.
I: y ¿Cómo fue ese proceso el proceso de solicitud de refugio?, ¿porque eligieron hacerlo le tuvieron que hacer?
P: Ya te presento la situación de tener que salir, por un tema de riesgo de la integridad yo particularmente no sabía nada de lo que era la figura del Refugio no sabía nada de eso, no sabía nada. Y cuando estábamos en el tema de buscar pasajes Y para dónde irnos de una agencia de viajes las vendió dijo mire. Porque nosotros le decíamos en la urgencia que teníamos de salir Entonces como los montos serán elevados porque éramos cinco personas. ¿Y por qué no se van como refugiados? Y porque nos vamos a ir como refugiados. No es que mire que existe eso que se llama refugio y yo sé que aquí hay gente que yo le vendido pasajes a personas que se van como refugiadas. Entonces yo le dije averigüe y me cuenta, entonces la mujer por Internet se averiguó y me mandó unos link. A todas estas nosotros estamos en un apartamento como un Hostal ahí sin salir Entonces era todo por teléfono y entonces ella me dijo que me mandaba al correo electrónico los link para que mirará. Entonces así que ahí miramos ella me mandó los datos me mandó hasta la dirección en esa época de la vicaría, entonces me dijo si ustedes van.
I: en Chile
P: Es que me dijo las opciones de refugios son en Europa pueden ir a España Francia no sé. Pero para poder ser refugiadas allá ustedes tienen que llegar y comprar un tiquete como turistas, y en el momento que lleguen ahí mismo pedir el refugio, pero si no les dan el refugio los devuelven. Y mientras le dan el refugio van a estar casi preso, o sea, metidos en un sitio mientras se investiga. Y nosotros no y aparte el tema del idioma si era Francia no sé qué, yo dije no ni loco como me voy a ir un en un avión con mis hijas y llegar y que me digan no devuélvase Y ahí sí qué terrible. Así que nos dijo otra opción mucha gente se va a Chile, porque en Chile Cómo hablan español si ustedes se van a refugiar allá dicen que chile es un país muy bueno no sé qué. Y me mandó todos los datos Así que yo me iré y en la página parecía la dirección de la vicaría. Entonces dije Cuando llegamos allá y nos vendieron un pasaje como turistas y en esa época era la vicaría la que acompañaba, la vicaría era la que hacía el acompañamiento a los solicitantes. Entonces llegamos a la vicaría y ahí no era porque habían cambiado de dirección ya era en el centro nos dieron una dirección de Yungay por allá de Brasil no sé.
I: sí sí.
P: Y llegamos acompañamiento consistía que la asistente social hacía como una entrevista con las personas y analizado el caso así como en forma general e inmediatamente le hacían llenar los formatos, y ellos esos son formatos los ordenaban cola documental in armaban un paquete a uno y le dejaron como todo listo vaya a extranjería y presente sé con esto.
I: Ah okay ellos…
P: Entonces uno iba extranjería y llevaba eso que ya llevaba como visto bueno de la vicaría y allá simplemente le llenaban el otro formato y le daban el maravilloso documento que nos daban

en esa época que era: una hojita que decía fulana de tal, o sea María Elena Osorio con documento colombiano número tanto se encuentra en de manera regular en el país y ya.
I: Era un papelito
P: Una hojita sin membrete del Ministerio de...
I: Sin sello
P: Nada ni del Ministerio del interior.
I: ¿y le daban RUT por ejemplo?
P: No absolutamente nada porque no había ley de refugio, si le daba refugio y no tenía ley de refugio. Entonces con uno con esa hojita iba por todos el mundo y todo el mundo se reía de uno, usted de dónde sacó esto (se ríe).
I: Claro como que usted hasta podría habérselo hecho.
P: Sí no sí varias veces me dijeron ustedes son muy ingeniosos hacen unas cosas. Quién le hizo esto y me daban unas ganas de todo y así estuvimos 22 meses más o menos. Y hay gente que estuvo 4 años con ese papelito.
I: y luego ahí en la vicaría le dieron orientación ¿Cómo se empezaron a orientar? en vivienda todo.
P: la vicaría, bueno nosotros fuimos como Afortunados entre comillas porque teníamos recursos, veníamos con un paquete turístico: Entonces nosotros íbamos a estar una semana, teníamos 8 días de hotel, teníamos 8 días de hotel con tour con todo así que nosotros fuimos a conocer viña, a los viñedos, esos tus normales, Nos sacaban del hotel y nos daban 10 vuelta estábamos en teatinos con huérfanos. Y el hombre no sacaba daba y vuelta en huérfanos cogían la costanera le daban no sé qué vueltas y luego nos traía la moneda.
(Risas de entrevistador y participante)
P: Qué lejos no, luego nos enteramos.
I: ¿y ellos los trataron como turista?
P: claro éramos turistas, pero nosotros al segundo día que llegamos ahí fuimos a la vicaría y empezamos el trámite. Entonces hicimos los tours. Y cuándo ya llegó que se iba a acabar el hotel, nos pensamos quedar en el hotel mientras. Porqué nosotros con lo de la hojita pensábamos que nos iban a resolver súper rápido, porque no nos dijeron más en la vicaría. Nos dijeron que teníamos que esperar porque luego nos iban a llamar a unas entrevistas y ahí iban a establecer si no daban el refugio no, pero que mientras tanto nosotros podíamos hacer lo que quisiéramos. Así que dijimos vamos a evaluar Cómo es el país y según eso nos quedamos o nos vamos para otro lado y miramos qué se hace. Pero bueno empezamos a mirar las calles y como era todo tan perfecto salíamos y dábamos la vuelta teníamos caminos por dónde íbamos y veníamos y nos parecía todo maravilloso. Pero yo empecé a mirar que el hotel valía mucha plata entonces yo dije lo poco que tenemos allá, las pelotas que nos estaban cobrando porque había gente que nos debía y mi hermana se encargaba de cobrar, y nos mandaba la plata pero eso era dividir por 4 o sea muy duro. Yo pensé yo decía Pero cuánto vale el hotel no esto es mucha plata, tantos dólares no sé qué. Dije nos tenemos que conseguir algo más cómodo: nos conseguimos un aparta hotel un apartamento en diagonal Paraguay era una cosa horrorosa qué se llama... No me acuerdo cómo se llama ahí en la calle quito en diagonal Paraguay, un departamento bien pero el ascensor era horroroso y también era caro. Entonces sí vamos allá Santa Isabel de Paraguay te diagonal Paraguay con Portugal y ahí cuando yo me puse a mirar los letreros que arrendaban; y decía departamento para turista amoblado 3 habitaciones. Y valía menos de lo

que podría valer una semana en un hotel. Ya le dije a mi marido mira Esto entonces llamamos al señor y el Señor nos dijo: venga véanlo. Y era y en carabineros de Chile. Así que fuimos lo miramos y el hombre nos dijo ustedes son turistas si es que no sé qué. ¿ Tienen $1000 dólares de garantía? Nosotros pues no hoy pero los conseguimos. Si se los consigue yo se lo arriendo. El departamento estaba recién amoblado porque el caballero recién había como organizado, había empezado el negocio entonces todo estaba nuevo. Así que hay arrendamos por un 4... Hicimos un contrato si hasta notar ya lo hicimos por 6 meses. Con los $1000 dólares de garantía.
I:¿ y ahí pedían visa?
P: a él no le importó porque como éramos turista y tenía los $1000 dólares.
I: Él no le hizo problema de la visa claro como tenía los $1000 dólares. Porque usted no tenían permiso para estar.
P: ahí no el tipo no le preocupo absolutamente nada. Con los $1000 dólares el feliz. Nosotros no le tomamos el peso a eso y estuvimos ahí marzo, abril, mayo y empezó junio y empieza hacer frío tan terrible, y pagamos el agua y fuimos a pagar el primer mes de agua y el lago eran como $150000 ¡en el 2007!. Entonces yo le dije a mi marido estamos pagando 500 mil pesos valía el apartamento 150 de agua ¡es mucha plata!
I: era el precio de turismo
P: y empecé a mirar en el periódico a ver Cuánto valía un arriendo, y ahí conseguimos una al apartamento que también corrimos con una suerte. Porque también le dijimos al Señor mire nosotros estamos pagando esto pero la verdad no podemos más y estamos dispuesto a darle la garantía para que nos arriende. Dijo si yo no tengo problema yo les arriendo. Y nos arrendo allá en parque Almagro, allá nos fuimos a vivir y con un mes de arriendo compramos los muebles de la casa. O sea con $500000 pesos compramos: las camas, compramos refrigerador, lavadora, lo que necesitaba la casa lo compramos. Pero nosotros fuimos Afortunados porque teníamos recursos, porque la vicaría nunca nos dijeron miré nosotros nos vamos a acompañar así, le vamos a dar esto aquello información de esto nada. No nos dieron nada y nosotros veníamos como nos ponían citas para ir y cuando íbamos a las citas veíamos: la gente salía con bolsas, la gente salía con colchonetas, Como con frazadas Cómo con cosas. Pero nosotros no intuíamos Y a nosotros nunca nadie nos dijo que nos podrían dar un tipo de ayuda. Nos preguntaron sí de nos estábamos quedando y claro como dijimos Cómo que en hotel y en condiciones que evidentemente esta gente tiene plata no les ofrezcamos nada. Bueno en ese momento nosotros estábamos haciendo el esfuerzo porque no sabíamos que existía nada, y en Colombia también entre toda la familia había juntado y nos mandaba y esto era tremendo. Así que nunca Ni siquiera bueno nosotros nos preocupó mucho la educación y ahí si nos dieron la orientación a medias pero no se las dieron.
I: Es que vinieron con hijas pequeñas ¿Cuántas son?
P: 3.
I: y qué edad tenían
P: venían de 8 10 y 12. Entonces mi hermana pronto nos mandó los certificados, pero nosotros a la semana siguiente estábamos preguntando eso de la escuela, nos mandaron al Ministerio de Educación y en el Ministerio de Educación nos dieron una lista de colegios. Pero nadie nos dijo Cómo funciona el sistema educativo acá, cómo era, el caso que forzamos el periplo por todos los liceos con letras para ver Donde había cupo y no nos recibían.
I: ¿y En qué período era del año?

P: llegamos en marzo, pero los cupos ya estaban cerrados, cerrado cerrado y luego conseguimos ya íbamos a matricular a la niña que a la mayor estábamos esperando ahí en la secretaría del colegio, cuando dos niñas estaban ahí en el mismo patio qué decían que habían robado que no sé qué. Salimos nos arrancamos. Finalmente escogimos tanto primero las públicas en las públicas no había para la mayor. Nos preocupaba más ella porque ella se estaba en un nivel donde les faltaban tres años para terminar la media, entonces queríamos apresurar eso y finalmente no conseguimos y nos dijeron busquen en un subvencionado ahí sí que con seguridad hay, no ustedes van a tener que pagar pero ahí hay cupo nosotros bueno hagamos el esfuerzo y la metimos ahí por un colegio por ahí tres cuartos en Santa Rosa Con...¿ qué es eso? se llama Santa María de los Ángeles.
I: Uno católico de ahí de Santa Rosa
Ay tan malo pero tan perverso, bueno ahí la recibieron y a las niñas dime las recibieron en una escuela muy... Ahí nos acogieron Apenas llegamos la directora ese viejecita Ay qué lindas las niñas que bueno aquí Tenemos muchos niños extranjeros pero no había ningún colombiano todos eran peruanos las niñas que bien ellas eran como reinas de la escuela.les fue muy bien a las dos chiquitas luego nos dimos cuenta que la escuela no era tan buena tampoco pero las niñas las pasaron muy bien ahí.
I: ¿mi amigo en el colegio en Colombia? ¿Qué experiencia tenían?
P: Bueno vivieron de todo pero cuando llegamos ya estaban en colegio público pero inicialmente ya se estudiaron en colegio personalizado. Qué es distinto al personalizado de acá el personalizado de acá el personalizado de allá son 6 o máximo 8 alumnos por curso.
I: Y eso son pagados
P: pagados pero la clase media los puede pagar, Bueno cuando la clase media haz un esfuercito nosotros éramos clase media y la podríamos pagar acá ni pensarlo. Y empezamos a buscar eso y que no y que no y cuando miramos los valores que nos dieron de los mejores colegios preguntábamos así con la gente nos decían el nido de águila.
(Se rían todos)
I: $2000 dólares.
P: los sagrados corazones no. Allá en Colombia hay colegios que...
I: no son tan caros
P: ellos estudiaron en colegios así pero allá Nosotros hicimos un periplo por Colombia por varias ciudades. Cuando nos cambiamos de ciudad donde tocará y el último era dónde estaban Ya ellos estaban en un colegio... Estuvieron hasta en un colegio agrícola porque en algún momento vivimos en el campo y ellas fueron a un colegio agrícola donde parte del uniforme era la botas estás de caucho. Porque tenían chanchos iban a... Puro campo con niños campesinos y todo. Ellos tenían un motor de adaptación y haciendo cuentas mis hijas: la mayor estudio en entre básica preescolar básica y media incluyendo Chile 10 colegios ya nueve de colegio.
I: ellas sí que saben cambiar.
P: y ella acá ella se adaptaron súper bien para ellas no fue problema que los salones fueron con hartos niños, Qué hubiera de todo no tuvieron problemas por eso.
I: ¿Y ustedes se movieron harto en Colombia entonces antes de venir por acá? ¿De dónde son originarios?
P: Nosotros somos de Cali, Yo soy de Cali y mi esposo es del centro del país del eje Cafetero. Pero anduvimos en varios lugares...

I: ¿y cuando vivían con su esposo en qué parte vivían?
P: cuando nos vinimos para acá vivíamos en el eje Cafetero.
I: Antes de venirse
P: Sí vivíamos en el eje Cafetero
I: ¿y esos movimientos También tenían que ver con los motivos que abandonaron el país?
P: Pues los primeros movimientos eran por trabajo pero los segundos movimientos y fueron por las situaciones de hecho yo tenía planes de irme a Estados Unidos y fui a Estados Unidos y estuve seis meses allá con la pretensión de organizar todo para qué él se fuera con las niñas. Pero él me dijo no todo está bien si todo está bien y cuando yo llegué de Estados Unidos no estaba tan bien. Ahí y ya sí qué... Yo llegué en octubre del 2006 y en febrero del 2007 fueron todas las situaciones fuertes y allí tuvimos que salir.
I: O sea se venía venir ¿cómo que aumentando la tensión?
P: sí
I: ¿Cómo fue que se produjo este conflicto?
P: porque Bueno mi marido trabajaba en una institución del estado y él ahí tenía digamos... Digamos que era como un PDI y de acá lo que pasa que el nivel de allá es muy elevado porque como los capacita la DEA e... Mucho organismo internacional los capacita para que hagan ese trabajo y él tenía un trabajo así como de mucha responsabilidad y donde se estaba en muchas cosas. Pero implicaba que nunca estuviera en la casa, siempre que estaba en la casa se iba por 2 meses un mes completo que no sabías nada nada de él. No había WhatsApp no había nada de eso Entonces será mensaje.
I: eran como misiones que no podían contar mucho tampoco
P: yo no sabía muchas cosas, entonces él te iba cuando los niños empezaron a crecer ya nos empezó a preocupar... O sea cuando él estaba y nos íbamos de paseo la pasamos súper bien. Pero se iba a ir 2 meses y los riesgos y todo ir siempre quiso estudiar y nunca pudo estudiar. Como yo ya había terminado mi carrera y todo y allá el sistema provisional si te permitía en esa época ahorrar te iba quedando para cuando terminas una vinculación laboral tienes dinero. Sí que yo juntamos mis Platas juntamos un subsidio que le daban a él y ahí montamos un negocio. Yo le dije ya montamos el negocio ya retírate. Y ellos tienen como una pensión de Gracia que después que tienen 15 años de servicio se pueden retirar con media pensión. Que no es malo y él se retiró porque dijo me pongo estudiar nos ponemos a trabajar en el negocio propio y...
I: ¿y a él no le gustaba el trabajo?
P: ya no le gustaba. Porque empezó a chocar con muchas cosas que iban contra principios y fuera de eso eran esfuerzos grandes de trabajo que finalmente como hay tanta corrupción. Yo lo supe después que por ejemplo: hacían todo una investigación para capturar al capo más capo de tal cosa y ya estaba listo el operativo el Capturan a ese tipo y va a implicar condecoraciones.
I: está bien dónde estaba
P: y faltando 10 minutos alguien decía no se hace y no se hace e... Y así como lo más fuerte Lo más fuerte que vivió que luego yo lo supe fue que... Que ahí fue cuando él quisiera retirar ahí vio un grupo de diputados secuestrados por la guerrilla y sabían dónde estaban los iban a rescatar sin una sola bala estaba todo listo. Una semana antes no se comunicaban con nadie porque eso ya estaba listo y faltando una hora que partiera todo el operativo llegó la orden creo que presidencial y no se hace el operativo y no los rescataron y los mataron. O sea el tiempo yo ya supe que ya lo habían matado yo estaba acá ya vivía acá cuando se supo que lo habían

matado. Entonces esas cosas a él le dieron muy duro y él empezó a pedir... Porque allá no se puede decir me voy y ya hay que esperar que lo autoricen. Se demoraron un año en autorizarlo.
I: Porque si tú te retiras no tienes autorización es como que estás fugitivo
P: Claro porque cómo tienen tanta información y Bueno le insistieron y me insistieron y le insistieron y finalmente ya no dieron más y de ahí le aceptaron la renuncia y finalmente ya no dieron más y ya y le aceptaron la renuncia. El renuncia y quedó como una persona civil de los grupos donde él ha estado trabajando se supo quién era él. O sea Él trabajaba con gente... Cuando él se infiltraba con gente que lo llego a conocer y una persona que concretamente que trabajó con él ella fue capturada era una mujer. Y ella confesó cómo se llamaba él en la vida real así como un caso de películas. El detective fulano de tal que trabajaba conmigo se llama tantos. Que eso no lo dijo amistosamente sino que ella fue torturada todo.
I: ¿ella era una compañera de trabajo?
P: No ella pertenece a un grupo y era la que te entregaba información porque ella quería salirse del grupo. Quería que el grupo se desarticula que por un tema personal porque la pareja ya le había quitado su hijo. Te lo han mandado para Rusia y ella quería rescatar a su hijo y la única forma de rescatar su hijo era que no tuviera papá. Porque le quitó la custodia de mi hijo Todo porque el tipo era un dirigente guerrillero Pero tenía muchísimo poder en diferentes esferas. Se le quitó el hijo y se lo mandó a Rusia y ella para que les articularán el grupo Ella hizo todo Eso de entregar información de todo eso y finalmente murió porque a ella la mataron y antes de matarla ella confesó. Entonces empezaron la cabeza del valía, este que nos traicionó que nosotros creíamos que era de los nuestros y no era de los nuestros hay que matarlo hay que secuestrarlo y bueno hacerle de todo. Y ahí inician a buscarlo pero inicialmente no querían matarlo era solamente llevárselo porque para ellos es una cosa de honor.
I: Cómo lograr capturar a quién los traicionó
P: sí y no matarlo de una sino que llevárselo a los Superiores para qué lo enjuician y bueno eso tiene una cantidad de cosas.
I: entonces todo eso dentro de la guerrilla
P: SI eso era dentro de la guerrilla, entonces empiezan a decir mira el que encuentra a Nassim le vamos a pagar súper bien. Como las autoridades haya son tan corrupta llegaron hasta donde la institución que Él trabajaba Cómo decirle a los PDI malos: miren están buscando a un fulano qué trabajo aquí averíguame donde vive dónde está tatata... Y los mismos de la institución empezaron a buscarlo también.
I: Claro porque en el fondo para todos es un enemigo
P: así que empezaron a... Entregaron información Pero afortunadamente él tenía amigos de verdad en esa institución Qué dijeron: mira acá llegó una información, te están buscando ellos eran los que le decían ándate tal lado porque allá llegaron. Y por eso él me decía ya no hagamos esto vámonos para tal lado y yo no sabía.
I: porque en ese intertanto tú no sabías esto.
P: Yo no sabía Entonces hasta que al fin ya un buen día él ya estaba estudiando en estudiaba derecho y un día e... Lo llamó a alguien así de mucha confianza de él y le dijo: Mira ándate hoy mismo Saca tu gente de tu casa porque este fin de semana sí o sí te llegan a la casa, y era como un día viernes Así que él me llamó y me dijo no nos vamos ya alista las cosas de la niñas las mochila y nos vemos en Bogotá. En otra ciudad nosotros vivíamos no sé tal vez en Talca Temuco

algo así capital para Santiago y ahí nos juntamos y ahí nos metimos a un hotel. Y esta noche la casa la balearon toda.
I: la casa donde ustedes vivían
P: claro eso no era para matarnos pero era para sembrar terror para aterrorizarnos y que él se enterara de pronto no sé. El caso es que se pasó y gracias a Dios nos salvamos de esa.
I: y ahí es cuando ustedes decidieron abandonar el país
P: ya sí ya cuando balearon nosotros ya estábamos lejos ya estábamos en otra ciudad. Y estuvimos como 12 días mientras que... Juntábamos dineros para salir del país porque cómo más.
I: no podían seguir dando vueltas por toda Colombia
P: claro todo eso fue como de película hay que escribir el libro, muy fuerte.
I: en el proceso de solicitud de asilo tuvieron que contar esta historia ¿cómo fue?
P: Ayyy (suspira) tremendo eso era lo más terrible porque la re victimización de eso es tremendo porque... La primera ocasión como que uno todavía estaba como el medio del susto Y todavía estábamos completando la historia porque había parte de la historia que yo no sabía tampoco... Y ahí te contó y no fue como duro. Pero cuando ya y como para uno no O sea como habíamos salido vamos a esperar que esto se calmen y ya volvemos. De hecho yo no me despedí de nadie solo mi mamá le dije qué...
I: ya las niñas se les dijo...
P: que íbamos de paseo, entonces como que uno todavía no asimilaba eso. Pero ya cuando en extranjería nos hicieron como unas tres entrevistas y siempre había que repetir lo mismo. Entonces en esas ocasiones que fue como a los 4 meses, a los 6 meses, ahí sí que era duro. Y nunca ¡nunca! Hubo un psicólogo ahí nunca las preguntas serán así como crudas. Como cuente y con detalle y en qué mes, como que le interesaba mucho arma la historia para presentarle a mí imagino, que ellos le presentaban en esta época creo que era como un comité con él ACNUR que se juntaba una vez al año no sé algo así. Entonces pero no había ningún tipo de... Era así como sin anestesia.
I: ¿cómo que buscaban que los datos coincidieran?
P: Sí yo creo que sí yo creo que para ellos era importante: Cómo que calzar a las fechas, Cómo que siga a ver si la persona es refugiado no. Y en algún momento esas pregunta si todo a mí me puso muy mal y la muchacha me fue a buscar un vaso de agua... Y estaban las carpetas así puesta entonces estaban así y yo alcanzó a ver había varios documentos eran consultas extranjería hacía al ejército de Colombia, a la policía de Colombia todo eso.
I: Como qué extranjería Estaba tratando de verificar...
P: verifican que es efectivamente porque efectivamente como mí marido trabajo con la policía y todo eso Entonces de seguro contando toda esa parte ellos la constataron entonces como que constataban todo eso. Así que este tema delante resguardar la confidencialidad no están así... No es tan así Y eso es lo tremendo porque como no saben vienen funcionarios de la policía a capacitarse acá de hecho de acá mandaron a los de la Araucanía para que aprendieran de las técnicas que allá tienen que esas técnicas las han aprendido allá Estados Unidos. Estados Unidos hace trabajo de que casi psicológico fuerte porque prácticamente en mi marido él tuvo un daño si fuerte fuerte fuerte muy fuerte.
I: es que como muy...
P: no llegó Aparentemente no tenía ningún...

I: daño

P: O sea cualquiera lo ve y no dice nada pero si le pasó la cuenta Luego si le pasó la cuenta la vida que le tocó vivir acá. Porque de vivir una vida llena de Adrenalina entonces esa cuestión y acá una vida relajada, pero sin esperanza de proveer económicamente porque allá la situación económica de nosotros era muy buena, porque él ganaba muy bien podía tener muchas cosas.

I: Claro porque estaba en un puesto de poder de alguna manera.

P: y cuando emprendimos nosotros montamos nosotros también estábamos bien. Y luego acá ya no había todo eso. Eso sobre todo a él le afectó mucho.

I: ¿a él eso daño?

P: sí.

I: ¿y en que lograron trabajar aquí? ¿Cómo empezaron?

P: no tremendo e... Nosotros teníamos allá una parcela y cuando nos veníamos teníamos un último... Nosotros creamos pollos así un galpón con 10000 pollos y eran 10000.

I: Una Avícola.

P: y una cosa grande y cuando nos venimos estaba la última camada para sacar y teníamos allá uno cultiva café y esto tiene unas cosechas entonces en marzo sale una y la otra sale en noviembre ya. Entonces vendimos la cosecha anticipa y con esto mi marido se compró un carro para trabajarlo, el dueño del apartamento que nos arrendo nos dijo mire Aunque usted puede trabajar sin problema es nene completo un auto y lo ponen de Radio Taxi pero licitado esos de placa anaranjada. Entonces con un esfuerzo de macho compramos un auto de esos y mi marido empezó a trabajar cómo del Uber Pero dentro de una empresa.

I: si el radio taxi.

P: era una cosa divertida porque él no conocía

I: en claro Por qué hay que conocer un poco la ciudad

P: en las páginas amarillas de este salían los mapas, no había Google Maps todavía

I: no todavía no.

P: Entonces yo me senté y cogí en la parte de abajo en el edificio habían un poco de estos directorios botados. Y yo cogí una cantidad y me los llevé en cómo no tenía nada que hacer arranqué las hojas y las pegue y arme un mapa gigante.

I: un mapa gigante

P: un mapa gigante de Santiago. Entonces mi marido me decía voy para toro mazote con 5 de abril. Ay toro mazote con 5 de abril ¿Qué comuna es? Qué es estación central listo chum. Mire tiene que tomar Grecia ahí está se convierte en mata ahí llegue a tal lado de ahí de la vuelta y no sé qué... No sé si va para arriba o para abajo y no sé qué yo era su....

I: usted era su...

P: y así él aprendió y nosotros conocemos Santiago

I: si la conocen muy bien.

P: si la conocemos muy bien porque eso era una cosa fuerte. Él empezó a trabajar en eso y bueno de ahí se conseguía no es muy bueno pero algo hacíamos. Y yo empecé a buscar trabajo ninguna parte conseguía porque con ese papelito, e... Como a los 6 meses me llegó mi hermana me hizo el favor de sacarme los documentos, que era así lo que tenía que presentar para el reconocimiento de título.

I: ¿usted qué estudio?

P: Yo estudié administración de empresa pero a nivel profesional porque acá hay técnicos que estudian 2 años Yo estudié 5 años y medio, y fuera de eso hice una especialización en finanzas y otras en recursos humanos hice un diplomado en certificaciones de ISO 9000.
I: O sea estaba súper formada.
P: Parecía un salchichón bien adobado, pero aquí nada de eso, cuando el digo administradora de empresas; así Mire te Hijo $ 200 mil pesos hijo 18 era el salario básico o me ofreció a menos del básico y todo eso porque yo no tenía ningún certificado.
I: Usted no tenía el título.
P: cuando me llegaron los documentos que los hice y en esta época el reconocimiento fue súper rápido; yo pasé los documentos y a la semana me estaban llamando me hicieron como una entrevista y entregué todo Y a la ya la otra semana fueran en dos semanas en total me entregaron el documento de reconocimiento de título, me dijeron ya usted puede ejercer su profesión. Pero lo que más me dijeron Pues que nadie me iba a recibir, o sea empecé a postularía postularía postularía postular. Entonces claro como el nivel de gerente claro cuando quieren contratar a persona sin que la persona les diga que es amiga del gerente del banco estado, ¿qué con qué banco tiene relaciones? Bancos de aquí pues no ninguno ya y mi currículum era todo de Colombia.
I: puras cosas desconocidas
P: para mí todo era desconocido que tener que hablar con un gerente de un banco sacan ni idea, nada de la legislación tributaria de aquí muy poco o sea muchas cosas que no, qué podría pero no o sea los gerentes para eso tienen asistentes, pero acá me pedían que yo ya dominar a todo esto y ya no los dominaba. Entonces empezó de pa bajo pa bajo. Finalmente me tocó irme a una consultora a coordinar una área y que tenían un proyecto con Lipigas no sé qué cosa y empecé a trabajar en una constructora que me dejaron trabajar pero a un nivel de asistente pero de asistente en nivel 1(se ríe), así como un poquito más del mínimo y eso nomás, y seguir postulando muchas cosas y luego tuve una muy buena oportunidad que fui con la Universidad Católica que tienen una fundación y ahí pasé todas las pruebas, todos todos todos, llegué ya la entrevista final y todo. Ya en la entrevista final cuando llegó la pregunta del millón: ya está todo perfecto si hay 2 candidatos está usted y otro candidato y tata ta... ¿Cuál es el número de su RUT? y yo no tenía RUT (se ríe). Porque resulta que nosotros acá sacamos un RUT tributario de 48 millones no sé cuánto para poder comprar el auto con ese funcionamos para todo. Y me dice: ¿es estés su RUT? ¿Y su carnet dónde está? No tengo carnet. No tiene carnet es que si no tiene carnet no es que usted va a ser la coordinadora administrativa de la fundación no me acuerdo cómo se llama pero función allá en la Alameda. Y usted va a ser la responsable de la cuenta corriente Cómo va a firmar un cheque lamentablemente no. Me mandaron una carta muy bonita y todo pero no, no pasó. Así que me tocó resignarme que afortunadamente tuve un proceso interesante que ya venía de Colombia trabajando en eso de crecimiento personal y me hizo aterrizar mea la realidad sin que fuera pues traumático. Porque conozco gente que ha entrado en depresión por no poder ejercer, no poder esto, no poder estar en las posiciones de estatus. Hay gente que le afecta mucho a mí es hoy en día me resbala pero en su momento fue por el tema económico me preocupaba mucho, más que la posición de lo que me generaba. ¿Oye pero tú no eras la gerente de no sé qué? ¿Y ahora en qué trabajas? Qué asistente del asistente del asistente del asistente. Eso no pero lo de la plata sí qué lata nunca ha llegado al ganarle acá lo que ni la cuarta parte de lo que en proporción yo ganaba en Colombia. Entonces

bueno me tocó me resigné Trabajé ahí y luego de ahí se terminaron los contratos porque era una empresa chica y se terminaron los contratos que tenían. Así que ahí me quedé sin trabajo un tiempo nunca hubo nunca hubo eso de una bolsa de empleos decente digo yo de la vicaría. Me llamaron una 5 6 veces pero es que claro teníamos situaciones económicas fuertes pero por temas de plata me ofrecían unas pestes. Fui a una vez una entrevista así muy entusiasmada porque no: es que vaya la entrevista ya usted la van a contratar. Era para un casino del aeropuerto para lavar los platos. Por Dios ¿cómo es posible que un ser humano recoja el currículum de una persona profesional y todo y la ofrezca? Sabiendo que hay mucha gente que estaba necesitando Y por supuesto que no iba a aceptar de una no los llamaban y me llamaban a mí. (Suspira) yo la verdad Yo le agradezco mucho pero la verdad no o sea, No porque sé a lavar los platos. Si mi por lavar esos platos me van a pagar $600000 siquiera yo voy y los lavó pero no. Prefiero ir a hacerme aseo de cuenta mía que yo manejé mi negocio yo digo voy a hacer aseo aquí aquí aquí, pero irme a patronal por el salario mínimo lavando platos no. Entonces yo digo eso sí que le hizo daño a mucha gente porque era como.... Y eso siempre le ha faltado a los programas porque es el Buscar abrirle puertas laborales No mirando las como un migrante que se vino a lo que toque hacer. Porque distinto escoger yo voy a hacer lo que toca hacer e... Y las circunstancias de uno Son distinta y el que está dispuesto a hacer lo que le toque hacer eso es otra cosa. Pero se supone que la protección debería también proteger eso no. Y hay gente que de la organización yo llegué a conocer gente y qué con profesión y todo le tocaba muy fuerte y refugiados que terminaron muy mal. Hay un caso de un peruano Raúl Paiva ¿ustedes lo conocieron?
I: No.
P: Hoy en día está interno en una clínica como de asistencia de asistencia mental algo así.
I-.Psiquiátrico.
P: Le dio ya demencia senil, comenzó a usar energía y parece que hace mucho tiempo que hace varias huelgas de hambre El día va como 30 años aquí o más. Él es un exiliado peruano del régimen de Fujimori de ese tema y él es estadista es un profesor universitario en su época cuando estaba lúcido no. Pero resulta que se comenzó a enfermar mucho y enfermó y se enfermó y la universidad en un momento en que comenzó a trabajar y no le dieron más trabajo. Y ya cuando se quedó sin trabajo él no sabía hacer nada más O mar ¿qué hace un profesor? o sea, cómo puede uno decirse que un profesor anda a trabajar a una construcción, como lo mandas a lo que es un joven de una empresa, un mar que es el chófer de una empresa ¿Cómo? si un profesor no sabe hacer otra cosa que enseñar entonces el joven cayó en una depresión de larga duración y se puso mal mal, y hoy en día esta ... A veces hay hacer eso en el planeta por qué cuando tienen que hacer las cosas así porque su salud se averió terrible por causa de que ... . Cuántas vacantes en las universidades ¿Cuántas? llenadas por gente pitutiada. Porque mis hijas estudian y me cuentan sus experiencias y yo digo ¿cómo se hizo ese tipo profesor? ¿O está señora cómo se hizo profesora? Porque mis hijas estudian y me cuentan sus experiencias y yo digo ¿cómo se hizo ese tipo profesor? ¿O está señora cómo se hizo profesora? ¿Cómo son profesores? la hija que estudia la administración pública de la verdad Digo deja mucho que desear la carrera de la administración pública de la USACH. Terrible De hecho mi hija está evaluando pasarse al vespertino.
I: A mira.

P: Qué Mira en lo que va corrido antes del paro había tenido a la semana en promedio ella tiene tres clases en promedio en una semana de 4 Ramos que tiene matriculados y él lo que ha ocurrido después del paro ha tenido tres clases, tres clases porque yo tengo los WhatsApp. Donde le llega hoy por motivos personales no puedo ir no sé qué más, la clase de a las 8 y le pone como yo tuve un asunto personal nos vemos a las 9:30. Entonces pues yo digo si está educación online mándale el material y qué estudien y que nos encontremos y revisemos o ponga les material. Entonces en ese sentido Terrible y que digo yo: a ese profesor con una persona decidida a entregar todo para ir para estar ahí y como no consigue un empleo y haciendo muchas gentes. Pero esto es porque no hay un programa que de verdad de acompañamiento al refugiado Aquí no hay, eso no hay. Hay voluntades personales de las personas que trabajan en las instituciones en el caso de FACIS, allá las asistentes que trabajan ponen todo su empeño. Pero claro no están amparadas por.
P: Pero ellas no tienen amparadas en un programa que de verdad tome en cuenta todo ella se Ejecutan lo que hay ¿Porque qué más pueden hacer? Y se mire hay recursos para tal cosa, ellas presentan sus propuestas a la medida de lo que hay porque no pueden.
I: ¿De qué manera los refugiados son tratados de la misma manera que los migrantes?
P: Como un migrante porque los recursos deberían ser en elevados. Porque hay otras cosas que tratar por ejemplo hoy en día: en la parte psicológica no es lo mismo que FACIS tenía un departamento de psicología con psicólogos de planta que se pongan la camiseta y digan ¿Por qué? Porque yo no creo que un psicólogo El adolescente de la edad de sí mismo, Con mucho respeto por ser capaz de comprender un cáncer que llega y le cuenta que viene traumado porque lo presenció otro cosas que ni son de la realidad ni de las noticias acá ni a las noticias .
I: ¿Ustedes llegaron en un contexto en que no se sabía mucho de...?
P: No hay nadie que haya dicho que haya llegado a otro nivel exiliado que haya empresarios y que vengan perseguido por guerrillas. Entonces, esta gente tuvo la protección del estado pero finalmente se llegó a las grandes prensas, porque ya tenía redes de contactos de nivel profesional, pero nosotros empezamos a llegar a la misma manera que las otras, nos conectamos con otras personas tema de que a uno lo persiguió la guerrilla, al otro lo persiguió a los paramilitares, al otro lo persiguió al estado, a otro la delincuencia. De hecho nosotros íbamos a la vicaría que fuimos como unas 4 veces, a nosotros nos citaron a las preguntas esas cosas y en algún momento nos citaron para evaluar el tema de los niños para un programa que eran como talleres artísticos, y la única vez que nos fuimos a un asistente como el grupo familiar llegamos a las niñas estudiando cómo hasta las 3:30 y nos pusieron la hora a las 4 de la tarde. Los niños estudiaban en Argomedo con Larraín ahí por Santa Isabel y nosotros salimos y e... Salimos a coger micro y qué micro y cogimos un taxi el taxi nos dio como 50000 vueltas para llegar a la catedral, desde ahí hasta ahí en media hora llegamos a las 4:10 en taxi Y todo llegamos a las 4:10 y el asistente social llegamos y le decíamos: llegamos es que teníamos una hora a las 4:00. Ay es que no sé sí los quiere atender y pues miramos y no había nadie entonces puede ser que nos atienda, llegamos y le tocamos la puerta el hombre salió: Sí. Es que teníamos la hora a las 4 de la tarde pero no alcanzamos a llegar. Lo siento pidan hora otra hora. Nos tiró la puerta en la cara. Entonces ese nivel de... Es que nunca más yo dije, si a mí me vuelven a llamar que el asistente social enrolle la cita y no, no nunca más.
I: ¿Este era un asistente social del FASIC?

P: No de la vicaría en esa época era la vicaría y nosotros como no tenemos o mar, nunca hemos estado conscientes de la necesidad del programa nunca nada de eso, ya por acá por los 2 años algo si ya. No nos gusta juntarnos con colombianos porque nos daba miedo.
I: Eso le iba a preguntar ¿cuál es la relación que se produce con la comunidad colombiana es la condición de refugio?
P: En esa época no nos juntábamos. Por allá por el 2008 a finales del 2008 más o menos con los talleres las niñas, las niñas empezaron a ir a esos talleres los días sábados.
I: Los talleres artísticos.
P: Entonces ahí vamos varias mamás, y usted de dónde es no sé qué y no sé qué más y ahí conocía al papá de unas niñas iban también ahí él había sido profesor universitario en no sé qué, él dijo Yo tengo unos amigos con los que me estado conversando y hemos estado pensando en hacer una organización y no sé qué les invito a mi casa. De hecho cuando la primera vez que me junté con ellos mi marido me dijo que se va juntar usted con gente ¿cómo se le ocurre? Yo le dije no pues es que yo voy a ir eso es aquí a una casa. Sí pero no sabe quién y no sé qué y no sé qué más, a lo que no me dejó ir sola así que él se fue ahí con las niñas ya los conoció vio que era una familia, Eran que tenía una situación nunca contamos los casos de cómo eran después nos dimos cuenta de cómo era todo inicialmente no.
I: ¿Usted es inicialmente no contaba mucho?
P: No contaba que hacía ni nada estoy ahí nos empezamos a hacer conocernos con otros amigos con otras familias porque en torno a los niños y las niñas estuvieron en un grupo de música que hasta que cuando inauguraron el museo de la memoria ellas tocaron en el museo de la memoria en la inauguración fueron el grupo artístico invitado.
I: Tuvieron un concierto grande
P: 2 canciones no sé qué 4 canciones.
I: ¿Que era como un grupo de música de niños migrantes una cosa así no?
P: Sí y ahí nos conocimos con algunas personas que todavía somos amigos la mayoría algunos ya no están ya en Santiago no volvimos a saber.
I: ¿Pero qué edad tienen los demás migrantes colombianos de los refugiados? ¿Existen poco de conflicto?
P: La sociedad en general cree que los refugiados hijo subsidiados por el estado. Ay pero también ustedes refugiada tan buena porque usted no le falta nada, (se ríes) porque cree que él refugiado es acompañado de asistencia económica también hoy en día aparecen solicitantes al Refugio con esa idea. Yo generalmente cuando me los encuentro o que me llaman lo primero que les digo si es que usted tiene una verdadera situación de riesgo para su integridad, utilice la figura del refugio pero si no no porque esto no conlleva ningún tipo de asistencia económica en Chile y de todos modos en ninguna parte del mundo, océano porque no tiene que ver con eso. Ahora lo ideal es que si las persona tienen la situación de y el país tiene condiciones hay un programa que acompaña eso. Pero no que la gente pide Un refugio para que le den plata. La gente dice no es que a mí me habían dicho que le daban $500000 peso mensuales.
I: ¿a dónde y a quién?
P: bueno un grupo de gente que sí los reasentados de Ecuador y de Costa Rica tuvieron asistencia económica como que ellos tuvieron medianamente cómodos, les daba cierta comodidad para pagar un arriendo así como para un año les dieron a ello. Pero ellos fueron traídos engañados porque ellos venían a vivir a... Les llevaron un video que yo tengo muchos

amigos de ellos, les llevaron un vídeo de las calles de Santiago que eran de Plaza Italia para allá: los edificios de cristales, el intercontinental, el Hyatt todo eso así, para allá se veía la Cordillera linda. Para ya iban a vivir ellos.

P: Estas son las calles de Santiago, los colegios les mostraron así los colegios... Estos son los colegios de Santiago... Los colegios dónde van a estudiar sus hijos y ellos contentos muchos ya tenían su vida nada ya Y se vinieron. Y cuando llegaron acá; los llevaron a vivir a la feta... Esa calle que se llama la Feta hasta Brasil esta Camín Brasil no sé por ahí por esos lados. Por ahí había una casa grande y ahí llegaron a vivir varias familias.

I: ¿Que eran como Los cites del centro?

P: Era como un cite y como que organizaron familias ahí, y luego cada uno fue buscando su sus cosas porque vivir ahí era como complicado. Pero creo que en esas casas vivían así como unas 6 familias algo así pero era un cite grande ya había varios departamentos ahí y eso fueron los departamentos lujosos que...Así que ellos vinieron engañados y ellos tuvieron asistencia un año pero nosotros no, Y los palestinos los sirios que han venido con programas de Naciones Unidas ya como con recursos listos para eso qué es distinto. Entonces también muchas veces dice la gente no es que no pasaron en televisión, la presidenta no nos fue a recibir al aeropuerto. Sí pero a ellos. Los que llegan allá por frontera que están en Tacna intentando entrar, los que entran por los pasos inhabilitados eso viene con una mano delante y otra atrás y nadie les da... Es más no les reciben la solicitud.

I: Eso le iba a preguntar ahora cómo está la situación de Refugio ahora ¿cómo ha ido cambiando porque usted con su historia conoció mucho no?

P: La ley fue una ayuda ya porque hubo por lo menos un reglamento que yo digo los hicieron con los pies porque tú sí mira la ley y miras el reglamento sola misma cosa. Se supone que el reglamento le debe haber ingresado a algo pero dice por ejemplo los refugiados deberán tener un trato más favorable, lo dice La Ley lo dice en el reglamento. Pero qué quiere decir más favorable. Dice el trato más favorable y en ningún caso inferior al que se le da a un migrante en igual situación. Entonces según eso el estado ha violado la ley porque los refugiados sirios, los refugiados palestinos están en las mismas condiciones que yo. Si al refugiado sirio al refugiado palestino la han puesto un departamento, le han puesto subsidio a todos les deberían dar porque ahí hay un tratamiento diferente para... ¿De dónde viene la plata? Eso no importa pero la ley dice qué tiene que ser en igualdad de condiciones para todos los refugiados, Cómo que no va a distinguir entre un refugiado y otro. Pero si ha distinguido entonces... Como la ley. Por la ley había cambio de papelito ya por un carnet o sea cuando te reciben la solicitud te estampan en el pasaporte la solicitud de refugio y te manda para registro civil y tienes un carnet de un mes. Y con ese carnet es una visa temporaria es un carnet provisorio Por 8 meses pero que te permite buscar trabajo, entonces la gente ya tiene trabajo. Ahora el reglamento tampoco dice Cuál es el plazo máximo en el que el estado tendrá que resolver la solicitud del solicitante; Nos dices si me 6 meses, no dice el estado estudiará la situación en menos de un año y máxima en un año tendrá una respuesta positiva o negativa pero en la tendrá, no diste nada de eso así que hay gente que creo que lleva 4 años. Creo que el maestro tú conoces al maestro lleva 10 años esperando que le digan si le van a dar la condición de refugiado o no. 10 años entonces ahí se renueva muchas veces el carnet

P: Le renuevan le renuevan pero esa renovada de renovado no es una solución. Porque mira hoy en día por ejemplo en el caso mío, cuando me dieron mi primer carnet igual no me recibían

pero ciertos cargos porque la empresa decía el cargo es de responsabilidad usted tiene un carnet de un año si usted después de este año es irse si o para otro país y yo ya invertí en usted en capacitación hay una cantidad de cosas que usted va a dejar tirada si es que se va ir. Entonces cuando tengo una residencia definitiva hablamos. No me daban un cargo importante bueno que estuviera al nivel de mis conocimientos, de mi experiencia porque no tenía una definitiva y le pasa a mucha gente, que no le dan un buen empleo por eso. El que te va arrendar si no tiene a definitiva yo no le arriendo, también porque en cualquier momento se va.
I: ¿Las cuentas en los bancos también?
P: Se puede abrir una cuenta vista Pero no se puede abrir una cuenta de ahorro ni una cuenta corriente ni nada, Yo no tengo cuenta corriente en mi casa nadie tiene cuenta corriente porque a pesar que tengan definitiva en méritos para eso. Pero en su momento cuando trabajaba así yo trabajara tuviera un sueldo medianamente bien no me habría una cuenta corriente. O sea no es sencillo entonces la gente se está dos tres cuatro años en eso y no le resuelven...Noo le resuelve y van y van y van y les ofrecen cada vez que van: bueno pero porque no se cambia de visa cámbiense de visa ya tuviera definitiva ¿y porque tiene que ser así?
I: O sea están de estimulando las solicitudes de asilo.
P: De hecho ahora que hubo el tema de La regulación migratoria ahí hubo una situación Bien bien y yo sé que el número si en este momentico se compara el número de solicitantes que hay yo creo que debe haber bajado cantidades. Porque muchos de los que eran solicitantes los motivaron extranjería y a través de ciertos personajes por ahí en redes sociales y todo a que se inscribieran en el proceso de regularización y que dejarán de lado la solicitud de refugio, que porque así le iba a salir más rápido. Ya a mí me llamó mucha gente: ¿es que mire que yo hago? es que ya se va a vencer el plazo y yo...U usted no es irregular. No es que me dijeron es que yo tengo que ir a inscribirme. Y utilizaron Lamentablemente utilizaron figuras... Tenemos un amigo Qué es refugiado y él perteneció a la organización inclusive quién participó en el comando de Piñera Cómo de esta cuestión, entonces y conoce mucha gente en las imágenes de donde estaban repartiendo los turnos allá en el primer día en el estadio...
I: En el estadio Víctor Jara.
P: Él estaba allá con su chaleco; el gobierno de Chile no sé qué. Claro que sí don Antonio se estalla pues hay que ir.
I: Utilizaron los liderazgos...
P: Y el de pronto estaba por intereses personales talla pero no porque fuera de la organización y mucha gente se inscribió mucha mucha mucha. Así que esa gente ya no es solicitante.
I: ¿Y Cuál es la diferencia Cuáles son los efectos de haber transformado las solicitudes de asilo en migraciones laborales?
P: Claro ellos dejan no sé, es que entiendo con la regularización necesariamente no les dan un permiso de trabajo, algunos la están dando temporarias pero si tenía un contrato el que tenía contrato bien pero el que no tiene contrato le van a pedir un contrato y se va a quedar con temporaria con temporaria contemporáneo por mucho tiempo. Porque si no cumple los méritos: no cumplen los tiempos de liquidaciones de AFP no va a tener nunca. Y el asunto es que si para el estado se merman la solicitudes, o sea ya no va a tener que resolver todas esas solicitudes y no va a tener que atender a los refugiados... Que el número de refugiados que podría ser si esas solicitudes se cursarán y ahora cuando venga la firma del pacto global que eso va a ser como a fin de año tal vez ahí chile está adhiriendo, y los estados que adhieren van a

cumplir a los compromisos a que se... Compromisos que acordaron allá. No sé qué compromisos serán porque de lo que sí hay es participación Sí o sí hay, por ejemplo es mejor que no haya tanto refugiado aunque los que haya sido que ya tengan resuelta su situación. Por ejemplo como para nosotros que yo hablo con gente de nuestra época y dicen ah no y para reuniones de la organización Es complicado convocarlos porque la gente dice no. Nosotros a horas nos reunimos los colombianos en torno al tema de la reparación por víctimas del conflicto armado, solamente por eso pero por el tema de refugio es difícil convocar. Porque la gente dice: a yo no voy a lidiar más con eso, nunca nos dieron nada, nunca nos apoyaron en nada Menos ahora. Pero de lo de que nosotros vimos en la Cumbre del refugiado Es que la idea es cómo que haya un estándar de atención al refugiado en todos los países donde hay el estatus de Refugio como medida de atención inmigrante. Entonces ahí por ejemplo no creo que vayan a medir por lo más bajo si no por una cosa media y hay muchos países donde el refugiado tiene por lo menos un cupo en la educación superior. Porque haya lo que vimos Pues que la cantidad de refugiados que hay de organizaciones casi todos son jóvenes y todos están en universidades pero súper bien: en Europa, Estados Unidos en todas partes y su universidad está completamente cubierta todo no tienen que pensar en nada. Y aquí ni siquiera un cupo ni siquiera un puntaje, ni siquiera en el registro único de hogares te cuenta que seas... De hecho a mí me merma por ser profesional, me merma yo no puedo acceder a nada.
I: ¿El hecho que se ha refugiado aparece de alguna manera?
P: Nosotros los planteamos hace tiempo como organización que fuera considerado dentro del registro de esa época, que eran la ficha de protección social. Como con un Plus independiente del nivel académico del refugiado. Porque no es lo mismo el refugiado profesional que el profesional migrante. O sea él Pudo armar todo un paquete nosotros no, pudo inclusive escoger donde ir a ejercer su profesión nosotros no.
I: Pueden venir con los títulos conocidos, pueden haber escogido el país donde tienen los contactos como decías tú.
P: E inclusive nos resta por qué claro como en el caso mío mi carnet dice profesión y yo no sé a qué hora me pusieron eso. Porque la emoción cuando me dieron la definitiva dentro del paquete de cosas iba para el registro civil y lavaste todas las cosas a la señorita, mire Yo vengo por mí cédula no sé qué y yo emocionada y ella me dio no miré tatatatata...y ahí va una copia del reconocimiento de título y ella me puso en el carnet y de una vez me clavó mi profesión. De nada me sirve, o sea, me perjudica porque por ejemplo por eso para cosas sociales no O sea yo tengo cero accesos. Porqué se supone que él como que el cartón viene acompañado de una cuenta en el banco.
I: Pareciera que sí que fuera inmediatamente requisito. (Se ríen).
P: Vaya Mira es todos los Uber todos llenos de profesionales.
I: ¿Y en términos específicos derechos en salud de necesidades en salud?
P: Mire ahí yo en lo personal no he necesitado e... Como un tipo de atención especial de salud.
I: ¿Pero por ejemplo cuando llegaron ustedes inscribieron a las niñas en los consultorios?
P: En esta época no nos dieron nada de información, allá y nada nada de información de hecho: nosotros incurrimos en un gasto pero grandísimo porque nos preocupamos mucho la salud Yo decía: ¿las niñas se me llegan a enfermar yo qué hago? Pero ellas nunca fueron enfermizas pero veníamos con tratamientos de ortodoncia la niña mayor y yo. ¿y cómo es una ortodoncia Cómo hacemos? averiguamos por allá una señora nos ofreció un plan de atención a todos con todos

los ortodoncia y nosotros felices porque ya no nos pidió nada. Pero claro que nos iba a pedir si no sacaron Hasta los ojos, la clínica Cumbre $25000 mensuales en esta época para que nos revisaran.
I: ¿Solo para que le controlaran...?
P: Eso era lo usáramos o no lo usáramos. Pero cada revisión eran 12500 pesos.
I: Carísimo.
P: Ay Dios nos metimos a eso y ella nos contactó un ejecutivo de banmédica. Que también nos entregó un plan de salud que pagamos como $150000, él no se explicó en esta época que las mujeres por el riesgo que cómo éramos cuatro mujeres.
I: Carísimo.
P: Y nosotros pagamos eso como un año. Cuando yo empecé a trabajar que ahí me enteré de Cómo era el sistema de salud dije: no ya chao. Y ahí había un personaje don Lorenzo Agar que fue director de Refugio de... La vicaría y él hicieron una reunión para explicar qué ya los refugiados teníamos derecho al nivel A, al nivel de indigentes y ahí los refugiados: Ay ¿Cómo que indigentes nosotros no somos indigentes? No es el título que le dan a la persona es igual que un chileno. Claro entonces ahí la conclusión es un refugiado el estado que simplemente le da toda la atención ero claro le da toda la atención al igual que a un chileno pobre. Pero cómo se hace que nosotros como refugiado le damos un tratamiento especial al estado sin ni a sus mismos connacionales te los da. O sea como vergonzoso decir nosotros no estamos vivienda, nosotros necesitamos educación.
I: ¿Usted conocía el sistema chileno?
P: No nada. No yo chile capital Santiago Argentina capital Buenos Aires, No ni idea de cómo era Chile ni idea ni idea ni idea... Y claro luego también la gente los que han llegado después mucha gente que ha escogido: no es que a mí me contaron que Chile era pero maravilloso, que aquí esto era llegar y recoger plata que esto era maravilloso. O sea nunca la gente se imagina: que existe La Pintana, qué existe La Pincoya, qué existe la Santa Julia, La Rosita renal la gente no sabe. Y si hay gente Quién fue ejemplo yo conozco colombianas que viven aquí en Ñuñoa ¿En Ñuñoa hay poblaciones hay Villas? Si La Freire, si La Exequiel váyanse a la Exequiel vayan a Rosita. Eso no es Ñuñoa. Cuando quiera lo llevó. Tengo son amigos que te cambiaron Se pasaron ellos vivían por Simón Bolívar y se cambiaron o niño en Excel Fernández con los espinos no sé por ahí. Hay un edificio y desde edificio por la noche se ve cómo se echan balas en la Rosita renal y ellos no sabían decían yo no me imaginé no esto es terrible no sé qué. Que como se vende qué Chile es la Suiza de Latinoamérica.
I: ¿En general qué imagen se lleva los colombianos de chile en cuanto a seguridad?
P: Pues claro hoy en día igual que al nivel que se vive en Colombia nada que ver con el tema de seguridad. Claro como uno tiene si la prevención como le dicen que todo es maravilloso Entonces no le da miedo por donde le da miedo a la gente. Yo en estos días por ejemplo fui a Colina y me cogió la tarde y me fui en bus porque yo iba sola, dije que me voy a hasta colina y me toca pagar peaje me voy en un bus y me devuelvo. Me cogió la tarde y me dieron las 10 de la noche y hasta las 10 de la noche sale el bus, que me parece terrible que hay gente que estudia y que trabajan allá. Entonces yo le dije a los amigos Dónde estaba chileno: le dije no me voy. ¿Cómo se va a ir? De alguna forma. No Cómo se le ocurre no sé qué. No yo salgo yo tomo algo de pasar un taxi. No es peligroso no sé qué me fueron acompañar como 10 personas que exageración. A mí no me daría miedo yo andaría como si nada. Finalmente me vine en un carro

Cómo pirata que me recoge que me trajo como Por 1500 al Mapocho idea y del Mapocho cogí un taxi y ya llegué al fin casa y ya. A mí no me da miedo porque no mido el riesgo Por qué no lo he vivido nunca y al principio nosotros éramos así cuando teníamos el auto, cuando compramos el auto nos fuimos a recorrer Santiago. Entonces empezamos a darle vuelta a la Vespucio y llegamos a la Florida en algún momento y estaba el taco para coger la autopista y había una callecita así despejada. La gente toda en ese taco nosotros atravesamos tranquilamente llegamos a gran avenida y todo. Al tiempo le Contamos a alguien que tan bueno que era esa calle.¿ ustedes pasaron por allá? Nosotros atravesamos la pura lengua sin saber que esa era la legua, las calles despejadas nadie no miro No solamente no miramos a nadie inclusive decíamos esto por acá tan entretenido la gente en las calles, todos los jóvenes compartiendo, hay como fogata Mira hacen como fogatas en la noche. Eso es lo bueno de no saber de hecho como nosotros vivíamos acá en Plaza Italia en carabineros de Chile en el piso 19. Los primeros días yo me asomaba por la ventana y se estaba empezando a cubrir de nieve Mira qué lindo. Por las tardes casi siempre los viernes se reunía la gente y echaban pólvora y había como bengala y esto iluminada bonito y bueno era como una fiesta. Nosotros miramos por la ventana ya están como en Fiesta ya vamos a escuchar ruido Como tambores todo chévere entretenido.

P: Y cuando un día vale Este me dice que tal ¿cómo les ha ido? Yo bien sí súper entretenido los viernes hacen una fiesta allá abajo (Jajaja se ríen). Muy buenas.
I: Eran barricadas
P: Esas son las protestas. Nunca vi el carro lanza agua nunca lo vi siempre veíamos la fiesta.
I: Ahí empezaban.
P: Claro eran los tambores ahí salía la marcha de ahí salía y luego el resto de cosas.
I: Terminaba después con detenidos.
P: Si al tiempo me enteré que por allá que daban la escoba por otro lado y claro Yo no veía noticias no me preocupaba de eso. Entonces la percepción de la gente de... Pero igual todavía aunque a mi marido le han robado aquí tres veces, una vez lo robaron... Le apuntaron con un arma y todo eso lo robaron, cuando trabajaba el carro lo intentaron robar también y otro día después de lo del carro él se consiguió una persona que le manejada al carro por el tema de la licencia porque no tenía licencia. Entonces ahí hubo que conseguirme una persona en usted y él consiguió un trabajo cómo Junior de una empresa en una motocicleta y ahí él era Junior pero no tenía nada que ver con platas. Pero le robaron la motocicleta en que la que andaba unos tipos le amenazaron él se estaba bajando de la moto y motocicleta y le dijeron pásame las llaves se llevaron la motocicleta. Y lo más divertido es que al tiempo esa motocicleta apareció el mismo labio en el centro, y me diste Esa es la motocicleta que a mí me robaron y fueron de los carabineros y dijo a mí como hace tres meses me robaron esa motocicleta que está estacionada halla es la patente tanto y el carabinero les fue con él y le dijo si quiere llamé y averigüe si está motocicleta es robada.
I: Está incautada por robo
P: Y sí ahí llegaron y la incautaron se la llevaron y no sé qué. Nunca nos llamaron nunca pasó nada pero recuperaron la motocicleta esa era de donde la empresa que Él trabajaba. Pero a mí a mí me han robado: me sacaron el celular, una vez me lo arrebataron de la mano. Tonteras así pero uno de todos modos la sensación de seguridad qué tiene vivir acá es diferente. En Colombia yo fui en diciembre después de 11 años fui…

I: ¿Eso le iba a preguntar si había vuelto?
P: Yo fui y sentía mucho temor.
I: ¿Por dónde estuvo?
P: Fui a Cali y la sensación... O sea nunca me dejaron salir sola, de mi casa siempre salí con mi hermana y conduje como unas 4 veces pero ahí mismo recordé como todo, o sea, yo cuando me vine para acá Escuchar una motocicleta al lado del auto para mí era terrible y ahora también cuando fui que salió en el carro de mi hermana y cuando en un semáforo de pronto sentí a una motocicleta al lado Ahí sentí terror Sentí mucho terror. Ya Bueno dije ya no ubícate no pasa nada pero cuesta. Acá no habían motocicletas cuando llegamos ahora es que hay motocicleta. Y con los colombianos que uno habla dice no si acá esto es súper relajado. Se dan más casos de los robos como con engaños como con cosas así que el nivel de violencia... Y bueno si uno anda como sitio como este como ya catalogados de peligroso como que uno no ve nada. Yo nunca he visto que asaltan como en Colombia veía: uno a veces iba en el carro y veía un asalto. Solamente una vez vi que un turista le robó por los lados del parque forestal su cámara, no sé cómo se la sacaron y el tipo corrió y corrió y corrió y se fue con la cámara. Pero cosas así como ya te sacan una pistola yo en Colombia vi eso muchas veces muchas veces me daba mucho terror y acá eso no se ve.
P: Y la gente de todos modos valora que hay oportunidades laborales sobre todo emigrante común y corriente, el migrante que me vino decidido a todo, porque tenía redes de contacto y todo conozco una gente que trabaja en unas posiciones muy buena ganan muy bien porque tuvieron la oportunidad de armar sus redes de contactos. Entonces es distinto porque se insertaron en otros medios, nosotros no la mayoría de refugiados de nuestra época no la mayoría estamos en situaciones todavía: Cómo con casa propia nadie, Cómo con acceso a cosa. No uno cómoda su estado su nivel de vida pero no alcanza a integrarse al 100 cómo lo hace un migrante Normal.
I: ¿La mayoría que vino de este grupo un poco de su generación tuvieron que ser como ustedes de arreglárselas de Cómo superar el miedo la depresión?
P: La mayoría por sus medios y las ayudas digamos en el tema de salud Mental que fue una cosa que cuando salió el proyecto de ley e….
I: ¿El 2010?
P: Antes del 2010 en las recomendaciones... En Las observaciones nosotros nos reunimos con varios refugiados y a través del padre Tupper hicimos llegar de Rodrigo Tupper, ya ahorita ya no es padre porque se casó el año pasado. Él se retiró del sacerdocio y el año pasado se casó y a través de él... Digamos como que no patrocino Porque él fue a presentar Las observaciones del lado de los refugiados. Nosotros ahí pedimos que los interrogatorios para los solicitantes que fueran acompañados para los menores y qué hubiera acompañamiento de un profesional si era un solicitante menor de edad que viniera solo. Y que ojalá que las personas tuvieran acompañamiento psicológico, por lo menos los de lo de los menores eso pasó eso quedó ahí con este resguardo. Pero los programas no implican… lo que les decía yo que no es lo mismo que sea un funcionario de planta que se especialice en esta área y todo esto, aunque sea al estudiante que está haciendo la pasantía que quiere acabar esas 400 horas para poderte titular, porque no hay nivel de compromiso no hay ningún compromiso. Yo aquí estudié una carrera técnica En vista que el tema Laboral era este yo dije me sale mejor ser un técnico, porque un técnico me prestan más y estudié prevención de riesgo y ahí yo hice práctica: y ahí me di cuenta

como son las prácticas. Yo tuve una práctica de lujo Yo trabajé en la mutual de seguridad A mí me mandaron a lo mejor de lo mejor. Pero veía los compañeros sacando fotocopia, escaneando documento, yendo a hacer el depósito al banco. Y también los que hacían yo lo que yo estaba haciendo hacían con los pies el trabajo porque había que acabar con estas horas rápido para poder titularse. Entonces no había ninguna expectativa de quedarse ahí en la empresa ni nada. Yo me quedé cuando termine la practica en la mutual trabaje la mutual otros seis meses con la mutual y gané bien todo. Pero lo que uno ve es eso que el estudiantes en práctica a veces no pones de todo de sí porque es una etapa

I: Un trámite

P: Lo mismo pasa con la asistencia legal. También si son estudiantes en práctica y cuando se van de vacaciones se van de vacaciones y la asistencia psicológica lo mismo. Entonces FACIS creo que tiene con... no sé me parece que con Alberto Hurtado qué tiene lo de psicología en la clínica psicológica y la vicaría tenía con... pero todo mandado de externos y esos eternos no...

I: No tenían elementos para.

P: entonces yo te digo un chico que estás tú Terminando su carrera, que no tiene herramientas suficientes para enfrentar , Esto está hasta traumante ellos así pollos terminando apenas la carrera, habiendo visto toda la teoría que se ve y le llega alguien y le diga mire yo estoy en esto porque yo vi como desmembraban a mi primo. ¿Cómo así? sí que lo que pasa qué le quitaron los brazos después de las piernas esto y lo otro le cortaron la cabeza. Cómo se enfrenta a eso él solo relató ya te trauma (se ríe) Así cómo va poderle darle una solución Es complicado.

I: Y la última pregunta que me surgía de esta historia ¿Sus hijas Cómo vivieron este proceso? ¿Ellos se sienten refugiada se sienten migrante se sienten colombianas?

P: Yo creo que ellos se sienten más migrantes. Porque como no conocieron la historia en ese momento Entonces ellas no estaban como Tenemos que irnos. No ellas creían que veníamos de paseo Aunque mi hija mayor... Yo a ella le dije porque de hecho yo pensaba que era así que cuando nos vinimos yo pensé que íbamos a estar un fin de semana por fuera yo no me di cuenta tampoco. Y entonces ella cogieron la mochila del colegio sacaron los cuadernos y echaron algo de ropa pero la niña mayor hecho fotografía Ella tenía una álbum con fotografía, ella echó las fotografías. No sé qué para cuando cumplió los 15 años yo llamando mi hermana Mira Búscame porque el papá le quería hacer porque él es fotógrafo entre otras cosas El fotógrafo y camarógrafo y todo. Y el estudio También acá el trabajo en chilevisión un tiempo. El hijo yo le quiero hacer un pequeño vídeo con todas las fotos que cuando ella estaba chica entonces dile a tu hermana que me mande las fotos así como una de cada año porque nosotros teníamos que los meses que no son años que no sé qué. Y mi hermana busca y busca y las foto y no entre lo que había juntado Ella dijo no se perdieron las fotos no hay nada, O sea, hay algunas pero no sé qué pasó con las fotos. Cuando ella dice es que yo las tengo ¿cómo? Y ella las tenía ella las había arrancado de los álbumes y había traído a un paquete así de fotos.

I: ¿No vivieron ningún momento de cómo oposición a todo esto qué estaba pasando En este período?

P: No ellas nunca, lo que pasa es que bueno lo otro Es que ellas empezaron a ir a natación. En Colombia yo siempre trabajé con deportes Entonces yo siempre las llevaba a cursos de natación ella sabía nada. Entonces cuando llegamos aquí que las ponemos a hacer en el rato libre la tarde sin hacer nada.

I: Sin familia sin amigos.

P: Sin amigos o sea los del colegio y nada más y llegan a la casa y nos veíamos la cara los 5 ni salíamos 5 y andábamos los cinco por todos lados. Así que empezamos a averiguar y la llevamos a la Chile y a halla empezaron ellos aprender estilos empezaron a aprender, la vida solos de la natación ahí son los amigos del alma de todo todavía se juntan para los cumpleaños. Y empezaron a nadar entraron en la rama competitiva de la Chile.
I: Yo también estuve en la rama, pero muchos años atrás.
P: ¿Conociste a Gabriel a Gabriel Torres?
I: Yo tenía a entrenador pelo largo Moreno No me acuerdo nombre Juan Carlos se llama pero yo terminé dejé de hacerlo a los 14 años.
P: Sí entonces ellas ahí se integraron sus amigos entonces ella salían a competir no sé qué, no sé qué más y se adaptaron también entonces ellas nunca sintieron... Y la natación para ellos ha sido de hecho ahora la Valentina estudio, la que ahorita estudió administración pública primero estudio enfermería. Ahí entró por cupo deportivo a la USACH.
I: Ah okay.
P: Y Natalia entró estudió en la mayor pero luego le entrenador de la USACH le dijo que se cambiaba de carrera que le conseguía cupo. Finalmente no utilizo el cupo pero está allá ellas son del equipo representativo de la USACH y aprendieron hacer juzgamiento en natación. Entonces ahora ellas Ya son jueces sudamericanas las 2 van a suprimir sudamericano en noviembre Argentina, pero ya son jueces nacional y Sudamérica de natación hacen juzgamiento. Entonces a veces van a competir y a veces van a... Cuando es por la universidad van a competir y cuándo es campeonato siempre las llaman para hacer jueces. La mayor no siguió ella no siguió porque ella Tuvo una bebé chilena y ella ya trabaja, está terminando su carrera, pero a ella ya no le queda tiempo de entrenar y no volvió a entrenar las otras dos todavía entrenan representan a universidad.
I: ¿y representan cómo?
P: Sí de hecho creo que la Natalia tiene los Récord en fondo de la USACH los tienen ella. La otra vez hasta el ACNUR le hizo un reportaje en la revista del ACNUR por el tema deportivo que tampoco hay ningún tipo de programa de apoyo. De hecho Mira la Valentina en algún momento fue y participó en un campeonato internacional, en una cosa que se llama la copa austral donde participan: chile, Argentina, Uruguay no sé y lo hacen en el sur pasó por debajo o sea nunca se percataron que no era chilena.
I: ¿Nunca se visibilizó?
P: Nunca se percataron que no era ahí chilena. Entonces claro ahí igual Hay una posibilidad de nacionalización por el interés del país. Pero lo que yo le dicho siempre eso es una decisión que tienen que tomar ellas pero que en una de esas de pronto nos... O sea ellas al ser chilenas van a hacer unas chilenas van a hacer unas chilenas pobres igual. o sea no hay mucha diferencia, Porque si tú miras quienes pueden acceder a una beca: los chilenos pero que digan que son pobres re pobres.
I: Pero clase media no tiene más
P: Las becas qué hay para extranjeros son una farsa. Cuando las niñas postularon al primer período yo tenía la ilusión ellas van a pasar. Cuando no y me pongo a mirar ¿cómo iban a pasar si las becas que hay son 100? 100 becas para todo Chile, para todas las carreras, para todas las universidades. Entonces eso viene siendo una posibilidad muy remota para hacer 100 entre

todos los migrantes. No tienen que ser... Yo creo que los entre los más pobres yo creo que se la rifa eso lo echan en una bolsita y sacan a ahí 100 y ya estos 100 son.

I: Bueno ya Muchas gracias

I: Yo quería hacer una pregunta si como del proceso En qué ha visto los cambios la ley y todo eso ¿cómo que con el primer mandato de Piñera como que muchas cosas salieron a la palestra, como el asunto que les hacían las entrevistas previas...? ¿Se acuerda de eso me puede hablar un poco de eso?

P: Si el filtro. El filtro que hace hoy en día lo que más funciona es eso. Cómo que eso no está... Como que el estado no lo reconoce Pero eso se hace porque yo conozco mucha gente. Bueno con el tema de las víctimas yo a muchas víctimas ¿y bueno y tú qué estatus migratorios tienes? No yo tengo definitiva. ¿Ya pero tú? No yo hice una vista sujeta a contrato no si yo fui allá pero sabe allá la señorita Me dijo: espero es que su caso no es para refugio. ¿Cómo así que señorita? No Ahí cuando me Atendió Yo vengo a solicitar refugio ¿pero por qué? No es que yo tuve un problema en Colombia a mí me mataron a mi papá, me mataron a mi hermano y nos habían amenazado. Ah no sí esos son problemas de seguridad su país pero no vaya ya consigue un contrato no sé qué no sé qué más. Mucha gente cuenta esa historia.

I: O sea el grupo de víctimas.

P: Sí Entonces ellos ahí me cuentan y mucha gente mi cuenta de eso este relato de hecho hace una más o menos dos semanas Conocí una muchacha y me dijo: sabe qué me llegó mi hijo le tocó venirse así tuve que juntar la plata como sea porque si no me lo matan. ¿Cómo así? Si imagínese a mi otro hijo a él sí lo alcanzaron a herir Pero él ya se fue para otro lado ya con él ya no pasó nada pero este que está menor ya le dijeron que tenía que irse y decir donde están hermano se moría. Entonces me tocó conseguirme plata y traérmelo ¿cómo así? Si ahí lo tengo en la casa a él le da miedo salir acá porque dice que acá hay mucho colombiano, ¿pero qué más hacía yo para dónde lo mandaba?... ¿y ya fue a solicitar refugio? Sí sabe me dijeron eso pero él fue y allá le dijeron que tenía que volver en unos 15 días ¿cómo Así? Sí le dijeron que volviera después o que mirará a ver si él se conseguía un contrato Porque si él quería trabajar acá tenía que tener un contrato.

I: ¿Y eso dónde es?

P: En extranjería

I: ¿En las oficinas de la Lautaro Ramírez ahí en el centro?

P: En San Antonio. Entonces yo le dije no. Como él ya fue una vez y él tiene ese antecedente que se haga acompañar váyase FACIS en FACIS lo van a orientar y van a... De FACIS lo mandaron a Diego portales y la Diego portales ya lo tienen en el programa y le van a hacer acompañamiento. Le van a hacer acompañamiento para presentar la solicitud lo van a presentar porque ya pasaron los 90 días del este. Y eso es lo otro que dicen: es que ya pasaron los 90 días usted ya está ilegal. Entonces el muchacho estaba muerto de susto porque el primero de agosto se le cumplían los 90 días y la muchacha le dijo si antes de los 90 días usted no ha resuelto va a ser ilegal en cualquier momento lo van a expulsar. De una vez le dijo si está ilegal a usted lo Pueden expulsar. Así que yo les dije No se dejen llenar de miedo vaya exponga la situación. No sé cómo le van hacer creo que le van a hacer el acompañamiento hacen un escrito tal vez la ayudan a redactar la solicitud para que la presente formalmente y le hacen ese acompañamiento. Entonces ahí estoy en pendiente de cómo evoluciona eso. Hubo un caso también de un colombiano que él se fue a Colombia y Cuando regresó tenía la cédula le faltaban

Cómo seis meses para qué se le venza Pero tenía residencia definitiva y era refugiado, y el funcionario de extranjería Ah no... Él entró al país fue a extranjería porque la cédula se le iba a vencer le dijeron a pero es que usted estuvo fuera del país algún tiempo no usted Es tan cédula se la vamos a retener ya lo que a usted le vamos a dar es una orden de expulsión. Pero él dijo pero como si yo soy refugiado. No eso no nos importa ¿cómo que no les importa? No nos importa. Entonces él les dijo el Carnet no me lo pueden retener ustedes no porque ustedes no son autoridades. En extranjería la funcionaria se lo iban a quitar en extranjería ahí en el escritorio. Le dijo No me lo puede quitar así la muchacha fue y le preguntó al... Y le dijo No se lo puedes quitar pero sí va a ver que a él le va a llegar una resolución no sé qué . Y él se fue a la Diego portales y Víctor Hugo lagos creo que fue el que le llevó el caso y cero le renovaron el carnet y su estatus está intacto.
I: ¿Los funcionarios tienen una actitud completamente arbitraria?
P: Sí súper, súper arrogantes y así súper dictatoriales Cómo que ellos ahí tu autoridad, para decidir.
I: ¿Y parece que no conoce la ley?
P: No y lo más divertido es cuando yo viaje, Yo podría haber viajado con mi cédula colombiana a Europa porque los colombianos entramos a Europa sin ningún problema, pero mucha gente me dijo Ese pasaporte azul no sirve para nada, esto nadie lo conoce, no sirve para nada solo sirve para problemas. Pero pues yo dije yo tengo que probar cómo es.
I: ¿Tiene otro color?
P: Si es azul y dice permitido para ingresar a cualquier país del mundo. Pero él refugiado Cómo desconoce tiene miedo no lo usa. Entonces la autoridades nunca tampoco lo van a conocer y en que el asunto es inclusive yo le dije a este funcionario de la PDI en el aeropuerto le dije: y me encantaría que como organización tuviéramos la capacidad de ser un programa de sensibilización y Partiríamos por ustedes, para mostrarle este documento y decirle que es que venga la autoridad que corresponde y les explique En qué consiste a todos los funcionarios están en esta ventanilla que no lo conoce. Porque el muchacho me dijo: ¿y esto y usted va a salir del país con esto iba a ir a Europa con esto? Con esto y esto es documento que a los refugiados se nos otorga porque nuestros países... Y nosotros estamos bajo el amparo del gobierno chileno y nuestro país no nos expide un pasaporte. ¿Ah no pero usted están como irregulares? Si le habló de refugio y todo y el tipo no tienen idea. Entonces me dijo deme un momentico. Entonces se fue hablar con el jefe y dijo: a no venga vamos. Y me llevó a hablar con el jefe y el jefe lo revisó no sé qué llamo, debe haber llamado extranjería y dijo no si Sí timbre le Ay mire vea yo no sabía de esto. Le dije imaginé sé qué interesante de verdad Y eso yo se lo voy a decir a Eli qué tal hacen según este que la gente de extranjería y los funcionarios de la PDI Qué son encargados en el aeropuerto, todo el que llegue al aeropuerto tenga que recibir esta información. Porque no es posible que un refugiado tenga que estar en oficinas explicándole el refugiado al otro En qué consiste el documento si es que son ellos los que saben y el documento cómo está hecho a mano, es un papelito es también muy diferente. También Creo que es por el tema de la seguridad de no tener esos papeles impresos en cualquier lado sino que lo maneja una sola persona el registro civil sólo una funcionaria maneja eso. Entonces yo Viajé con ese en cambio en París Tampoco sabía (se ríe). Pero el de inmediato no sabía pero si el jefe sí sabía. E inclusive yo llegué sin reserva de hotel porque no tenía reserva de hotel porque no me la mandaron nunca. Entonces yo le dije que usted que tengo reservado hotel

pero no me mandaron el papel. Ya Entonces ellos se averiguaron no sé cómo averiguaron pero me dejaron pasar sin problema. Pero fue un show la salida Entonces no lo conocen.
I: ¿Y la organización de refugiados articula después que ustedes llegarán?
P: Si en el 2008 empezamos a conversar y en el 2011, en 2009 más o menos ya era más o menos organización y FACIS nos ayudó a eso Elizabeth San Martín nos ayudó en eso no nos dejaban constituirnos porque nadie tenía definitiva cuando ya teníamos definitiva ya metimos a él este y en estación central nos permitió, porque lo otro que nos decían es que Todos viviéramos en la misma comuna. ¿Cómo hacemos para vivir todos en la misma comuna pues?
I: ¿Y eran sobre todo refugiados colombianos?
P: Si la organización de refugiados colombianos
I: ¿Existía ya antes la de peruanos?
P: La de peruanos lleva mucho tiempo sí. Y ahí en estación central nos dieron la posibilidad de armar la organización hacer los estatutos ahí y el funcionario que nos Atendió nos dijo coloquen todos la misma dirección, entonces no había que llevar certificado de domicilio de donde estábamos ni inventarla sino que todos pusimos la misma dirección sin ningún problema. Y nos aceptaron los estatutos y ahí tenemos la personería de estación central por eso.
I: ¿Y ahora no son tan activos en torno a la...?
P: En el tema de Refugio no estamos en ésa lucha porque hay cosas importantes ahora con lo de la Cumbre y lo que se puede venir para el tema de refugio, yo creo que van a seguir cerrando la puerta así de manera arbitraria y calladito. O sea no van a decir aquí no recibimos refugiados no somos deportados. Por algo la presidenta está conseguido los cargos que te ha conseguido es porque chile es país de acogida, porque Chile... Lo dijeron América, América Sudamérica Este ejemplo del tratamiento de los migrantes y a los refugiados en especial Porque chile es país de acogida. Allá se suponía que nosotros teníamos casa carro y beca por ser refugiados; nadie sabe que en un refugiado se la tiene que arreglar así a su modo. Ni los mismos funcionarios del ACNUR, los mismos funcionarios creen que aquí todo funciona de maravilla.
I: ¿La ACNUR de allá?
P: Los de ACNUR de allá. Por qué bueno ya me encontré con alguien de una memoria que me: dice yo te conozco a ti tú ibas a la vicaría en una reunión yo te vi hace muchos años. Yo sí. ¿Tú eres refugiada colombiana? Yo sí. La mujer No sé si es francesa no sé de dónde ella me dice: pero en Chile está perfecto todo. Y estoy en eso que nos reunamos todos que para pedir una reunión con Delfina Lawson, que entiendo que es la persona que está ahora encargada Delfina pues no conoce hace rato, ella no puede mentir porque sabe la situación de mucha gente y sabe lo que se vive acá. Entonces sería bueno eso porque hay que visibilizar eso.
I: Aparte si estando desestimulando y haciendo...
P: Y lo que pasa lo que otro es que evidentemente los colombianos necesitan todavía seguirte viendo Refugio porque las cosas en Colombia no están bien. De hecho la guerrilla no toda negocio hay algunos frentes que no negociaron Y esos frentes están activos ELN todavía no negocia Por qué la mesa la tenían en Ecuador pero la tuvieron que trasladar a Cuba por los mismos temas de seguridad. Y ellos nosotros estuvimos en Ecuador en un encuentro de víctimas del conflicto armado y nos reunimos con la mesa de ELN los que van a negociar y ellos lo dijeron inclusive ellos tratan súper mal a los refugiados. No quieren a los refugiados porque para ellos Nosotros somos como las ratas que abandonan el barco.
I: Sí como traidores.

P: Ellos te dicen nuestros Defensores porque ellos se suponen que actúan para defender el pueblo colombiano. Pero nosotros tampoco nunca van le hemos pedido que nos defiendan de esa forma y yo no me siento representado por ninguno de ellos, o sea no y ellos fueron muy claros: la negociación va a ser en la medida de ellos y Sí o sí con la participación de todas las instancias de la sociedad civil, hasta inclusive nos dijeron a los colombianos que se encuentran viviendo en el exterior. Entonces nosotros existimos hasta los refugiados hasta las víctimas que vivimos en el exterior. No hasta todos los colombianos que vivan en la exterior; ellos los refugiados y víctimas no lo reconocen porque reconocer que somos víctimas del conflicto armado y ellos son agentes al igual que el estado entonces también somos Víctimas de ellos. Entonces ellos dicen que no negocian bajo las regla del Estado si no las bajo las reglas de ello y con la participación. Entonces cuando dicen con la participación ahí nosotros tenemos que participar, Porque si nosotros no participamos ellos tampoco ellos van a negociar. Entonces puede que en Colombia haya sociedad civil de víctimas pero nosotros no nos podemos quedar por fuera, porque se supone que nosotros algún momento la gran mayoría quiere regresar y nosotros tenemos que ayudar a construir al país que queremos volver. Entonces si no participamos no hay nada, entonces ahí en eso estamos en hacerle conciencia a la gente que todo nos toca con los mayores muchos evalúan el tema del retorno de todo que se ponga las pilas y que nos integremos Porque la única forma de exigir es estando Unidos porque si no estamos Unidos no, y también dejar la puerta de los que necesitan entrar acá porque hay muchos que van a necesitar entrar formas el gobierno que se montó ahora en Colombia terrible terrible terrible. Porque aunque todavía no se sabe nada al que se pueda Cómo concreto, pero lo que ha estado pasando que no los muestra los medios, cuándo matan de a dos ya es una masacres.
I: Las muertes de los líderes sociales.
P: Después que ya Matan a dos juntos eso ya son masacres y se están presentando ya, hay líderes que están viviendo con Escolta eso no puede ser que una persona en el campo tenga que andar con Escolta, porque están amenazados, Mira nosotros tenemos formamos partes de la red... es arreglamos entre todas las víctimas de América Latina de las del encuentro y ahí llegan las copias de los líderes mandan las amenazas de las Águilas negras de no sé qué y lo ponen con nombre propio: Estamos dándole un ultimátum a Pedro Pérez que si no abandona el país en menos de un mes va a ser objetivo militar y Nosotros sabemos dónde vive su familia nanananananana. Así de terrible el que está en la lista olvídese que la lista al cajón esos terror. Así que chiquillos están en un paraíso ustedes.
I: Muchas gracias.
P: Bueno no cualquier dato adicional que necesiten y algo y cuando salga el estudio mi cuenta.
I: Nosotros tenemos la idea que cuando tengamos las entrevistas y los resultados vamos hacer un conversatorio en el FACIS.
P: Chévere.
I: Para poder exponer qué cosas están repitiendo Cuáles son las necesidades Porque todo lo que estás contando se refleja mucho con lo que están diciendo los equipos de salud, que en el fondo ellos no tienen instrumentos que llegan las personas en esta situación que a las personas se les desincentiva a pedir refugio, que no es lo mismo que las personas que están en desplazamiento de este tipo que las personas que han planeado su migración. Entonces hay

una serie de hechos que no se están cumpliendo y queremos también reveló también a un nivel de política también poder ponerlos en las redes sobre diálogos de derechos importantes.
P: Y no y bueno y las patologías cosas que la gente no sufría en su país de origen las empieza a sufrir acá: por ejemplo para nosotros el estrés no existía y la depresión no existía. Hasta que yo llegué y licencias por depresión yo no sabía que era una licencia Cuando alguien estaba por licencia por depresión estaba en un sanatorio de tratamiento psiquiátrico. Yo conocí como unos dos casos de personas que tenían trastornos mentales que empezaron con esto era una depresión y los tuvieron que internar pero como que en el trabajo te encuentras un compañero llegué de licencia ¿y porque tenías licencia? No es que estaba con depresión y yo tuve personas a cargo y yo nunca le Recibe un trabajador una licencia por estrés o por depresión, empezar a familiarice con eso y eso se pega.
I: Esa es la epidemia que se encuentran aquí.
I: Muchísimas gracias.
P: (inicio de audio B) a veces no salgo en todo el día ustedes me verán en la casa en la cocina.
I: ¿En qué está trabajando ahora también en prevención de riesgo?
P: No yo a veces hago asesoría en prevención pero ya lo hago como voluntariado porque esto es una farsa, no quise ejercer más porque yo me ilusioné cuando conocí la carrera y todo y empecé a estudiar y yo lo veía como Hay que rico: evitar que cualquier trabajador se vaya a enfermar.
I: Seguridad laboral y todo eso.
P: Cuando yo trabajaba en la mutual me encontraba con que yo visitaba a las empresas a revisarle cómo tenían las empresas el tema de seguridad en el trabajo y yo iba revisar a los prevencioncitas de las empresas y ellos ahí me contestaban: de verdad Qué elementos de protección están. ¿Pero porque están firmado que están? yo le decía Bueno vamos a la bodega. Quiere que le diga la verdad Ahí dice que los implementos... No tenemos bodega (se rían) ¿Pero cómo? Honestamente Yo tengo un hijo y yo firmó o firmo. Hay una cosa que llaman negligencia inexcusable que es que: tú te tiraste a la máquina para que te atropellaran para que te pagaran.
I: Como en China
P: Y muchos dicen yo esté accidente la verdad yo tuve que pelear la negligencia inexcusable o lo lograba o me echaban. Y yo decía Yo estudié para ir a firmar al empleador la sinvergüenza y la estas, a decir qué el trabajador tuvo la culpa, Qué es que se quiso cortar el dedo, a decir que tienen los elementos de protección de trabajo cuando no les ha dado la gana comprarlos y arriesgar que una persona se muere y si es que no lo había pasado el informe, me vaya yo a la cárcel por negligente. ¿Y tú sabes que hacen? ellos hacen por ejemplo yo pongo sobre aviso que si no se ajusta a esto lo otro lo otro lo otro... Hay empleadores que le dicen saben qué tiene dos opciones: o por el contrario usted escribe que todo está correcto y me lo deja firmado o se va. Porque yo esas cosas no las voy a comprar: yo no voy a cambiar eso zapatos por otros, Yo no los voy a cambiar Los cascos, yo no lo voy a comprar... ¿para quienes voy a comprar más si ya les comprado dos veces más? Hoy no no no como dueños de fundo Así que no no no no no yo no voy a comprar nada, nada que sea gasto ¿y cómo hace para hacer prevención si esas mascarillas se dañan y el filtro de Carbón se agota en determinado tiempo? No les compre mascarillas el mes pasado. Pero las usan todos los días y se agotan porque tienen químicos agresivos. Entonces uno estudiar para ir eso es como Bueno yo digo; cómo los abogados que sabiendo que el tipo es violador y todo y defenderlo Y tratar de sacarlo igual. Y al menos tiene Claridad

de la situación iba a buscar los argumentos legales para sacarlo, pero cómo mentir yo trabajar en eso antes no. Cuando alguien necesita un plan de emergencia y algo Si yo le puedo ayudar, la ayudó los asesoro y todo pero no me dedico a eso. Y además que ahora hay un prevencioncita en cada esquina también ofrece nada de sueldo.
I: Claro porque también con eso hay una competencia desleal.
P: Yo hago Network marketing desde el último empleo van a hacer dos años que Me despidieron, hace un año y medio que Me despidieron era encargada de administración y finanzas de una importadora. Quiero una empresa que empezó para atrás y para atrás y para atrás, no se dejaron asesorar: yo les decía hagamos esto hagamos lo otro y ellos pequeños empresarios sin experiencias no sé dejaban asesorar, no tienen Con qué invertir más y se requiere invertir mucha plata. Entonces en un momento a pensar nos queremos mucho Nos apreciamos mucho pero nos toca hacer nada y chao. Así que chao cerraron y me cerraron el tiro y yo pa mi casa. Así que ahí yo empecé a mandar currículum porque ya había cumplido los 50, todos los empleos son hasta los 42, hasta los 45. Así que yo ya tenía un código de Network tenía un código inteligente y ahí empecé a estudiar, qué tal Sí sí porque uno no cree mucho en esas cosas no ¿cómo y siente que consiga trabajar desde su casa? ¿Cómo es que hay cosas que hacer y que no sé qué yo? ¿Será verdad? Bueno hoy en día vivo de eso Entonces ya manejo mi tiempo ya todo es muy distinto. Cuando se presentó el viaje para esto si hubiera sido en un empleo yo no pudiera ir. Ya porque de hecho me pagaron una parte pero la otra parte la Tuve que poner ¿pero cómo yo en un empleo puedo sacar para eso? El tiempo y plata para ir no se puede, cosas que no se pueden en el mundo laboral, ahora ya conocido eso. Cuándo tengan tiempo muchachos me avisan y los invito a una charla para qué hagan por los laditos. Hay un movimiento de jóvenes y eso porque el panorama laboral no está fácil, con el estatuto joven. Para los grandes es tan malo y para los jóvenes Está peor así que ahí hay cosas que hacer....

Entrevista 02- COLOMBIANO código 02.

I: Bueno, estamos… Señor X y usted ¿hace cuánto tiempo llegó a Chile?

P: Ya ajusté… un año. Hace ya un año larguito… El cuatro de este mes precisamente, estaba ajustando el año de haber ingresado acá al país de Chile…

I: Y… y ¿Cómo era su vida? Como… ¿Llegó al tiro…? ¿Cómo se puso en contacto? Como…

P: A ver… primero que todo, o sea, lo mío ha sido… ha sido como de emprendimiento… de tratar de crecer, de avanzar, de hacer otras cosas… Porque en el país… en Colombia de donde me vine, teníamos problemas allí de… de problema social, or la parte económica, yo tuve allá negocios y… y nos extorsionaban… No sé acá como se dice…

I: Sí… extorsión… extorsión.

P: ¿Sí? Que los llaman o… le exigen montos de plata mensuales que tienen que dar a ellos y entonces… eh… económicamente nos quebraron, o sea… Llegó un momento en que nosotros no pudimos ya… aportar más y entonces… Eso hace más o menos cuatro años atrás… Y entonces yo ya viajé con mi esposa en el última cafetería, en Colombia una cafetería es como una especie de… de sitio donde se come a las once… O… como variedad de comidas, de mecatos, cosas así es más o menos una cafetería.

I: Claro…

P: Eeeh… mi esposa y yo trabajamos casi doce años en dos que teníamos, ya luego que vinieron estos problemas sociales eh… con bandas delincuenciales entonces… En un trascurso como de dos años eeeh.... pasó toda esta parte de que nos quebraron y… mi esposa y yo pensamos en emigrar siempre a otros países a buscar nuevas opciones, nuevas posibilidades de vida… Ya pues que viendo el problema social de nosotros en Colombia… ha, ha sido tan fuerte y como es basada en la corrupción es difícil de que cambia como la idea… la idea social o de los presidentes o de la oligarquía, que… Que cambien la forma de ver, de pensar, sino que ya tienen todo un esquema montado… donde siempre ellos a través de la corrupción van a… van a beneficiarse en ellos como… como entidades… políticas, máquinas políticas y no van a estar prestos atención a lo que sufre la gente… la sociedad en el común no les interesa. Entonces, viendo pues el esfuerzo que hicimos mi esposa y yo allí trabajando, para tener algunas cosas, el bienestar de la familia, y ya después todo derrumbarse… Salimos primero a Panamá, estuvimos en Panamá…

I: La ciudad de Panamá…

P: Sí, la ciudad de Panamá. Estuvimos allá… mmm… mi esposa estuvo siete meses conmigo, luego se fue a Colombia de nuevo. Yo me quedé (ininteligible min 03:05) a… cuando en eso estaba el presidente Martineli y entonces… Por el crisol de razas era que llegaba uno… cuando cumplía un año de estar allá ilegal, pues porque uno entra y está tres meses legal, pero ya luego se queda ilegal y… Entonces eeeh… en ese tiempo, cuando ya fui a cumplir el año, habían unos proyectos, porque ya teniendo papeles, identidad allí, era más fácil de uno… poner ya un negocio y…

I: ¿En Panamá?

P: En Panamá… Pero resulta de que cumpliendo el año, ya estaba con toda la papelería todo listo para entregar. Pero… en… en ese entonces ese presidente, pues que hace poquito salió Santos, habló públicamente de que Panamá era un paraíso fiscal para… para Colombia, o sea, un lavado de dólares activos, que eso es verdad… Pero él lo dijo públicamente y entonces allí mismo el presidente tomó cartas en el asunto de Panamá, y empezó a todos los emigrantes el… eh… Esa publicación la hicieron en la tarde y al otro día en la mañana ya estaba… emigración por todas partes…

I: Tuvo una consecuencia política hacia los migrantes…

P: Total, total… A todos los emigrantes de todas partes nos empezaron a sacar así de Panamá… Yo fui uno de ellos, yo estuve quince días más o menos eh… tranquilo después de ello. Pero a los quince días igual también, saliendo del trabajo… Yo trabajaba en el… hotel Donald Trump de Valle Parque, allí conduciendo un carro, dentrando, parqueando los autos. Y cuando salí… cumpliendo un horario de trabajo… ahí me interceptaron unos policías de migrato… emigratorios de Panamá y estuve casi quince días detenido, ya yo tuve que comprar el pasaje para volverme a… a mi país, porque yo decía, bueno y… y este tiempo mi familia necesitan de que yo les mande dinero y no puedo hacerlo. Entonces había, me aligeraron y yo compré el pasaje y me volví a Colombia… Acepte… de unas clausulas que ellos tenían ahí formalmente ya estudiados… no sé de donde sacaron, total de que yo acepté de todo ello, con tal de que me dejaran regresar rápido a mi país, para yo poder seguir funcionando…

I: Cuando me… Discúlpeme una cosa, dijo eso de…

P: Si…

I: Dijo eso del crisol de razas… no me quedó claro…

P: El crisol de razas es como… como… un acuerdo que había allá, no sé si todavía lo hay. Que lo había en el momento y era que cuando el emigrante cumpliera allá un año, al año ya podía hacer… a través del crisol de razas que hacían a mitad de año. Y a fin de año, de los que cumplían el año, a mitad o a principio de año, podían acercarse con la papelería requerida y ya empezaba el proceso de… de dejarlos estar allá por un tiempo, mientras le daban la nacionalidad.

I: Claro… Era como una facilidad…

P: Sí… Bueno, entonces después de ahí con mi esposa, estando también en Panamá emigramos a… a Costa Rica. Y en Costa Rica pues nos pasamos y… ilegalmente…

I: … Pasaron por…

P: … Por Panamá mismo, por un puente que hay allá… que es solamente un puente y ya, se comunica la frontera. Nosotros nos….

I: … (Ininteligible min 06:33)

P: ¿Ah?

I: ¿Por el Atlántico o el Pacífico?

P: Por… el Atlántico… por el Atlántico…

I: Ah… Ya.

P: Sí, y entonces fuimos allá también mirando la posibilidad de trabajo… Pero… Nos volvimos a Panamá porque estuvimos andando y vimos que no… no era tanta la posibilidad. Pues, optamos por Panamá… Pero ya luego, eso fue como en el proceso de estar allá… Ya luego regresamos a Colombia… Yo regresé como… como rechazado, y ya me puse a trabajar otro tiempo, pero ya en Cartagena, que es una ciudad de…

I: …Sí, sí…

P: Yo soy de la ciudad de Medellín…

I: Usted es de Medellín

P: Sí, y la ciudad donde luego me fui a trabajar taxi, como taxista, a conducir, bueno fue en una ciudad de Cartagena que es turística, porque yo además por la parte que le comentaba de extorsión no podía estar con mi familia…

I: No… no podía estar en Medellín…

P: En Medellín… Ni en mi hogar ni nada. Entonces… yo estuve casi un año también allá… Ya luego… Mmm, ya pues me aburrí después de tanto tiempo fuera de la casa, los hijos, y todo ese proceso de no ver de ellos el crecimiento… uno sin poderlos… estar con ellos… Ellos se… debido a este problema social en Colombia… dejaron… Hasta ahorita llevaban casi cuatro años sin estudiar, ahorita están estudiando acá en Chile.

I: ¡Ah, volvieron! Llegaron acá…

P: Sí… Y entonces ya… de allá, cuando o llegué de Cartagena a Medellín eh… Como que no sé, esa gente como que se da cuenta o lo ven a uno, quién dice, no sé que pasó… Y como a los quince o veinte días… eh… en un centro comercial yo fui interceptado… yo ahí mismo vi la malicia, que me iban a coger o algo… Y yo me volé…

I: De Cartagena…

P: De Cartagena… No, no de Cartagena no… De Cartagena regresé a Medellín, a allá en Medellín me sucedió ello y entonces ya me volví a ir a otra ciudad de Medellín. Después mi familia fuimos también a... en turbo, que esa fue otra forma como de alejarnos de Medellín para tranquilizarnos….

I: Claro…

P: Y después de allá… ahí si, logré venirme para acá, porque ya al último quedé encerrado, en que no tenía la parte económica si quiera para viajara ninguna parte… Mi familia pasando todas las consecuencias económicas, y un señor que le administré una ferretería emmm… En el Bajo Cauca, que viajé también allí en Colombia como… cuatro o cinco meses, este señor al fin me… me… me dijo que me prestaba la plata, me compré el ticket con su tarjeta de crédito y pude viajar acá a Chile… Entonces viaje acá a Chile y… y acá en Chile estuve dos meses… solo, pero entonces yo vi que hay mucha posibilidad, hay mucho que hacer y entonces… Hay posibilidades de trabajar y todo esto… Y emprender que, en la vida después de nosotros… d e mi esposa y yo…y entonces les

dije que se vinieran, mi esposa vendió los enceres, muebles, televisores, todos los enceres en Medellín. Y con esa plata empezó a viajar, yo sabía que con esa plata no alcanzaba a llegar hasta Chile, pero… algo… yo hacía para que funcionara que ella acabara de llegar. Y efectivamente llegaron hasta Perú… hasta Lima Perú.

I: Mmm…

P: Y en Lima Perú ya… ya ellos se quedaron allí casi cuatro días y yo averiguando como hacía... Allí aparece la fundación FASIC.

I: Ah, ¿se contactan desde Perú… o acá?

P: No, yo ya estaba acá… Y entonces yo ya había empezado a asistir a una trabajadora social anterior que se llama Y, una señora con una calidad humana… fuerte, grande eh... me atiende y se empieza ella a poner al tanto del caso, se dio cuenta cuando mi familia empezó a viajar… Y… y ya pues por medio de FASIC eh… una plata que me dieron para… para un arriendo… no sé si fue por parte de ella que… que me dio como otra platica para que mi familia pudiera acabar de llegar… Lo cierto fue que… se dieron las cosas y…

I: … Y pudieron llegar…

P: Y pudieron llegar… mi familia, después de cuatro días de estar en Perú… Yo agregándoles a mandar para… para quedarse en un pequeño… una pequeña habitación de motel… Que allí les, un señor les favoreció. Y entonces llegaron a los dos meses precisamente de yo haber estado acá. Y los presenté a la fundación FASIC, estuvimos acá, y empezamos a hacer de nuevo los mismos papeles que ya…FASIC me había asesorado para yo hacer a través del refugio político. Y acá estamos… acá estamos en el momento, como te cuento ya llevo más del… ya llevo un año y díitas acá en Chile… Averiguando como se funciona… Y… casualmente hace por ahí un mes, mes y medio hacía acá se nos apretó de nuevo la situación económica…

I: Ahá…

P: Totalmente. Porque nosotros al principio como… como refugiados y mientras se presentaba todo esto de papelería… no nos daban trabajo… por no tener documentación ni nada en la casa. Entonces empezamos a trabajar de cuenta de nuevo nosotros, a vender yogurt con cereales en unos tarritos plásticos higiénicos, en un carrito de supermercado que conseguí y lo adecuamos y empecé… así a trabajar ambulantemente en la calle…propiamente aquí en la… en la… en la estación de… de República…

I: Ah… oka…

P: Por la salida norte del metro, al lado de la iglesia, de la fuente. Y así nos empezamos a trabajar y… y a raíz del esfuerzo y… de mi esposa, estaba por la mañana y yo en la tarde. Luego pusimos otro carrito también que vendía en la tarde con sopaipilla… Por el tiempo de invierno… Bueno, empezamos a sostenernos y allí nos sostuvimos mucho tiempo y entonces…

I: ¿Cuánto tiempo estuvieron desde…? ¿Cuándo empezaron con este emprendimiento?

P: En la venta ambulante y con el emprendimiento… desde que yo llegué aquí a Chile me empecé a hacerlo… Porque de hecho yo llegué… Yo llegué primero a… a Pucón…

I: A Pucón… ¿Y llegó a Pucón?

P: Sí, porque a poco había una seño... una muchacha amiga de nosotros en el barrio en Medellín, que ellos trabajaban… nosotros trabajábamos la parte de la comida y ellos trabajaban la parte de... de… de ¿Cómo se llama? Se...

I: ¿Mucama?

P: No… como receptarios de… de drogas para enfermos, droguería… ¿Cómo se llama?

I. ¡Farmacia!

P. Farmacia, exacto…

I: Ah, ya ya.

P: La parte de farmacias tenían… Entonces éramos amigos y ella había viajado antes acá… Y por ella fue que vine, y entonces ella me recibió allá en Pucón. Viajé hasta Pucón, pero entonces allí me iban a dar un trabajo los patrones de ella y no se dio. Resulta que… de que ellos habían viajado a Santiago a un tiempo de vacaciones que, porque allá estaba muy quieto, por que es por temporadas y la economía…

I: … Claro… se habían ido de vacaciones y allá no iban a….

P: No iban a estar. Entonces yo llegué en el momento en que ellos no estaban, pero que esperara quince días, que ellos regresaban y que me empleaban. Pero yo no podía esperar quince días, porque yo necesitaba moverme, mi familia… para mandar algo ¿Cómo hacía? Aunque en realidad, mmm hasta que ellos llegaron aquí y nunca les pude mandar nada y ellos allá pasaron muchas necesidades fuertes… pero bueno, eso es parte de la vida. Y… luego de estar allá como cinco días… en Pucón, viajé a Viña del mar, porque vi que allá no había pues la forma de quedarme… mucho tiempo estuve buscando por todos lados, nadie me empleó.

I: ¿En… en qué época llegó? ¿En qué época del año? ¿Marzo, abril?

P: Eeeh… No sé… estábamos en septiembre…

I: … ¡Ah, llegó en septiembre!

P: En septiembre del año pasado, el cuatro precisamente.

I: ¡Ah! Disculpe, disculpe, disculpe…

P: Tranquilo…

I: Ya jaja…

P: Y entonces luego de cinco días de ver que no había trabajo, me vine a Viña del mar, con unos currílulos, a regar allá por ser una parte turística, en un hotel una persona me dijo que por más que regara no me iban a emplear en ningún momento, por no tener documentación… Entonces bueno, entendí un poco. Ya ahí al ver que no había nada viajé aquí a la capital a Santiago, llegué a la terminal de buses… me tocó pasar una noche allí muy fría además… que casi… no, pues que no dormí porque el frío no lo deja dormir a uno. Luego pasé a un albergue, estuve en un albergue…

y… estuve en la calle, estuve en la calle inicialmente… Eh… pasando muchas necesidades, aguantando hambre, frío, sin poderse uno bañar, nada… Bueno, y así estuve como cinco días hasta que ya llegué a un albergue… y el albergue… ahí estuve viviendo como dos meses más o menos. Hasta que ya pude recaudar una platica y.… ya cuando… o sea, se dieron las cosas como casualmente cuando iba a llegar mi familia acá, yo ocho días antes había reunido la plata, la mitad de la plata, para poder dar donde iba a empezar a vivir… Que eran cien mil pesos… y lo logré re… al… o sea, de la nada los pude…

(suena teléfono)

I: Conteste no más señor …

P: ¿Sí?

I: Sí, no hay problema

P: (Contesta el teléfono) Buenas tardes, sí, con él. Sí, ya le informo a mi esposa para que salga un momentico, que en la casa está siempre adentro y tal vez no le escuchan de ahí. Yo ya le digo a mi esposa para que salga. Listo, bueno señor.

I: Llame a su esposa entonces…

P: Un momentico

I: Después le ponemos… retomamos.

P: Perfecto, un momentico…

I: No, tranquilo. Voy a poner en silencio…

P: Precisamente la señora de direct tv a retirado porque se nos canceló… no hubo con que pagar los servicios de parabólica y de... de internet, entonces va ir a retirar el móvil y eso…

(Se escucha ruido ambiente, niño hablándole a padres, ruidos)

P: (Marcando el teléfono, no contestan) Como mi esposa está ahí en la casa, es una casa grande de varias habitaciones, (ininteligible) y no lleva celular…

I: Pero intente no más… da lo mismo… Si estas cosas… yo siempre digo, siempre… Después no transcribo esta parte, no tiene ningún sentido…

(Intenta contactar a esposa y no hablan de entrevista, min 16:12-19:58)

I: Eh… Bueno, entonces estaba contando en que estaba trabajando… logra reunir este dinero para que llegue… y justo llega su familia

P: Exacto… Ocho días antes que pudiera co… juntar los cien mil pesos para que llegara la familia y llegaron a la habitación. Y ahí es donde estábamos… en una habitación, hasta el momento estamos ahllí todavía.

I: ¿Y cuantos hijos tiene?

P: Tres.

I: Tres

P: Ahá. A M de 18 años, V de 16 y E de 14.

I: Una sola niña

P: Sí

I: ¿La regalona?

P: Hace… No… todos

I: ¿Todos son regalones?

P: Sí… los tres son deportistas de alto nivel. Ya en Colombia en el fútbol los dos muchachos eh… el de 18 lateral y el otro, el de 14 es arquero.

I: Mmm

P: Jugaban en el atlético nacional, en las reservas.

I: Aah…

P: Pero al llegar aquí… resulta que aquí nos dimos cuenta…

I: (Ininteligible)

P: No, no… o sea, manejan… manejan una mentalidad todavía de que no, primero lo de acá y luego lo de afuera, no importa el talento…

I: Mmm… no…

P: ¿Cierto? Entonces de… de hecho la ANFA acá, que es la parte que da las leyes de fútbol y todo eso nunca para el extranjero, así sea de buen… dicen que tienen que pasar cinco años estando aquí para poder empezar a jugar los torneos de ellos… Entonces pongamos, el hijo mío de 14 y el otro… de 14 y el de 18, mientras pasan cinco años en verdad se les pasa el tiempo a ellos de hacerse profesionales…

I: Claro… de estar en el torneo… al nivel de…

P: Entonces el hijo mío, el de 18 regresó a Colombia, hace como tres meses ¿por qué se fue? Porque vio que aquí no habían las posibilidades del fútbol, por la misma negligencia… por las expresiones de pronto cambian de país a país, pero de pronto con la negligencia de acá todavía tienen la mente encasillada, pienso yo, en pensar solamente en lo de acá y no en el talento que pueda apoyarle y tener más variedad, más posibilidad… al tener otras personas, entonces…

(Suena teléfono)

P: permiso (contesta) ¿Aló? Amigo, no… no le… ella como sería que salió, pienso yo, porque no me contesta… Y yo salí, yo estuve esperando hasta las tres, que usted quedó de llegar de dos a tres… Exacto… si usted quiere programa para mañana de nuevo, para que estén más seguros. NO yo estaba allá, pero usted no llegó a tiempo, entonces yo tuve que salir. Exacto, entonces allí no tengo yo problema, si usted hubiese llamado más antecito, yo ahora estoy cumpliendo otra cita,

entonces si usted me dice a que hora llega, me llama con tiempo, entonces yo hubiera programado diferente, pero como no me llamaste entonces me tocó salir. Pero si quiere programamos pa mañana de nuevo, porque mi esposa parece salió. Precisamente se quedó allá pensando en que usted llegaba, pero no… de pronto salió. Son las tres y veinticinco en mi reloj ¿Qué horas son en su reloj? Ajá, ahí la única opción es programas para mañana de nuevo o más tarde, yo por ahí en una hora regreso de nuevo. Si de pronto está en el mismo sector, por ahí en una hora estoy de nuevo allá en la casa… (sigue hablando por fono hasta min 24:26)

I: Entonces… Se fue su hijo a… a Colombia

P: A Colombia si…

I: Y… ¿se va a reintegrar al atlético?

P: Mmm… No se reintegró porque ya es como… se había salido, no se pudo reintegrar a mismo club de fútbol, pero está en uno de un municipio que se llama de hecho… qué está bien bueno y saca mucho talento, y está entrenando allí… Y está contento, porque está haciendo lo que le gusta, de hecho, nosotros a ellos desde pequeñitos en Colombia, desde los cuatro cinco años, a los tres, con mi esposa y yo desde que nos casamos decidimos, por el ambiente social del país, de que los hijos de nosotros íbamos a encaminarlos desde pequeños a una actividad deportiva, para que cogieran disciplina y… y por el deporte, para que… empezaran de pronto a fijar unas metas, unos sueños, que los tienen… para quitarle como… tiempo de que estuvieran en la calle… o en rumbas o en cosas… Mientras que el deporte les lleva a la disciplina de estar haciendo deporte, una vida más sana. Y lo logramos, lo logramos están grandes y… aplomados aquí, los conocen en la institución, aquí estuvimos con ellos… Y son personas muy manejables, acá está V, está E y los dos estudian, V y E, V hace modelaje, es una niña pues además que es bien linda, hace modelaje, que lo hacía también en Colombia… Y E sigue practicando fútbol en Maipú Unidos…

I: … En Maipú unidos…

P: Sí… Y, pero el problema sigue siendo latente y estando ahí, de hecho, el entrena toda la semana… Eh… desde principio de año ha entrenado y ahora llegó un muchacho nuevo a.. a, de arquero…

I: Mmm

P: Él tiene muy buenas condiciones, pero por el otro ser de acá… y… y así hace poquito llegó, lo convocan a los partidos y al hijo mío no… Ahí mismo se ve la diferencia…

I: … Aaah… Ahí se ve la cuestión (se acoplan las voces 26:30 min)

P: Sí… ahí mismo se ve el regionalismo digámoslo así. Pero igual… el propósito de que el hijo de nosotros regrese de nuevo a… por ahí en febrero, en enero o febrero a Colombia a… a realizar su sueño sobre el fútbol, que ellos están viendo que aquí no hay posibilidades y… y entonces les decimos es que se entrene para que haga las cosas lo mejor que pueda, para él porque…

I: … ¿Acá ellos están en el colegio…?

P: Sí, están en Amunategui… Amuna…

I: Amunategui

P: Eso… allá estudian, pero hubo un problema de que les incendiaron una parte… un sala, y por eso el presidente se quiso escudar para arreglar y cerrar el colegio y quitar la propiedad… Y no han podido, porque los papás y otras personas que han salido, que es un colegio emblemático de acá…

I: Si po…

P: Y entonces lo dejaron y… todos acudieron… y nos unimos porque fuimos a hacerles sentir pues que no se podía cerrar el colegio, entonces dieron otra cede y ellos… es… y un transporte que los lleva allá y vuelve y los trae de otro colegio…

I: ¿Y… y como llegaron a ese colegio…?

P: Yo fui, por medio de la institución…

I: ¿De FASIC?

P: De FASIC, me dijeron donde ir a pedir un rut provisorio, porque como no tenían todavía carnet, me dieron un rut provisorio y ellos pudieron empezar a estudiar… Y ya ahora ellos tienen cada uno su… su carnet. Y ya ahí estamos…

I: Aah… Disculpe, Carlos…

P: Sí…

I: ¿Usted es solicitante o refugiado? ¿Como? ¿Ya está en proceso de solicitud?

P: Yo estoy en proceso de refugio

I: Ah…. De solicitante, ¿ya formalizando la situación?

P: Sí, (ininteligible 28:12) Todo lo que yo comente, lo que te estoy contando, lo conté también allá con una trabajadora del estado… Y… y dicen de que por ahí en dos años, o de dos años a tres años nos dan, si nos quedamos o si nos dan… eh, ¿Cómo es? La permanencia como por cuatro o cinco años… O la rechazan, entonces estamos como en ese proceso…

I: Y entre medio, todavía le dan esto de la visa de ocho meses…

P: Exacto…

I: Como que ya la reactivado varias veces…

P: Una… vez

I: Ah… una vez, si po cierto…

P: Ya llevo un año, entonces ya me la volvieron a… a reactivar, entonces hasta marzo del próximo año…

I: Mhm…

P: Y ahí vamos poco a poco…

I: De a poquito… paso a paso

P: Si…

I: Pero que bueno lo de… sus chicos, que sean tan empeñosos

P: Sí… deportistas y son personas con… con muchos sueños, muchos ideales y… y buenas costumbres, son muchachos manejables en la casa… muy sanos… De hecho, con la disciplina del fútbol, como te digo, no se interesan tanto por un fin de semana o a salir a rumbear o a trasnochar… no, siempre están en la casa…

I: Jeje, son como hijos caseros

P: Sí, es algo para uno como padre, bueno, porque ellos tienen sus metas y sus sueños… Entonces ahí estamos, en esa parte.

I: Y… ¿Cómo ha sido el tema de la salud acá? ¿Ha tenido…?

P: Eh, nosotros al principio nos hicimos por medio de Fon… ¿Fonasa?

I: Mhm…

P: Fonasa… Nos hicimos suscribir, de acá también nos dijeron como hacíamos, fuimos allá también con una trabajadora social. Pero hace como dos meses la hija mía V, tuvo un dolor como de espalda o algo… Y fue con mi esposa a que la atendieran… y no la atendieron, aparecía como… vencido, no sé…

I: ¡Ah…! (Ininteligible, se acoplan voces min 30:10)

P: No…

I: La inscripción…

P: La inscripción había como que… vence, como cada seis meses… no entendí como bien mi esposa, entonces había que ir de nuevo como a obrar…

I: Y… y eso ¿en qué comuna fue?

P: En Santiago centro, donde estamos nosotros, estamos aquí en Cueto.

I: En Cueto… Y ahí tuvo, entonces ¿no la pudieron atender?

P: No… no nos pudieron atender y entonces, lo que más… lo que más… ¿Cómo es su nombre? perdón

I: Nazzim

P: Nazzim… Nazzim, lo que nos ha dado más duro y nos ha movido mucho, es la parte económica de desde hace por ahí un mes… un mes larguito hacia acá, que como en República no se nos dio lo de los puestos que teníamos trabajando, porque… como nosotros por, pues colombianos atendemos bien a la gente, y somos pendientes…

I: Claro

P: En ese sentido… entonces la gente se sentía bien atendida y acudían mucho (ininteligible, se acoplan voces min 31:00)… A atenderlos…entonces los mismos… eh… ¿Cómo dicen? Comerciantes del lado… y por una señora que chilena que empezó a hacer muy mal ambiente… Que nosotros qué que teníamos que estar haciendo allí… que colombianos, que quitándoles clientes… Empezó a armar una mala ambiente… Y entonces empezaron a tirarnos carabineros y nos hicieron, nos quitaron los carritos, nos volvieron a… Nos quitaron lo que nosotros habíamos hecho un proceso de casi ocho, nueve meses trabajando y logrando avanzar… Ahí nos tiraron todo... Nos quitaron, entonces ahí nos quitaron un carro con surtido de comida y todo, entonces empezaron… Ya luego nos fuimos para allá más para abajo, a otra estación, y nos quitaron también otro carro, al final nos quitaron dos carros y… Había hecho… un señor me estaba dando unos huevos para yo empezar a trabajar, volver a salir adelante…

I: Mhm…

P: Y ayer en la noche me quitaron otra vez el surtido de huevos… Yo tengo un triciclo moto…

I: Mhm

P: De esos que son eléctricos, los chinos, y me sacaron la mercancía de ahí… todo…

I: Los… ¿Los carabineros?

P: Los carabineros… Y ahí quedé otra vez volteado como antes, con una deuda con el señor de los huevos y sin poder trabajar… No hemos podido trabajar bien… Se nos volvió la vida un complique a través de ellos… Lo que he estado agregando hacer es… no sé si de pronto… a través de alguna persona que se conociera para, para sacar la licencia…

I: Mhm

P: Para hacer lo que es legalmente, y conducir un uber…

I: Claro…

P: Porque es algo legal, que se puede hacer y… es algo que en todo país está pasando, que el avance de… de la comunicación y de la información… y el uber está funcionando en todas partes, aunque ponte en algunos países, fue acá, los taxistas se recienten mucho, en Colombia también, que por lo del uber… Pero eso tiene que seguir avanzando…

I: Sí…

P: Porque es… es como… el… el pasar del tiempo y de las cosas, que todo va avanzando y todo va cambiando… Entonces, estoy averiguando como hago para… para sacar licencia para ponerme a conducir un uber, para estabilizarme un poquito…

I: Claro…

P: Al menos tener seguridad y llevar el alimento a la casa, porque hemos estado… nada menos ayer, la fundación nos hizo un mercado, de bienes… de comida… Entonces es una serie de cosas que le van pasando a uno… al estar… si estuviera dentro de su país sufre de una forma, y si está fuera sufre de otras… Es un poco de consecuencias que hay…

I: Si… es fuerte, todo el camino… Como esforzándose y luchando… Y enfrentarse de nuevo como a problemas… Mmm… Y… Y su niñita también está yendo al colegio, ¿Al Amunategui?

P: Sí, todos, E y V, ambos están estudiando y haciendo la actividad

I: Y ahora me imagino que están con todo esto del dieciocho…

I: Está… Hoy día estaban con una presentación allá en el colegio, van a tener una presentación. Que mi esposa no pudo ir, con muchas ganas, por no tener un pasaje para ir en el metro allá a ver los hijos y volver…

I: Claro, difícil…

P: Es pesado, pero bueno… si…

I: Entonces, y… como… Y bueno… Y FASIC siempre les esta… le ha prestado la ayuda como…

P: Sí, ellos han estado ahí apoyándonos en todo sentido, de hecho, cuando se fue mi hijo para Colombia, acá para mi esposa fue un poquito duro pues volver a… la idea de nosotros, como te conté en Colombia, yo casi no pude estar hace cinco, seis años… Como muy…

(Suena teléfono)

I: Conteste no más

P: Sí, y después llegar aquí… Quién es

(min 35:17 contesta y habla por teléfono hasta min 38:45)

I: ¡Una buena noticia parece!

P: Si… deliciosa…

I: Si tiene que llamar y hacer… llame al tiro…

P: Sí… Pero así está bien

I: Porque de repente, no sé, viendo la hora… después… pero…

I: Sí… Está niña Z precisamente del puesto de acá de República, ellos tienen permiso ya de la chilena (ininteligible min 39:09) Entonces ella tiene… ella y la mamá tienen un puesto con permiso y todo eso, y al ladito de ellas era que nosotros nos hacíamos a vender la fruta… Porque vendíamos la fruta picada como…

I: Mhm…

P: Fruta, yogurt y… y sándwich y… Entonces allá ellos fueron los únicos que nos apoyaron, del resto los otros ante los carabineros para que nos fuéramos. Entonces ella ayuda a los viñedos a trabajar, cortando vi… eh… a los viñedos…

I: Sí, haciendo…

P: A las uvas… Entonces que le va muy bien y todo, entonces me estaba dando la información, porque yo le he dicho, yo estoy dispuesto, si tengo que viajar para alguna parte y quedarme ocho

días, un mes, quince días, no tengo ningún problema, lo importante es trabajar para hacer algo, para producir… Ahora lo otro es que me dice que no es de este sábado, sino de este sábado al otro…

(Se acoplan voces, hablan al mismo tiempo)

I: Sí… pasado el dieciocho, porque ahora la gente está… como en otra… como que se para Chile…

P: Con la mente de rumba…

I: De rumba y esas cosas, y ahorita también me imagino que los pasajes deben de estar altos…

P: Si…

I: Pero después bajan…

P: Sí, pero si es por trabajar, y me dice que es por la empresa y todo… entonces vienen buenas noticias. Yo casi siempre digo… de que... la vida… tiene sus contratiempos, digámoslo así… pero deseos contratiempos aprende uno también. Aprende a mirar la vida con esperanza, con fortaleza y de todo momento, después de que uno supere las cosas se hace cada día más fuerte y eso es lo que ayuda al ser humano a formarse como… como persona… A valorar más todo… A ir llegando a ese grado de madurez que se va llegando a través del paso del tiempo, de los años… Y es de todas esas vivencias que le va tocando a uno tener de la vida, de confrontar ¿eh?

I: De enfrentar… De pararse…

P: Sí

I: Y usted se ha parado varias veces…

P: Sí…

(Suena teléfono)

I: Conteste no más, jajaja conteste

P: Perdón por dentrar las llamadas, pero es que…

(Contesta y habla por teléfono de min 41:13 hasta min 45:00)

P: Buen y… antes le decía que, retomando de nuevo… Entonces siempre ha sido si…

I: …Sí, si, nos están (ininteligible) un mensajito, me acordé jajaja… Como que… siempre ha sido el desafío… de pararse y todo eso, como que va aprendiendo, eso me estaba contando….

P: Sí, y… y que no es fácil, porque… de todas formas uno sale con la idea de… de lograr con unos sueños… unos objetivos, que… en mi hogar mi esposa y yo los hemos tenido muy claros desde siempre y…Y lo que te contaba que… a raíz de esto, pues cuando llegamos aquí vimos la posibilidad de que íbamos a poder estar todos juntos… Compartir bien, bueno como hogar, como familia… Pero resulta pues, lo que te contaba ahora, la parte deportiva de mis hijos… no fue posible, entonces y a mi hijo tiene que volver a regresar allá a Colombia… que la ventaja es que, nosotros a los hijos como que nunca los involucramos, no los dejamos como involucrar mucho en los problemas que tuvimos mi esposa y yo… O que nos ocasionaron por tener esos negocios, los

hijos siempre tuvieron pues, sus quehaceres por aparte… su estudio, su fútbol y entonces casi nunca ni nosotros les contamos como para no contaminarle mucho el tiempo, la vida o ponerles una vida de amargados, entonces…

(suena el teléfono)

I: Conteste no más Don Carlos jejeje

P: (Contesta y habla por teléfono min 46:34-48:42)

I Buena amiga la M… jajaja

P: Sí… sí, está al tanto como… bregando a que las cosas funcionen, eh… Sí. Ella también ha sido de las personas que le apoyan a uno y que lo han aprendido a estimar porque, porque uno empieza a trabajar, a trabajar… Somos de los colombianos buenos ¿eh? Entonces la otra parte, de cuando ven que la gente es así y que tenemos familia, y que queremos avanzar, entonces hay muchas personas que entienden eso y… y quieren apoyar, quieren comunicar de que forma ayudan, de que forma lo hacen… Entonces, ha sido muy bonito…

I: … Y a parte de la M, que la conoció como en el pasar de los días y todo eso… ¿Ha conocido más gente acá, cómo lo ha hecho?

P: No, la verdad que no mucho… No mucho, porque pongamos… lo que es el, la fruta, nosotros nos levantamos a las cuatro de la mañana, yo iba a la Vega en el triciclo moto a comprar surtido, luego llegaba una hora después a la casa donde vivimos y empezábamos a preparar… A picar la fruta, a empacar, el yogurt, los cereales, a hacer los sándwich con la suegra y los hijos…

I: ¿Tiene también suegra?

P: Sí…

I: ¿Y también esta acá?

P: Sí, también… La suegra ha sido un apoyo, es como mi segunda mamá, porque en Colombia, mi esposa es única hija, al ser única hija cuando llevábamos un año casados eh… mi suegra se separó del esposo…

I: Ya...

P: Entonces me llamó y me dijo que… que ella pues, no tenía para donde irse con familia, pero que la familia que tenía era C que es mi esposa, que es su única hija. Y yo le digo, no, no hay problema, vengase con nosotros, porque yo con mi suegra siempre me he llevado muy bien… desde novios, todo. Y toda la vida ha vivido, después de un año casados, mi esposa y yo llevamos diecinueve años de casados, vamos pa veinte de casados…

I: Lo de su edad… Casi del hijo mayor…

P: Del hijo mayor, al año nació mi hijo. Y al año, mi suegra se fue a vivir con nosotros y parte de la dieta la atendió a mi esposa el… el primer nieto. Y a partir de ahí empezamos a vivir juntos, entonces como te digo, mi esposa y yo siempre hemos ido en emprendimiento, a ver que más se hace… Casi no hemos estado trabajándole a nadie, siempre queremos cumplir nuestros sueños,

nuestra meta, nos hemos salido como un poquito de lo convencional… De… de… de esas ideas orquestadas que maneja todo gobierno en todo país, no le interesa que la gente progrese, que avance… sino que todos hagan en la misma línea para tener una esclavitud perfecta que todavía se ve en los medios de comunicación, todo esto hace que la gente malinterprete, malviva las, las cosas de la vida… Y entonces así alcanzan ellos a tener sus objetivos, por eso uno a veces escucha las palabras de que el rico es más rico y el pobre más pobre, es porque… de pronto no nos hemos puesto a mirar que socialmente ellos tienen todo un plan y toda una organización montada, de mentes brillantes de personas, que manipulan todo el país a nivel de ideas, de información, en los que hacen que todo el medio gire en torno a las ideas o principios que ellos quieren para manipular y así…

I: (interrumpe) Claro, como que mantienen la hegemonía…

P: Exacto, así es, mantienen ellos la… la familia Rockefeller, a nivel mundial todo eso… es algo. Entonces todos los países copian exactamente lo mismo, son maquinarias políticas que eso… nunca se acaba, eso…

I Claro, usted me contaba que ha tenido muy…. Siempre ha estado muy enfocado en el trabajo, entonces como tiempo de conocer…

P: No… la verdad no mucho

I: No ha tenido, me imagino…

P: Entonces siempre, siempre he estado ahí… trabajando…

I: Mhm

P: Con mi esposa, entonces ya llegábamos al puesto, yo llegaba y… abría a las… estar allá a las siete o antecito de las siete, por ahí faltando 15, ya con la producción, así picaba un poco más de fruta, acababa de empacar, mientras iba atendiendo, ya llegaba a las diez, once de la mañana… ya llegaba mi esposa que se quedaba despachando, ayudando a despachar los hijos… Y hacer alguna cosa ahí en la habitación, adelantando…

I: Claro…

P: Ya llegaba y me recibía, y yo volvía y me iba a la vega allá a comprar lo que iba a hacer en la tarde, que te conté que teníamos otro carrito… Eh… que se vendía sopaipilla… empanadas y…. Y entonces eh… yo salía en la tarde de nuevo, compraba y ya descansaba un ratico y mi esposa se quedaba de once que me recibía o diez hasta las cuatro. Yo a las cuatro, cuatro y media llegaba al mismo sitio y… y me quedaba ya empezar a vender la otra parte de la sopaipilla y ya ella se iba con el carrito para la casa… a esperar a que los muchachos llegaran, a hacer acompañamiento, a ver como les fue, en que hay que ayudarles para estudiar… mi esposa siempre ha estado muy pendiente en la parte de acompañamiento… Y ella se quedaba, y yo me quedaba hasta las once o doce de la noche trabajando en la sopaipilla y ya regresaba, volvía y me acostaba y eso era una rutina así diaria.

I: No… no hay espacio… donde…

P: De viernes a sábado, que sábado si sacábamos el de sopaipilla a las dos de la tarde y lo guardaba a las seis, porque mermaba y por qué el cuerpo estaba muy exhausto jajaja

I: Jajaja ¡me imagino! A esa hora… es como un horario como de… no sé…

P: De locos…

I: … de veinte horas al día… fuerte….

P: Fuerte…

I: Fuertísimo

P: Porque siempre hemos querido tratar de que las cosas donde estemos funcionen… de tal manera, que la parte económica estemos tranquilos y nos proyectemos… Que eso era lo que hemos querido, pero… bueno. Así se empezó a interrumpir todo cuando vieron que empezaron a fluir las cosas… y total que hace un mes para acá se nos volteó otra vez, que quedamos como cuando llegamos… Sin nada, con muchas ganas, pero sin muchas posibilidades… Debido a la parte social… no se sociabilizaba uno casi con nadie, sino con la misma gente que le compraba o le vendía allí…

I: ¿No ha conocido más colombianos? ¿Nada?

P: No, como te digo, siempre como encerrados, encasillados en… en el trabajo, entonces no…

I: Claro…

P: No ha habido vida social

I: Y conocidos de allá, de acá…

P: No…

I: No sabe que estén…

P: Muy poco la verdad… donde nos hemos apoyado más es aquí en la fundación FASIC, que es donde… si hemos estado acá con la señora Y, ahora con el joven nuevo que hay, y estamos pues en ese proceso, funcionando, mirando a ver como se hace…

I: Oiga y cuando tuvo… el proceso de solicitar y formalizar… ¿Ahí lo aconsejó alguien? ¿Cómo llegó al DEM? Fue en el 2017, el gobierno pasado…

P: Sí…

I: ¿Cómo fue ese proceso?

P: La verdad que yo llegué… y entonces yo…. Yo pensé bueno, ya estoy acá… y ya vería aquí en Santiago como posibilidades de empezar… Y entonces yo dije, bueno voy a empezar también por mis papeles, y yo recuerdo que… Que varios días que me quedé aquí en el terminal, como viviendo entre comillas allí… Yo subía con mi maleta arrastrándola por la alameda hasta llegar a la plaza de armas allá, a hacer el papeleo y todo eso, hasta que fui cogiendo la información y… y ya solicité refugio…

I: Alti… Y ahí le dijeron altiro…

P: Sí…

I: Usted venía… claro con la idea de solicitar…

P: Sí, o sea, de solicitar para poyar y mi familia… Total de que cuando ellos vinieron, yo ya había empezado… a los dos meses que vinieron, yo ya había empezado la solicitud de refugio, ya sabían aquí en la fundación FASIC, porque ahí mismo también le dicen que le recomiendan la fundación para que lo asesoren… y todo eso…

I: Ah, de den… de… ¿de la plaza de armas?

P: Le informan, entonces…

I: No le pusieron como entrevistas previas… fue como un proceso amigable…

P: No, el primer… Ya cuando me atendieron allá, ya… que fue en una madrugada, me atendieron y… y ya me preguntaron porque iba a solicitar… yo expuse el caso y todo esto, y ya con ellos empecé a participar de… del centro. Cuando ya mi esposa vino a los dos meses, ella antes de salir de Colombia, nosotros los negocios en Colombia los teníamos formalmente legalizados, en cámara de comercio, o sea, registrados….

I: Sí, todo…

P: Todo legal…

I: (ininteligible)

P: Exacto, entonces… eh… Eso dijimos aquí que aparecía y todo eso. Allá mi esposa fue a poner el denuncio antes de venirse, ¿Por qué antes no? Porque corríamos el riesgo de que pasara algo….

I: Y esa es la prueba de…

P: Y la presión allá de que de pronto pasara algo así, entonces nunca se hizo… Nosotros éramos (ininteligible) Pero ya mi esposa sabiendo que se iba a venir fue… por decir algo, allá salió en la madrugada… Y ese… Pongamos hoy salió en la mañana a… allá se llama fiscalía

I: A la fiscalía…

P: como la PDI de aquí y todo eso, a… a denunciar. Pero entonces ya le dijeron si usted denuncia no puede salir del país. Porque tiene que tener un proceso y eso era lo que nosotros no queríamos, porque en ese proceso, en ese esperar es donde la delincuencia allá… eh… cobra vidas o hace daños, porque no sé que información o… o como manejan de que se dan cuenta, se dan cuenta de quién está informando y… le hacen algo a él o a la familia… Para que, porque dicen que si a los comerciantes los dejan, que todos empiezan a hacer lo mismo, se les salen las cosas de las manos, o sea, a los delincuentes… Entonces, no sé como la policía o… como tienen la comunicación entre ellos, tanta corrupción, tanto problema… Entonces mi esposa desistió, dijo no, entonces no pudo denunciar. Pero tampoco dijo que se iba a venir…

I: Mhm…

P: Porque si dice eso, sigue siendo un problema…

I: Claro…
P: Ella dijo que, si iba a denunciar, entonces usted debe estar en el país, no puede salir del país… Entonces mi esposa se quedó allá, y dijo que iba a esperar tros días a ver que hacía… Y se regresó a la casa, y estuvimos hablando telefónicamente y ya… pues, eso fue pongámosle hoy en la mañana… Y ya regresó a la casa, descansaron un rato y en la noche salieron, en bus, se vinieron por tierra viajando en bus… Se demoraron casi dieciséis días para llegar…

I: ¡Se demoraron aaaartos días! Me imagino, de Perú a Ecuador… Todo cruzando…

P: Todo cruzando…

I: Y después ni siquiera… Llegar a Arica y acá… es como dos Otros días….

P: Otros dos días… No, y llegaron de pronto a una parte como… llegaron a una habitación… Una serie de cosas…

I: Pero por suerte, por suerte… Si pos también… Y cuando… la pregunta que le iba a decir ¿Y cuando volvió a hacer el trámite para…? ¿No tuvo problemas?... ¿Cómo cuándo?

I: ¿Para…?

P: ¿Para el segundo carnet?

I: Sí, para el segundo carnet

P: No, de hecho, para este fin de mes deben de… debemos otra vez solicitar para mi esposa y mis hijos…

I: Claro, porque tienen una diferencia…

P: Exacto… Y… pues el mío ya me llegó como te conté, y ya hacemos el proceso…ellos creo que tampoco se va a problema… Hasta ahora ha sido todo como normal.

I: ¿Y con esa visa que le dan temporaria como le va para conseguir trabajo?
P: Mmm… Como estábamos encaminados en el emprendimiento y en todo esa parte…

I: No había tenido la experiencia

P: No… no hemos tenido, pero ahora si estamos viendo la necesidad o que va a tocar emplearnos…

I: Mhm…

P: Porque… para el emprendimiento es más… más dificultoso, en eso no hay… el gobierno no apoya, no… O sea, hay muchas cosas que de pronto aquí antes atacan al inmigrante. Con este nuevo gobierno ha sido más pesado todo, todo según yo más duro…

I: Mmm ¿Y cómo ha vivido eso? ¿Cómo cambió?

P: No, muy fuerte, muy fuerte el cambio con este señor. Eh… porque tengo entendido de que él es uno de los empresarios grandes de acá y entonces, claro, por lo general esas mafias políticas siempre tiran para el lado de ellos y no les interesa el qué, el que quede atrás o que tenga que

pasar por encima del que sea… no les interesa, es gente que manejan unas mentalidades muy pobres… Esa es la verdadera pobreza del ser humano, una mente pobre, la corrupción… Cobran mucho perjuicio, mucho perjuicio.

I: Claro, y el proceso de … no le incentivaron… o sea, no le llegó… el proceso de regularización no le… A nosotros nos ha llegado, nos han contado otras personas, sea, este hallazgo, que como con el proceso de regulación… regularización en a las personas que están solicitando refugio, les decían, como que los engatusaban, les decían “no, pero usted regularice y como… tome esta vía y no la otra”. ¿Le pasó eso? ¿Le llegó eso?

P: A mí no, la verdad que no

I: ¿No le pasó nada de eso?

P: No, por ese lado no. He escuchado, pero igual he escuchado a quién le haya pasado, pero no me ha pasado. De pronto ahorita, como hay mucha gente y… inmigrantes todos, como hay tanta presión de los carabineros votando, llevándoseles las cosas a los venteros ambulantes, y todo esto… Han habido momentos en que les dicen que… que hay bonos para que se devuelvan a sus países, todo eso… A los haitianos, a los colombianos….

I: (Interrumpe, ininteligible)

P: … Feriantes… que mucha gente como que está haciendo eso, la verdad que no sé hasta donde es verdad, pero he escuchado…

I: Mmm…

P: He escuchado esa parte…

I: Claro, como que eso está muy nuevo desde ahora como, un avión para los haitianos, para el que quiera… Cosas raras que están pasando ahora…

P: Pero es por eso mismo, porque ahora como que no quieren que… haya mucho inmigrante… Todos con mirada muy capitalista y…

I: Sí po…

P: Y muy desde… desde sus principios… entonces…

I: Es una corriente difícil…

P: Sí…

I: Y… y fue… Y a parte de esa experiencia, antes… como que me contó en el servicio de salud, ¿había tenido otra? Con lo de la chica que tenía el dolor.

P: No

I: ¿Nunca?

P: Antes… antes… antes… Mmm… Antes me parece que el jo… el joven eh… solicitó la otra vez y lo atendieron bien.

I: ¿Su hijo?

P: Mi hijo, sí. Pero ya después con la ayuda, pues como pasado un tiempo fue como que había caducado, pero el resto…

I: Mmm… Y… ¿Cómo se ente…? Me contaba antes que… entre que llamaba… (ininteligible) Son cosas que pueden pasar…

P: Sí

I: El tema de como que usted se volvía a parar y todo eso, de como que iba como… que cada vez que superaba algo como que se volvía más fuerte… En ese tema, yo le quería preguntar, si usted también había… ¿Cómo? Lo de… ¿es religioso?... ¿Cree en dios?

P: Sí, si claro… O sea…

I: En ese… Lo liga a ese proceso como de… reinserción… de resiliencia también con la religión, o lo ve más por separado, como… ¿Cómo es ese proceso de resiliencia que usted… que me cuenta?

P: Mmm… Yo toda la vida he tenido claro que hay un ser superior…

I: Mmm…

P: Eso no lo inventó, eso no existe pues por coincidencia… Tiene que haber una mente brillante, súper brillante y… superior, que nos da la posibilidad de hacer todas las cosas ¿eh? Mmm… Que no permite, sino que ya se rompe e que empieza a hacer mal uso d e los beneficios que ha recibido, y entonces eh, empiezan a formar esas clases sociales y esas diferencias donde empiezan a paullar o a dañar a muchas personas, que tienen unas ideas, unos principios grandes… Pero como no es de tal apellido, o no viene de tal familia, no se le presta atención, pero eso pasa exactamente en todos nuestros países latinos. Que… mmm… dañan, dañan, dañan los sueños, las ganas de muchas familias, de muchas personas, querer salir adelante, querer hacer cosas, nuevas, querer apoyar incluso a los mismos países con su forma de vivir, de actuar, de ser… Pero… el medio de consumo… las máquinas políticas no dejan que esto funcione, que esto surja… por lo que hablábamos ahora en algún momento, de qué … de que a ellos les conviene mantener un caos… Entre más…

I: Claro, como un desorden

P: Exacto, entre más desorden mantengan dentro de una comunidad, para ellos mejor, porque los poquitos que quieren hacer algo diferente o pensar o actuar de otra forma diferente, no lo van a hacer, van a estar siempre… mezclados entre todos y usted sabe que donde, donde… la mayoría es maldad, daño y todo eso… el mundo se va contaminando y entonces a eso es de pronto a lo que estamos llamados como personas, como seres humanos, a no dejarnos contaminar de esa forma, sino tratar de mantener uno su horizonte, su sueño, sus metas claras… Y… independiente de como esté o como le esté yendo o lo que esté pasando, esos sueños y esas metas no pueden abolir, nunca se pueden desligar de la persona, de lo que uno quiere… de lo que uno puede alcanzar, de lo que uno quiere para sus hijos, de lo que uno quiere para uno ¿eh? Y… entonces, esto… esto es un reto con uno mismo, de no dejarse robar esa posibilidad al menos de soñar, porque como le digo, he escuchado a un escritor que decía que cuando una persona sueña el sueño es el que mantiene vivo, el espíritu, el alma… lo que en sí la persona es, la esencia. Pero si se pierden los

sueños, la meta, las ganas, entonces pasamos a ser uno más del común, uno más… Y es lo que no se puede dejar que pase… Es tratar de hacer cosas diferentes, para lograr cosas diferentes. No se puede hacer cosas diferentes haciendo siempre lo mismo, así que hay que lanzarnos, botar los miedos. De hecho, yo como inmigrante, lo que primero boté es el miedo, porque si le diera a uno miedo entonces no sale, no busca, no crea otras opciones… no cree que hay posibilidades mejores… Pero como hay esa fe, esa fortaleza, esas ganas de… de hacer nuevas cosas, de que los hijos tengan nuevas posibilidades, de uno… vivir nuevas experiencias bonitas… dejar un legado, o sea, que en realidad uno no puede solamente estar aquí y ya… De pronto que la vida trascienda y cuando uno trasciende, o la vida de los que han trascendido… Que uno recuerda a través de la historia… de… de los historiadores nuestros, de personas que han sido capaces de… de trascender en la vida, digámoslo de la parte social…

I: Claro…

P: O de la parte educativa, o de la parte académica o de la parte política… O de la deportiva…

I: De muchas formas…

P: De todos los ámbitos, campos del ser humano, digamos personas que han sido berracas, soñadoras, han sido personas que se han lanzado… Que no les da miedo hacer las cosas…

I: Y usted ¿tuvo un modelo así… cercano… como de toda esta fuerza, de toda como esta energía? fuerza que…

P: No le entiendo…

I: SI ha tenido como un modelo… Alguien que le haya inspirado como en esta lucha que…

P: Sí…

I: O… de a donde saca como esto… Es mucha la fuerza, entonces por eso le pregunto…

P: Ya, te entiendo. A ver, lo que pasa es que, como te decía, a través de que va pasando el tiempo… uno se va dando cuenta de tantas cosas, pero obviamente que uno mantiene sus principios, esos valores y ese rumbo cuando uno lee, cuando escucha audios o se entrevista, o habla con personas como tú o como muchas personas que son valiosas en cada parte donde he estado, casi siempre forma uno… un vínculo, aunque sea pequeño con distintas personas, pero son con personas valiosas, personas que te aportan, no personas que te quitan , si no….

I: Y de eso…

P: … Personas que te dan ideas, que te estimulan a seguir a delante…

I: ¿Y allá también tenía en Colombia a esa gente?

P: Sí

I: ¿Y era su familia en general?

P: Mmm… No tanto mi familia, no tanto, yo casi siempre hemos vivido muy aparte de mi familia y mi esposa de la familia de ella, hasta hemos tratado de vivir más bien como entendiendo que, bueno si vamos a la biblia entendiéramos de que… cuando habla dios de la familia, dice que el

hombre cuando se casa pues ya pertenece a ese nuevo hogar y… y a raíz de ese hogar esa es su nueva familia y así. Obviamente sin abandonar o sin dejar de estar pendiente de los de uno, pero si prestándole más atención al hogar que ella formó, a su esposa y a sus hijos y como va a ser para que aquellos avancen y crezcan… Entonces cuando uno adquiere en la vida las responsabilidades, o cuando yo me casé, que ya tenía claro que iba a ser o para donde iba y que era algo que iba a ser complicado, pero que yo tenía que estar al frente como cabeza de hogar… entonces cuando uno asume todas esas responsabilidades eso lo empieza a hacer fuerte a uno. Esa fortaleza va llegando a través de usted querer hacer las cosas bien, cuando usted se pone metas y sueños, no solamente de a lo suyo, sino cuando uno ya se casa, y quiere usted ver unos hijos bien formados y que crezcan viendo un papá, una mamá… Que casi siempre el setenta por ciento he escuchado yo, o he leído en libros, que son los modelos para seguir de los hijos, entonces ve uno la importancia de tener un hogar bien establecido, y a raíz de yo y de unas buenas lecturas y… y enfocar siempre la vida en metas, en avanzar y en crecer… eso es lo que lo que hace a uno bien. Y entonces es ahí lo que uno alcanza de pronto la felicidad, que la felicidad no es tanto un carro… o un apartamento lujoso, una mansión… Son cosas que de pronto son venas y que uno gozar de ellas sería muy sabroso, pero sería mas sabroso gozar teniendo una educación verdadera…

I: Mmm…

P: Pienso yo que la educación verdadera del ser humano no nos la dan en los colegios, en las universidades, esas las tenemos que aprender y… Yo creo que la educación financiera, la educación emocional del ser humano, esas dos educaciones, nunca nos las han participado y cuando una persona se hace exitosa… Eh, hablo yo exitosa, pero no por la cantidad de plata que tenga, sino exitosa en el campo humano.

I: Claro

P: El que yo te puedo mirar a los ojos, respetarte, y… y entender que en usted hay una gran persona, que yo debo respetar y… y que tienes unos valores, una grandeza, y que puedo hacer de mí una persona también digna y avanzar, ósea es una cuestión de valores…

I: Claro

P: … Grandes… Esa es una verdadera riqueza. Ahora, si habláramos de la verdadera riqueza está en la mente. He escuchado de la riqueza, la riqueza mayor está en la mente, entonces de acuerdo a los principios o a lo que usted piense, esa es su riqueza o su pobreza, lo que pasa es que a nosotros los medios, la sociedad nos tiene todo revolcado, y todos los términos los tiene tergiversados totalmente…

I: Claro, como que… o sea, totalmente, pensar en el dinero y no pensar… Claro… Oye y…. y eso… Entonces, ¿usted es originario de Medellín también?

P: Sí, siempre de ciudad de Medellín

I: ¿Y su señora, todos de Medellín?

P: Todos, mi esposa, todos ahí de Medellín. De hecho, pues, de familia antioqueña que se llama, del departamento de Antioquia, mi papá es del suroeste, mi mamá también… Pues…

I: Un poco tirado pa la región ¿o no?

P: Sí, ya es para la región como del campo, algo así y… Igual los papás de mi esposa C también, también, ellos son… unos de ahí cerca, pero de un pueblo también Girardota y otros también son del lado del suroeste, así todos somos de ahí mismo de Medellín, oriundos de allí y todo. Sí…

I: Genial… No… Muchas gracias por la entrevista, si quisiera agregar… Bueno, como le conté, bueno, ahora no sabemos hasta cuando va a ser este tema, por la nueva por sintonía… Pero… Eh, muchas gracias por la entrevista… Y… tal vez le puedo pedir, bueno y dejó su teléfono, ¿el xxxxx?

P: Sí

I: ¿No empieza con +569?

P: Sí…

I: xxxxxx (repite número de teléfono) Ya, sí, porque yo voy a… Bueno, sociabilizar jaja como solamente con ella, con la investigadora responsable y como, como en esta ocasión, como en este formato, que yo estoy aprendiendo, porque normalmente yo siempre usaba la entrevista estructurada…

P: Sí…

I: Que eran como más preguntas y más direccionada, pero esta es como una entrevista biográfica, de hecho, ahí está como la entrevista semiestructurada, pero esa es como para informantes clave. Y esta es como la corita (min 01:16:43 explica fin de la entrevista y agradece entrevista)

Entrevista Carlos código 01

P: Ya… ajajaja. De hecho, hay una idea grande empresarial.

I: Mhm…

P: Mi esposa y yo tenemos un proyecto… que… lo vamos a realizar. ¿En qué momento? No sabemos, pero lo tenemos claro. Y… y es porque en qué momento, porque no… No hemos dado… no hemos dado como… como en el ambiente o como en el momento apropiado… Para empezarlo a realizar.

I: Mmm

P: Pero tenemos una idea empresarial grande, grande, grande, grande.

I: Mmm

P: Buena, muy buena…

I: ¿Y es como su meta?

P: Esa es la meta fin… final mayor digámoslo así, porque a través de esa meta final mayor… no solamente nos vamos a realizar nosotros como… como personas, mi esposa y yo, nuestro hogar, sino que van a haber muchas otras personas que se van a beneficiar… Ese e s el sentido grande del

porque vivir… porqué vivir para usted solo o para usted… En una forma egocéntrica, realizarse como persona y tener todo lo que usted quiere, realizar sus sueños… Pero si usted no pone eso al servicio de otro…

I: Mmm…

P: No es nada… No se hace nada, no se logra nada. Y… entonces, la vida se vuelve bonita y se vuelve exitosa…

I: Mhm…

P: … Cuando usted su vida la pone a girar en torno de…

I: Claro… como que agarra sentido…

P: El bien común ¿Eh? Cuando piensas en el otro… cuando, cuando lo que estas haciendo da posibilidad que otras personas estén…

I: Mejor

P: Bien… y que tengan una calidad de vida y que puedan adquirir una buena información, una buena educación…

I: Mhm…

P: Entonces ya eso te hace… te hace más libre, te hace una persona mejor.

I: Mhm…

P: ¿Eh?

I: Oye… Pero, principalmente, entonces como, entendiendo, pa entenderlo también como su experiencia y su… ¿La mayor fuerza es como el sueño? Más que la religión pareciera ser el sueño ¿no?

P: Sí, es real… La religión te esclaviza… Eh… hablémoslo así, o sea, si hablamos de la religión católica… de los musulmanes… Hablemos de cuantos miles de sectas que hay ahora ¿cierto?

I: Claro… de todo…

P: No es para… para decir que es malo…

I: Mhm…

P: No, pero… han habido manos humanas que entienden la biblia o quieren interpretar a su modo, entonces se van por otros lados…

I: Mhm…

P: Con eso no quiero decir que es malo ni es bueno, simplemente entender que hay un ser superior… Y es de pronto mirar en la parte humana ese ser superior, como le gustaría que usted viviera con las otras personas…

I: Claro

P: Entonces un dios humano, un dios bonito… Donde hay un dios de amor…

I: Mhm…

P: Pero no de esclavizar, no de dañarte… No de manipularte… no un dios de bolsillo y lo saco cuando yo quiero o como lo quiero… interpretar ¿cierto? Por que yo vengo de países donde… donde el que es malo va… y echa una plata a una alcancía de una virgen… o de un dios para que le ayude a ser malo…

I: Ah no, claro… Como que hay un poco como… como… si po, porque como el carril de ese dato, de este… es que es un poco distinto, como que la santería es mucho más fuerte ¿no?

P: Exacto, exacto…

I: ¿Como?...

P: Entonces, a eso me refiero yo, que acomodarse a un dios de bolsillo no… no es justo, o sea, si te pones a imaginar o… o sea, te pones a ver… cuando te levantas, que puedes despertar y ver un bello amanecer… ver el campo…

I: Mhm…

P: Ver a tu alrededor… sentir tu cuerpo que ya se despierta, que puedes hacer algo nuevo, que tienes una mente, que tienes cinco sentidos, o sea, te poner a mirar un poquito solamente alrededor de tu vida y…

I: Si po…

P: … Caes simplemente como en éxtasis y sientes y… Hay un ser superior, tiene que haberlo. Y nosotros tenemos razones grandes por las que vinimos aquí… Ahora vamos a hacer que esas ideas surjan y que se dé… O sea, no podemos dejar adormecer… cada uno de nosotros como personas, todos sus valores, todas las capacidades que tenemos…

I: Claro…

P: Porque eso si es… O sea, si nos pusiéramos a verlo en la parte religiosa… eso sí es pecado… no sacar ese genio y esas capacidades y esos valores tan grandes…

I: …Como… Como no realizar…

P: Exacto, y no realizarnos como personas… por dejarnos involucrar de tantas cosas que no tienen sentido… Entonces la vida es grande, es grande… y tiene muchas cosas bonitas. No podemos dejar perder la sensibilidad de lo pequeño… Que ahí es donde está la grandeza… en lo pequeño, en lo que uno a veces… ignora muchas veces, ahí hay más grandeza de la que uno se imagina… Entonces cuando, volvemos a retomar un poquito, cuando la vida de nosotros empiece a girar… en torno a y por el beneficio de… entonces nuestra vida cobra un verdadero sentido y nuestro padre eterno, ese ser superior, va a estar contento… de haber permitido venir personas como tú o como yo a esta vida. Por eso los objetivos y los sueños de los otros tenemos que realizarlos, tenemos que hacer que se realicen…

I: Muchísimas gracias jaja… Don X… Ahí… ¿No quiere llevarse eso?

P: Sí…

I: O… O esta, más fácil jajaja yo me llevo esta…

P: Ah, muchas gracias

VENEZUELA 5.10

Muchas gracias...
E : Entonces me contaba usted señora Sulay que llego a hace un año y medio aquí
S **:** Un año y tres meses tengo
E: Mmm… recién tres meses. ¿Y porqué motivos partió?
S: Por el acoso político, un amedrantamiento total, pertenezco a un partido político… de voluntad popular con Leopoldo Lopez y Lilian Tintores, que es contra el gobierno y bueno no se podía, me metieron presa tres días por repartir volantes en contra del gobierno, me golpearon me sacaron un diente, y bueno mal trato total. Me llevaron a declarar a un tribunal militar siendo civil, a los, a mi edad que ya soy sexagenaria prácticamente
E: Aha
S: tengo 68 años pero me siento de 15
E: Así la veo
S: Y bueno por el futuro de mis nietos, mis hijos, pero fue imposible y mis hijos me, me pudieron sacar del país y mandarme para acá para Chile, que agradezco muchísimo porque hace...3 o 4 meses me dio un infarto y me atendieron maravillosamente en el Hospital San José, una maravilla, desde el médico hasta la que limpia, fabuloso. Me dan mis pastillas, tengo mi tratamiento y gracias también a la la Fundación Fase me han ayudado mucho espiritual y materialmente, estoy muy agradecida de Chile
E: Eso fue lo que paso en los últimos añ**os**
S: Si
E: ¿Usted de que ciudad es? ¿o donde vivía?
S: ¿Perdón?
E: ¿Usted de que ciudad es?
S: Caracas
E: ¿Caracas?
S: Si, Caracas
E: Y toda esta vida activismo político de participación en el partido fue en Caracas
S: Si, si desde uh! muchos anos
E: ¿Y eso ha sido toda su vida o empezó a actuar cuando empezó el chavismo? ¿como fue?
S: No, no toda mi vida he estado participando políticamente desde afuera, pero ya cuando ganaron estos delincuentes, narcotraficantes de todo, me dediqué mas, mas adentro y entonces ya tuve mas contacto con Leopoldo Lopez, con todos esos políticos y ayudaba en cierta forma a hacerle entender a la gente que estaban equivocados. De hecho la prueba, a través de los de los años, ya van 18, 19 años de dictadura. Y ahora es que se están dando cuenta, y gracias al apoyo internacional, y ojala, y desde aquí estoy aprovechando estamos reuniéndonos para que desde aquí también poder ayudar, en lo que se pueda
E: VY usted antes de tener que partir así, había pensado en migrar? ¿Había pensado salir de Venezuela?

S: No, para nada, y me vine en contra de mi voluntad, fueron mis hijos que me obligaron porque ya era demasiado, me golpearon... y ya ellos tenían mucho miedo, pero si por mi fuera no me hubiese venido, prefiero morir de pie y no arrodillada ante esa gente (tono enfático) y sufro mucho porque mis hijos están allá, y ... y claro que me tratan bien pero, no es como
E: No es su familia
S: No es como (ruido de golpe en la mesa), como mi país
E: Claro
S: Perdoname
E: No, imagínese, no, no...
S: Perdoname (sollozos)
E: Es muy difícil la verdad
S: Salud
E: Salud, por Venezuela... yo viví en República Dominicana
S: Si
E: Y ahí estábamos recibiendo muchos venezolanos, personas como usted que nunca habían pensado en salir
S: no, no, para nada
E: Que eran felices en su
S: Oh! inmensamente
E: En su tierra y
S: Inmensamente y...mas o menos éramos de la clase media (golpea la mesa), media baja pero, teníamos todo, carro, negocito, casa propia, y por herencia pues mis hijos, mi mama, mis ancestros me han dejado, eso se respetaba ahora no. Cuba, peor que Cuba, que es lo peor, eso es lo mas fuerte que es peor que Cuba
E: Y tan rápido, porque hace 20 años
(hablan al mismo tiempo)
S: Yo creo que no hay ladrones, no hay asesinos no hay sádicos no hay delincuencia así como la de Venezuela, es increíble. Increíble como dio un vuelco mi país
E: claro porque nosotros siempre conversábamos con ellos que Venezuela en los años 80
S: Ohhhhh
E: Que para nosotros fueron años muy difíciles, era un destino donde todos querían llegar
S: ¡Todos querían llegar a Venezuela! !Como no! Fue un cambio radical, total, gracias a Dios yo nunca vi ese hombre como...no tengo ese arrepentimiento porque me engaño, me, me impresiono
E: Claro ¿eso también aparecía en la gente de Venezuela no? que hay un periodo en que hay un encantamiento respecto a este proyecto
S: Si, si, si , el hombre decía cada cosa, pero yo estaba ya con Irene sabes que, que era su
E: Era su regalo políticamente
S: Si, si políticamente. Irene sabes que fue Miss Mundo, Miss Universo... y la gente decía que no, no, es bonita no podía ser, presi...que la gente (voz baja), ¿como estuviera

Venezuela si esa mujer hubiera ganado? apoyo internacional, era una mujer bonita, inteligente, con estudios políticos ¡pff! Pero la gente, también hay mucho bruto

E: Mmm

S: Hay el hombre buen mozo! por favor...y el tipo hablaba, como le gustaba a la gente que hablar man yo nunca estuve de acuerdo y yo siempre he dicho: un presidente tiene que ser millonaria rico para que no robe, que tenga ¡pff! (golpe de mesa), este no es un pobre diablo estúpido militar, que engaño a todo el mundo, engaño a todo el mundo. Mucha gente que hoy en día esta en contra, me deslumbro, como hablaban mentiras. Yo decía que actuara así seria maravilloso, ¡es mentira! Pero a todo nivel, a todo, a todo nivel; un país tan rico, que por eso tengo la esperanza y la fe, que esa gente caiga que va a ser pronto porque ya están, están acorralados. Sin embargo mi familia sufre vieras tu. A veces tienen, el dinero que tengan no pueden comprar comida porque no hay, no hay medicinas, no hay nada. tengo amigos con SIDA que se están muriendo, muriendo, viejitos, con la tensión como...niños, increíble en cajas. Un país que era tan (golpea la mesa)... Venezuela era como es Chile ahorita en sus peores tiempos, porque tenemos petroleo, hierro, oro, de todo! (golpea la mesa)

E: De hecho tiene mucho mas que Chile

S: Y tengo la esperanza cuando esa gente se vaya, Venezuela, claro suena inmediato pero si, porque todavía hay oro, petroleo, oro, ¡pff! ¡petroleo! que se podría

E: Y al principio cuando empezó usted estaba en oposición, ¿verdad?

S: Mmm

E: O sea siempre estuvo en oposición

S: Si

E: ¿Emm, al principio era mas posible, o sea? ¿Como se transformo en una amenaza el estar ahí? ¿O desde el principio fueron amedrentamientos? Como usted se fue dando cuenta del nivel...

S: Si, si al poco tiempo, pero ya desde que, a partir de, de los primeros años que el hombre, el hombre empezó a ser agresivo, cuando salia de su carro (golpe de manos), hacia así durisimo. Eso significa: violencia. Y la su, la gente, una mujer que se murió que ella quiso reclamar y la mataron, que se llamaba Nena Romo, que se encargo de meterse en los barrios y armo a la gente, les dio muchas armas, a mucha gente porque yo vivía en Catia

E: Mmm ¿Catia es un barrio?

S: Catia es una parroquia de clase media, una de las, 23 de Enero, cerca y ahí era donde nosotros mas trabajamos, por eso, por esa gente débil, tan ignorante. Y ellos también y armaron a un poco de gente que empezaron (sonido que hace como de tiros de un arma de fuego) los muertos los atracos, como el ajedrez, esos eran los peones, a los mendigos

E: Los armaban

S: Los armaban

E: ¿Pero era el mismo gobierno que armaba a la gente?

S: El mismo gobierno, esos fueron los primeros años ya, que la gente empezó a darse cuenta, bueno, marchas, las marchas eran ya como a los 5 o 6 años para que él no volviera, fue mas presión y cada vez mas y cada vez mas (golpea suave la mesa varias veces), ya se metieron los cubanos, hicieron muchos módulos en los barrios, con médicos

cubanos, comprando a la gente con tonterías, dinero, bolsitas de comida. Construyeron edificios de verdad, pero en las zonas de mayor fluidez, qué se yo, en la clase media, media alta, construyeron edificios y metieron malandros ahí, gente de lo ultimo, ¡de los cerros! Y así fueron equipando todo, toda Venezuela, entonces ya la clase alta, secuestros (golpea la mesa 3 veces), claro esta gente bruta e ignorante, me voy al Marqués por ejemplo, que era una de las zonas mejores, qué se yo, Chacao, poblador puro, pura gente marginal, marginal, extremadamente que yo no sé. Yo decía Dios mio ¿de donde salio tanta gente fea y chusma? ¿marginal? Yo no sabia que había tanta gente así, en mis tiempos en los 70s 80s había gente pobre, pero no gente marginal, ¡feisima! Desdientados (no estoy segura de esta palabra 13:25), feos, sucios, no entiendo de donde salio tanta gente así, no entiendo. A veces pienso que ni Venezolanos serán, porque he hablado con mucha gente, bueno mucho cubanos, ¿cubanos hay? ¡pfff! Esto se mino de cubanos (revisar esa frase 13:46). Y quisimos (trabajar? 13:54) pero no pudimos, no se puede, mucho dinero, compran conciencias. A mi hermana le ofrecieron que ella trabajaba en un ministerio militar, le ofrecieron una cantidad de dinero para, y ella dijo que no, mas vale que no. La botaron, la...

E: Ella trabaja para el gobierno entonces

S: Si, trabajaba en ministerio

E: Ministerio

S; La botaron y...ni esas cajas que ellos regalan, de todos modos nosotros nunca aceptamos eso, pero hubo un momento en que hubo que aceptar,¡¿qué vamos a comer!? Por una caja de esas de comida (golpea la mesa) que México le regala, y ellos nos las venden, ¡son unos desgraciados! son como el diablo, no sé, son satánicos, es horrible. Bueno yo creo que ni diafra (no segura de esa palabra, revisar 15:01), ni diafra. Venezuela es horrible ahorita, mis hijos me dicen bueno como pueden..que tengo un hijo que, que trabaja, él es director de informática en la alcaldía de Chacao, la única alcaldía que queda contra el gobierno, no sé como todavía subsisten, pero subsisten, y mas o menos bueno se rebuscan vendiendo cosas. Una pasta de dientes que vale millones, ¿como es posible? Mis nieta, que se acaba de graduar ahorita, no le quieren apostillar el titulo para poder, yo quiero que se venga , que se salve ella por lo menos. Todos los días salen mas de 5.000 personas de Venezuela, todos los días, para todos lados del mundo. Es increíble, es injusto, a veces le pido perdón a Dios y también reniego: ¡Oye donde esta Dios! ¡¿Qué hemos hecho?! ¡¿Qué hicimos?! No hay, no hay fuerza y son tan, tan pero tan... que hasta pertenece a, hacen ritos. Hubo un tiempo que, la santería se hace con animales que sé yo. Pues tengo un amigo que él quedo como loc prácticamente. El era chófer de Chavez y vio ,él lo vio como sacrificaron un niño para darle la sangre a ese hombre. ¡Un niño muerto! un bebé, recién nacidos, porque la sangre que, yo no sé que. En ese tiempo hubieron varios eh lateros, mendigos que amanecían muertos, sacrificio para ese tipo (golpea la mesa). Miraflores, La Casa Blanca y que esta, la gente que esta, siempre la gente, dice las cosas que, eso que parece un templo de brujos, tiene cocodrilos, cabeza, cocodrilos embalsamados, cosas rituales ¡pff! rituales, Miraflores es eso

E: Se transformo como en un templo, una cosa

S: Si, si así de brujos, de brujería, satánica! Y entonces ¿donde esta Dios? vale. Que el bien tiene que poder mas que el mal pero hasta cuando (golpea la mesa suave). Queremos ser como Cuba, ya Cuba lleva 60 años, ya llevamos 20! Y no pasa nada, porque el pueblo queda, ya todo el mundo; me perdonas ¡pero los mas rechos! (revisar esa palabra 18:19) se han ido, han tenido que salir, y me incluyo ahí, que soy mujer y vieja pero con la fuerza para lo que sea. Que yo le dije a Leopoldo y a Lilian Tintores: pero adiestrame yo quiero morir así! como hacen los chinos, me adiestran, ¡me estrello contra ello! Llénenme de bombas y yo me voy pasada, a explotar y morirme con ellos. Mucha gente como yo esta dispuesta a todo, pero ¿porqué no lo hacen? Entonces la oposición, parte de la oposición ahorita es, hay convenios parece, que hay dinero, así que
E: Pero no han podido tampoco sacar un liderazgo fuerte, ¿no? porque
S: Porque hay gente que se ha vendido! dicen que Julio Borges que era uno de los, se ha vendido. No por favor, yo no entiendo como la gente pueda venderse. No creo! te lo juro que no lo creo. Pero hay algo que...porque Trump que el que pueda, presidente de los Estados Unidos que pudiera, como dicen, que van a intervenir a Venezuela militarmente, ahora no, porque no le interesa Venezuela, le interesa ehh..en ese caso el petroleo
E: Mmm, bueno algo tiene que haber ahí (silencio)
S: No vaya a ser como lo que paso con Irán, que se metió Estados Unidos, ahora el culpable es Estados Unidos porque era la mitad y mitad y ahora hay una guerra civil
E: Entre ellos, claro
S: Y por eso ahora no quieren intervenir en Venezuela, de verdad hay mucha gente, de rojo cuando sale una marcha de ellos, ¡ahhh! ¿de donde sacan tanta gente? obligados ¿no? El ministerio que los obliga, que me consta. Son muchos los obligados porque tienen miedo a perder el trabajo que sé yo. Pero..que no entiendo, no entiendo...ahora parece que Trump ta' diciendo que si. Si van a intervenir militarmente porque nada hacemos nosotros, "el pueblo que dice no, que el pueblo no sale", si hemos salido! y bombas, ¿y cuantos muertos no hay?
E: Mm, muchísimos
S: Pitos, banderas contra plomos porque no les importa, no les importa si a la gente le da miedo, ahora ya no se puede salir del país, eso se acabo. Ayer me pasaron la noticias: prohibida la salida de venezolanos de, del país. Yo dije: ¿como? (silencio). Y aquí bueno, llegué casi una, hermana de mis hijos
E: Aha
S: Su esposo y su niña, chévere, los primeros días, chévere. El esposo ya, vino otra familia y ya yo como que molestaba, o sea
E: Es difícil
S: Lamentablemente, tengo una amiga que también deserto, es militar, deserto, me la conseguí aquí en Chile, también le dieron asilo político, esta trabajando. Tiene, ella tiene como 7 títulos, sabe 3 idiomas, lo habla y lo escribe perfecto, esta de cachifa, cachifa no aquí le dicen "nana"
E: Nana
S: Nana (golpea la mesa despacio dos veces)
E: Con 7 títulos universitarios

S: Y bueno esa familia gracias a Dios la tienen, porque es una tipa inteligente, educada, hace todo bien, cuida un niño, limpia la casa (aplauso enfático) y gana bien, tiene alquilada una casa que, en vista de mi situación, somos amigas de toda la vida y nos conseguimos aquí bueno, y supo de mi y vio que estaba mal donde estaba y estoy viviendo ahora allá, con ella

E: Esta viviendo con ella

S: Divino! sola. Ella, esa casa esta alquilada con un peruano que se caso con una venezolana, ella y yo. Felicisimo, chévere y estoy tranquila en ese aspecto, estoy buscando trabajo en lo que sea, yo quiero trabajar pero mi edad, por el infarto

E: Claro

S: Mm, y aquí me han ayudado mucho, y en esa situación (golpea la mesa con los dedos varias veces) me dio el infarto, que, ya no podía estar muy bien ahí donde estaba y me voy para esta casa (golpea de nuevo) y aquí me han dado (golpe)... me dieron un mercado como de 50.000 ah! que digo: que Dios no me abandonado. Me salvan la vida me da un infarto que pff!! yo! y eso quiero que le dije a... ¿como se llama psicóloga?

E: Inés

S: Inés, ¿pero qué quiere? Yo lo que quiero es: donar mi cadáver. Yo no me quiero morir que, que se vayan a hacer responsables por ejemplo esta, hermana mis hijos, ni nadie, mi amiga tampoco, nadie, o sea eso seria, yo pienso en eso. O mis hijos que se vayan a querer no, buscar barrial (revisar 24:30) donde no tienen, ¡no!

E: Mm

S: Quiero donar mi cadáver, algún órgano que sirva, pero como tengo, como estoy tan vieja ya! pero mi cuerpo voy a servir que los estudiantes estudien cualquier cosa pues (diga lo ves o no? revisar esa frase 24:46)

E: Mm

S: Le dije a ella, me dijo: no yo creo que eso es (golpea la mesa varias veces) donde uno, como ya tengo que, ya tengo...tiene que darme otro rut, creo que es allá que se hace esa gestión

E: En el registro civil quizás ¿no?

S: Si en extranj... en...aquí en, en inmigración

E: Eso que esta

S: Donde me dieron el asilo político

E: ¿Usted ya tiene el asilo político?

S: Si!

E: Se lo reconocieron

S: Si! si, si

E: Eso fuer rápido entonces

S: Fue rapidísimo, que yo tengo pruebas, videos, tengo videos cuando me golpearon aquí, que guardia me dio con el, con el escudo. Me sacaron un diente, que, yo cuidaba a un niñito y me pagaban, me pagaban 10.000 semanal

E: ¿Cuanto es eso en...?

S: Y todo eso lo reuní y me puse un puente, porque, tanto renegado uno dice, cuando uno escupe la cae la saliva en la cara, vieja fea y dientada, ¡imaginate! Y reuní, reuní y me puse mi prótesis, me puse un puente que, me costo ciento y pico pero (golpea veces

entre medio la mesa despacio) ¿que no hacia?, planchar, yo planchaba en mi casa y yo pagaba para que me plancharan, y me puse a planchar, mira planché (voz mas baja y medio quebrada) ...y reuní algo, por lo menos me puse mi diente. Y aquí bueno me dijeron ese mercadito, oye pero que fino!, que bueno! ¿Sabes lo que es 50.000? Fui con Sebastián

E: Si Sebastián

S: Fuimos al mercado, y tenia la lista de los...fabuloso; Y en el hospital me dan las medicinas mensual, todas! el tratamiento que tengo pues, tengo dos catatirismos (sic) y hay una arteria que no se me ha destapado. Y con el tratamiento van a ver si no había, no me tienen que intervenir otra vez

E: Claro

S: Pero me duele mucho aquí, tengo dos días botando sangre por la nariz, o sea me siento

E: Todo le duele

S: Me duelen las piernas. Tengo, estoy haciendo terapia cardiovascular dos veces a la semana

E: Ya

S: En el Hospital San José

E: Y tiene que ir allá

S: Y aquí los lunes yoga

E: A que bien

S: Maravilloso, maravilloso, me sentí tan divino el lunes que fue mi primera clase por Inés, aquí este salón por cierto. Divino, maravilloso, maravilloso. Pero yo quiero donar mi cadáver rapidito no mas, si un día ahí me muera

E: No quiere ser un problema para los demás

S: Si, si eso es dejarle un problema a otro, no quiero. Eso es lo que quiero solucionar lo mas pronto posible. Y que tengo que estar saliendo pa' allá y pa' acá y no tengo ni medio, que esta muchacha amiga mía me da. Ahorita cargamos la bip, 5.000 y ya. Ese es el problema

E: Si claro

S: Pero para adelante yo soy fuerte (golpea la mesa)

E: Y usted que hacia en Venezuela ¿en que trabajaba? ¿Como fue su vida antes de esto?

S: Mira yo de joven fui la primera promoción en los bomberos, el cuerpo femenino, voluntaria. La primera promoción de bomberos voluntaria del cuerpo femenino fue la mía y duré un tiempo, pero después bueno, conseguí trabajo en el seguro social. Trabajé muchos años, que estoy pensionada

E: Usted tiene pensión de ahí

S: pero allá, se lo dejo a mis nietos a mis hijos: ¡cobren eso hasta que puedan!

E: Claro hasta que exista

S: Y este, he trabajado así y después en la política que me pagaban, no un sueldo completo pero, si para moverme

E: Claro

S: Me gusta leer mucho, me gusta... soy bachiller nada mas porque no alcancé a estudiar antropología por cierto
E: Ah si por eso le pregunté por, me dijo mis estudios
S: No, no lo...duré un mes. Después me casé, salí. Después me dije: no tengo que terminar, tantas mujeres que hay casadas con hijos y
E: Estudian
S: Después me metí, me inscribí en derecho, también un mes, no pude
E: Con niños
S: Varias, tampoco, trabajo social también me inscribí, me dijeron que aquí podía estudiar. Y a mi ni me da pena con esta edad que tengo, ojala. Ya averigüé donde podemos inscribirnos, mi amiga y yo nos vamos a inscribir. Que ella es mas o menos como yo, pero es mas joven tiene 60 años, pero muy activa una tipa ¡ppf! Pero sus títulos ya, eso no le vale aquí nada
E: Porque no pudo sacar ninguna apostilla ni nada
S: Nada, nada. Tiene el de economía y administración, si los tiene pero no los tiene apostillados. Pero ella me dice no me importa, nos metemos estudiamos algo ahí, sacamos parece que tres meses una básica y podemos entrar a...total. Total para no estar haciendo nada
E: Claro ¿y cuantos hijos tiene?
S: Tengo dos hombres
E: Los dos hombres ¿y los tuvo joven?
S: Si muy joven. Me casé a los 16 años, lo tuve a los 17 y ahí ellos tienen sus hijos
E: Tiene nietas grandes entonces
S: Mis nietas, soy bisabuela
E: ay ay ay
S: Porque un nieto tiene 3 y una nieta se caso
E: Mmm, bisabuela de varios, de 3
S: Bisnietos imaginate. Pero yo a veces me siento de, de... de 15 y otras veces me siento de 100
E: Claro
S: Pero estos días he tenido mucho animo porque Inés me ha dado mucho animo. El yoga me encanto
E: Que bueno
S: Me ha fascinado. El otro lunes...y ahora que estoy viviendo con esta, con esta amiga que me siento mas cómoda, segura estamos investigando también por la edad no me dan trabajo mira (silencio)
E: Creo que en el metro ¿tiene usted la tarjeta de adulto mayor? Porque en el metro usted puede pagar menos
S: Si
E: ¿Tiene esa tarjeta?
S: No
E: Claro, mayores de 60 años
S: Si
E: Si, voy a averiguar

S: Ah voy a averiguar!
E: Déjeme anotárselo y así se lo, se lo mando o la llamo al teléfono porque ahora que usted me dice que la bip dura poco
S: Si!
E: Sulay...Porque si usted ya tiene rut
S: Si
E: Claro que ya tiene
S: No y lo voy a renovar. Yo fui, y tengo que ir el 15 de este mes a renovarlo
E: Ok
S: Y ahí es donde puedo decir que voy a donar mi cadáver
E: A perfecto si, claro que si, y ahí
S: Ahí es donde me dieron mi asilo político (golpea la mesa varias veces)
E: Claro ¿Como fue ese proceso? ¿Usted conocía la figura del refugio del asilo antes de venir?
S: No me lo dijo la hermana de mis hijos, a donde llegué
E: Ahhh, ella fue la que sabia de refugiados
S: Si, fui a inmigración
E: Ya, ella
S: Fui con ella
E: Ok
S: Y me dieron, no, eso es en el tercer piso en refugio, refugiados. Entonces expliqué yo tenia mis pruebas, mis películas , mis...no si me traje de allá la orden la fiscalía militar que me dieron
E: Cuando la llevaron presa
S: Si, y... de ahí me mandaron para acá, la iglesia yo no sé qué. Y vine bueno a la municipalidad también
E: ¿En que municipio estaba?
S: En Independencia
E: En Independencia. Su, la hermana de sus hijos vive en Independencia
S: Si, y ahí fui y fue una trabajadora social allá, donde yo estaba. Y me ofrecieron que silla de ruedas, ¡bastones y yo no! No quiero eso, o sea, no necesito gracias a Dios hasta ahora. Yo quiero donar mi cadáver, una notaria algo, ¿qué hago? Y ahí en la municipalidad no...me mandaron acá (golpea suave la mesa varias veces). Allá lo que me podían decir era para conseguir trabajo ahí, y me llamaron pero en eso me dio el infarto
E: ¿Como fue que le dio el infarto? Un día caminando, que...
S: No, estaba con la niña que la iba a llevar para, porque yo la bajaba
E: ¿Una niña que cuidaba?
S: Claro, la hija, la hermana de mis hijos (golpea la mesa suave varias veces)
E: Ah! Perfecto
S: Para ganar dinero, estar ahí por lo menos
E: Usted la cuidaba entonces
S: Claro
E: ¿Una niña chiquita?
S: Si tiene 4 años, me dice abuela y todo

E: Claro es como si fuera
S: Preciosa, si, si si. Y me llamaron para un trabajo en esos momentos en que me dio el infarto y eso es los desastres que hice antes: comía salsas, fumaba, ya no fumo. Desde que me dio el infarto ya no fumo, y comía muchas eh salsas, cosas que no debía comer y tenia tapadas las venas, las arterias
E: Claro, las arterias
S: Y me dio eso
E: ¿En la calle?
S: No, con la niña en el apartamento
E: ay ay ay ¿Y la encontraron en el momento? Usted se sintió
S: Me dio eso y esa niña es tan inteligente que le aviso a su mama, porque la mama y el papa siempre me llamaban: ¿como esta la niña? ¿como están que?
E: Entonces usted tenia
S: Y la niña, no sé como hizo, le dijo: mi abuelita esta mal ¿puedes venir a buscarla? Y él llamo y ella (sonido con la boca) atendió y por la video llamada, estaba en el mueble, Samanta, porque me daba miedo que fuera a abrir la puerta
E: Claro
S: Nunca perdí el conocimiento
E: Nunca perdió el conocimiento, se dio cuenta que estaba llamando la niña
S: Claro! Inmediatamente ellos llegaron y me llevaron al San José, porque ellos son profesionales y están trabajando gracias a Dios. Y ellos, personas excelentes, pero el esposo (pega a la mesa) le vino un familiar, 2 entonces ya no cabíamos, ciertas cosas que
E: ¿Era un departamento de esos pequeñitos?
S: ¿Mmm?
E: ¿Era un departamento de esos pequeñitos que hay?
S: Si, si. Frente a la municipalidad de Independencia, en una residencia que están ahí
E: Si, si
S: Residencia Las Flores
E: Ok
S: Bien bonito, chévere. Pero claro yo me retiré sin pleitos ni nada gracias a Dios (aplauso enfático), no estamos claros que
E: ¿Y sigue viéndolos?
S: Si como no
E: Claro, que bueno
S: Claro cuando uno llega a esos extremos así
E: Claro, que se llega porque a veces es difícil
S: Si señor
E: Poco espacio ¿Convivencia la familia, verdad?
S: Cuando, nosotros nunca fuimos emigrantes, ¡jamas! No, mira tengo amigos colombianos, dominicanos, chilenos. Conocí a un profesor, profesor no, bueno si él era profesor pero, él trabajo toda la vida en la biblioteca nacional, y yo trabajé en la biblioteca nacional, chileno. Y él era el jefe de nosotros
E: Mira

S: Excelente persona, no se que seria, no se de él, he pensado en buscarlo pero él esta en Venezuela
E: Quién sabe ahora
S: Quién sabe
E: ¿Como se llama él?
S: El se llama Rogelio Martinez...Bock, una cosa así
E: Mmm, no me suena como si fuera una persona, publica
S: No esta muy viejo porque yo ya estaba joven y él estaba viejo, pienso si ahora estará
E: Si ahora estará, claro. Si con todo esto que ha pasado en Venezuela
S: Si! seguro que ha salido, claro. Estamos con los (palabra y copellanos? 39:08) que era democracia, que eramos felices y no lo sabíamos, pero siempre reclamando. Es como aquí, yo veo aquí, ¡tanta gente de izquierda! que tengan cuidado porque así empezó Venezuela, por favor. Aquí hay mucha gente de izquierda, entiendo. Acaban de salir, de una dictadura prácticamente. Y todavía hay gente. Yo me quedé asombrada con las fiestas patrias. Se forma una plomamentazon revisar palabra) 39:46 porque estaban protestando con unos carabineros, ¡por favor!
E: ¿Donde vive usted ahora con su amiga?
S: Ahora vivo en... esto se llama Huachilaba (sic), ehh...
E: Para orientarme
S: Se llama... aquí, yo lo anoté lo puse aquí para
E: ¿Esta es su dirección?
S: Bien bonita la...
E: ¿Recoleta sera? Huechuraba
S: ¡Huechuraba!
E: ¡Oh! por eso, si, si A pues si, le queda lejos
S: Mas o menos. Bueno yo agarro la B02 y me deja al frente
E: ¡Ah! perfecto, Banco del Estado, B05. ¿Y esa B02 la trae hasta acá al centro?
S: No, si. Me trae hasta Independencia y al metro Héroes
E: Bueno, Caracas es tan grande como Santiago ¿no? Ya estaba acostumbrada a vivir en ciudades tan grandes
S: Si, si, si, hacia...
E: ¿Y sus hijos como están allá? ¿Como puede hablar con ellos?
S: Subsistiendo, subsistiendo
E: ¿Qué hacen?
S: Uno es director de, de informática en el mercado de Chacao que es independiente, es un mercado muy grande, y el trabaja pa' la alcaldía de Chacao que es la única posición que queda en el país
E: Claro, claro que usted me estaba contando. Y entonces el vive en Chacao, no en, no en...
S: No, vive en Catia. Bueno él me dice que ese mercado lo que van a comprar es puros millonarios, él no puede comprar un kilo de arroz ahí. Que no, o sea no le alcanza, él hace otras cosas. Y mi otro hijo vive en Margarita, es gerente de espectáculos ahí en Margarita en un motel, el mejor hotel
E: Aha y que sigue funcionando

S: ¿Ah?
E: Siguen funcionando
S: Si porque los, los...ladrones estos que ahora son burgueses... y él ve cada cosa ahí, como malgastan el dinero, tanta gente muriéndose de hambre, y un recién vestido de su...un hombre que hasta preso estuvo, que es un...es como un juez, un juez...muy famoso muy, un hombre que estuvo preso, un delincuente, llega con sus hijos, su familia pero a derrochar dinero en ese hotel
E: Dios mio
S: Y él tiene que...yo no sé
E: Claro
S: No puede solo contra el mundo, yo le dije: ¡no! tranquilo, ¡no! Dependiente, no te metas eso, mira lo que me paso a mi, porque yo, uno cree que, yo creo que soy superman...pero bueno, por lo menos subsisten, que le...
E: ¿Y usted los crio sola?
S: Sola
E: Porque el papa
S: Nos divorciamos ya ellos tenían como 15 años, y nos divorciamos, precisamente por esa hermana (golpea la mesa varias veces)
E: Ah o sea, ahí esta la hermana
S: Que ya
E: Que formo otra familia
S: Si
E: ¿Y ella vino acá también por la crisis?
S: ¡Uh! Hace
E: ¿O esta hace mas tiempo?
S: Si ella ya tiene como 4 años
E: ¡Ah! ok pero igual en el contexto de...
S: Si, si
E: ¿Y ella salio ya con su marido? ¿O salio sola también?
S: No, con su esposo si
E: ¿Y su esposo era chileno?
S: Mm, mm (de negación)
E: ¡Ahh! venezolano
S: Venezolano
E: Había entendido que se había casado con un chileno
S: Se caso allá, se vino . Todavía no estaba ese
E: Aha
S: Que la gente se escapara de allá
E: Claro
S: Quisiera escaparse pues. Se sufría pero todavía no habían, hasta que empezaron a, a meterse con la gente civil
E: ¿Entonces ella escapo no como refugiada?
S: No, no. Son profesionales si apostillaron sus títulos y están trabajando
E: Pudieron hacerlo con mas tiempo

S: Si, si el trabaja en una editorial muy famosa aquí, porque son unos argentinos. Y él es un economista muy ilustre, el tipo sabe

E: O sea le ha ido bien acá a ellos

S: Si, bien en el sentido que, ni tan bien porque, no gana lo que él debería ganar porque es Venezolano, o sea, aquí a los extranjeros los...no le dan lo que se merece, era porque tienen poco tiempo también, que yo, él, cuando llego aquí trabajo en una bomba de gasolina y ella también en una caja, cajera de una bomba de gasolina. Y él echando gasolina. Porque, mientras hacia los

E: Papeles

S: Los tramites. Le iban a pasar un trabajo, y ahora que se esta viniendo su familia también

E: Ah entonces esa otra familia que llego, también es de Venezuela

S: Si. Entonces yo como yo quiero traerme a mis hijos y mis nietos, ojala pudiera pero...mi hijo mayor me dijo: no, estamos tratando de apostillar el titulo de su hija, mi nieta

E: ¿Qué estudio ella?

S: Normalista y la otra es periodista, pero la otra se caso y vive en, también se quiere salir pero aquí un periodista, un abogado son ¡pff!

E: Son difíciles

S: Tengo unas amigas que son, enfermeras eso si, que cuidan viejitos

E: Si, eso puede funcionar mejor

S: Pero las 3 que conozco trabajando duro, pero eso la paga bien, ya tienen apartamento, porque gana bien pues. Y se han sacrificado ahí, le han echado pierna dura (golpea la mesa varias veces) trabajan de lunes a lunes. Así si. Eso es lo único que da aquí, a menos que, por ejemplo este muchacho, que la hermana de mis hijos

E: Aha

S: Se apostillaron los títulos, ahora fue que, consiguió trabajo en esa editorial, pero le pagan como, 600 que no deberían. Y aquí el sueldo mínimo es, 200 y pico, que gana la esposa de

E: Y ella ganaba 50

S: Y nada mas el pagar ahí en esa residencia, el condominio, la cosa, se les va la comida...

E: Si, si porque solamente ya un arriendo cuesta 300, 400, 500

S: SI, pero bueno, estamos vivos y con la esperanza que intervengan a Venezuela, de verdad Estados Unidos, claro que eso no sera rápido, pero si, ¡todos! nos volveríamos a Venezuela a reconstruir Venezuela otra vez. Eso lo tengo yo pero así

E: Usted pienso mucho en la vuel...en el regreso, en volver

S: Si, claro... claro y muy agradecida con Chile, por supuesto ¿no? Muy agradecida con Chile. Y lo que me da miedo aquí, nosotros allá por ejemplo, la droga, la marihuana eso es ¡ah! Y aquí es... yo me quedé asombrada, huele a marihuana. ¡Los muchachos! Chamitos 48:19 (revisar palabra se refiere a jóvenes), fumando su marihuana

E: Si, si es muy normal, en la calle

S: En la calle, y la gente así muy, los muchachos, los jóvenes...así como, no sé. Como trastornados, de haber...Razón de, tenían unos tramos, que le digo que en la Tercera Guerra Mundial se acaba el mundo y ya estamos: esta es la Tercera Guerra Mundial. Y los

evangélicos decían, ay esos evangélicos hablan paja, que los hijos le pegaran a los padres, que vendrán enfermedades, que ¡bleeee! Verídico, esta es la Tercera Guerra Mundial. Porque no es Venezuela, bueno Venezuela es el caos, lleva la corona, pero esto es a nivel mundial, desastre. A todo nivel, hasta natural

E: Si, si es verdad

S: Y la gente, la gente...los europeos son apáticos así, no les importa nada. En China yo he visto, pasan, muertos por encima, nada, no les importa o sea... Los americanos les importa el dinero, digo: ¡verga! Entonces Dios, donde esta Dios, ¡Dios mio! que no...se ha perdido eso: de creer en Dios. Que la gente tiene que tener miedo de algo. Nadie tiene miedo, todo el mundo es agresivo, es...

E: ¿Como le ha parecido que somos los chilenos aquí de Santiago?

S: Los chilenos, a veces son apáticos también, o sea (dice algo en voz muy baja revisar 50:27 , retraídos, como retraídos, muy retraídos ¡no sé! Claro no es todo el mundo, porque yo...los, las personas con que trabaja mi amiga por ejemplo, son maravillosos, son dos mujeres con un niño, que viven solas, muy ricos porque, por supuesto pagan una cachifa para

E: Para tener esos...

S: Pero son maravillosos, muy buena gente, aquí yo he conocido ¡ppf! Yo he conocido muchos chilenos muy buenos (pega en la mesa), así...Una vez tuve, me llevaron pa' un bingo ahí en la avenida Independencia, a un bingo, eh como.. y metí mis cosas y empecé a jugar en la maquinita y ¡pa! se acabaron los 1000 pesos, y yo; bueno se acabo, nos vamos. Y una señora que estaba al lado me dijo: usted no es de aquí ¿no? Le dije: no, venezolana, ¡y me abrazo! Y me dio para meter ahí. O sea que: damos lastima. Pero no es gente que...entonces estuvimos hablando y me dijo que: no yo soy de izquierda también porque, no estoy de acuerdo...¡noooo! ¿como va a ser de izquierda? Todo el que hablaba es de izquierda, no entiendo

E: Es que aquí es muy distinto

S: ¿Verdad?

E: Si. Yo todas las personas venezolanas que, que he conversado me dicen cosas como la de usted, porque para nosotros fue al revés. Aquí la dictadura fue de derecha, fue de la gente de derecha

S: ¡Mmmm! fue de derecha, ok

E: Y la gente de derecha fue la que torturo, fue la que expulso, fue la que mato

S: ¡Ah!

E: Fue la que hizo un régimen de dictadura. Y estaba Pinochet que era de derecha. Entonces toda la gente que lucho por hacer la transición a la democracia, es gente que se considera de izquierda porque lucharon contra esa gente de derecha. Y entonces hasta ahora que hay un presidente de derecha, incluso los de derecha, niegan ese pasado oscuro que tienen

S: Aha

E: Entonces por eso todos son de izquierda, pero, no es una izquierda que es como la izquierda venezolana en el sentido que, la mayoría, esta contra estatificar todo

S: Claro

E: ¿Me entiende? O sea por ejemplo, que usted diga: educación publica, educación privada
S: Aha
E: Y hay colegios
S: Es carisimo
E: ¡Es carisimo! carisimo. Y los colegios públicos...y cuando los chicos, salieron a la calle para manifestar que quieren educación publica para todos, mucha gente considera que la educación tiene que ser privada. ¿Ve? Y entonces esas son las diferencias, porque en verdad la izquierda aca no es una izquierda que piense en expropiar por ejemplo, o, o eliminar la propiedad privada. Todo es es privado ¿usted ya vio? Hay hospital publico, hospital privado, hay educación publica, educación privada, ehh, no sé los buses no son del Estado. Los buses son de una empresa privada que concesiona para el Estado. O sea el Estado compra sus servicios. Pero ahí es un dueño, un empresario que pone los buses, por eso es tan cara la locomoción, porque se lucra, o sea
S: Claro
E: No es el Estado que da servicios, entonces es distinto. Por eso cuando usted ve aquí a una persona de izquierda, en Chile, no va a estar necesariamente de acuerdo con las ideas de izquierda que están ahora en el...
S: Hubo un señor
E: Aparte que no son militares, los militares son especialmente de derecha
S: Peero, si, si, aha
E: Acá. Entonces me llama la atención eso porque cada vez que hablo con venezolanos dicen pero: ¡esto es capitalismo!
S: Si
E: Lo que nosotros no teníamos allá nunca fue, porque claro en Venezuela nunca existió esto que es extremo
S: Si, extremo, si... Y el frio, ¡me encanta!
E: ¡Ah si!
S: ¡Uh! fabuloso, fabuloso. Que ya este tiempo, se acabo el invierno pero esta haciendo frio
E: Frio igualito
S: Bien divino, me encanta. Pero me han dicho que es extremo también, ¡cuando hace calor es!
E: Los chilenos se quejan mucho del calor porque va a ver, ahora va a pasar enero, ya paso un enero aquí ¿no? ¿Enero y febrero ya estaba aquí?
S: Si, cuando llegué es que no salia
E: Ese es el calor que hay, que claro
S: ¡No! pero no es mucho
E: Que hay 30, 35 pero es seco
S: Ahh
E: Entonces como es seco, uno no suda tanto, no se como sera en Caracas pero ahí en Dominicana, era caliente pero ademas era muy húmedo, entonces ¡ese es el calor!

S: ¿Tu viviste mucho en Dominicana? ¿O eres dominicana?
E: No, yo viví mucho en Dominicana, yo soy chilena pero viví en Dominicana
S: Ah ok
E: Y antes viví en Italia
S: Ah ok
E: Entonces allá vengo como de vuelta, pero también estuve 10 años fuera de mi país
S: Ah ok
E: Si, si, si. Por estos así que es otra situación
S: Claro, claro
E: Pero por eso entiendo también lo que significa estar lejos de la familia y sobre todo cuando
S: Cuando es involuntario
E: Involuntario
S: Es peor
E: ¿Y las etapas de la vida no? Porque yo también me fui cuando tenia 20 annos entonces
S: Ya
E: Quería hacer mi vida no estaba
S: Claro
E: Los echaba de menos, la navidad el año nuevo, que se yo, pero uno esta tratando de hacer su vida, no esta tan...
S: Claro, claro. Ser independiente es muy importante
E: Claro
S: Como no. Ser independiente de buena forma
E: ¿Y usted allá en Venezuela había tenido problemas de salud? ¿O este fue así como un primer remezon?
S: Si, primera vez, primera vez
E: ¿Hasta entonces usted no tenia ninguna..?
S: No
E: Ni síntomas ni, ni tratamientos, ni nada
S: Nada, nunca
E: Y como
S: Pero es que también los años. Son 68 años
E: Claro
S: Y claro, la, la depresión, de...aunque yo me doy ánimos y mis hijos me dicen: tranquila mama, tu puedes, ¡tu sabes! Y, a veces cuando, esas maquinitas yo ganaba, y ahí aquí antes (golpea la mesa dos veces), por, hay una casa de venezolanos que mandaban, se hacían transferencias de 10 mil pesos que podían, y yo les mandaba a ellos, a mis nietas. Eso era un dineral
E: Claro
S: Pero lo subieron todo a 20
E: Ah mínimo

S: Y ya no pude mas. Me dio el infarto ese (aplaude), entonces no he podido hacer cosas. Y yo estuve aquí una vez (golpea despacio la mesa) y una señora me dijo que consiguió un empleo, y yo: que divino, qué chévere. Porque hay de todo, hay para hacer todo sin esfuerzo, esa señora le dieron empleo para vigilar, a las enfermeras que le dieran, sus remedios y trataran bien a unos viejitos en un asilo

E: ¡Ah mira!

S: Si, ese era su trabajo en 2 horas, 3 horas. Y yo, eso yo le dije a la muchacha de aquí, algo así que no me (golpea la mesa varias veces) con el infarto mientras tengo el tratamiento. O no decir nada, porque yo me siento bien, muy bien. A veces me voy por ahí, a ver si consigo un empleo, a los viejos no le dan

E: Es que eso es muy difícil porque acá tenemos un sistema, en que las jubilaciones están muy bajas, la gente esta ganando muy poco dinero

S: Si señor

E: Y entonces eso significa que los ancianos también están trabajando. Usted ha visto por ejemplo: los que estacionan los autos, a veces en los supermercados

S: ¡En las calles barriendo!

E: Barriendo, son ancianos

S: Son, si

E: Y esas personas claro, ya podrían estar con su jubilación, pero esa jubilación esa pensión, es muy, es muy baja, y entonces tienen que seguir trabajando, y eso es muy cruel

S: Si

E: Entonces eso hace que para las personas mayores que ya están acá, que llegan también es mas difícil buscar

S: Eso es lo que he visto aquí que, este una persona que dure años en un trabajo, no, allá en Venezuela si. Los liquidaban pero con mucho dinero

E: Eso aquí ya no existe. Esa ha sido una de las consecuencias de, de las leyes que quedaron de la dictadura, porque eran tan liberales de tratar de que, producir, producir hasta el final, que las, las jubilaciones se hicieron, se pusieron en manos de, como aseguradoras privas. Entonces ahora ya no es el Estado el que garantiza las, las pensiones, si no que son eh, que lucran con eso. Entonces están ganando muchísimo dinero con los ahorros de la gente

S: Bueno a mi Piñera me cae bien, muy bien, porque esta contra la dictadura de Venezuela y ha hecho mucho (golpea la mesa), ahorita

E: Si ahora esta lo de las visas democráticas...

S: Si (silencio)

E: Y esto de la depresión ¿Es la primera vez que lo sintió con esto o usted tenia también en Venezuela?

S: No yo en Venezuela era muy feliz. A pesar de todo, esto es ahora. Jamas en mi vida pensé, nunca en mi vida, pensé, esto que estoy viviendo ahorita, ¡ppf! yo veía en otras personas... pero ¡vivirlo yo! jamas, jamas. ¿Como son las cosas no?

E: ¿Y acá ha encontrado apoyo en otros venezolanos? ¿O sea se juntan en la posición de los refugiados? ¿Los solicitan desde asilo?

S: ¡Si como no! Pero todo es tan, necesitado o sea. Y nos ayudamos, por ejemplo esta amiga, ahí estoy cómoda, feliz

E: Y eso ha sido gracias a la amistad que han tenido

S: Si, y yo soy muy agradecida, estoy muy agradecida con ella y hago todo lo que puedo: le hago el baño, barro, que se yo,

E: Tratar de tener la casa bonita para que ella esté contenta también

S: ¡Claro pues! Y ellos todos están muy felices también porque yo les cocino, yo no hago nada, no produzco, me están manteniendo, entonces yo no puedo...tengo que hacer algo. Y bueno, cuando me duele que tengo mucho ajetreo, me acuesto un rato, se me pasa y sigo. Y tengo todo limpio, estoy como Blanca nieves y los 7 enanitos

E: (se rie)

S: Y aveces, les hago unas, hice unas caraotas, en Venezuela las caraotas negras, un pabellón

E: ¿Qué son?

S: Son como estos blancos que ustedes tienen aquí, como se llamaban? Las caraotas blancas

E: Qué son para comer? Ah ok ¿como una...sera como cocada?

S: No esto se llama...

E: ¿Empolvado? una cosa dulce

S: No estos son granos, granos blancos

E: ¿Granos blancos?

S: Que ustedes aquí los...lo comen mucho. Peroto (sic)

E: ¡Poroto! Ok, ¡si!

S: Pero nosotros es negra, son negras

E: ¡Ah claro! caraotas

S: Caraotas

E: Claro caraotas negras, ya si, si

S: Y en mi país es el pabellón

E: Si

S: Es caraotas negras, arroz, tajadas, plátano, tajadas, el que se fríe, no el que se come

E: Si, el que se come se llama

S: Cambur

E: Cambur, que allá en Dominicana se llama guineo y que se come, y plátano al que se fríe

S: ¡Ah!

E: Entonces yo me acuerdo que mis amigos venezolanos le decían cambur

S: ¿Y aquí? El plátano es el que

E: Se come, y no hay que se fríe, o sea, ahora se encuentran en el mercado, pero no, es difícil

S: No hay, no hay aquí. No hay yuca aquí. Bueno entonces ese plato, el pabellón es caraota, carne mechada, tajada y arroz, entonces yo le hago su pabellón, su pasta bechamel que le fascina y limpio, están muy contentos y yo también

E: Claro

S: Son gente muy agradable porque peruano esta casado con una venezolana y mi amiga
E: Qué bueno
S: Y la casa, divina. Y una zona bien bonita
E: En Huechuraba
S: Tranquilo, bien bonito. Y tiene, tiene 4 habitaciones
E: Ah pues grande
S: Ellos pagan 400
E: Esta bien por 4 habitaciones
S: Entre ellos, entonces yo quiero. Esos 50 mil en comida que ¡ah! Digo ¡no! ¡pero fabuloso! porque es una colaboración por lo menos
E: Claro
S: Me sentí, bueno...yo le dije a este muchacho: ¡ah no! Dios no me ha desamparado. Fabuloso estamos felices, hasta ahora
E: Que bueno. Eso ya es, son tantas cosas positivas a pesar de todo ¿eh?
S: ¡Si! Y
E: Yo le cuento, el refugio es, para mucha gente se le hace muy largo
S: ¡Uy si!
E: Años
S: Gente que no puede
E: 8 meses, mas 8 meses, mas 8 meses...y, y gente que no tiene nadie aquí
S: ¡Nada! Ni nadie, ¡mm!
E: Durmiendo ahí con los mendigos en los hogares
S: Si señor, si señor
E: Y gente como usted que ha tenido una vida normal, con una casa con una familia
S: Si, claro, si señor. Claro tengo muchos amigos que me han dicho: no yo llegué aqui y tuve que dormir en una plaza, otro que durmió en una iglesia, vivía en una iglesia
E: Imaginate
S: No sé si era esta, no sé
E: Que tengo tiempo que no la veo, y uno trata, que yo, que a mi nada regalado tampoco me gusta o sea, a mi me dio pena: no que una ayuda que...¡ahhh! pero, la situación tiene cara de...
S: Claro pues. Y, bueno...estoy muy agradecida, y por eso cuando Inés me dijo: mira tu quieres, no te da, te puedes poner otro nombre, ¡no!
E: Claro yo le iba a decir
S: ¡No señor! (golpea la mesa) ¡Quiero mi nombre! Por el contrario, y en lo que los pueda ayudar, mira estoy muy agradecida
E: Gracias señora Sulay
S: Y lo que sea
E: También tuvo una buena experiencia ahí en el...
S: ¿Templo?
E: Ahí en el hospital
S: ¿No sentiste algo?
E: No sé porque esto es madera

(se ríen)
S: ¡Hay coño! ¡esta temblando!
E: ¿En Caracas no, no, no tiembla tanto?
S: No, no
E: No, aquí muchísimo, ¿esta en un piso alto usted?
S: ¿Mm?
E: ¿Esta en un piso alto en el departamento?
S: Cuando sentí los temblores si, porque (golpea la mesa varias veces) la hermana de mis hijos vive en el piso 10 (hablan al mismo tiempo), ahora estoy en una casa
E: ¡Ah! perfecto
S: Si, pero, los primeros días: ¡coño esta temblando! (se ríen)
E: Ademas se acaba, se hecha abajo el mundo aquí (se roen)
S: Ahora no, y coño es una , bueno es normal allá
E: Si yo sé
S: pero parece que es grosería
E: No acá no sé, es que se oye como una palabra de afuera, pero allá en Dominicana era grosería
S: ¿Si?
E: Si y era muy divertido porque mis amigos venezolanos escuchaban todo el tiempo a los dominicanos
S: ¡No ya se me ha quitado! porque, esta, esta es la cola. ¡No es la fila! (se ríen), porque la cola es otra cosa (se ríen). Y no sé que paso con el: dame el pito, el pito ¡prr! un pito, no pito es el pene
E: Ah ya! (se ríen) bueno si
S: ¡Toca el pito!
E: Toca pito ¡si!
S: ¡No! ¡no digas eso! ¡qué! (se ríen)
E: Igual que coger, que acá se usa,o sea acá no se usa mucho coger, se usa mas tomar, tomar las cosas. Pero yo sé que en Argentina coger es tener relaciones sexuales, entonces tu dices: cógeme
S: ¡Cógeme!
E: (se ríe) ¡estas diciendo eso!
S: Si, yo vi un pleito en una, en una micro. Un latero, eran asi gente, como latero, mendigo, una mujer y un hombre. Sus, así todos feos cortados, asi como...y subieron los dos a vender, algo unas curitas
E: Si que se suben a, que venden pero también piden en el fondo
S: ¡Ah! Entonces el hombre parece que, que tropezó a una mujer que estaba ahí. Y la mujer: no sé que gua, gua, ¡huevon! Parece que huevon es
E: Huevon es fuerte si, si
S: Es fuerte aquí
E: O sea claro, si, si te tratan de huevon es como que les estas insultando
S: ¡Ah! mira que, o sea porque...entonces la mujer, la, la latera que estaba con el hombre, le dijo: mira porque tu le dices ¡huevon! ¡ah! ¡ah! ¡cabeza de árbol!
E: Comenzaron a insultarse

S: Eran, trivialidades las que le decía: ¡care' mango! ¡cabeza de!
E: (se ríe)
S: Y yo: estos son los insultos de (hablan al mismo tiempo)
E: De Chile. No pero acá también hay cuando se, se quieren pelear, empiezan con cosas fuertes, que quizás uno no sabe que significan eso
S: Si
E: Pero para del huevon para adelante hay mucho, hay otra palabra que es bien fea acá, que es culiado
S: ¡Ah! ¡culiado!
E: ¡Eso si! de ahí para adelante cuando ya están en ese tono significa que se están yendo ya a la pelea, al pleito mas duro
S: ¡Si! y la mujer se quedo, una muchacha joven, que yo, yo...a veces me creo superman siempre, protectora de mis hijos de mi familia, protectora de la gente. Yo decía, ¡esta! le hacen algo a esa mujer, bueno, yo la defiendo, porque no vaya a tener un cuchillo o algo
E: Por eso aquí la gente no hace nada, la gente es muy indiferente cuando pasan estas cosas
S: ¡Si! ¡apática! O sea
E: Por eso cuando usted decía que ser apático, claro, porque la gente tiene mucho miedo de meterse en cosas, entonces
S: Si
E: Usted va a ver que le pueden pegar en la calle a alguien y nadie se va a meter
S: ¡Hay no! ¡imaginate!
E: O bueno, poco
S: Si, si
E: Pero es...una la piensa dos veces antes de meterse
S: Yo he visto dos cosas aquí que me impresionaron: una vez iba caminando por Independencia yyyy veo ahí una mujer que lanzo
E: ¿Qué se lanzo?
S: Y estaban gritando, porque estábamos, y la mujer se lanza, se medio paro y un negrito la agarro, eso salio hasta en el periódico
E: ¡Si! si, si si...hay Dios mio que se había tirado de un edificio
S: Se tiro una mujer y yo iba ¡ah! Y cuando, como que se agarro de, de un balcón, rebajo un poco la, y un bicho, un negrito de esos, ¿como se llama?
E: ¿Un haitiano?
S: Un haitiano. Salio corriendo, y ¡ta! ¡ta! y la agarro (al unisono). Y la mujer no se murió
E: Hay Dios mio
S: Y el presidente le dio 2 millones, de peso, al negrito. Después vi en el periódico, pero yo vi, yo iba pasando y vi a la mujer de verdad
E: Hay Dios mio, qué fuerte
S: Eso me impresiono mucho
E: ¿Y como se ha sentido tratada por los chilenos? Porque los chilenos no, también discriminamos
S: ¡Bien! No te digo que esa señora en el bingo hasta me abrazo y me metió 1000 pesos mas en la maquinita, y otros, otra gente, chévere, en el metro

E: Nunca ha sentido que la han...
S: Que yo pregunto mucho: mira, la transferencia, ¿no es transferencia?
E: La combinación
S: La combinación, entonces me dijo, dos veces. Un señor me dijo: tu no eres de aquí. No, venezolana, ¡ay! ¡me abrazo!
E: ¡Ah mira! ¡le ha ido bien! O sea ha tenido buena experiencia
S: En cambio con los colombianos no. La gente, parece que a los chilenos no les gustan ni los colombianos ni los haitianos
E: Por eso le digo que, es por el color de piel
S: Si, sera, pero los colombianos ¡por tramposos! (golpe en la mesa) en cambio con los venezolanos la gente ha sido mas receptiva
E: Si ademas que los venezolanos que han llegado son muy educados
S: Aha
E: Como usted dice, gente profesional
S: Muchos profesionales
E: Entonces por supuesto el trato es otro ¿no? porque son personas que en general tienen mucho mas estudios de lo que tienen los chilenos. Entonces tratan bien, tu vez ahí en un café llega ahí un mozo, es un venezolano y todo (hablan al mismo tiempo)
S: En la micro; buenos días, ¿como estas? permiso
E: Claro, buenos días como estas? eso ya en Chile, la gente ya lo perdió, no lo hablan
S: Si
E: Uno a veces va a comprar, te dice: es el primero que me saluda, dame esto (chasquido de dedos), no, no es buenos días
S: Claro
E: Mire le quería comentar que acá, están mis datos por si usted necesitara cualquier cosa. Este es mi teléfono celular
S: Ok
E: Yo igual me voy a llevar ese dato a ver si le puedo dar alguna información sobre lo del metro. Esto, esta es la dirección donde yo trabajo, bueno y este el teléfono de la Universidad, pero cualquier cosa que usted necesite este es mi teléfono, ya para que lo tenga, porque así como yo me quedo con sus datos
S: Ok, Alejandra
E: Alejandra Carreño, ok
S: La hermana de mis hijos se llama Alejandra
E: Ah también con la que vivió
S: Si, bella, linda
E: Trate de seguir en contacto con esa niña
S: No, claro
E: Que le hace muy bien a la gente, a todos. Yo tengo un hijo de cuatro años ¡y también! cuando necesito consuelo, le digo él, él voy a ir a hablar con mi hijo
S: Hoy la iba a ver, hoy día la iba a ver y mi amiga me dijo: mira lo que te traje, lo que le traje para mañana que vas a ver a tu, a tu Samanta

E: Ah va con regalo
S: Y no la vi, no llego
E: ¿No pudo llegar? ¿Qué le paso?
S: No porque yo estaba en el, consultorio, pidiendo una cita porque me siento mal y en la terapia me dijeron ve al consultorio
E: Ok, porque es el dolor de espalda lo que tiene ahora
S: No, debajo del pulmón, o sea es algo... entonces, yo fui a pedir la cita, la hora
E: Claro, pedir hora al consultorio
S: Me la dieron para diciembre, no hay. Pero si me quedaba , a ver si alguien no fue , me vengo
E: ¡Ah! claro
S: Pero dije no, yo tengo una cita a las 11
E: ¡No! mire ¡puf!
S: Entonces, voy a ir o mañana o ahora
E: Ok, a ver si es que falto alguien que pudiera darle la
S: Si, exacto
E: Ok, mire y acá esta lo del reembolso porque aquí, le damos reembolso, una compensación por el, por el haber venido, no alcancé a preparar un sobrecito que tenia cuando llegamos antes
S: ¡ah! ¡*Oh my god!* ¡Me parece...!
E: Pero esto es por venir, por el gasto que es, ahí hay una pequeña compensación
S: ¡Ah!
E: Por poquito que...
S: Me da pena pero...
E: No, no mire, esto no es por caridad ni nada, es porque usted nos dio su tiempo y porque es parte de ayudar a que la gente pueda, moverse ¿no? cargar la bip
S: Si, no se lo voy a meter a la bip
E: Y para que pueda llamar por teléfono, lo que sea aquí ¡hay todo! 1:16:25

AUDIO 2

S: Porque eran, fueron como 9 o 10 días de

E: Claro si son como 10 días de viaje

S: Entonces mientras estos se bañaban, estos se quedaban, y ellos, bajaron y eso fue en pleno Perú

E: Mira
S: En el centro de Perú, y era ya de noche, pero tipo con armas largas y todo. Y ella se resistió pero ¡juii! el tipo traqueo de
E: Esta armado, no tengo nada que hacer

S: Si, y la dejaron viva de broma, la pistola
E: ¡Claro! si encontraron una señora ahí
S: Con otra señora, un muchacho, y un joven. Se, bueno por lo menos no la
E: Claro, llego viva
S: Y ahí le quitaron 1000 dolares que traía de 900 y pico de dolares que tenia en el bolso. Y aquí llego, sin medio, y sin teléfono, porque uno se aprende los teléfonos
E: Claro, como se va...
S: Y da la casualidad que una de las muchachas que estaban viajando, ella se quedo sin... ¿como se llama?
E: Sin celular, sin cargador
S: Sin cargador! Se le acabo la batería, y la muchacha, quien la iba a esperar, que el muchacho que la iba a recibir
E: ¡Ahh!
S: Y le dio el numero, y de eso que la robaron, la muchacha estaba del otro lado y le dijo: mira yo tengo el numero! del que me diste, creo que no lo he borrado
E: Mira menos mal
S: Y fue que pudo comunicarse con... y la espero en una estación central, y ella ahí, sola robada
E: ¡Dios mio!
S: Así llego. La paso fea también
E: ¿Y usted como fue que eligió Chile? Porque usted fue por una familia, un familiar indirecto
S: Si, y ella, porque ella es santera, ¿sabes?
E: Si
S: Como hay católicos buenos y malos, hay santeros buenos
E: Si pues
S: Hay gente buena y gente mala. Yo la admiro muchísimo, una tipa muy inteligente, tan inteligente creyendo en eso, pero, hay que respetarla. Entonces ella y que echo sus caracoles, le echaron unos caracoles, porque ella quería ir a Panamá
E: Claro, en Panamá hay muchos venezolanos también
S: Si, que nos lo quieren porque
E: Que no los quieren
S: Panamá, Perú y Chile. Entonce los caracoles le dijeron que Chile
E: El mas lejano
S: Entonces ella para escaparse de Venezuela siendo militar, imaginate
E: Y ella no pidió asilo, claro porque si es militar
S: No, ella no
E: ¡Ah no!
S: No porque, o sea no...ella no quiso, ella le da, no confía, militar no confía en nadie. Dice si no, si saben que estoy aquí me mandan a matar. Yo le hablé de esto le dije: mira una entrevista, entonces me dijo no, porque me da miedo, van a saber donde estoy yo. Miedo ella no, me dice que van a saber donde estoy
E: Y empieza de nuevo todo

S: Y no creo, o sea, yo te digo, yo a veces he sentido: ya no tengo miedo, para nada, por el contrario. Y pongo por ahí en las cadenas esas: ¡desgraciados! si que les queda poco tiempo, no me importa

E: Siempre fue opositora

S: Y algo que yo nunca tuve, es un odio que les tengo. Mira yo perdí una amistad, todavía es chavista (golpea la mesa) ¡madurista! Esta con Maduro ¡ahh! No puedo, no puedo, yo no quisiera, no la soporto, la borré de todo, que las cosas que pone a favor del gobierno, la quité

E: Claro es que llega un momento en que uno no soporta eso

S: No soporto eso, que todavía haya gente que, que apoye a esa gente, y pasando trabajos que es lo peor. O sea tan inteligente, tan gradual, o sea la universidad no paso por tu

E: Parece que no pasa por eso

S: No entiendo

E: Parece que no

VENEZUELA Y VENEZUELA (MADRE E HIJO)

I Bueno, entonces ¿hace cuanto tiempo que están acá, en Chile?

P: Bueno, yo particularmente tengo dos años…

I: Mhm…

P: Yo llegué aquí el quince de agosto del dos mil dieciséis… por un tema… muy distinto a migrar.

I: Mm

P: Mi hermano mayor que tiene veintiséis años viviendo acá en Chile, a él le habían diagnosticado… leucemia, afortunadamente un tipo benigno. Y eh… el necesitaba una quimioterapia, entonces me vine a prestarles apoyo.

I: Perfecto…

P: Eh… En un principio, yo me quedaba por tres meses, o sea, yo había logrado conseguir un boleto, a por fuera, no desde Venezuela, logré que por fuera me lo consiguieran por tres meses… Y… eh, en teoría que regresar en agos… en noviembre

I: Mhm

P: Pero al momento de regresar, a mí me dio mucho nervio, yo tenía a mi hijo menor en… Venezuela, mi hijo mayor vive en Argentina.

I: Okey

P: Y en ese momento, pues decidí irme Argentina para poder regresar y volver a tener tres meses más como para dar un tiempo… a ver que decidía…

I: Mmm

P: Porque siempre estaba renuente, porque lo tenía él en Venezuela, más mi papá más mi mamá

I: Okey

P: Que están en condiciones bien específicas ellos allá. Y… bueno, así estuve entrando a Argentina y viniéndome de nuevo, o sea, me iba donde mi hijo…

I: Siempre con visa turista entonces…

P: SÍ, estuve con visa de turista hasta que en un punto la situación a mi hijo menor se le puso… bien difícil, quedarse en Venezuela. Bien sea, también por persecuciones… eh… que hacían a los estudiantes universitarios, como en la situación que él presentaba en casa, entonces…

I: Mhm…

P: Eh, con… mi hermano acordamos traerlo. También por fuera busqué la manera de conseguir un pasaje y él llegó en septiembre del dos mil diecisiete.

I: Okey

P: En ese momento pues a mí la situación ya me cambiaba, pues ya no podía entrar entrando y saliendo

I: Mm

P: Así que ambos decidimos solicitar refugio…

I: Mmm… Que esa fue digamos pues la razón principal por la que yo no solicitaba refugio, era porque lo tenía a él en Venezuela y no sabía en qué momento me tenía que devolver.

I: Claro

P: Pero al traérmelo acá, ya la situación se puso… un poco más seria.

I: Mmm

P: Y… este, desde… septiembre del dos mil diecisiete hasta ahora es que estamos nosotros en condición de solicitantes de refugio

I: solicitantes…

P: Sí

I: Solicitantes. ¿Cómo fue el proceso de… la solicitud? O sea, ¿Cómo tomaron la decisión de usar este… esta… modalidad, no cierto? Y… y ¿Cómo ha sido el buscar como se hace, que caminos hay que seguir?

P: Sí, este… Bueno… eh, toda la información está en web, pero a mí me asesoró una amiga colombiana

I: Okey

P: Que pasó por todos estos procesos en Estados Unidos y en Canadá y entonces en vista de la condición que nosotros teníamos en Venezuela, porque nosotros allá habíamos sido víctimas de…. Eh…. de extorsión… secuestro… no, sí, directamente en la familia habíamos tenido secuestros, extorsión, eh… amenazas, todo. Este… ella nos sugiere la modalidad, porque nosotros estando allá no teníamos conocimiento de esta modalidad.

I: Mmm… Claro. ¿Es la amiga colombiana entonces, quién les sugiere que pueden apelar a eso?

P: Sí, que vas a la comisión, y sobre todo porque podíamos tener acceso a un tipo de ayuda

I: Mhm…

P: Que le favorecía a él también como joven

I: Claro

P: Hasta ahora no se ha dado

I: Mhm…

P: No se ha dado este tipo de ayuda, o sea, ese sueño de migrante…

I: Mmm…

P: Pero… por lo menos a nivel de… de documentación, hasta ahora es lo que nos ha funcionado.

I: Okey

P: Aunque con esta nueva modalidad que ha tenido el presidente Piñera…

I: Mhm

P: Esa modalidad también nos hubiese servido

I: ¿La de la visa por, de responsabilidad democrática?

P: Sí, también hubiese servido, porque si nos ponemos a sacar cuentas, entre eso y la modalidad que nosotros tenemos ahora no hay mucha diferencia…

I: Claro.

P: Entonces… eh… Hubiésemos también apelado a esa, pero… por nuestra condición, decidimos que el refu… y decidimos quedarnos también por eso, porque nosotros somos candidatos a ser refugiados

I: Claro

P 2 (voz masculina): No, y que todavía el gobierno de Chile no había tomado esa decisión de la visa…

P: Exacto, ya teníamos un tiempo recorrido, así que entre eso y renovar la visa, decidimos renovar la visa de solicitantes de refugio.

I: Okey, ¿porque al principio dan una visa temporal por un tiempo? ¿Cómo funciona?

P: Sí, cada ocho meses tenemos una visa…

I: Cada ocho meses, mmm

P: Eh… donde se estampa que somos solicitantes de refugio

I: Okey

P: Entonces cada ocho meses tenemos que renovar el rut… y el proceso es bien engorroso y cada vez está siendo más… engorroso…

I: ¿Cuánto tiempo han renovado? ¿Varias veces?

P: Esta es la segunda vez que renovamos

I: Okey. O sea, por lo menos ya dieciséis meses de…

P: Sí, tenemos dieciséis meses, pero… este proceso, donde renovamos ahora la visa a la anterior, esta fue… catastrófico. Por la cantidad, por la cantidad de solicitantes… de visa

I: Eso… ¿Qué fue lo que vieron en ese proceso? O sea…

P: El primero fue bien expedito y…. y teníamos que como una atención privilegiada

I: Mhm…

P: En este nos pusieron con todos los solicitantes de cualquier visa y…
I: De cualquier visa…

P: Y…

I: ¿A dónde tuvieron que ir, a Eleuterio Ramírez? ¿A esa oficina…?

P2: Sí, a las dos modalidades, o pides la cita o a Eleuterio Ramírez

P: Claro. En un principio nos dijeron que podíamos hacerla presencial, fuimos presencial y fue…

I: Un desastre…

P: Fue horrible, porque claro, también estaban activando la… ¿la oficina de Matucana?

I: Mmmm… Sí, matucana

P2: Sí, Matucana

P: Y… entonces los funcionarios nos decían cualquier cosa con tal de que nos fuéramos… fue horrible. Hasta carabineros estuvo ahí presente organizan… Fue horrible, entonces después pedimos la cita y nos fuimos a… a la cita, que esa si fue rapidísima.

I: ¿Ahí en Eleuterio Ramírez?

P: Mhm

P2: En Eleuterio Ramírez

I: Ah, okey. Con cita funciona

P: Sí, con cita sí, pero sin cita… y como estaban mudándose a la nueva oficina, de verdad que fue terrible.

I: ¿Y había muchas solicitudes de asilo? Eh… de refugio, perdón, ¿O era por la cantidad de solicitudes en general?

P: En un principio también, si hubo muchas solicitudes de refugio

I: Mhm

P: Cuando empezamos nosotros…

I: Sí

P: Y luego como nos derivaron con todas las demás visas… todos

I: Ahí fue un desastre…

P: Sí, todos… todos

I: Y en general hasta este momento ha sido principalmente como, eh… cosas más burocráticas o han tenido también que presentar testimonios… ¿Cómo funciona todo eso…?
P: La primera vez si tuvimos que presentar el testimonio. Y… burocrático no ha sido tanto, más que ir…

(Se escucha que P 2 habla al mismo tiempo, no se entiende min 06:17)

P: Hacer, decir que queremos presentar… Cuando fuimos ahora a esta renovación, lo que se nos había dicho la primera vez era que con un mes de anticipación manifestábamos que queríamos renu… a ver…

I: Hacer la solicitud

P: Hacer la solicitud, renovar, perdón

I: Ah, perdón

P: Y ahí…. Eh, nos presentábamos al mes, cuando se vencía la visa. Cuando fuimos a hacer la solicitud de renovación, nos dijeron que ya no era necesario, que con ir y presentar…se en el momento de la renovación ya era suficiente…

I: Okey

P2: Pero al final nos cambiaron otra vez, y ahora sí nos tenemos que volver a presentar un mes antes

P: ¿En serio? Ah…

P2: Un mes antes para…

P: Entonces hay mucha… todavía como mucha ambigüedad

I: Hay mucha desinformación

P: Entonces, si a eso te refieres con burocracia, hasta ese nivel ha habido burocracia… Que todavía no tienen como bien estandarizado el proceso.

I: Y el proceso, en cambio, de los testimonios ¿Quién es el que los toma, que es lo que busca? ¿Ustedescomo se presentaron en ese proceso?

P: Eh, son los funcionarios que están en la oficina de ACNUR de… de

P2: No, los funcionarios de extranjería

I: Son de extranjería, claro

P2: Son los funcionarios.

I: Mhm

P2: Ellos son los que toman la declaración y ellos deciden si… está apto o no el testimonio para…

P: Exacto

P2: Ser solicitante de refugio

P: Sí

I: Okey

P2: Porque según la ley chilena, después tienes que volver… te llaman a otra entrevista, que ahí es donde deciden…

P: ¿Con quién es la entrevista?

P2: Es con el… si recuerdo bien, es con el jefe de extranjería

I: Mhm

P2: Dos funcionarios de extranjería y dos funcionarios del ministerio de relaciones exteriores

I: Okey

P2: Y ellos son los que deciden si… pasas al estatus de refugiado

I: Okey

P: Sí

P2: Yo…

P: ACNUR no tiene nada que ver desde ese punto

P2: Puede estar… es que pueden estar en… varias personas presentes, número u otro…

I: No, parece que no son quienes toman decisiones

P: O sea

(Se traslapan voces, no se escucha con claridad 08:00)

P2: Ellos tienen voz, pero no votan

P: Exacto

I: Exacto

P2: Los que tienen voto son los cinco funcionarios del gobierno

I: Exacto. ¿Y entonces esas entrevistas ustedes las pasaron ya? Eh, o sea…

P2: No, pasamos la primera

P: La primera es donde… por eso tenemos la condición de solicitantes. Digamos que en la primera entrevista es donde ellos te dicen si tu testimonio aplica o no aplica para ser solicitante

I: Mhm

P: Eh… fueron bien receptivos…

P2: Sí

P: Fueron bien amables…

P2: Ninguna queja…

P: Sí… este… sí

P2: Con todo… con todo son…

P: Desde principio a fin, desde que uno llega a la oficia el proceso es bien agradable…

I: Okey

P2: Lo único sí, es la desinformación

P: Eso…

P2: Tú preguntas y nadie sabe, o sea, te mandan para un lado, te mandan para otro…

P: Pero, claro es… es entendible por cuanto ellos… El proceso se les salió de las manos…

I: Mmm… absolutamente

P2: Claro

P: Entonces ya llega un momento en que lo que ellos tenían estipulado para un principio ya no les funciona

I: Mmm

P: Porque ya hay muchas personas

I: Claro

P: Entonces hasta ese punto es entendible

I: Claro, no, si es un fenómeno explosivo en ese sentido… A pesar de que como ustedes dicen, los colombianos estaban presentes hace mucho tiempo acá

P: Sí

I: Pero de alguna manera no… no sé por qué, eh, esto se… como que se…

P: Sí

I: Se salió de las manos

P: Sí, porque ya muchas personas lo están tomando para la facilidad de los documentos, o sea, ya no es tanto por su condición, porque es… Sí, veamos la situación que tiene ahora Venezuela, todos los venezolanos somos candidatos…

I: Mmm…

P: A ser solicitantes de refugio

I: Mmm… claro

P: Porque la… violencia generalizada, en cualquier ámbito en cualquier nivel. Pero muchos connacionales lo utilizan para nada más la facilidad de documentos, tienen las primeras visas y ya después renuncian. Entonces, lo que están haciendo es ralentizar, ojo, yo no soy quién para decir si ellos son o no son candidatos

I: Claro

P: Desde mi punto de vista todos somos candidatos a solicitantes… Pero, utilizan esta modalidad más por la facilidad de tener documentos

I: Porque se supone que… ¿Y porque circula esta idea de que hay alguna ayuda o no? Por qué usted decía que habría…

P2: Es cierto

I: ¿Habría sido mejor para… para él?

P: Sí… Sí, en un principio es… se hablaba… (suspira) Claro, no fue el origen por el cual nosotros solicitamos eh… la condición, hicimos la solicitud. Sino que se hablaba en un momento, si no hay casa, si no hay dinero, que sí sé que muchos venezolanos llegan acá, este, se podía tener acceso a un tipo de ayuda

I: Mhm

P: En nuestro caso no era lo primordial, porque nosotros tenemos aquí a mi hermano y…

I: Claro

P: Y eso estaba como… solventado

I: Mhm

P: Pero yo sí sé que vienen muchos venezolanos sin nada….

I: Nada…

P: Con esta promesa de que acá los pueden ayudar, siendo… solicitantes de ref…, con lo cual no es cierto… Porque hay como niveles.

I: Mmm

P: Dependiendo de quién esté peor es que se puede ayudar

I: Claro

P2: Depende también de la ley del país donde estés, es que se de esa ayuda

I: Mmm

P: Sí, exacto y los montos y todo esto, entonces… Bueno y eso básicamente…

I: ¿Y cómo llegaron a FASIC?

P: Nos dicen eh…

P2: (Ininteligible min 10:45)

P: Desde que te informan, te dan el papelito y te dicen si usted necesita ayuda, diríjase acá.

I: ¿Esto desde misma extranjería?

P2: Ininteligible min 10:59

P: Sí, te da el mapita y todo te dicen… Pero, lo que, si te dicen desde un principio, que es solo sí lo necesitas realmente…

I: Mmm

P: Porque hay muchas personas, ¡y es verdad! Hay muchas personas que vienen en condiciones que ni ropa traen adecuada al clima de acá… no traen comida, no tienen donde quedarse, entonces, claro, uno entiende, uno entiende el criterio de que nosotros no estamos tan mal como para pedir la ayuda. Que hay gente que si lo necesita.

I: ¿Y se acercaron igualmente acá?

P: Sí, es que hay que acercarse…

P2: Sí, sí, exactamente

P: Como venir a ponerse derecho, mira nosotros somos solicitantes…

P2: Nos entregaron dos planillas en extranjería y tú tienes que traerla acá, para que guarden tu planilla…

I: Ah, perfecto, para un registro acá… okey

P2: Tienen un registro de las personas solicitantes

P: Exacto, sí. Y supongo que ellos harán el estudio socioeconómico y todo esto, y allí verán…

I: Y ahí toman un poco sus datos de… de la situación que… en la que están

P: Sí, de repente sí… eh…. Cuando venimos y traemos la… la planilla, no nos entrevistan con una…. Visitadora social

I: ¿Asistente social?

P: Asistente social… Pero… si uno lo requiere te… te hacen la agenda.

I: Okey. ¿Cómo fueron orientándose en Chile? O sea, jaja, como se fueron… no sé, estudios, trabajo, salud… ¿Cómo fue ese primer proceso?...

P: Claro, básicamente por tener familia, ellos nos orientaron

I: Mmm, okey

P: Pero lo demás se va aprendiendo… en el día a día, según como… la suerte que tengas. Nosotros hasta ahora hemos intentado trabajar dos veces, en el primer trabajo si no me fue muy bien, era una empresa inestable…

I: Mmm

P: Inestable en el sentido que, este, dependía de una empresa más grande, y esa empresa no les renovó contrato y nos quedamos todos nosotros en el aire.

I: Mmm…

P: Y ahora, más o menos la misma dinámica, estamos en una pyme, y las pymes son bien inestables.

I: Claro…

P: Él ahora si está trabajando en una empresa más grande

I: Mhm

P2: Más grande…

I: (Ininteligible se interponen voces min 12:46)

P: Sí, yo estoy trabajando en mi área, pero es una pyme

I: Okey

P: Entonces no hay garantía, por ejemplo, el mes que viene…

I: Depende de la ganancia de cada mes…

P: En cambio él si está… el sueldo es poco, pero por lo menos está en una empresa que garantiza que lo poco se le va a pagar

I: Se lo pagan. ¿Tú habías estudiado entonces en Venezuela? ¿Eres estudiante?

P2: Yo llegué hasta bachi…

P: No, tiene hasta el tercer semestre de administración…

P2: Exactamente, tercer semestre de administración…

P: Sí, pero, o sea, él tiene su bachillerato completo, su básica, su todo completo…

P2: Educación media completa

I: Claro, lo que sería el cuarto medio completo

P2: Exacto…

I: Y luego lograste hacer algunos años universitarios ¿no?

P2: Exactamente

I: Que estuviste… y ¿tenían ideas de seguir estudiando acá?

P: Sí,

P2: Sí ahí… jaja

P: Sí, lo que pasa es que está el proceso este… ya él está reconocido del proceso de básica y media de Chile

P2: De media, sí

I: Ya convalidaron los títulos…

P2: Ya convalidé el título acá de educación media

I: Okey

P: Ya lo que nos falta es como la…

P2: Presentar la PSU

P: La PSU… No sé en que estatus está eso, ya llevo un año aquí y no sé, no sé cómo lo lleva. Y… pues las opciones de… que puede estudiar acá, según lo que le ha gustado… porque le gusta la diplomacia, no puede acá…

I: Claro

P: Tiene que ser chileno

I: Mmm

P: Entonces… este… Por ahí de repente… Que opciones tiene que pueda pagar, porque los dos estamos en la misma condición. Yo no le puedo pagar estudios. Y que lo vaya llevando más o menos a la carrera que… le hubiese gustado tener

I: Claro, en un poquito de tiempo ir a… ir a…

P: Sí

I: ¿De qué ciudades son ustedes?

P: Eh, Caracas

I: De Caracas

P2: De la ciudad capital

I: Sí… ¿Y como se llegó a esta situación?, fueron, ¿me decías que fueron varios años en que empezaron a ponerse más difíciles las cosas?

P: Sí, nosotros somos de caracas, pero este, los últimos años estuvimos viviendo en el pueblito de donde es mi papá

IM Mhm

P: Que es una colonia alemana

I: Okey

P: Y…

I: Herika Helliman, ¿verdad?

P: Sí jaja. E iba… netamente este… se depende de la agricultura allí

I: Mmm

P: Todo este proceso que lleva el gobierno que está instaurado en Venezuela logró destruir la agricultura, entonces ya nosotros nos quedamos sin medio de producción, sin…

I: Porque ahí tenían una… hacienda, alguna…

P: Sí, es decir, la empresa de nosotros se caracterizaba porque compraban las cosechas y las colocaban en las grandes cadenas de supermercados

I: Perfecto

P: Es tema es que… los agricultores se vieron… mermados en su producción porque el gobierno venezolano no facilitaba los dólares para comprar los químicos que se necesitan para la cosecha

I: Okey

P: Entonces… netamente por la manera en que se cosecha en esa zona, son plantas de cuatro estaciones y necesitan de ciertos químicos que las hagan creer que están en el proceso, estando en el trópico, ellas tienen que creer que están en el proceso

I: Aaah, perfecto

P: Entonces se utilizan unos químicos específicos, que, para poder tener acceso a eso, necesitas dólares

I: Mhm

P: Y el gobierno… este gobierno… este régimen, no da los dólares

I: Mhm

P: Y entonces ya ellos no tenían acceso a eso

I: ¿Y eso desde hace cuánto se empezó a crear? Como… ¿Esa situación?

P2: Es que los dólares tienen…

P: Lo que pasa es que …

P: Los contra cambiaron

P: Lo que pasa es que ha sido una dinámica que se ha generado por la, la misma manera en como ellos han administrado los recursos del país

I: Mhm

P: Entonces, el control de cambio en Venezuela está del dos mil tres, desde el dos mil tres

I: Perfecto

P: Mientras estuvo la bonanza petrolera eso no hacía mella. Pero cuando bajó el barril de petróleo, eso se empezó a… sentir más fuerte, porque todo el ingreso de dólares de Venezuela entraba por PVS, por petróleos de Venezuela, por la renta petrolera

I: Claro

P: Entonces al tener menos dólares, ellos no van a distribuir ni para medicina, ni para salud, ni para… comida, ellos se lo van a robar. Porque toda esa arca entra por ellos. Entonces ellos tienen ahora menos dinero, dicen, para que voy a dar para esto… ellos que resuelvan. Y eso es lo que están haciendo, y por eso que Venezuela está en la situación en que está, porque lo que entra por dólares, ellos no lo van a distribuir equitativamente, ellos roban primero y después ven… si queda algo

I: Mhm… Entonces ahí se empezó a…

P: A deteriorar…

I: Mermar la… la producción agrícola

P: Y todo, agrícola, salud, comida, todo todo

P2: Sí, mientras subió más el gasto público tu vas a ver la…

P: Exacto. Y a eso súmale el gato público y que todo lo regalan, todo…

I: Mmm

P: Todo lo tienen…

P2: Subsidiado

P: Subsidiado… hasta el mismo dólar está subsidiado, nosotros podíamos tener acceso a un dólar subsidiado, que evidentemente en un momento dejaron de darnos lo que se llamaba el cupo de dólares

I: Mhm…

P: O s ea, síentran mil dólares, ellos no lo van a repartir en los venezolanos

I: Mhm

P: Se lo quedan

I: Claro

P: Los venezolanos que vean que hacen… Nosotros en realidad somos un daño colateral

I: Mmm

P: Y a parte de eso, que tenían todo subsidiad para tenerlos a todos contentos, entonces tienen que ver que… Es que yo no sé, ni siquiera se pagan los subsidios… Porque ya la gasolina los (ininteigible) dejaron de estar subsidiados…

P2: Dejaron el mercado… (ininteligible, se intercalan las voces min 17:53)

P: Estalló esa bomba…

P2: Era como una burbuja

P: Ni siquiera burbuja, era como una olla de presión, que a tenían contenida, la tenían contenida y ya eso explotó

I: Entonces ustedes vieron fueron viendo… ustedes fueron a vivir a este pueblo ¿no?

P: Si, nos fuimos con mi papá y ellos terminaron ahí su escolaridad, mis dos hijos y… este, nosotros por tener una situación privilegiada entre comillas, privilegiada en el sentido de que estábamos un poquito mejor que los demás

I: Mhm

P: Empezamos a recibir amenazas de extorsión, de secuestro… ya me habían llamado para avisarme que me lo iban a secuestrar a él, que estaba ya en el último año de colegio. Este…

I: ¿Cuándo todavía estaba en el colegio?

P: Sí, entonces en lo que él terminó lo mandé con su papá a estudiar en otra zona de Venezuela, pero resultó que ya en ese momento, cuando él estaba estudiando, ya los estudiantes universitarios estaban siendo estigmatizados

I: Mmm

P: Entonces los perseguían, él tuvo compañeros que…. Que tuvo que correr para que no lo… no lo secuestraran.

P2: Los servicios… los servicios de inteligencia y…

P: El brazo armado del chavismo

P2: Los famosos colectivos, la obra militar de ellos…

P: Sí… Inclusive tanto en la legal como en la ilegal… la legal y la paralegal, porque son legales pero paralegal

I: Claro

P: Este… y entonces empezaron a perseguir a los estudiantes…

I: Pero eso lo perseguían por ejemplo… en un contexto de protestas, o en un contexto cotidiano… ¿Cómo eran las… las persecuciones?

P: Se empezó a estigmatizar que los estudiantes universitarios se estaban quejando por nada… Algo así

P2: Sí, exacto

P: Entonces, por ejemplo, era muy común oír en las calles personas que estaban en disgusto con los estudiantes que decían “ellos son estudiantes” en lugar de decir delincuentes

I: Mhm

P: Decían “ellos los estudiantes” Entonces, ya se tergiversaba un poco lo que era el estudiante comunista, que… este protestaba por su derecho, al estudiante que ahora está protestando y no tiene por qué protestar. Entonces, ¿viste como se van cambiando las visiones?

I: Mmm

P: el comunista se queja del estudiante, porque resulta que el estudiante es burgués, es más o menos lo quese presentó en la china de Mao con los estudiantes. O sea, si tu eras estudiante o eres profesor, eras de una elite que no tienes derecho, porque no eres campesino

I: Mhm

P: Eso

I: ¿Y eso también estaba vinculado con el hecho de que ustedes fueran hijos de colonos o no…?

P: No necesariamente… No… no…

I: ¿Era más una cosa de clase, no?

P: Sí…

P2: Sí, exacto

P: Exacto, el rechazo que nosotros…. Este, sufrimos no fue tanto de… de etnia porque, tanto… o sea, en Venezuela tanto…

I: (Ininteligible min 20:22)

P2: Si, exacto

P: Pero sí con estatus, entonces… el el… el hecho de ser rubio si implica que tú tienes dinero, y en algún momento se cree que tienes dinero. Pero no es tanto por ser rubio, sino porque tienes dinero

I: Claro

P: Eso

I: Mhm… entonces se empezaron a hacer naturalmente considerados como contra el… gobierno en ese momento

P: Sí, puede ser

P2: Exacto

P: Aunque no tengas dinero, eres burgués

P2: Porque estas en contra del… (es interrumpido)

P: Porque estás en contra o porque tienes acceso

P2: … Ser

P: Por ser opositor al chavismo, por tener acceso a recursos que… el común no tiene, que es estudios

I: Mmm

P: O porque de repente te planificaste un poco mejor y tienes un poquito más de dinero o porque tienes una empresa propia

I: Las empresas propias…

P2: Exacto

I: ¿Y las empresa la expropiaron, la atacaron?

P: No, la tuvimos que cerrar, tuvimos que hacer cierre técnico

I: Okey

P: Porque al no tener cosecha que comprar… tuvimos que paralizar todo

P2: (ininteligible, intenta hablar, pero es interrumpido min 21:14)

P: O sea, llega un momento en que yo tuve que enfrentar a mi papá y decirle, estas financiando a los supermercados que no necesitas que los financies… Claro, para él eso fue… un golpe al ego porque estas personas fueron amigos de él…

I: Claro, eran sus clientes me imagino ¿No?

P: Sí, se convirtió en cliente luego de haber tenido una amistad, entonces, así como, ¿cómo les voy a quedar mal si son mis amigos?

I: Mmm

P: Pero los estas financiando y ellos no necesitan que tú los financies… Como tú dirás ¿Cómo los estábamos financiando?

I: Mmm

P: El traslado, nosotros teníamos que movilizar camiones, y a ellos les teníamos que pagar un flete y teníamos un montón de gastos de logística

I: Mhm

P: Que a fin de cuentas lo único que estaba saliendo bien era el supermercado, porque cuando nosotros recibíamos el dinero, ya eso no valía, o sea, ya eso era más costoso…

I: Mmm

P: Entonces cuando mandábamos las cotizaciones se tardaban en aprobar las cotizaciones, no ralentizaban a nosotros y cuando aprobaban esa cotización, ya estaba diez veces por debajo de lo que en ese momento nos estaban vendiendo… Entonces lo que él estaba haciendo era financiar eso…

I: Claro

P Gastábamos nosotros camón, personal… Logística, y cuando el supermercado nos lo pagaba, ya eso no era lo mismo…

I: Mmm

P: Entonces… Dígamos una dinámica hubiera sido que el supermercado nos pagara en el momento del despacho…

I: Claro

P: Pero no nos lo iba a pagar en el momento del despacho, nos lo pagaba a treinta días, y con la dinámica que había en la economía de Venezuela, treinta días ya eso…

I: Se había devaluado…

P: Muchísimo… Entonces, hasta que mi papá lo entendió, quiso cerrar la empresa porque es que ya no tenía donde vender, porque si hubiese conseguido cosecha que vender, hubiese seguido en lo mismo

I: Mmm

P: Entonces… eso… eso, eso es un hito nada más… A ese hito súmale la delincuencia…

I: Mmm

P: Que los camiones no pueden salir sin un seguro, sin seguro todo riesgo

I: Wow

P: Y ya yo no podía pagar el seguro

I: Mhm

P: Por lo menos, yo tengo un camión, por lo menos ese camión no lo podía pagar… Porque ya me costaba mucho más de lo que el camión me generaba…

I: O sea, cualquier desplazamiento era carísimo…

P: Y… y peligroso...

I: Mmm

P: Porque eso era una cosa, la otra es que te pueden matar al chofer ¿cómo le respondes tu a la familia?

I: Mmm

P: La familia iba a venir por tu cabeza

P2: Lo otro es lo que hay en el camión también…Porque piensan que… Quieren alimentos de primera necesidad

P: Esa es otra cosa… alimentos…

P2: De primera necesidad

P: Pero subsidiados, o sea, arroz, leche

P2: (ininteligible, lo interrumpen)

P: Sí, pero hay alimentos de primera necesidad que no son… Porque tienes el arroz que no está subsidiado, la pasta que no está subsidiada… que son las que se importan, pero son alimentos básicos subsidiados…

I: Ya… y esos camiones los tienden a asaltar…

P: Te esperaban afuera del supermercado, o cuando tu llegabas

P2: O en la autopista…

P: Estabas fuera del supermercado… cuando llegabas, te paraban, de hecho, a uno de mis choferes se lo hicieron, lo pararon con pistola y le pidieron la guía… la guía de traslado

I: Mmm

P: Porque es que están informados… Y cuando le he mostrado la guía que ellos vieron que no eran artículos… este que estaban…

I: Subsidiados

P: Subsidiados…

P2: Y le pidieron abrir atrás también pues…

P: Para corroborar, porque yo en la guía también me puedo poner cualquier cosa

I: Claro, claro

P: Ellos no están al tanto de saber si lo que yo estoy poniendo es verdad, o sea, ni siquiera la guardia nacional, que es la que te tiene que vigilar, te hacía eso… Sí lo hacían, pero no de esa manera… Y ellos venían y con la (ininteligible) te cotejaban…

I: Entonces, ¿pero esas eran figuras legales se supone? Eran de…

P: ¡No! ¡Delincuencia!

P2: Delincuencia

I: Ah, eran delincuentes, pero con una… una acción…

P: Sí, con una actitud de gobierno…

P2: Acciones violentas…

P: Porque es que el gobierno no los va a parar… al gobierno les conviene eso… Entonces hasta ese nivel uno se tiene que dejar… dejar, dejar. Entonces… imagínate, me hubiesen matado a ese chofer… yo no tengo como responderle a la familia, ni vendiendo el camión, ni vendiendo lo que tuviera yo le podía responder a la familia.

I: Claro

P: La familia iba a venir por mi cabeza… Porque él me estaba trabajando a mí. Y entonces no importaba la mercancía, no importa el camión, la vida… Que nadie te la garantiza.

I: ¿Y esos fueron como los últimos… años?

P: Sí, cuando… yo me vine… Eh, cuando, en el dos mil seis la situación ya estaba… ahora la situación debe estar súper peor…

P2: (Ininteligible min 25:13)

P: Tan peor está la situación que nos dicen que no digamos que estamos bien, porque a mi papá lo pueden secuestrar para que nosotros mandemos dólares…

I: Claro

P: Entonces… siempre le digo a mi papá no digas que estamos bien, no digas que tenemos trabajo… di que estamos mal, que yo me voy a regresar… Porque… si se llegan a enterar que nosotros… podemos generar algún tipo de dinero lo pueden secuestrar a él para pedirlo…

I: Y eso... tenía que ver… ¿Ustedes tenían alguna pa... acción política más pública…? ¿Eran… opositores más públicos…?

P: O sea, la gente sabía que, por ejemplo, yo mis hijos, no estaban de acuerdo con el gobierno, porque no participábamos activamente en… en…
P2: Mítines del gobiernos

P: Eh, sí… algún tipo de reunión, por ejemplo, nos obligaron a todos a entrar… es que… te lo juro, se me parece tanto a la China de Mao, nos obligaron a todos a entrar en la… este… consejos comunales

P2: En los consejos comunales

P: Porque si tú no pertenecías a un consejo comunal, entonces los recursos que te… ofrecía el gobierno… no te lo derivaban. Por ejemplo, sí tenías una carretera que había que reparar, si tú no estabas en el consejo comunal, directamente te… te aislaban. Ahora ya ni siquiera es a ese nivel, ahora es comida, si tu no perteneces a un consejo comunal, no te dan comida… Ahora es peor, ahora tienes que sacar una tarjetita, que se llama…

P2: El carnet de la patria

P: El carnet de la patria… que, si tú no tienes el carnet de la patria, no te dan comida. Ahora peor, mi mamá hubo que sacarle el carnet de la patria, porque si ella no tiene el carnet de la patria no le dan su jubilación, no le dan su pensión… No tiene acceso a nada… ¡A nada!

I: O sea…

P: Por lo que ella lucho toda su vida

P2: O sea, porque tenemos uno que es la cédula de identidad de nosotros…

I: Claro

P: El rut

P2: El rut, y sacaron esto que es un… el carnet de la patria que es del partido del gobierno.

I: Es como de afiliación al par…

P: ¡Exacto!

P2: (Habla y es interrumpido, no se entiende min 27:00)

P: ¡Es una manera de humillarte!

P2: Ahá

P: Una manera de humillarte porque… o sea, si tu no tienes eso no eres venezolano

P2: O sea, tanto que si tú no lo tienes… (es interrumpido)

I: … Claro y no teniendo es como…

P2: Exacto, ni subsidio de la gasolina

P: Eres apatrio…

P2: Apatrio

P: Eres un tipo de apatrio… no te lo dicen, pero te van metiendo en el saquito…

P2: Exacto

I: ¿Y ustedes tuvieron que participar de estos consejos comunales…?

P: Sí

P2: Sí, para el carnet de la patria…

P: … El carnet de la patria… me vine antes, pero este año yo tuve que movilizar a mi mamá para que le pudieran sacar el carnet de la patria

P2: A mí me quisieron intentar, pero yo me negué

P: Mhm, sí

I: Y esas cosas entonces, de alguna manera ¿estaban detrás del hecho de que los empezaron a perseguir… o solamente el hecho de tener el dinero…?

P: Es que cualquier cosa lo puede disparar…

P2: Mhm

P: Aunque tú no tengas dinero… principalmente van tras la cabeza de la gente que tiene dinero

I: Mhm

P: Este… a un primo lo secuestraron… él estaba dejando un… eh… o sea, de lo último, el caso más terrible, porque hubo… hubo otros hechos que involucraron incluso muertes de… de… de guardia nacional que no tenía que haber ocurrido, pero que ocurrió… mi primo estaba dejando un… uno de sus obreros a las siete de la noche, que ya a las siete de la noche es de noche en Venezuela

I: Claro

P: Y ahí lo secuestraron a ambos, se los llevaron…

I: A él y al obrero… ¿y su trabajador?

P: Sí… se los llevaron, pero, en el camino dijeron "no, este no tiene dinero, suelten al obrero". Se llevaron a mi primo este… se lo llevaron para, nosotros decimos rancho, a las casas muy humildes. Y lo tenían allí, y quienes lo estaban cuidando eran estudiantes de básica… lo torturaban… Y con la camioneta de él, y la que se robaron, fueron a robar otra similar y resulta que cuando vieron que era un guardia nacional lo mataron. Lo dejaron ahí con la esposa y lo mataron…

P2: Un guardia nacional es como un carabinero

P: es que el que está entre carabineros y militares…

I: Okey

P: Y… este, lo dejaron ahí tirado con la esposa, lo dejaron moribundo desangrado… En la huida impactaron otro vehículo donde murió una niña de dos años, o sea, fue una cosa así…

I: Demasiado…

P: A ese nivel está Venezuela… Dan el rescate, damos el rescate de mi primo… Lo sueltan, como dos tres días después…

I: O sea que tenían identificado quién era su familia…

P: ¡Sí! (ininteligible)

P2: Hacen como un…

P: Sí, un sondeo de mercado, por eso que a mi me llaman para decirme mira, sabemos dónde… si tú no nos pagas esta cantidad, sabemos en que colegio está tu hijo, en que horarios va… O sea, i papá tuvo que pagar. Cuando mi papá se tardó, mataron n obrero y lo dejaron en la puerta de la casa… A mi me desertaron los gritos de la mamá. Menos mal que en ese momento él no estaba conmigo… Él estaba con su papá… Pero así, a ese nivel… A te tardaste, mira esto es lo que te va a pasar…

I: Y eso fue eh… ¿Se tardó en dar el… la extorsión que le habían pedido por él?

P: O sea, no por el primo mío, sino que… ya está en una extorsión directamente… a mi papá, mira tienes que darnos tanto… Cuando mi primo paga su… este… cuando pagan su…

P2: Extorsión

P: Eso… sí, cuando pagan por él, para soltarlo, no recuerdo ahora el término, este… A los quince días lo están llamando de la cárcel… ah, porque la extorsión es de la cárcel, desde la cárcel

I: Ah, la extorsión es de la cárcel

P: La extorsión es desde la cárcel… Llaman de la cárcel a mi primo y le dicen "bueno, ahora nos tienes que dar tanto" Hubo que llevar el dinero y… Cuando estamos en la cárcel pagando la

extorsión, le dicen a mi primo… ah no… a su hermano, perdón, porque no fue mi primo en persona. Le dicen bueno tranquilo, que cuando necesitemos más te llamamos.

I: O sea…

P: De a cárcel… Sí, ¡sí! Es que el negocio está en la cárcel… Y tú dirás ¿y por qué no salen? Yo tampoco entiendo…

P2: Porque prefieren… Ellos prefieren estando presos porque están más protegidos… Adentro estando en prisión que fuera…

P ¡Que afuera!

I: Mmm claro

P: Es más, sacaron una banda de la cárcel porque ellos no tenían por qué estar presos… Y los sacaron… Así, a ese nivel, ese nivel que tu dirás no entiendo, no… nosotros tampoco entendemos, pero sí…

I: Llegó a un nivel de…. total, de….

P: De perversión, de sociopatía. Ya esto es un nivel sociopático, la delincuencia en Venezuela que se está levantando es sociópata…

P2: Pero es que se decía que la ministra que mantenía (ininteligible es interrumpido por participante)

P: … Por cualquier cosa

P2: Por cualquier cosa, entonces, el ministerio de las prisiones, la ministra tenía un amante que era uno de los jefes de una prisión… Entonces esto era….

P: Pero jefe… del lado… que nosotros llamamos pran…

P2: Pran, exacto

P: Era un pran… de la parte de ser procesado… procesado, reo…

I: De las personas que han cometido delitos

P: Exacto, de los delincuentes. Ella está asociada a eso, entonces ¿Qué te puedes esperar tú?

I: O sea… ¿Cómo se vivía esto a niveles de trabajos… de estudios? O sea, ¿ustedes podían hacer una vida normal en todo esto? ¿Cómo fue avanzando esta experiencia?

P: Como toda indefensión que te van creando desde un principio, tu lo vas sorteando hasta que llega un momento en que tú dices “ya no puedo más con esto”. Porque el tema en Venezuela es que hay una delincuencia desbordada y hay escasez…

I: Mmm…

P: Yo creo que con las dos cosas nos e puede vivir

I: Claro

P: O sea, ya llega un punto en que yo me resistía a hacer fila para tener acceso a la comida… Entonces, ya llegaba un momento en que costaba conseguir los alimentos… Por que yo no los iba a exponer a ellos, él si tenía compañeros que sus familias los ponían a hacer filas… para tener acceso a comida.

I: Mhm

P: Yo a ellos no los iba a poner en eso… o sea, yo me resistí hasta el último y preferí migrar… antes de que ellos tuvieran que hacer fila para conseguir comida.

I: Mhm

P: Que ya creo… creo que es lo último de la humillación. Y no solamente comida para nosotros, comida para sus abuelos, porque yo no iba poder poner a mi mamá a hacer una fila…

I: Claro

P: O sea, yo me resistí… me resistí lo más que pude a eso.

I: En cambio, su hermano se había venido antes a Chile…

P: Sí, (ininteligible min 32:56)

I: Ah, claro había venido por otros… o sea, en otro momento, por otros motivos…

P: Mira, el tema de él, aunque fue distintos motivos, fue en vista lo mismo
I: Mmm

P: Él se había casado… en Venezuela con una chilena que sus papás habían tenido que salir por ser comunistas…

I: Mhm

P: Por cuanto ellos tuvieron el beneficio de retornados. Pero en el momento en que este, hubo ese beneficio él tenía un hijo y ya el vislumbraba lo que iba a ocurrir en Venezuela. Y se vino con su…

I: Su hijo y su esposa

P: Y su esposa para acá. Pero ya él veía lo que venía…

I: Hace veintiséis años

P: ¡No! Casi veintiséis años era terrible vivir en Venezuela todavía

I: Bueno, yo no conozco bien toda la historia, pero claro, pero hubo un momento en que Venezuela parecía en cambio como un… sueño. Estaba super bien…

P: Es rico…. Es una ilusión que se creó, porque teníamos una cantidad grotesca de dinero entrando, pero…

I: Claro, eso. Hablaba de los dólares venezolanos ¿no? Y Miami…

P: Pero todo fue una ilusión…O sea, en Venezuela digamos que hubo dos grandes ilusiones: cuando se quemaron las ¿venezuelas sauditas? (no se entiende bien min 34:00) Que fue en los años ochenta…

I: Claro

P: Que fue una ilusión, que no sé porque se generó en ese momento…

P2: Por… estuvo…

P: También un alza de petróleo también…

P2: Es que se tomó un buen… un alza… eh… un… (ininteligible min 34:13)

P: Mmmm sí…

P2: Al resto de occidente empezó Venezuela…

I: Donde se transformó en…

P: Tuvo ese boom

P2: Tuvo ese boom

P: Y se creó la ilusión que teníamos un gran poder adquisitivo, cosa que es mentira. Porque te estás dando cuenta ahora que no tenemos…

P2: Y que más que la democracia en esa época era… ser igual de corruptos, pero por lo menos era eficiente…

P: Mmm exacto

P2: Eficiente, porque por lo menos…

P: Invertían…

P2: Invertían en hospitales, tenían…

P: En infraestructura…

P2: En infraestructura…

P: Que la mayor parte de la infraestructura que hubo en Venezuela la dejó la… la derecha, el último… dictador de derecha que hubo en Venezuela, que fue el que dejó la gran infraestructura

P2: Infraestructura

P: O sea, que cuando llegaron los… los gobiernos… democráticos, digamos que aprovecharon de esa infraestructura y se montaron en la ola…

I: Mmm…

P2: Exacto

P: Ellos se montaron, siguieron, continuaron, no fue del mismo impulso, pero continuaron…

I: Continuaron…

P: Hasta que se montó el chavismo, cuando se montó el chavismo fue el acabo ese…

P2: Pero si fueron ya cuarenta años que se cansaron de ese bipartidismo…

P: Mhm…

P2: Salió… el fulano…

P: Sí, pero yo creo que ya venía un proceso de ellos por detrás… Del… del comunismo cubano, ya venía por detrás. Porque cuando se montó el Caracaso, que fue el intento de golpe a un gobierno… democrático

P2: El Caracaso fue… primero y después… el golpe…

P: Ahá, pero cuando se montó el Caracaso ya ellos estaban… ya el chavismo estaba por detrás, a mí eso nadie me lo quita de la cabeza… Porque justamente cuando hubo el Caracaso son los que están ahora montados en el gobierno… ¡Ah, qué casualidad! Entonces… Pero, en lo que se montó Chávez vino la de (ininteligible min 35:52) … Hubo… también por este tema del petróleo… un… (ininteligible min 35:57)

P2: Sí, un… (ininteligible min 35:58)

P: Una vulgaridad de dinero que entró a Venezuela, que se dieron el lujo de regalar… de votar, menos de invertir. O sea, lo que invirtieron fue tan mal lo que invirtieron, que nada de eso yo creo que queda…

P2: No… nada, nada…

P: No, o sea… Es decir, si termina el chavismo y comienza otro gobierno, nada de eso funciona…

I: Claro

P2: Nada, o sea, la infraestructura que tiene hoy Venezuela es de… los cincuenta.

P: Y es la que…

P2: Las renovaciones que hicieron en el gobierno democrático…

P: Claro, porque, por ahí lo que intentó hacer el chavismo ¿es qué? Un tren que intentó hacer…

P2: Y… se robaron todo jaja

P: Eso está abandonado… Todo lo que tenga que ver con (ininteligible min 36:22). El metro, la ampliación del metro fue lo único… que puede quedar…

I: ¿Y ustedes alguna vez habían pensado en migrar antes de hacerlo?

P: Yo sí

I: Mhm

P: Yo sí, porque… justamente como mis dos hermanos habían migrado… mi hermano se fue a Argentina y mi hermano se vino para acá… Es así como, yo no me quiero quedar aquí… Además, que ese desastre peculiar de Venezuela a mí nunca me gustó, ni siquiera de pequeña. Pues porque es muy sui generis…

I: Mmm…

P: Es sui generis, muy, es sui generis… O sea, está este tema… tropical como la… la vida irresponsable, con… el este… digamos eh… una… una pasividad… para… para las cosas buenas y una hiperactividad en las cosas malas… Muy propio del trópico…

I: Mhm…

P: O sea, si te pones a ver en la franja tropical es como muy característico esto ¿no?

I: Mmm…

P: Muy relajados para una cosa… muy alborotados para otra… entonces todo el tiempo un bochinche… Eso a mí nunca me gustó, pero porque los tenía a ellos que siempre… postergué la migración.

I: Mmm

P: Hasta que ya no nos tocó de otra que migrar… Nos hubiese gustado migrar de una manera un poco más controlada…

I: Mmm…

P: Pero no… Eh… digamos que fui pasiva en ese sentido, no te voy a decir que me agarró… de improvisto ¿no?

I: Mhm…

P: Ya yo…

I: Había algo siempre en la historia de….

P: Sí, previamente, pero fue muy pasivo, yo fui muy muy irresponsablemente pasiva…

I: Mhm…

P: Porque yo ahora viendo atrás, yo dije yo pude haber hecho cosas que a nosotros nos resultara… en cuanto ellos tuvieran la mayoría de edad y no lo hice… Me quedé como esperando a que no ocurriera…

I: Mmm… Como…

P: Ese tio de indefensión, que te quedas esperando que no ocurra, pero yo sabía que iba a ocurrir…

I: Mmm…

P: Todo esto que está ocurriendo en Venezuela a mí no me extraña

I: Mmm…

P: Inclusive el éxodo tampoco me extraña… Nada de esto me extraña… Pero yo sí fui pasiva…

I: Mhm

P: Pero yo si se lo puedo adjudicar a un tipo de indefensión…

I: Mmm…

P: O sea, como que te dicen que no lo puedes lograr que te tienes que quedar ahí y en algún punto tu… lo crees y esperas que no pase…

I: Bueno, y la figura de la familia, me imagino también ¿No?

P: Sí

I: Los padres…

P: También, y la comodidad y creo que la… zona de confort también va en contra. Porque claro, tenía una vida cómoda… tenía mis ingresos…hasta que eso me lo cortaron…

I: Claro

P: Entonces tú dices, ¿si sabías que venía porque no te moviste antes? No lo sé…. No sé porque no me moví antes… Tal vez no fue la capacidad de ver que podía hacer afuera… Algo tuvo que haber ocurrido... en mí.

I: ¿Y nunca pensaron en Alemania? Pensando en que ustedes son hijos de colonos…

P: Sí, pero por ese lado tenemos todas las puertas cerradas. Porque… mis ancestros están en Venezuela… Mira se está bajando del caballo (ininteligible, habla muy rápido min 39:03) Yo creo que mil ochocientos…

P2: Mil ochocientos treinta…

P: Mil ochocientos treinta y cinco…

I: ¡Mmmm! Ya no son generaciones recientes… Sus papás son venezolanos…

P: ¡Exacto! No tenemos ni si quiera el idioma…

I: Claro… lo iba a decir… el… el o la… el pasaporte, que es…

P: No… no, ese tipo de comentarios… Nosotros si acaso mantenemos el apellido y el fenotipo.

I: Mhm

P: Porque… eh… este… Alemania creo que reconoce hasta la cuarta generación

I: Mmm

P: Nosotros ya somos como la sexta…

I: Okey

P: Y… este, eso fue por un convenio que se hizo con… Codasi, un geógrafo que vive en Venezuela, italiano, que el gobierno de Alemania y el gobierno e Venezuela… de la parte venezolana para ese tiempo, y se trajeron a estos colonos, porque siempre se supo que los alemanes eran buenos colonos…

I: Mmm claro…

P: Se contrataban para colonizar, por eso…

I: Sí, si, si… por eso en el Sur hay muchísimos

P: Sí, de hecho, en Venezuela sería el segundo intento de colonización, porque ya primero hubo… una empresa austriaco-alemana… Sí, austriaco-alemana

P2: Sí, austriaco…

P: Austriaca, que… contrataron para la colonización y formaron dos pueblos en Venezuela…

I: Mmm…

P: Pero, tal fue el nivel de desafío que yo creo que dijeron no mira… jajajaja no nos renueven el contrato

I: Jajajaja

P2: (ininteligible min 40:17)

P: Nos siguieron… y luego viene esta segunda… eh… oleada de alemanes, digamos, que fue más o menos la época en que empezaron todos a colonizar en América…

I: Mhm…

P: Y… este… de allí vienen ellos, pero en nuestro caso particular, tuvieron menos suerte…

I: Mmm…

P: Fueron como más… a la deriva…

I: Mhm…

P: Fue menos organizado…

I: Menos organizado…

P: Entonces los dejaron en una playa… A su… a la buena de ellos, y ellos se fueron abriendo camino…
I: Mhm

P: De hecho, esa playa es muy conocida, porque los negritos que estaban allí son de ojos claros…

I: Ah, porque había mezcla…

P: Ellos fueron (ininteligible min 40:48) jajaja

I: Jajajaja

P: Entonces, formaron estos dos pueblos, la colonia Tobar que a nivel… digamos turístico es bien reconocido, y el pueblito donde mi papá que es más rural…

I: Mhm

P: Más agrícola… Entonces está estos dos pueblitos ahí. Y ya por ahí nosotros no tenemos oportunidad… Por el lado de mi mamá sí teníamos oportunidad, porque su familia era española…

I: Mhm…

P: Y son más cercanos… Pero se presentó el problema que cuando yo voy a recabar la información de mi abuela… de mi yaya, antes se acostumbraba cambiarse los nombres…

I: Mmm…

P: Entonces mi abuela está reconocida con un nombre y… en su cedula aparece con otro, así que (ininteligible min 41:29) me ha tocado de inclusive sumarios… un cadáver….

I: Para poder reconocer que esa personas es la misma…

P: Claro, y como un poco pruebo yo que María del Pilar, con lo que ella ella fue reconocida, eh… presenta… es la misma María Josefina que es mi abuela…

I: Son dos nombres distintos…. Jajaja completamente

P: Entonces ahí no…

I: Ah… y ahí se cerró la… posibilidad

P: Sí

I: Y de Chile… ¿qué conocimientos tenían, que… que proyecciones, ideas sobre este lugar donde estaban partiendo?

P: Bueno, ocurrieron dos modalidades, teníamos la versión de nuestra familia, que por su puesto era la más real…

I: Mhm…

P: Y… que bueno, este es un país duro para migrar, que este…

I: De la familia que estaba acá…

P: Sí, mi hermano básicamente

I: Claro

P: Que es… un país muy costoso, pues lo que es la realidad… Y … eh la visión que se presentaba, digamos a nivel macro…

I: Mhm

P: Que es la Venezuela, la, perdón, Suiza latinoamericana… eh… blablablá, que no es la real…

I: Mhm…

P: Digamos que este fue como el marketing que se hicieron para traer la versión… la inversión extranjera… pero… Obviamente está la versión real, que es que cuando uno llega acá es un país muy duro, es muy fuerte… Y… por eso te digo yo nunca me vine para acá con… intención de migrar, yo me vine por otro motivo…

I: Mhm

P: Me vine para ayudarlos…

I: Claro…

P: Porque ellos no solamente tenían un problema de salud, sino que tenían un niño (ininteligible min 42:46)

I: Mhm

P: Entonces… o sea…

I: Habían dos razones por las que venir….

P: Muy fuertes por las que necesitaban ayuda de familia…

I: Mmm…

P: Y bueno, eso fue por ese lado, pero mis intenciones nunca fueron migrar… Me quedé como migrante porque… entre eso y volver al desastre de Venezuela, prefería quedarme acá intentándolo… Que regresar y traérmelo a él, inclusive… O sea, jugármela… ya como que mucho mejor que irme allá… Aunque yo tuviera casa, tuviera carro, tuviera ingresos, auto…Es… era mucho mejor quedarse aquí sin nada… Que volver allá, porque allá nuestra vida está en riesgo, nadie nos garantiza la vida, nadie. Y ya por el hecho de no tener un… una (ininteligible min 43:27) menos….

I: Mhm…

P: Más o menos decente, ya… implica que tienes dinero…

I: Claro…

P: Por lo menos no lo están pasando mal… Entonces ya, era eso pues, jugármela acá

I: ¿Y qué fue lo más difícil de llegar a Chile?

P2: Yo creo que el clima… El clima… jajaja

P: Jajaja

I: Sí, te veo bien abrigado… jajaja

P: Nosotros siendo tropical… pues sí… nos pegó. Y tropical de un buen clima, porque como vivimos en montaña no es el calor…

I: El calor…

P: … asqueroso que hace en otras partes… Sino… vivíamos como en una primavera todo el tiempo…

I: Mhm…

P: Y vivíamos como en una especie de burbuja, porque en esa montañita todos somos familia, todos nos conocíamos… el… no entraba mucha gente rara…

I: Mmm….

P: Los que entraban por lo general eran delincuentes… o turistas…

I: Mmm….

P: Pero… no era tanto, así que… Era un sitio como que bien… bien reservado…

I: Mhm

P: Entonces claro, como venir y… y eh… ah… Acostumbrarnos a un clima, otra idiosincrasia, otras cuestiones… Pero… ha sido bueno… Yo lo comparo con irse a vivir con la suegra…

I: Mmm jajaja

P: Yo digo que migrar es irse a vivir a la suegra, porque estás en una casa que no es la tuya…

I: Mmm…

P: Con unas condiciones que no son las tuyas… eh… tienes que cumplir tareas a las que no estás acostumbrado… entonces… básicamente es como irte a vivir con la suegra.

I: Mmm… Y les costó como esta di… me imagino porque además es como… uno viaja y como que pierde lo que tenía ¿no? O sea, tú doces tenía casa, tenía carro… tenía…

P: Sí

I: Eh… eso… ¿cómo se vive aquí…?

P: Bueno… Eh… en este caso ha sido dos modalidades distintas porque… eh… En un principio aquí moverse es muy fácil…

I: Mhm…

P: Así que por ese lado para nosotros no ha sido un problema

I: ¿Cómo moverse en la ciudad?

P: Exacto, o sea, extrañar un auto, la verdad es que yo no lo extraño…

I: Mhm

P: En Venezuela lo necesitas

I: Mhm

P: Para poderte mover, porque el transporte público es… es bien deficiente más el peligro…

I: Calro

P: Porque a ti te asaltan en el transporte público…

I: Claro

P: Osea, más o menos algo así como lo que ustedes pueden enterarse en Río de Janeiro…

I: Mmm…

P: Algo así

I: Mmm

P: En Venezuela, en todos lados

I: No hay que subirse a… una guagua ni nada…

P: O sea, te subes a un… autobús, pero corres el riesgo de que te atraquen… Eso no nadie te puede garantizar que no ocurra…

I: Mhm…

P: Y… este bueno, entonces acá movernos en transporte público de verdad que ha sido como que bien…. Bien de elite jajaja

P2: Jajaja

P: O sea, Así como (ininteligible min 45:46) que tenemos un transporte eficiente... A un costo de eficiencia…

I: Claro… Si porque los santiaguinos se quejan del transporte público jajaja

P: Se quejan porque pueden… porque cuando no pueden no se quejan…

I: Mmm…

P: O sea, nosotros no nos quejamos de las cosas que… vemos bien… Que… o sea, sí se pueden mejorar, pero de verdad es que lo tienen tan bien y es tan eficiente que… ¿para qué nos vamos a quejar?

P: Mmm…

P: O sea, si nosotros venimos de un desastre… Igual con carabineros, se quejan de carabineros y… eh… es la policía… mejor de Latinoamérica… Muchos dicen ¿de qué se quejan? Si los de nosotros son delincuentes… y no tienen idea…. De lo… caballeros que son carabineros…

P2: (ininteligible min 46:22)

P: Son unos caballeros… Pero bueno, por eso te digo… la gente se queja porque puede…

I: Porque puede… jajaja Claro, acá los puntos de referencia son distintos ¿no?

P: Claro, porque ya cuando salen a otro país y tu te das cuenta que la policía no es tu amiga…

I: Mmm…

P: Que te maltrata, y que si puede te abusa, hasta te secuestra o te roba… ¿Porque a mí él que me dice? Que yo estoy bajo amenaza de secuestro, me lo dijo un policía… Entonces tu no sabes si es el policía el que te quiere secuestrar…

I: Mmm…

P: O que él ya se enteró porque tiene amigos delincuentes… que te quieren secuestrar, entonces…

I: Se confunde todo ¿no? Quien te protege…

P: Cualquiera puede ser…

P2: Yo… yo le cuento, porque tenía un primo que era…. como de PDI,

I: Mhm…

P2: (Ininteligible min 46:47) Que… Si contaban que ellos mismos, los mismos detectives desaparecían cuerpos… y nadie sabía… desaparecían cosas…

P: Sí…

P2: Y nadie sabe donde están, los entierran en… (Ininteligible min 47:07)…

P: O sea, imagínate tú teniendo familiares que están en ese medio, que te dicen… (Ininteligible hablan al mismo tiempo min 47:16) nosotros nos encargamos de desaparecer los cuerpos…

P2: (Habla entre medio, no se entiende min 47:16)

P: Y tú por ser familiar, también corres el riesgo…

P2: Exacto…

P: Acá tu eres el familiar de fulanito… tiene… no tengo la culpa… Nadie te garantiza… o sea, esa estabilidad emocional… Por eso lo que está allá es muy insano… Porque no hay… no… no puedes tener una estabilidad emocional… Entonces, si la delincuencia allá son sociópatas… o sea, estamos hablando de niños…

I: Claro

P: Que ya se cuentan cuantos muertos pueden tener… niños de once, doce años… Que ya cuentan los muertos que tienen encima. ¿Qué puedes esperar tú de esa sociedad? Ya eso es sociopatía…

I: Mhm…

P: O sea, el delincuente en Venezuela es irrecuperable…

I: Claro..

P: Muchos dicen… no… los adultos te podrás… (ininteligible) A parte de eso, que el tiempo de vida es muy corto, bajo ese ritmo de vida…

I: Claro, porque es muy fácil morir…

P: Claro, entonces van por todo, es tu vida o la de ellos… Van por todo…

I: Y esa sensación de… inseguridad que vivían allá, contrasta con… como ustedes están viviendo acá… en términos de seguridad… o siguen teniendo todavía estos recuerdos como de…

I: Sí, sí. Evidentemente, es una fase, nosotros vivimos paranoicos… Eh… algo muy sencillo es al cruzar la calle… Tu te das cuenta del venezolano, porque el venezolano se frena…

I: Mmm…

P: ¿Por qué nos frenamos al… cruzar la calle? Porque hay motorizados… que te pueden llevar… Y no se van a parar a ver si tu estás bien. Entonces… el… la primera reacción que tenemos nosotros… al enfrentarnos a cruzar la calle es pararnos a ver si los autos primero s e detienen y… sí no, nos queda la sensación de que puede venir un motorizado… Porque los motorizados no respetan nada… o sea, ellos no van a respetar un (ininteligible) rayado… y sí por ahí te le cruzaste te llevan…

P2: Otra cosa sería la inseguridad en moto…

I: Mhm…

P2: Porque ellos mismos roban en moto, entonces…

P: Claro, por eso digo, lo más sencillo sería pararse a esperar que todo el mundo se detenga, pero no cruzar la calle… Acá no, acá cruzan y… y que también es irresponsable eso…

I: Mhm…

P: Pero… lo… el otro caso sería eso, que de repente venga un motorizado y te venga a atracar…

I: Mmm

P2: Entonces, todavía queda un poco de miedo…

P: … Mhm, sí…

P2: De que vengan a robar en… moto

P: Claro, que acá no hay tantas motos como….

P2: Por eso, eso… (lo interrumpen, ininteligible)

P: ¡Claro! No existe esa modalidad de robo…

P2: Sí, pero…

P: Hay muchas otras modalidades…

P2: … Da esa sensación como de…

P: (lo interrumpe) Imagínate, un nivel básico que yo estoy cruzando la calle y estoy viendo entre los autos… entre… que no venga un motorizado a llevarte…

I: Mhm…

P: Es muy como… si tu reconoces a un venezolano… De repente…

I: Lo vez mirando…

P: Sí, los autos…que no venga un motorizado… a llevárselo por el medio… se llevan lo que sea.

I: ¿Y cómo han logrado ustedes superar todas estas cosas? Esto es un… es un tema como bien recurrente, en tema de refugio, entre personas que… eh… se quiebran en todo este proceso, y no lo soportan… personas que logran… reconstruir una vida digamos… O sea, todo esto…

P: A nosotros todavía nos está costando… Nos está costando porque… ese, cuando nos hablabas de cómo lo estábamos llevando… que yo te decía que teníamos dos modalidades…

I: Mmm…

P: Hacía referencia a eso… primero que… este, todavía no nos acostumbramos a que uno acá puede estar más relajado…

I: Mhm…

P: Entonces, vivimos todavía acelerados…

I: Mhm…

P: Y lo otro es que tenemos una dinámica familiar que no nos permite sentirnos cómodos… Porque hay temas en la casa de… como estamos arrimados…

I: ¿Ustedes están viviendo con la fam… con su hermano?

P2: Sí

P: Mhm…. Y ahí hay un tema bien fuerte… que por eso estamos nosotros así… yendo a terapia con I, entonces eso tampoco nos permite… sentirnos cómodos… O sea, todavía sentimos que no pertenecemos…

I: Mmm…

P: Entonces, por eso cuando te digo que siento que vivo donde la suegra es más o menos… con la suegra y todo incluido…

I: Claro….

P: Con la suegra y…

I: O sea, en la dinámica… de la familia también

P: Sí, con la suegra sintiéndote que eres un estorbo, que estás molestando, que… que… que… los estás haciendo sentir incomodos… que… ya no es lo mismo, que en un principio si eras útil pero ya ahora no… Entonces tenemos esa dinámica que yo creo que es la mayoría de todos los que migran…

I: Mmm…

P: Aunque tengamos familia acá no es lo más cómodo…

I: Claro

P: No es lo más cómodo…

I: Y el tema de vivienda, por ejemplo…

P: Mmm… bueno, si solicitamos la ayuda acá, pero están tardíos…. Entonces, imagínate, si a mí… me hubiesen sacado de casa de mi hermano, no sé dónde estuviéramos nosotros ahora…

I: Mmm…

P: Hubiésemos tenido que pedir otro tipo de… de ayuda.

I: Mmm…. Porque uno de los temas que también estamos hablando es que… eh… no existe como una… un acompañamiento de ningún tipo por la situación de… de solicitud de refugio…

P: No…

P2: Nada…

I: A parte de… el… el tema más burocrático ¿no?

P: Exacto

I: Que me contaban en el fondo…

P: No, no lo hay… O sea, si tu no vienes y pides la ayuda… Y si de repente la hay… no hay más acompañamiento, más que un apoyo psicológico o de repente algo social… que donde te dicen, bueno ten paciencia…

I: Mhm…

P: Ten paciencia porque me están echando donde estoy, ten paciencia pero no tengo para la comida, ten paciencia pero no, no… no tengo como buscar trabajo porque no tengo dinero… A ese nivel de… de desasistido si estamos…

I: Y ¿Cómo se relacionan con el resto de la comunidad venezolana? Porque yo me imagino que también estos conflictos que hay allá… se reproducen un poco acá

P: Sí…

I: ¿O no? El ser refugiado cambia la relación con los…

P: Bueno, nosotros…

P2: No….

P: No tenemos contactos refugiados venezolanos, no… si tengo muchoso amigos venezolanos ac´… bueno, unos cuantos amigos, pero estamos en la misma situación…

I: Mmm….

P: Estamos en depresión… este… estamos en añoranza…

I: Mmm…

P: La añoranza yo creo que tiene que ver más con la estabilidad con la que uno tenía… Yo tengo acá una compañera y colega… una amiga y colega, que ella me decía… estoy comiendo mal, yo no comía mal… por último yo a mis hijos les podía dar comida allá bien… Claro, ella está añorando la estabilidad que tenía…

I: Mmm…

P: Pero el país que nosotras dejamos no es el mismo país que tenemos ahora, entonces, en algún momento ella se vio encontrada con la posibilidad de que no les iba a tener que dar de comer a sus hijos…

I: Mmm…

P: Pero es la añoranza… es añorar estar tranquilo… O sea, yo… este… he tenido… eh… digamos que un carrusel emocional porque, justamente, pues uno no está ahí… en algún momento yo pensé en devolverme, o sea, dejarlo a él estabilizado acá, salir de casa de mi hermano, dejarlo a él estable y yo devolverme…

I: Mmm…

P: Devolverme porque, no nos podíamos mantener los dos, por lo menos en última que él estuviera fuera… Yo… me entregaba… o sea, es así como si alguien tiene que morir, no importa, yo me sacrifico… Pero… este, sí… ha sido bien difícil, bien difícil… Porque Chile no es un país fácil… Ni social, ni económica, ni… No es fácil… entonces, claro nosotros acostumbrados a ese otro desastre… Ni siquiera estamos hablando del orden, sino… la idiosincrasia…

I: Mmm…

P: De repente uno se toma otras cosas más tranquilo… otras no…

I: Mhm…

P: Acá es como lo contrario… Los… muchas cosas se toman tranquilo donde nosotros somos más serios… y donde somos nosotros más… rochaleros, ustedes son más serios… Entonces así como… hay que acostumbrarse ¿no?

I: Mmm….

P: Eso es… yo creo que lo que más impacta…

I: Y que de alguna manera también… eh… el hecho de estar en un contexto distinto… tus propias capacidades ya no son valoradas como antes ¿no? Eh…

P: Yo le comentaba a la chica de… de asistencia social, que yo nunca había sido tan rechazada en mi vida…

I: Mmm…

P: O sea, a mi nunca me costó tanto conseguir trabajo como ahora…

I: Claro

P: Porque si no estoy… sobrevalorada, no estoy… tampoco… dentro de lo esperado…
I: Claro…

P: Entonces nunca me han rechazado tanto.

I: Mmm…

P: I sea, por lo general, yo conseguía trabajo en el momento en que lo buscaba…

I: Claro…

P: Pero… es así como no, no califica… ¿Cómo que no califico? Y entonces todo mi currículum… es mucho, quítale, o no eso no es lo que estamos buscando…

I: Mmm…

P: Viste que ahora la dinámica de… perdón, de… este cuando buscan trabajo, las ofertas laborales son unas cosas tan irreales… O sea, recién graduado con cinco años de experiencia máster y… No les da para cumplir todo esto, porque ni siquiera yo lo tengo…

I: Claro…

P: Entonces son… a eso nos hemos tenido que enfrentar

I: Mhm…

P: A él también le ha costado… Hay personas que dicen, mira en quince días yo conseguí trabajo… No sé como lo han logrado, porque nosotros…

I: ¿Quizás también tiene que ver con los tipos de trabajo a los que se aspiran?

P: Mira, desde vendedor… hasta especialista… O sea, yo no me… no… yo he buscado hasta de limpieza…

I: Mhm…

P: Pero claro, cuando me ven me dicen, no, tú no limpias…

I: Mmm cuando te ven…

P: Y si es verdad, yo no limpio…

I: Si…

P: O sea, porque en realidad…. Estamos como en una… eh… posición en que, si yo voy a buscar de mucama me dicen “tú no tienes experiencia de mucama”

I: Mmm

P: “Yo necesito a alguien con experiencia” ¡Y es verdad!

I: Claro

P: O sea, no me pueden dar la oportunidad para yo aprender… Entonces… si es de limpieza me dicen “no, tu no… tu no limpias”

I: Claro…

P: Y si busco en mi área, me dicen… "No, tu no sabes esto, tú estás para gerenciar, pero no tengo para ofrecerte de gerencia"

I: Mmm

P Entonces… es así como, pero dame lo que sea… "Pero lo que sea tu no lo sabes hacer" Y es verdad… Entonces… es así como que wow, todo lo que uno estudió, todo lo que uno… no aplica.

I: Mmm….

P: Afortunadamente ahora estoy en el área…

I: Okey

P: Pero no ganamos lo que yo quisiera, pero… estoy en el área

I: Mhm…

P: Eso me empieza a abrir puertas…

I: Y también el hecho de estar ocupados ¿no? De poder salir en la mañana a hacer algo…

P: Salir de la casa

I: Me imagino…

P: Y volver de noche, no importa así nos quedemos en un mall…

I: Mhm…

P: No importa, pero llegar lo más tarde que se pueda… Con lo cual eso también nos genera un conflicto, porque entonces empiezan las… los… los… ¿cómo es que dicen ustedes? Los… alegar… qe solamente estamos utilizando la casa para dormir…

I: Mmm… las quejas…

P: Entonces, no… no hay nada que hagamos bien… nada que se haga que esté bien…

I: Mmm…

P: Nada, todo lo que estamos haciendo está mal…

I: ¿En qué zona están viviendo? En Santiago…

P: En Maipú

I: En Maipú…

P: Sí… en Maipú… pero es por la dinámica familiar que hay allí…

I: Sí…

P: Entonces… bueno….

I: ¿Y tienen acceso a temas de salud? A parte de la… salud mental acá que están yendo a terapia…

P: Sí, afortunadamente sí, hemos ido al consultorio… Y excelente, te digo, yo no tengo quejas, o sea es como una clínica…

P2: En eso el gobierno chileno si… tuvo este… te ponen FONASA.

P: ¡Mmm!

P: O sea, tienes tu rut y ponen FONASA (no se entiende muy bien min 57:30)

P: Claro, o sea…

I: Y a ustedes les dieron rut a penas… eh… sacaron la solicitud de asilo ¿No?

P2: Eh, estamos en la visa y después te entregan tu rut

P: Un mes después

I: Okey

P: Un mes después de…

I: No fue tan largo entonces obtener el rut…

P: Sí, fue largo…

P2: Si, fue largo, porque si tú no pones…

P: O sea, desde que tú haces la solicitud hasta que te estampan la visa es un mes…

I: Ah, okey

P: Y luego desde que te estampan la visa hasta que haces el proceso, es…

P2: Hasta que te entreguen el plástico…

P: Te entreguen el rut, es un mes…

I: AH, okey entonces estuvieron dos meses sin este famoso rut que sirve para todo…

P: Exacto… para pedir trabajo y todo esto… es largo.

I: Calro…

P: No es inmediato

I: Claro… claro que sí… Pero ya teniendo rut pudieron acceder a… a Fonasa

P: Inclusive desde antes… solo por ser migrante con tu pasaporte…

I: Ya…

P: Entras en una… calidad de indigente

I: Mhm…

P: Pero… desde un principio a nosotros nos atendieron, nos atendieron muy bien, así que…

I: ¿Y fueron a buscar atención de salud? O sea…

P: Sí…

I: ¿Fueron al consultorio?

P: Sí, porque yo tengo… eh… tiroiditis de Hashimoto, entonces tengo que estarme…

I: Controlando…

P: Controlada

I: Y allá lo tenías… pudiste acceder… ¿tenías control de esa enfermedad todo el tiempo?

P: Sí, yo lo hacía… en Venezuela lo hacía de manera particular…

I: Ya…

P: También estaba la parte… este… social, pero ya estábamos llegando a un punto que la hormona no se conseguía…

I: Claro, porque yo sé que había, hay una crisis enorme en términos de acceso a medicamentos… Entonces, ahora están llegando muchas personas venezolanas que no tienen tratamiento hace mucho tiempo.

P: Exacto, cuando yo me vine todavía se conseguía el tratamiento… que yo tenía. De hecho, yo había dejado mi tratamiento allá porque yo regresaba a los tres meses, y lo tuve que regalar…

I: Mmm….

P: O sea, regalar eso para que lo aprovechen…

I: Claro…

P: Pero yo inclusive tenía mi tratamiento…

I: Y eso tuviste que acercarte acá, para buscar el tratamiento acá…

P: Sí…

I: ¿Y cómo fue esa experiencia? Pudiste…

P: Muy bien, si de hecho… este la doctora que me… recibe a mí, una venezolana…

I: Mhm…

P: Y… ella me… incluyó en un proyecto… que se llama GES…

I: Sí… las enfermedades GES

P: Sí, esa. Entonces yo ya estoy suscrita en el plan, con lo cual me garantizan la hormona…

I: Ya, y de lo… el tratamiento es gratis de la…

P: Sí, si

I: Claro, porque es GES

P: En Venezuela también lo tenemos

I: Mhm…

P: Pero… como te digo, yo podía hacerlo particular porque tenía mi ingreso, acá no…

I: Claro… no, por eso…

P: Acá me tocaba comprar… hasta que pueda mejorar mi estado…

I: Mhm…okey. Y entonces eso implica que vas mensualmente…

P: Sí, siempre tengo un control y… y… según como me sienta si voy mensual, o voy cada tres meses, porque si me estoy sintiendo bien con la dosis que tengo, no es necesario ir mensual…

I: Okey

P: Tengo que ir mensualmente a retirar…

I: A retirar la medicación

P: La medicación… Pero el control, por ejemplo, tengo anual. Ahora si yo me empiezo a sentir mal… tengo que ir antes y avisar que me hagan exámenes… El control digamos que es tomar… los exámenes y ver que todo esté… dentro de lo esperado.

I: Mhm… Okey. ¿Y entonces cuales creen4 que son las principales necesidades que tienen hoy en día los refugiados en Chile? ¿Cuáles son las… lo que ustedes sienten de su experiencia que son las cosas más importantes que habría que resolver respecto a esto…?

P: Sí, lo que tú hacías referencia, el acompañamiento yo creo que es primordial… Por lo menos un seguimiento de como estamos y en que condiciones estamos…

I: Mmm

P: Pero eso no se hace…

I: Mmm…

P: O sea, ellos asumen tu palabra y ya está… Entonces, por ejemplo, si yo no vengo y dugo que tengo este problema familiar… no sé enteran que yo estoy pasando por un proceso… refugiado bien difícil…

I: Mhm…

P: Porque se supone que yo estoy… saliendo por unas condiciones precarias y estoy tratando de que no las tenga acá…

I: Mmm…

P: Y… ni se enteran…O sea, si yo no vengo y digo, mira estoy mal…

I: Mmm…. O sea, fuiste tú la que buscaste ayuda…

P: Sí… No… no están… Creo que no tienen la infraestructura para hacer seguimiento como está el refugiado…

I: Mhm…

P: Si de verdad está recibiendo una atención…

I: U por ejemplo…

P2: Es más, al principio, en Latinoamérica nunca se había visto estos movimientos migratorios tan… tan…

P: Tan fuertes…

P2: Tan fuertes… tan altos de personas salientes…

I: Y todas en condiciones distintas ¿no?

P2: Exacto…

I: ¿Y en el consultorio nunca pesquisaron esto tuyo?

P: No, uno informa, pero es así como ah, ya… Mira somos solicitantes de refugio: ya…

I: Tú lo explicitaste, pero por tu voluntad… ¿Nunca te preguntaron?

P: Ahá… Entonces más que todo lo toman en cuenta para saber en donde te ponen… Entonces, ah bueno sí, eres indigente… ah, okey…

I: Okey…

P: Pero… el acompañamiento con asistencia social no… al menos que tu lo pidas, o sea, no te lo rechazan, porque tampoco es….

I: Claro… tampoco te lo ofrecen

P: Exacto.

I: Ni tampoco… tampoco derivación a salud mental…

P: No.

I: ¿Nunca te propusieron eso?

P: No, eso lo conseguí acá cuando vine con la asistencia social… Que nosotros venimos y pedimos la… la cita con la asistente social, porque ya estábamos en un punto en que no conseguíamos trabajo… La situación en casa estaba muy mal…

I: Mmm…

P: Entonces, ellos ahí se dieron cuenta de mi estado… y aes cuando me derivan con ella…

I: Y ahí te derivan…

P: Mhm…

I: Mhm… Claro, porque… esto sucede gracias a que tú te mueves, pero hay muchas personas que no logran ni siquiera, porque están en un momento aún más dramático, que aún no se movilizan en búsqueda de alternativas ¿no?...

P: Sí… Y… bueno, sabemos que los que migran tienen… sufren, llegan a sufrir de un complejo… el complejo de Ulises…

I: Mhm…

P: Y no, no veo que hagan seguimiento tampoco del aspecto psicológico… Porque migrar es un impacto… como te digo, o sea, es… es tan traumático que… mucho más traumático evidentemente, como cuando te mudas de tu casa a casa de otra persona… con la que no tienes lazos familiares… o… que no es tu entorno. Entonces, eso… eso genera un trauma… Y acá por supuesto se amplifica…

I: Claro…

P: Porque estás en otro país, otra idiosincrasia…

I: ¿Y han logrado generar vínculos con chilenos? Han sido… ¿Se sienten recibidos por la comunidad chilena?

P: Sí… sí… O sea, hemos tenido de todo…

I: Mhm…

P: De todo… Hemos tenido acceso a muy buenas personas, que inclusive nos ayudan… Nos ayudan más que la familia

I: Mmm…

P: Como también hemos tenido en la parte de… de… este…. Xenofóbica…

I: ¿Sí?

P: Que nos han insultado… Sí, a mí sí me han insultado… Bueno…

I: Es muy fuerte porque…

P2: Sí, exacto…

I: Porque…

P2: No tanto, pero un poco

P: Pero digamos que (ininteligible) una… Pero sí lo hemos tenido…

I: Mmm… Pero es muy fuerte, porque generalmente se piensa que eso sea solo cuestión de racismo… Pesando en las personas, por ejemplo, haitianas, o más morenas ¿no? Pero pensando que ustedes en apariencia…

P: Nos (ininteligible) por el acento (Interrumpe entre medio, no se entiende lo que dice min 01:03:48)

I: No parecen en imagen… ni se ven como personas tan distintas…

P: En lo que te sienten es el acento, nosotros evitamos a veces hablar… Porque en lo que te sienten el acento… ya de una vez se ponen como alerta. Obvio, es lógico, también uno entiende que acá no estaban acostumbrados a la migración…

I: Mmm…

P: No… y menos de este tipo. Pero en seguida uno siente… si, si… he tenido la sensación de que me tengo que callar, de que no tengo que hablar.

I: Mmm….

P: Y… más cuando te comparan con colombianos… Que los colombianos no son muy bienvenidos…

I: Mmm….

P: Entonces, sí… si he sentido…

I: Claro, de alguna manera en este mundo desconocido, que es Chile ¿no? Se pone como una única etiqueta como migrante…

P: Exactamente

P2: Exacto

I: Y…

P: Sí… Claro y… bueno, está bien, muchas veces hubo un tiempo en que lo que les llegaba acá no era bueno…

I: Mmm…

P: Y es lógico que tengan cierta… Y la manera también como la migración tampoco fue buena… porque no fue algo controlado, fue que de repente les llegó… y… la gente empezó ver personas ra… distintas, no estaban acostumbrados…

I: No…

P: Por así decir…

I: No y lo que tu dices, que todo lo que se les dio para afuera como un Chile exitoso, en el fondo, acá ustedes llegan a ver los problemas que tiene ¿no?

P: Sí…

I: Este modelo… las desigualdades que hay…

P: Sí…

I: Como la gente también… chilena, que ha vivido siempre acá ha tenido muchos problemas para sobrevivir…

P: Sí…

I: O sea… los sueldos bajos que hay, lo caro que es…

P: Sí, sí, sí, sí… terrible… sinceramente yo no sé como hacen… Yo no sé… Y sí… mucha desigualdad y… sí… ocurre esta dicotomía que tú dices… bueno, son consumistas ¿pero de dónde sacan?

I: Mmm…

P: ¿De dónde sacan? ¿Cómo se sostienen? Entonces… la… el marketing agresivo, el capitalismo… o sea, nosotros que ve… yo ahora que vengo a caer en cuenta que Venezuela siempre fue socialista… siempre… Tú dices… o sea, sí…

P2: Si es que el…

P: ¿Cómo hacen para…? Este… O sea, este bombardeo de compra, compra, compra, compra… y… tarjetas de créditos, y la gente se endeuda… Y pasan a dicom y tú dices, pero ¿cómo? No es que allá no lo tengamos… pero lo ves menos…

I: Claro…

P: Lo ves menos… porque no hay tanto bombardeo… O sea, imagínate, si Chávez decía que era un capitalismo salvaje, nunca habría vivido aquí…

I: Jajaja

P2: Un poco…

P: Esto sí es capitalismo salvaje… O sea, en lo… la primera impresión que yo tuve fue con los planes de celular, que no duran nada… Entonces te lo venden por dos o tres días, y tu dices no, pero sí… Yo lo quiero para un mes…

I: Jajaja

P: Pero... por este precio ¿cómo lo vendes por dos, tres días?

I: Claro…

P: Entonces es así como… un exabrupto… Y con todo eso tú ves gente… compra, compra, compra… Y no entiendes de donde sacan o ¿cómo pueden sobrevivir teniendo en dicom…?

I: Mhm…

P: No sé…

I: Sí, sobregiradas… hiper giradas…

P: Sí… Entonces le exigen tarjeta de crédito… Y la gente sigue endeudándose… Y… y… y… También el tema de la comida, o sea…

P2: Y los servicios también…

P: Exacto, que… eh… o sea… no… no es que yo rechacé a las personas obesas, pero te das cuenta de… Lo mucho de todo…

I: Mmm…

P: Entonces tú dices… (ininteligible min 01:06:56) Paren un poco… Tampoco la vida es así…

I: Claro, es la traducción del consumismo también en la alimentación…

P: Eso, exacto, entonces tú ves en las personas que comen mucho, muchos niños obesos… Mucha azúcar… mucho, mucho, mucho, mucho… Tú dices ya paren, paren, paren, pero tampoco la cosa es de… En cambio, nosotros no venimos de un país ordenado, esta bien… Pero tampoco acá lo están haciendo bien en ese… eh… exacerbado gasto, consumo…

I: Mmm…

P: Tampoco está bien… En un momento les va a reventar feo…

I: Sí… Claro, porque además ustedes vieron que pasa cando las economías revientan ¿no?

P: Exacto

I: Está economía… También en este momento todos tienen capacidad de consumo, pero tienen… hayun nivel de endeudamiento monstruoso…

P: A parte de eso la enfermedad… Entonces tú ves tú… O sea, que Chile sea uno de los países que tenga mayor… índice de diabetes infantil… Y la gente no quiere entender… Diciendo que el chileno come así, no, están envenenados… Están envenenando a los niños… Entonces es terrible, porque va a llegar un momento en que… el gobierno chileno no va a tener dinero para tanto…

I: No, claro…

P: Entonces es terrible… de verdad… Eso… estamos viendo como que los dos extremos…

I: Mmm… Sí, porque ustedes salieron de un… de un (ininteligible) que se veía como que se había destruido el capitalismo… y jaja por otra parte están viendo… lo que…

P: Esto es un capitalismo salvaje… Y la gente no tiene…

I: Porque el proceso chileno fue al contrario… En el fondo, nosotros anhelamos lo que se perdió, cuando todavía había un rol del estado más regulador ¿no?

P: Exacto…

P2: Sí… que se encargaba de…

P: Como más de arbitraje… sí…

P2: Sí, más control de la economía…

P: Exacto… Que eso esta bien, hasta cierto punto…

I: Claro…

P: Malo es como ocurre en Venezuela, como ya tiene el poder absoluto…

I: Claro… Y además los niveles de corrupción también…

P2: Mhm…

P: Sí…

P2: Claro, y el control del estado, porque el estado tiene…

P: No tiene… Exacto…

P2: (ininteligible min 01:08:40) Ni controlar ni regular…

P: Exacto… Eh… como auditar…

P2: Exacto

P: Más que controlador, auditar… Meterse y ver que está pasado… y ya.

I: Mmm…

P: Y llamar la atención…

I: Porque después hacen lo que quieren jaja… Las empresas realmente hacen lo que quieren... (No se entiende bien esta parte min 01:08:55)

P: Nos hemos dado cuenta, de verdad… O sea, y el riesgo de esto, yo sé que no, yo sé que no va a pasr acá… Pero el riesgo de esto es cuando la gente se empieza a resentir… por los excesos…

I: Mmm….

P: Entonces cuando empieza a votar estas lacras populistas que te dicen yo soy el mecías que te viene a salvar, y no es verdad…

I: Mmm…

P: No es verdad… Entonces ahí es cuando se tergiversa…

I: Claro, se hacen estos giros y… empiezan a cambiar…

P: Sí, porque estos son… estos son encantadores de serpientes… O sea, son los típicos Robin Hood…

I: Mmm…

P: O sea, que… empiezan a crear el enemigo… externo… y te dicen que ellos son los culpables, resulta que no… Es terrible… O sea, podríamos hacer una ¿teleserie? (ininteligible min 01:09:37) jajaja

I: Jajaja

P2: Jajaja

I: Oye muchas gracias…

P: No, tranquila…

(Fin del audio min 01:09:41)

XX

COLOMBIA

XX tiene 62 años y viene de Cali, Colombia. Llegó a Chile en Marzo del 2017. Vive en Estación Central, en una pieza con su marido, que estuvo por largo tiempo recuperándose de un accidente, una fractura a la rodilla que sufrió a penas llegando a Chile. Zeneida dice que prefiere ocupar el nombre que le dio su último patrón, Amparo, pues le decían que su nombre era muy feo y no se entendía. Por ello en el consentimiento firmó como Amparo.

Desde su llegada a Chile trabajó como empleada doméstica en una casa "del barrio alto", seis meses puertas afuera y luego seis meses puertas adentro. Hace poco renunció por las tensiones y el cansancio que le producía vivir puertas adentro y las necesidades de su marido, que una vez recuperado recomenzó con trabajos de cargador y pioneta.

Zeneida nació en la Vega, en la región de Tolima, en el Cauca colombiano. Esta zona vio nacer al llamado "conflicto colombiano" y Zeneida fue testigo de ese proceso desde su infancia. Cuando era pequeña vio en varias ocasiones llegar a fuerzas de las FARC a acampar en los patios de las casas de su comunidad "llegaban y dormían ahí, nada preguntaban, pedían comida y la familia de uno tenía que darle los animales, la comida, luego sin decir nada se iban". Los episodios de violencia marcaron su infancia, por el temor a que desaparecieran los hijos hombres, situación que sucedió con dos de sus hermanos. Ellos probablemente fueron reclutados por las FARC, y no se supo de su destino sino hasta hace un par de años en que uno se comunicó con su familia. Los enfrentamientos y tiroteos eran frecuentes así como allanamientos y ajusticiamientos en las calles. Conoció a su marido en La Vega, pero él al provenir también de una zona marcada por el conflicto, la incitó a migrar a Cali, la ciudad más cercana. Allí Zeneida se desarrolló como trabajadora textil, consiguió un trabajo estable, con un empleador que la valoraba y quería. Por ello ella nunca pensó en migrar y pensaba estar con sus hijos en la ciudad. Tuvo 3 hijos, dos hombres y una mujer, ellos son adultos actualmente y uno de ellos, decidió iniciar la carrera militar e insertarse en el ejército. Esto Zeneida lo sintió como la pérdida de un hijo, pues debido a su acción dentro del conflicto él desaparece por meses y "no se sabe dónde está, si está vivo o muerto", el hijo más pequeño en cambio ha tratado de llevar adelante una actividad comercial, sin mucho éxito. Ninguno de sus hijos pudo estudiar y ella siempre ha temido por sus vidas, dado lo que sucedió con sus hermanos, por eso casi no volvió a Tolima y evitó que los hijos tuvieran relación con su comunidad de origen.

La partida de Zeneida se debió a los hostigamientos que empezó a sufrir su marido, debido probablemente a la participación de su hijo en el ejército. Trabajó como taxista y sufrió amedrentamientos y luego, trabajando como uber, tuvo diversos intentos de asalto y la quema de su carro. Por este motivo emprenden viaje a Chile, donde decían que la situación sería mejor. Los hijos lo entendieron e impulsaron su partida que realizaron por via terrestre pasando por Ecuador y Perú, para luego entrar a Chile por Arica. "Fue muy largo, semanas viajando, pasando por lugares peligrosos, también nos robaron antes de entrar a Chile, en Perú nos robaron y ya no teníamos nada, no más un número telefónico que le quedó marcado a una señora que venía en el bus, porque me había prestado el celular."

Zeneida no tenía idea de la existencia del refugio como posibilidad, pensaba más bien en encontrar trabajo que decían en Chile abundaba. Sin embargo, al llegar y sin encontrarse con ninguna posibilidad de alojamiento, se dirigieron a una iglesia en la que los dejaron dormir e invitaron a seguir algunas de sus actividades. En esa instancia escucharon hablar del refugio y una persona de la iglesia los orientó a FASIC. Ahí recibieron apoyo económico con el que arrendar una pieza y luego fueron siguiendo los pasos para la solicitud de refugio. Hoy, a su decir, están con el refugio reconocido (no se entiende bien si es solicitante o tiene ya el refugio) y ha podido trabajar en labores de limpieza. "Una mujer vieja como yo, qué otro trabajo va a tener aquí". El accidente de su esposo al llegar a Chile dificultó aún más las cosas y la obligó a pasar de asesora puertas afuera a puertas adentro, dadas las necesidades económicas. Sin embargo, a pesar de que relata haber tenido una buena experiencia con sus patrones, decidió renunciar dadas las dificultades de desplazamiento y la imposibilidad de estar en casa que implicaba este tipo de trabajo. Hoy se encuentra sin trabajo, no ha estado inscrita en ningún consultorio ni se ha hecho chequeos de salud. Sin embargo, con la experiencia de su marido, que tuvo atención de salud en hospital (no se acuerda cuál) supo del funcionamiento del sistema de salud chileno. Tiene dolores y cansancio general pero no está siguiendo ningún tipo de tratamiento. Hay episodios de llanto durante el relato, prefiere no ser grabada y sus testimonios son, a ratos, confusos. Se excusa por no poder dar muchos datos precisos, se lamenta de la nostalgia de su familia, de sus nietos que han nacido durante este periodo y que no ha podido conocer.
